# Structurally diverse polyketides and alkaloids produced by a plant-derived fungus *Penicillium canescens* L1

**DOI:** 10.1007/s13659-025-00503-0

**Published:** 2025-04-03

**Authors:** Wei-Ye Wu, Xun Wei, Qiong Liao, Yi-Fan Fu, Lei-Ming Wu, Lei Li, Shu-Qi Wu, Qing-Ren Lu, Fang-Yu Yuan, Dong Huang, Zhang-Hua Sun, Tao Yuan, Gui-Hua Tang

**Affiliations:** 1 School of Pharmaceutical Sciences, Sun Yat-sen University, Guangzhou, 510006 China; 2 Laboratory Animal Center, Sun Yat-sen University, Guangzhou, 510006 China; 3 Guangdong Provincial Key Laboratory of Utilization and Conservation of Food and Medicinal Resources in Northern Region, Shaoguan University, Shaoguan, 512005 China; 4 The Laboratory of Effective Substances of Jiangxi Genuine Medicinal Materials, College of Life Sciences, Jiangxi Normal University, Nanchang, 330022 China

**Keywords:** Endophytic fungus, *Penicillium canescens*, Polyketides, Alkaloids, Cytotoxic activity

## Abstract

A series of structurally diverse polyketides (**1–3**), sesterterpenoids (**24** and **25**), and alkaloids (**26–34**) were isolated from the fermentation of a plant-derived fungus *Penicillium canescens* L1 on solid rice medium. Among these secondary metabolites, penicanesols A**–**G (**1–7**) were new structures, which were elucidated by NMR, HR-ESI-MS, ECD calculation, and X-ray diffraction. Penicanesol A (**1**) represented a rare dimer derived from phthalan derivatives, characterized by a 5/6/6/6/5 heteropentacyclic core. The bioassay on the NCI-H1975 cell model showed that two compounds had good cytotoxic activities, and the most significant activate compound **13** had an IC_50_ value of 4.24 ± 0.13 μM, more than the positive control drug (12.99 ± 0.13 μM).

**Graphical Abstract:**

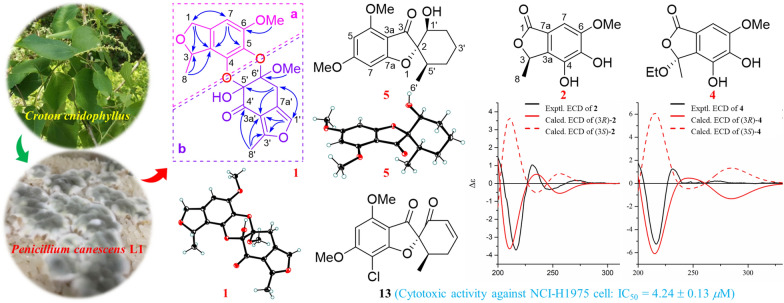

**Supplementary Information:**

The online version contains supplementary material available at 10.1007/s13659-025-00503-0.

## Introduction

*Penicillium* is a widely distributed filamentous fungus, which has great potential to produce novel metabolites with diverse biological activities. So far, a variety of structurally diverse secondary metabolites including polyketides, alkaloids, sterols, terpenoids, and macrolides have been discovered from this genus, and most of them have multiple biological activity potentials such as antibacterial, cytotoxic, anticancer, antioxidant, antifungal, and antiviral properties [1–5]. Drugs like penicillin, mevastatin, griseomycin, and cyclosporin are star molecules produced by the *Penicillium* genus.

The gray mold fungus *Penicillium canescens* is a common microorganism in the genus *Penicillium*, which is distributed in soil, ocean, and plant bodies. Previous studies have investigated the secondary metabolites of this strain and discovered various molecules with novel structures and diverse activities, including aromatic polyketides, terpenes, azathiones, anthraquinones, and alkaloids. Their pharmacological effects involve antibacterial, anticancer, and *β*-glucosidase inhibition [6–15].

**Fig. 1:**
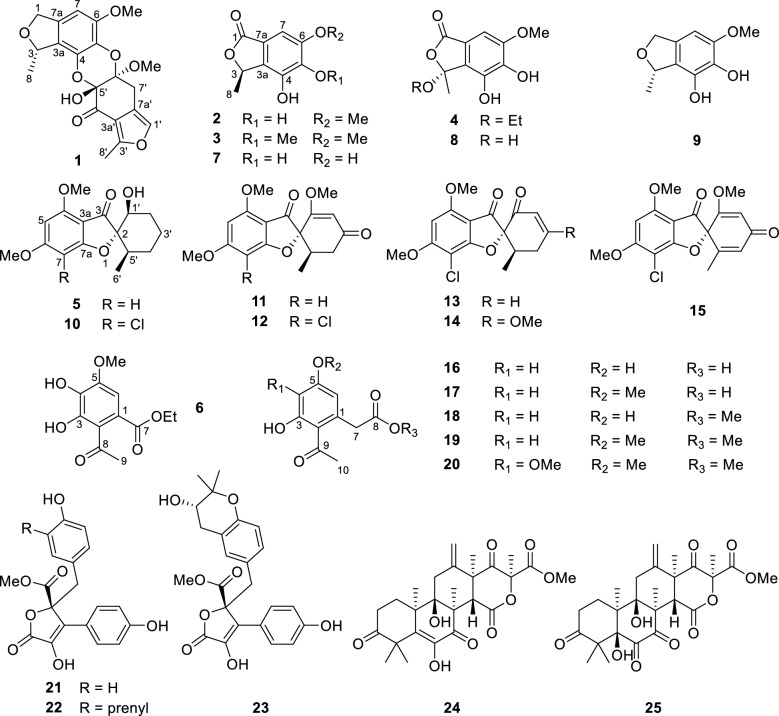
The structures of polyketides (**1–23**) and sesterterpenoids (**24** and **25**) from *P. canescens* L1

**Fig. 2:**
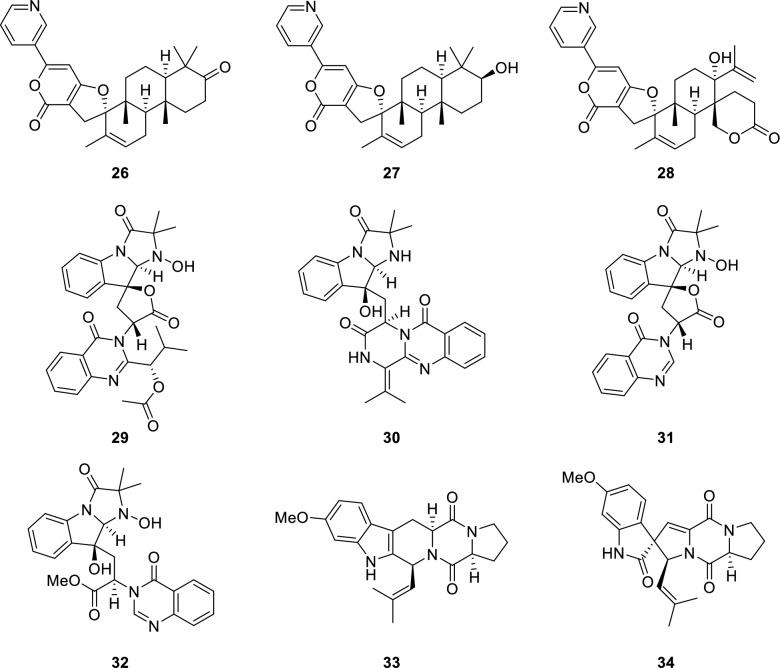
The structures of alkaloids (**26–34**) from *P. canescens* L1

Our research group had carried out the investigation on the chemical composition of the plant *Croton cnidophyllus* for the first time [16]. In order to continue searching for active molecules from endophytic fungi [17, 18], we studied the secondary metabolites of fungus *P. canescens* L1 isolated from the plant of *C. cnidophyllus*. As a result, a total of 34 structurally diverse secondary metabolites, such as polyketides (Fig. 1), sesterterpenoids (Fig. 1), and alkaloids (Fig. 2), were obtained from the fermentation of fungus *P. canescens* L1. Their structures were analyzed by NMR, HR-ESI–MS, ECD, and X-Ray single crystal diffraction data. The selected compounds were evaluated on the NCI-H1975 cell model for their cytotoxic activities. Herein, the isolation of all compounds produced by the plant-derived fungus *P. canescens* L1, the elucidation of new structures, as well as their cytotoxic activities against NCI-H1975 cell were described.

## Results and discussion

Compound **1** was obtained as colorless needles and had a molecular formula of C_20_H_20_O_8_ corresponding to 11 degrees of unsaturation based on HR-ESI-MS data *m/z* 411.1055 [M + Na]^+^ (calcd for C_20_H_20_O_8_Na^+^, 411.1050) and 1D NMR data. The IR spectrum of **1** exhibited absorption bands for the presence of OH (3357 cm^−1^), carbonyl (1705 cm^−1^), and phenyl (1612, 1572, and 1496 cm^−1^) groups. The observed signals in the ^1^H NMR spectrum suggested the presence of one doublet methyl [*δ*_H_ 1.86 (3H, d, *J* = 5.6 Hz)], one singlet methyl [*δ*_H_ 2.50 (3H, s)], two methoxy groups [*δ*_H_ 3.33 and 3.83 (each 3H, s)], two aromatic proton signals [*δ*_H_ 6.54 and 7.41 (each 1H, s)], one isolated methylene [*δ*_H_ 3.73 and 3.85 (each 1H, d, *J* = 15.8 Hz)], and a hydroxymethyl group [*δ*_H_ 4.95 and 5.15 (each 1H, d, *J* = 11.6 Hz)] (Table 1). The ^13^C NMR combined with DEPT spectra showed a total of 20 carbon signals (Table 1), which were classified as one conjugated carbonyl (*δ*_*C*_ 186.9), two hemiketals (*δ*_*C*_ 93.5 and 99.8), eight sp^2^ quaternary carbons (including four oxygenated ones), three methines (including two oxygenated ones, *δ*_*C*_ 80.4 and 137.9), two methylenes (including an oxygenated one, *δ*_*C*_ 73.5), four methyl groups including two methoxys (*δ*_*C*_ 49.7 and 56.8).

**Table 1:** The ^1^H NMR (400 MHz) and ^13^C NMR (100 MHz) data of **1** in pyridine-*d*_5_

No	*δ*_H_, multi. (*J* in Hz)	*δ*_C_, type	No	*δ*_H_, multi. (*J* in Hz)	*δ*_C_, type
1	a 5.15, d (11.6) b 4.95 d (11.6)	73.5, CH_2_	1′	7.41, s	137.9, CH
3	5.52, brs	80.4, CH	3′		159.7, C
3a		124.3, C	3a′		117.9, C
4		137.8, C	4′		186.9, C
5		129.7, C	5′		93.5, C
6		150.5, C	6′		99.8, C
7	6.54, s	98.7, CH	7′	a 3.85, d (15.8) b 3.73, d (15.8)	24.8, CH_2_
7a		133.5, C	7a′		119.7, C
8	1.86, d (5.6)	22.2, CH_3_	8′	2.50, s	14.1, CH_3_
6-OMe	3.83, s	56.8, CH_3_	6′-OMe	3.33, s	49.7, CH_3_

**Fig. 3:**
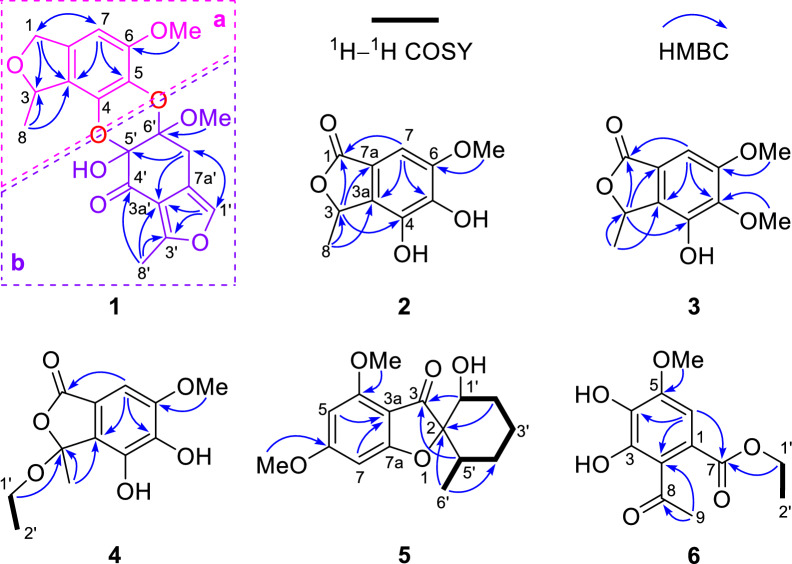
The ^1^H–^1^H COSY and key HMBC correlations of compounds **1–6**

**Fig. 4:**
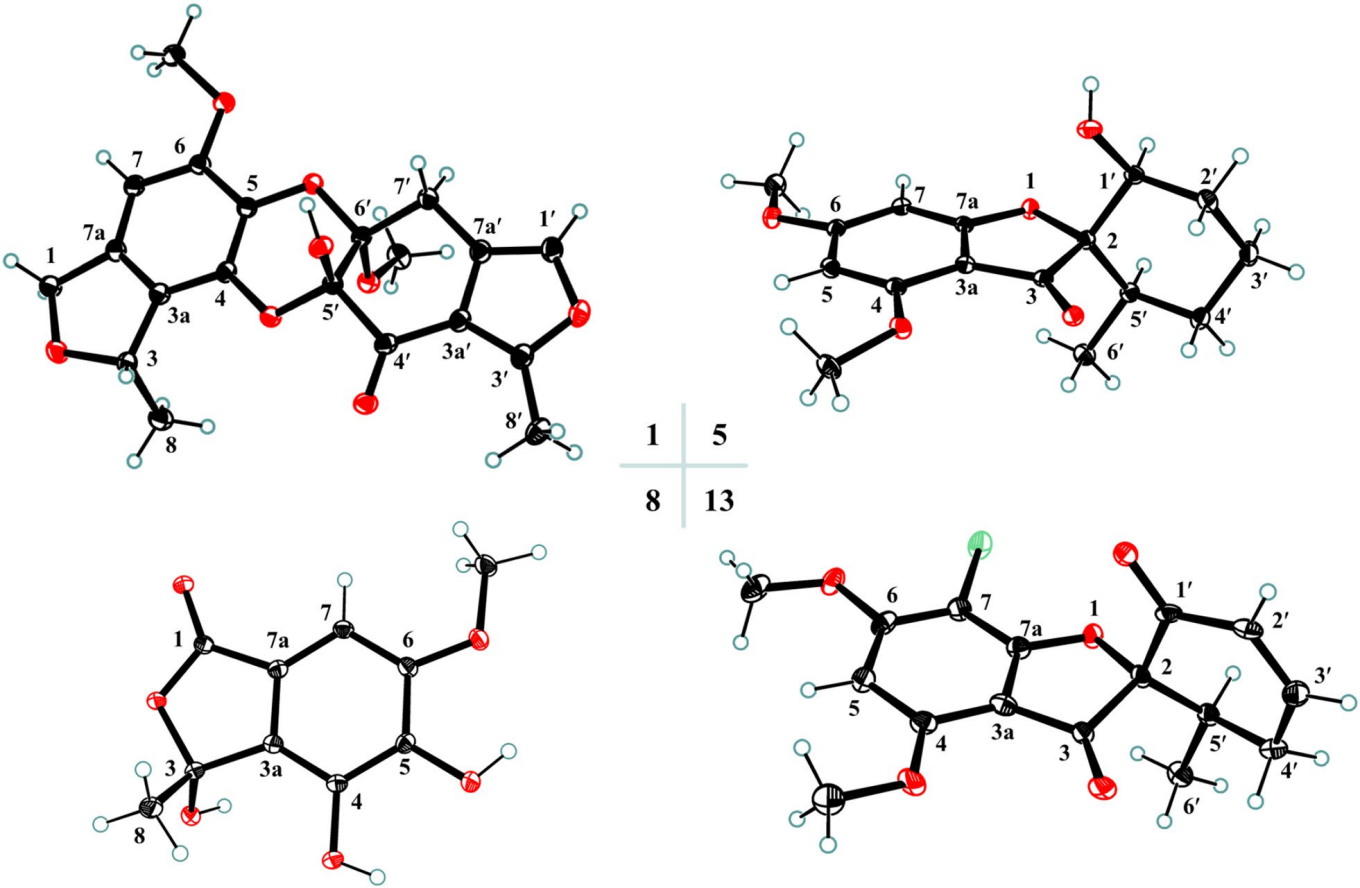
ORTEP drawings of compounds **1**, **5**, **8**, and **13**

Analysis of the HMBC correlations, it could be determined that **1** had two similar 5/6 bicyclic units **a** and **b**. As shown in Fig. 3, the HMBC correlations from H_3_-8 to C-3 and C-3a, H_2_-1 to C-3, C-3a, C-7a, and C-7, H-7 to C-1, C-3a, and C-5, and the methoxy protons to C-6 confirmed that unit **a** was the same as curvulol (**9**) [19]. While another fragment **b** was also determined to have a skeleton similar to fragment **a** by the HMBC correlations (Fig. 3), with the difference being that its 6-membered ring was a non-aromatic ring characterized by an one conjugated carbonyl and two hemiketals and its five membered ring was a furan ring. The connection of units **a** and **b** through C-4–O–C-5′ and C-5–O–C-6′ determined by analyzing its degrees of unsaturation and comparing NMR data of similar compounds such as canescone E [14]. The relative configuration of compound **1** could not be determined by its NOE correlations. Fortunately, the crystal of **1** obtained from the solvent MeOH/DCM (5:1), and subsequent analysis of its X-ray single crystal diffraction data confirmed the structure of **1** including the (3*S*,5′*S*,6′*S*) absolute configuration (Fig. 4).

**Fig. 5:**
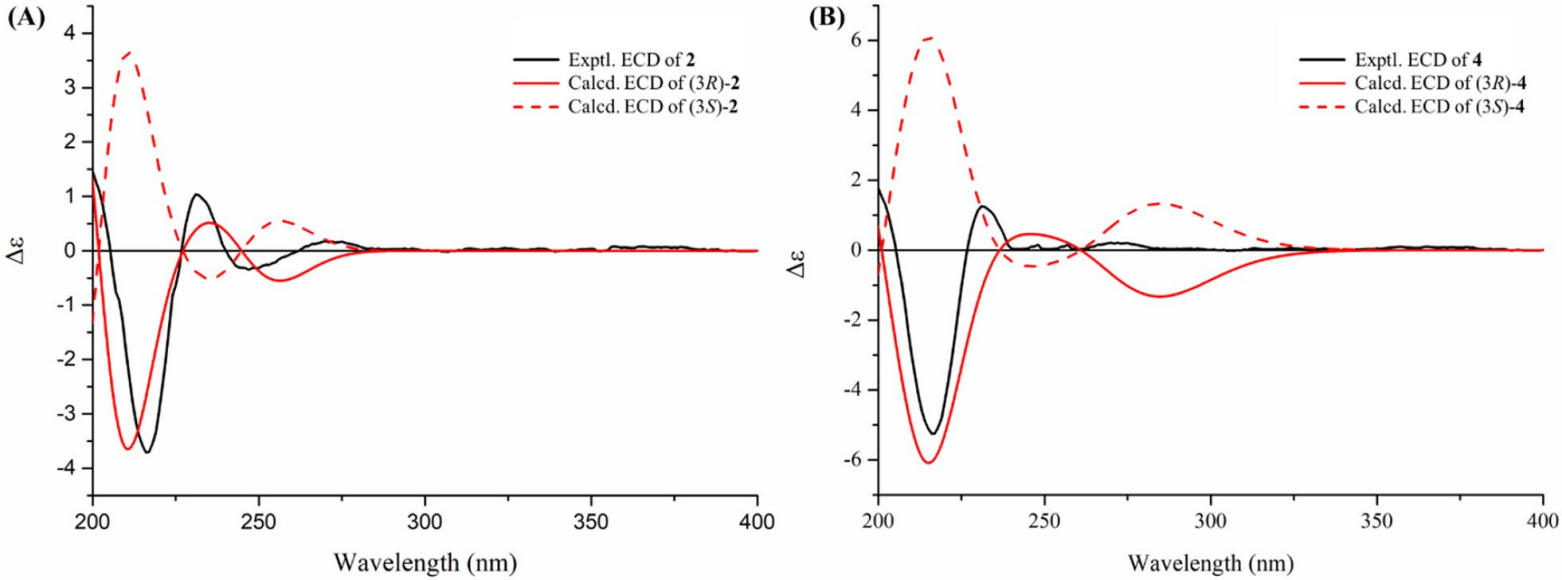
Experimental and calculated ECD spectra (**A** and** B**) of **2** and **4** in MeCN

Compound **2**, a white amorphous solid, had the molecular formula C_10_H_10_O_5_ as determined by the HR-ESI-MS data *m/z* 233.0422 [M + Na]^+^ (calcd for C_10_H_10_O_5_Na^+^, 233.0420) and 1D NMR data. Comparison of its NMR data (Table 2) with those of 4,5,6-trihydroxy-3-methylphthalide [20] indicated that **2** possessed an additional methoxy group [*δ*_H_ 3.89 (3H, s)]. The HMBC correlation of this methoxy protons to C-6 confirmed its position at C-6. The absolute configuration of the only chiral carbon in compound **2** was determined by the ECD calculation method. As shown in Fig. 5, the experimental ECD curve of **2** matched well with the calculated ECD curve of (3*R*)-**2**, which indicated that the absolute configuration of C-3 in **2** was *R*.

**Table 2:** The ^1^H NMR (400 MHz) and ^13^C NMR (100 MHz) data of **2–4**

No	**2**^a^		**3**^b^		**4**^a^	
	*δ*_H_, multi. (*J* in Hz)	*δ*_C_, type	*δ*_H_, multi. (*J* in Hz)	*δ*_C_, type	*δ*_H_, multi. (*J* in Hz)	*δ*_C_, type
1		173.7, C		170.9, C		171.1, C
3	5.50, q (6.5)	78.1, CH	5.52, q (6.5)	76.6, CH		109.8, C
3a		133.9, C		130.7, C		128.7, C
4		140.8, C		143.8, C		141.9, C
5		141.6, C		140.5, C		141.8, C
6		151.1, C		154.0, C		151.9, C
7	6.88, s	99.3, CH	6.97, s	99.9, CH	6.88, s	99.5, CH
7a		116.6, C		121.4, C		118.7, C
8	1.59, d (6.5)	19.5, CH_3_	1.64, d (6.5)	19.3, CH_3_	1.77, s	24.9, CH_3_
1′					a 3.22, dq (9.0, 7.0); b 3.06, dq (9.0, 7.0)	60.7, CH_2_
2′					1.07, t (7.0)	15.4, CH_3_
5-OMe			3.96, s	61.3, CH_3_		
6-OMe	3.89, s	56.8, CH_3_	3.89, s	56.4, CH_3_	3.87, s	56.9, CH_3_

^a^Recorded in CD_3_OD

^b^Recorded in CDCl_3_

Compound **3** was obtained as a white amorphous solid. Its molecular formula was inferred to be C_11_H_12_O_5_ on the basis of the HR-ESI-MS and 1D NMR data. The 1D NMR data of **3** (Table 2) was very similar to that of **2** except for the presence of an additional methoxy signal [*δ*_C_ 61.3; *δ*_H_ 3.96 (3H, s)]. This methoxy group was determined to be located at C-5 by the HMBC correlation from the methoxy protons (*δ*_H_ 3.96) to C-5 (*δ*_C_ 140.5) (Fig. 3). The absolute configuration of **3** was determined by comparison of its experimental ECD spectrum with those of **2** (Fig. S1.2). Both compounds displayed similar Cotton effect, which suggested that compound **3** had the same (3*R*) absolute configuration as **2**. Thus, the structure of **3** was defined as shown in Fig. 1.

The molecular formula of compound **4** was determined to be C_12_H_14_O_6_ by the HR-ESI–MS ion at *m/z* 277.0687 [M + Na]^+^ (calcd for C_12_H_14_O_6_Na^+^, 277.0683) together with its ^1^H and ^13^C NMR data. Its NMR data (Table 2) were similar to those of penicanesin E (**8**) [15], except that **4** had an additional ethoxy group [*δ*_*C*_ 15.4 (CH_3_) and 60.7 (CH_2_); *δ*_H_ 1.07 (3H, t, *J* = 7.0 Hz), 3.22 and 3.06 (each 1H, dq, *J* = 9.0, 7.0 Hz)]. This was supported by the ^1^H–^1^H COSY correlations, and the location of the ethoxy group at C-3 was determined by the HMBC correlation of the protons at 3.22 and 3.06 ppm to C-3. The (3*R*) absolute configuration of **4** was also determined by the ECD calculation (Fig. 5). Thus, the structure of **4** was determined to be the C-3 ethylation product of **8**.

Compound **5** was obtained as colorless needles and its molecular formula of C_16_H_20_O_5_ was determined by the HR-ESI-MS and 1D NMR data. The 1D NMR data of **5** (Table 3) were resembled those of penigriseofulvin E (**10**) [21], with the only difference being the presence of a 1,3,4,5-tetrasubstituted aromatic ring [*δ*_H_ 5.96 and 6.16 (each 1H, d, *J* = 1.9 Hz)] in **5** instead of a pentasubstituted aromatic ring in **10**. The MS data and the key HMBC correlations of H-7 to C-3a and C-5 indicated that **4** was the dechlorination product of **10**. The NOE correlation of H-5′/H-1′ suggested that these two protons were cofacial and arbitrarily designated as *α*-configuration. The relative configuration of C-2 could be the same as **10** by comparison of their 1D NMR data. A single-crystal X-ray diffraction analysis using Cu Kα radiation [Flack parameter = 0.09 (4)] (Fig. 4) further confirmed the structure of **5** including the (2*S*,1'*S*,6'*R*) absolute configuration.

**Table 3:** The ^1^H NMR (400 MHz) and ^13^C NMR (100 MHz) data of **5** and **6** in CDCl_3_

**5**			**6**		
No	*δ*_H_, multi. (*J* in Hz)	*δ*_C_, type	No	*δ*_H_, multi. (*J* in Hz)	*δ*_C_, type
2		95.7, C	1		124.2, C
3		197.0, C	2		118.3, C
3a		107.4, C	3		146.7, C
4		158.5, C	4		136.3, C
5	6.16, d (1.9)	88.3, CH	5		148.5, C
6		169.8, C	6	6.91, s	105.8, CH
7	5.96, d (1.9)	92.6, CH	7		167.7, C
7a		175.7, C	8		204.2, C
1′	3.90, dd (11.9, 4.8)	75.1, CH	9	2.48, s	30.7, CH_3_
2′	*α* 2.30, m; *β* 1.87, m	29.3, CH_2_	5-OMe	3.95, s	56.5, CH_3_
3′	*α* 1.84, m; *β* 1.38, dt (13.1, 3.9)	23.4, CH_2_	1′	4.34, q (7.1)	62.1, CH_2_
4′	*α* 1.99, m; *β* 1.50, m	28.5, CH_2_	2′	1.37, t (7.1)	14.2, CH_3_
5′	1.94, m	38.8, CH			
6′	0.82, d (6.4)	14.9, CH_3_			
4-OMe	3.85, s	56.0, CH_3_			
6-OMe	3.85, s	56.0, CH_3_			

Compound **6** had a molecular formula C_12_H_14_O_6_ determined by its HR-ESI-MS and 1D NMR data. The ^1^H NMR data of **6** (Table 3) showed two singlet methyls including a methoxy [*δ*_H_ 2.48 and 3.95 (each 3H, s)], one ethoxy group [*δ*_H_ 1.37 (3H, t, *J* = 7.1 Hz), and 4.34 (2H, q, *J* = 7.1 Hz)], and an aromatic proton [*δ*_H_ 6.91 (1H, s)]. The 12 carbon signals in the ^13^C NMR and DEPT spectra were assigned by 2D NMR data analysis as a pentasubstituted aromatic ring, an acetyl group, an ester carbonyl group, one methoxy group, and ethoxy group. The HMBC correlations from H-6 to C-4, C-2, and C-7, H_3_-9 to C-2 and C-8, 5-OMe to C-5, and H-1′ to C-7 from H-2 to C-7 confirmed that the acetyl group at C-1, the ester carbonyl with an ethoxy group at C-1, the methoxy group at C-5, and two hydroxy groups at C-3 and C-4 of the aromatic ring (Fig. 3). Thus, the structure of **6** was determined as shown.

The planar structure of compound **7** was determined to be the same as 4,5,6-trihydroxy-3-methylphthalide, a racemic mixture $\left( {[\alpha ]_{{\text{D}}}^{{{25}}} = 0} \right)$ [20], by comparison of their 1D NMR data. The experimental ECD curve of **7** was completely consistent with compounds **2** and **3**, so as their optical rotations (**7**: $[\alpha ]_{{\text{D}}}^{25} = + 34.8$, **2**: $[\alpha ]_{{\text{D}}}^{25} = + 36.9$, **3**: $[\alpha ]_{{\text{D}}}^{25} = + 12.5$), which suggested that the absolute configuration of **7** was also 3*R*. Thus, compound **7** was defined as (*R*)-4,5,6-trihydroxy-3-methylphthalide and named as penicanesol G. In addition, the same planar structure with negative optical rotation $\left[ {[\alpha ]_{{\text{D}}}^{25} = - 25.9} \right]$ synthesized by Zhang et al. was (*S*)-4,5,6-trihydroxy-3-methylphthalide [22].

Twenty-seven known compounds were one phthalide derivative, penicanesin E (**8**) [15]; one phthalan derivative, curvulol (**9**) [19]; six griseofulvin analogues, penigriseofulvin E (**10**) [21], dechlorogriseofulvin (**11**) [23], griseofulvin (**12**) [24], 4′-demethoxyisogriseofulvin (**13**) [24], isogriseofulvin (**14**) [24], and dehydrogriseofulvin (**15**) [24]; five 2-acetyl-phenylacetic acid derivatives, curvulin acid (**16**) [25], *O*-methylcurvulinic acid (**17**) [26], methyl curvulinate (**18**) [27], methyl 2-(2-acetyl-3-hydroxy-5-methoxyphenyl)acetate (**19**) [28], and methyl 2-acetyl-3-hydroxy-4,5-dimethoxybenzeneacetate (**20**) [29]; three butyrolactone derivatives, butyrolactone II (**21**) [30], butyrolactone I (**22**) [30], and butyrolactone-V (**23**) [31]; two sesterterpenoids, terretonin (**24**) [32] and terretonin B (**25**) [33]; three decaturin alkaloids, decaturin D (**26**) [34], decaturin E (**27**) [35], and 15-deoxyoxalicine B (**28**) [36]; four indole alkaloids, tryptoquivaline (**29**) [37], quinadoline A (**30**) [38], tryptoquivaline L (**31**) [39], and tryptoquivaline R (**32**) [40]; and two diketopiperazines, fumitremorgin C (**33**) [41] and 6-methoxyspirotryprostatin B (**34**) [42]. Their structures were identified by comparison of their spectroscopic data (Tables S2.1–S2.16 in Supporting Information) with those reported information.

**Fig. 6:**
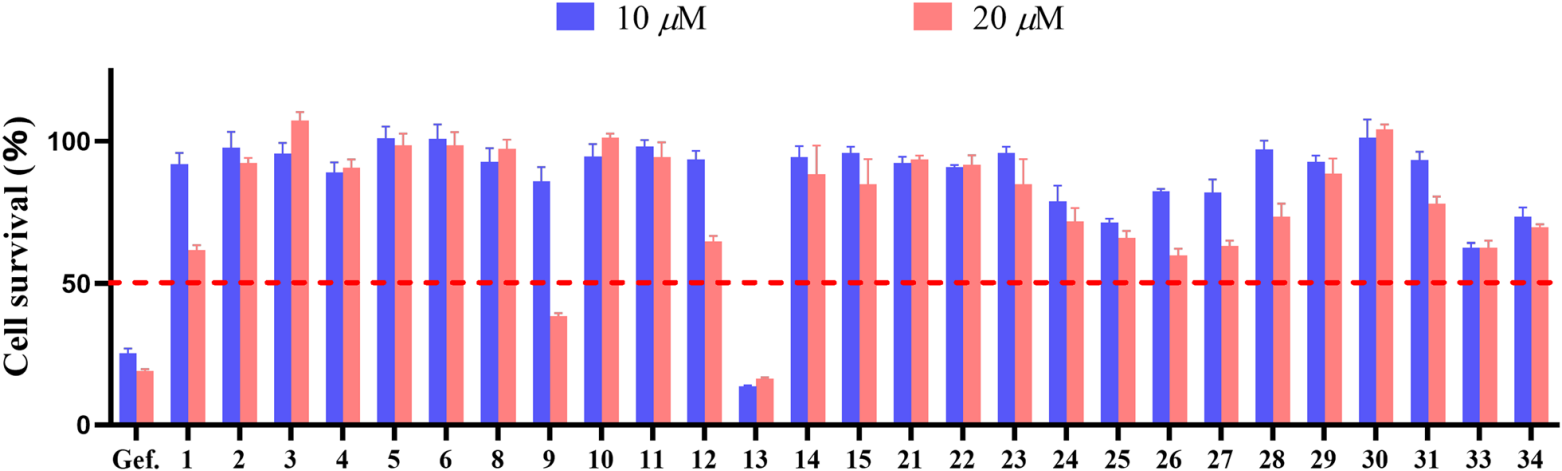
Preliminary screening of the cytotoxic activities of compounds **1**–**15**, **21**–**31**, **33**, and **34** against NCI-H1975 cells. Cells were treated with compounds at the initial screening concentration of 10 and 20 μM for 72 h, then cell viability was determined by Cell Counting Kit-8

The selected compounds **1**–**15** and **21**–**34** isolated from the fungus *P. canescens* L1 were evaluated their cytotoxic activities towards NCI-H1975. At a concentration of 20 μM, two compounds (**9** and **13**) had good cytotoxicity, and **13** had significant activity even at a sub-concentration of 20 μM (Fig. 6). Subsequently, these two active compounds were subjected to evaluate their 50% inhibiting concentration (IC_50_), and compounds **13** and **9** had the IC_50_ values 4.24 ± 0.13 μM and 18.02 ± 0.48 μM, respectively. Gefitinib was a positive control drug with an IC_50_ value of 12.99 ± 0.13 μM.

## Experimental section

### General experimental procedures

Optical rotations were measured on an Anton Paar MCP200 modular circular polarimeter. UV and ECD spectra were recorded on an Applied Photophysics Chirascan spectrometer. IR spectra were obtained from a PerkinElmer Spectrum Two FTIR spectrometer with a UATR accessory. 1D and 2D NMR spectra were collected from Bruker Ascend TM 500 and Bruker Avance III 400 spectrometers at 25 °C with TMS as the internal standard. HR-ESI-MS data were acquired via a Waters Micromass Q-TOF spectrometer and a X500R QTOF spectrometer from SCIEX. Semipreparative HPLC was carried out on a Shimadzu LC-20 AT equipped with an SPD-M20A PDA detector. A NanoChrom ChromCoreTM 5–120 C_18_ column (250 × 10 mm, 5 µm), a Phenomenex Lux cellulose-2 chiral-phase column (250 × 10 mm, 5 μm), and a YMC-pack ODS-A column (250 × 10 mm, S-5 μm, 12 nm) were utilized for HPLC purification. Solvents MeCN for HPLC were purchased from BCR International Trading Co. Ltd. Reversed-phase C_18_ (Rp-C_18_) silica gel from YMC Co. Ltd. (12 nm, S-50 μm), MCI gel from Mitsubishi Chemical Industries Ltd. (CHP20P, 75–150 μm), Sephadex LH-20 gel from Amersham Biosciences, and silica gel from Qingdao Haiyang Chemical Co, Ltd. (100–200, 300–400 mesh) were utilized for general column chromatographic separation, and fractions were monitored via silica gel TLC (GF254 plates, 0.25 mm thickness) visualized with 15% sulfuric acid in EtOH.

### Fungal material

The fungal strain L1 was isolated from the fresh roots sample of *Croton cnidophyllus* and identified as *Penicillium canescens* on the base of ITS region (GenBank OQ438055). The fungal strain (No. L1) is deposited in School of Pharmaceutical Sciences, Sun Yat-sen University.

### Fermentation and extraction

The strain *P. canescens* L1 was cultured on potato dextrose agar (PDA) plates (PDB 24.0 g and agar 18.0 g in 1.0 L H_2_O) at 28 °C for 7 days. The seed medium (PDB media 24.0 g in 1.0 L H_2_O) was inoculated with strain *P. canescens* L1 and incubated at 28.0 °C for 3 days on a rotating shaker (180 rpm). For chemical investigations, a large-scale fermentation of *P. canescens* L1 was incubated for 32 days at 28 °C in 1.5 L × 160 Erlenmeyar flasks (each flask contained 100 g rice and 100 mL H_2_O). After incubation, every flask was ultrasonically extracted with 4 × 0.4 L 95% EtOH for 30 min. After removing the solvents under vacuum, 250 g of crude extract was obtained, which was then suspended in water (3 L) and successively partitioned with petroleum ether (PE, 2 × 3 L) and EtOAc (3 × 3 L).

### Isolation and purification

The obtained EtOAc fraction (150 g) was firstly separated over a silica gel column eluted with a gradient of PE/EtOAc (50:1 → 1:1) followed by the solvents of DCM/MeOH (70:1 → 0:1) to afford eight fractions (Frs. 1–8).

Fr. 4 (4 g) was separated by Rp-C_18_ silica gel CC (MeOH/H_2_O, 20% → 100%) to give five fractions (Frs. 4A–4E). Fr. 4A (1.3 g) was subjected to Sephadex LH-20 CC (MeOH) to yield Fr. 4A1, Fr. 4A2 (150 mg), and compound **9** (87 mg). Compound **3** (22 mg, *t*_R_ 20 min) was obtained from Fr. 4A2 by semipreparative HPLC equipped with a YMC-pack ODS-A column (MeCN/H_2_O, 27:73, 3 mL/min). Compound **29** (23 mg) was precipitated from Fr. 4E (160 mg).

Fr. 5 (18.4 g) was subjected to Rp-C_18_ silica gel CC (MeOH/H_2_O, 20% → 65%) to give five fractions (Frs. 5A–5E). Fr. 5A was divided into three fractions (Frs. 5A1–5A3) by Sephadex LH-20 CC (MeOH). Fr. 5A2 (4.6 g) was further divided into seven fractions (Frs. 5A2A–5A2G) by silica gel CC (PE/DCM, 1:1 → 0:1 and then DCM/MeOH, 500:1 → 70:1). Fr. 5A2D (661 mg) was separated by Sephadex LH-20 CC (MeOH) to give three fractions (Frs. 5A2D1–5A2D3). Compound **20** (8 mg, *t*_R_ 10 min) was obtained from Fr. 5A2D1 (17 mg) by semipreparative HPLC equipped with a chiral column (MeCN/H_2_O, 45:55, 3 mL/min), **19** (58 mg, *t*_R_ 8 min) was obtained from Fr. 5A2D2 (219 mg) by semipreparative chiral HPLC (MeCN/H_2_O, 40:60, 3 mL/min), and **18** (19 mg, *t*_R_ 8 min) was obtained from Fr. 5A2D3 (270 mg) by semipreparative chiral HPLC (MeCN/H_2_O, 35:65, 3 mL/min). Fr. 5B (824 mg) was separated by silica gel CC (PE/DCM, 1:1 → 0:1 and then DCM/MeOH, 500:1 → 70:1) to give Frs. 5B1–5B4 and compound **17** (10 mg). Compounds **4** (18 mg) and **2** (47 mg) were obtained from Fr. 5B1 (90 mg) by Sephadex LH-20 CC (MeOH). Fr. 5C (934 mg) was further divided into six fractions (Frs. 5C1–5C6) by silica gel CC (PE/DCM, 1:1 → 0:1 and then DCM/MeOH, 500:1 → 70:1). Fr.5C2 (260 mg) was successively purified by Sephadex LH-20 CC (MeOH) and semipreparative HPLC equipped with a NanoChrom ChromCoreTM 5–120 C_18_ column (MeCN/H_2_O, 30:70, 3 mL/min) to yield **6** (44 mg, *t*_R_ 7.5 min). Fr. 5E (3.9 g) was separated by Sephadex LH-20 CC (MeOH) to give three fractions (Frs. 5E1–5E3). Fr. 5E1 (1.93 g) was further divided into three fractions (Frs. 5E1A–5E1C) by silica gel CC (PE/DCM, 1:1 → 0:1 and then DCM/MeOH, 500:1 → 70:1). Compounds **11** (83 mg, *t*_R_ 7.5 min) and **13** (24 mg, *t*_R_ 10.5 min) were obtained from Fr. 5E1A (200 mg) by semipreparative HPLC equipped with a NanoChrom ChromCoreTM 5–120 C_18_ column (MeCN/H_2_O, 60:40, 3 mL/min). Fr. 5E1B (520 mg) was subjected to Sephadex LH-20 CC (MeOH) and followed by semipreparative chiral HPLC (MeCN/H_2_O, 53:47, 3 mL/min) to give **5** (14 mg, *t*_R_ 10 min) and **10** (64 mg, *t*_R_ 12.5 min). Fr. 5E1C (818 mg) was subjected to silica gel CC (PE/EtOAc, 70:1 → 0:1) to yield **24** (20 mg) and **1** (2 mg). Fr. 5E2 (1.2 g) was separated by silica gel CC (PE/EtOAc, 70:1 → 1:1) to give compound **22** (220 mg) and Frs. 5E2A–5E2E. Fr. 5E2E (63 mg) was further purified by semipreparative chiral HPLC (MeCN/H_2_O, 50:50, 3 mL/min) to yield **21** (30 mg, *t*_R_ 6 min) and **23** (2 mg, *t*_R_ 8 min).

Fr. 6 (24 g) was subjected to Rp-C_18_ silica gel CC (MeOH/H_2_O, 20% → 70%) to give six fractions (Frs. 6A–6F). Fr. 6A (5.2 g) was further separated by silica gel CC (DCM/MeOH, 500:1 → 70:1) to yield Frs. 6A1–6A5, compound **8** (84 mg), and Fr. 6A6 (300 mg). Fr. 6A6 was firstly separated by semipreparative chiral HPLC (MeCN/H_2_O, 15:85, 3 mL/min) to give two fractions (Frs. 6A7A–6A7B). Then, Fr. 6A7A (91 mg) was purified by HPLC equipped with a NanoChrom ChromCoreTM 5–120 C_18_ column (MeCN/H_2_O, 15:85, 3 mL/min) to yield **16** (21 mg, *t*_R_ 9 min), and compound **7** (8 mg, *t*_R_ 8 min) was obtained from Fr. 6A7B (40 mg) also by HPLC equipped with a NanoChrom ChromCoreTM 5–120 C_18_ column (MeCN/H_2_O, 20:80, 3 mL/min). Fr. 6E (4.7 g) was further separated by silica gel CC (DCM/MeOH, 500:1 → 70:1) to give Frs. 6E1–6E9, **28** (23 mg), and **34** (14 mg). Fr. 6E2 (200 mg) was purified by semipreparative HPLC equipped with a YMC-pack ODS-A column (MeCN/H_2_O, 45:55, 3 mL/min) to yield **12** (22 mg, *t*_R_ 20 min), **15** (3 mg, *t*_R_ 21 min), and **14** (14 mg, *t*_R_ 23.5 min). Fr. 6E5 (510 mg) was separated by Sephadex LH-20 CC (MeOH) to give **25** (34 mg). Fr. 6E6 (500 mg) was subjected to Sephadex LH-20 CC (MeOH) to yield two fractions (Frs. 6E6A–6E6B). Fr. 6E6B (250 mg) was further purified over semipreparative HPLC equipped with a YMC-pack ODS-A column (MeCN/H_2_O, 35:65, 3 mL/min) to afford **31** (3 mg, *t*_R_ 17 min) and **33** (1 mg, *t*_R_ 20 min). Fr. 6E8 (220 mg) was divided into compound **30** (20 mg) and Fr. 6E8A (150 mg) by Sephadex LH-20 CC (MeOH), and then compound **32** (16 mg, *t*_R_ 17 min) was obtained from Fr. 6E8A by HPLC equipped with a NanoChrom ChromCoreTM 5–120 C_18_ column (MeCN/H_2_O, 40:60, 3 mL/min). Fr. 6F (800 mg) was subjected to silica gel CC (PE/DCM, 1:1 → 0:1 and then DCM/MeOH, 500:1 → 70:1) to give compound **26** (140 mg) and Fr. 6F1 (150 mg). Compound **27** (8 mg, *t*_R_ 16 min) was obtained from Fr. 6F1 by semipreparative HPLC equipped with a YMC-pack ODS-A column (MeCN/H_2_O, 68:32, 3 mL/min).

### Spectroscopic data of compounds

#### Penicanesol A (**1**)

Colorless needles; m.p. 256–257; $[\alpha ]_{{\text{D}}}^{{{25}}}$  + 19.9 (ca. 0.09, MeCN); UV (MeCN) *λ*_max_ (log *ε*) 230 (3.50) nm; ECD (ca. 2.6 × 10^−4^ M, MeCN) *λ*_max_ (Δ*ε*) 203 (+4.50), 218 (−2.06), 240 (+ 0.26) nm; IR (UATR) *ν*_max_ 3357, 2921, 2852, 1705, 1612, 1572, 1496, 1349, 1130 cm^−1^; ^1^H and ^13^C NMR data see Table 1; HR-ESI-MS *m/z* 411.1055 [M + Na]^+^ (calcd for C_20_H_20_O_8_Na^+^, 411.1050).

#### Penicanesol B (**2**)

White amorphous solid; $[\alpha ]_{{\text{D}}}^{{{25}}}$ + 36.9 (ca. 0.12, MeCN); UV (MeCN) *λ*_max_ (log *ε*) 190 (3.50) nm; ECD (ca. 4.8 × 10^−4^ M, MeCN) *λ*_max_ (Δ*ε*) 193 (+1.84), 216 (−3.70), 231 (+ 1.04), 247 (−0.35) nm; IR (UATR) *ν*_max_ 3349, 1720, 1630, 1492, 1447, 1353, 1113 cm^−1^; ^1^H and ^13^C NMR data see Table 2; HR-ESI-MS *m/z* 233.0422 [M + Na]^+^ (calcd for C_10_H_10_O_5_Na^+^, 233.0420).

#### Penicanesol C (**3**)

White amorphous solid; $[\alpha ]_{{\text{D}}}^{{{25}}}$ + 12.5 (*c* 0.13, MeCN); UV (MeCN) *λ*_max_ (log *ε*) 190 (3.70) nm; ECD (ca. 4.5 × 10^−4^ M, MeCN) *λ*_max_ (Δ*ε*) 193 (+1.96), 216 (−4.63), 231 (+ 1.11), 247 (−0.37) nm; IR (UATR) *ν*_max_ 3353, 1645, 1516, 1489, 1043 cm^−1^; ^1^H and ^13^C NMR data see Table 2; HR-ESI-MS *m/z* 225.0743 [M + H]^+^ (calcd for C_11_H_13_O_5_^+^, 225.0757), 247.0565 [M + Na]^+^ (calcd for C_11_H_12_O_5_Na^+^, 247.0577).

#### Penicanesol D (**4**)

White amorphous solid; $[\alpha ]_{{\text{D}}}^{{{25}}}$  + 44.9 (*c* 0.10, MeCN); UV (MeCN) *λ*_max_ (log *ε*) 210 (4.80) nm; ECD (ca. 3.9 × 10^−4^ M, MeCN) *λ*_max_ (Δ*ε*) 193 (+2.22), 216 (−5.25), 231 (+ 1.25) nm; IR (UATR) *ν*_max_ 3361, 2921, 2851, 1738, 1629, 1497, 1462, 1184 cm^−1^; ^1^H and ^13^C NMR data see Table 2; HR-ESI-MS *m/z* 277.0687 [M + Na]^+^ (calcd for C_12_H_14_O_6_Na^+^, 277.0683).

#### Penicanesol E (**5**)

Colorless needles; $[\alpha ]_{{\text{D}}}^{{{25}}}$ + 58.9 (ca. 0.11, MeCN); UV (MeCN) *λ*_max_ (log *ε*) 254 (4.70) nm; ECD (ca. 3.4 × 10^−4^ M, MeCN) *λ*_max_ (Δ*ε*) 192 (−12.06), 215 (+ 15.63), 233 (−3.04), 280 (+ 2.60), 329 (−4.75) nm; IR (UATR) *ν*_max_ 3479, 1681, 1615, 1596, 1459, 1216, 1155 cm^−1^; ^1^H and ^13^C NMR data see Table 3; HR-ESI-MS *m/z* 315.1206 [M + Na]^+^ (calcd for C_16_H_20_O_5_Na^+^, 312.1203).

#### Penicanesol F (**6**)

White amorphous solid; UV (MeCN) *λ*_max_ (log *ε*) 254 (4.81) nm; ^1^H and ^13^C NMR data see Table 3; HR-ESI-MS *m/z* 277.0678 [M + Na]^+^ (calcd for C_12_H_14_O_6_Na^+^, 277.0683).

#### Penicanesol G (**7**)

White amorphous solid; $[\alpha ]_{{\text{D}}}^{{{25}}}$  + 34.8 (ca. 0.14, MeCN); UV (MeCN) *λ*_max_ (log *ε*) 216 (3.49), 238 (2.50), 266 (2.98) nm; ECD (ca. 5.1 × 10^−4^ M, MeCN) *λ*_max_ (Δ*ε*) 193 (+1.71), 216 (−4.05), 231 (+ 0.97), 247 (−0.32) nm; IR (UATR) *ν*_max_ 3275, 1702, 1621, 1522, 1497, 1317, 1043 cm^−1^; ^1^H and ^13^C NMR data see Table S2.1 in Supporting Information; HR-ESI-MS *m/z* 197.0441 [M + H]^+^ (calcd for C_12_H_15_O_6_^+^, 197.0444).

### Crystallographic data

Compounds **1**, **5**, **8**, and **13** were recrystallized MeOH/DCM (5:1) to afford colorless needles at room temperature. Crystallographic data for the structures determined in this study have been deposited at the Cambridge Crystallographic Data Centre (deposition number: 2402573 for **1**, 2402743 for **5**, 2407293 for **8**, and 2402745 for **13**) and can be obtained free of charge from the CCDC Web site (https://www.ccdc.cam.ac.uk/).

Penicanesol A (**1**): Crystal Data for C_20_H_20_O_8_ (*M* = 388.36 g/mol): orthorhombic, space group P2_1_2_1_2_1_ (no. 19), *a* = 8.79670(9) Å, *b* = 12.20777(15) Å, *c* = 16.4867(2) Å, *V* = 1770.47(4) Å3, *Z* = 4, *T* = 100.00(10) K, μ(Cu Kα) = 0.959 mm^−1^, *D*_calc_ = 1.457 g/cm^3^, 18,304 reflections measured (9.014° ≤ 2θ ≤ 158.02°), 3748 unique (*R*_int_ = 0.0477, *R*_sigma_ = 0.0310) which were used in all calculations. The final *R*_1_ was 0.0372 (*I* > 2σ(*I*)) and w*R*_2_ was 0.1020 (all data). Flack parameter = 0.02(8).

Penicanesol E (**5**): Crystal Data for C_16_H_20_O_5_ (*M* = 292.32 g/mol): monoclinic, space group P2_1_ (no. 4), *a* = 6.08950(10) Å, *b* = 10.3226(2) Å, *c* = 11.9172(2) Å, *β* = 104.211(2)°, *V* = 726.18(2) Å^3^, *Z* = 2, *T* = 99.99(10) K, μ(Cu Kα) = 0.818 mm^−1^, *D*_calc_ = 1.337 g/cm^3^, 7450 reflections measured (7.652° ≤ 2Θ ≤ 156.932°), 2703 unique (*R*_int_ = 0.0154, *R*_sigma_ = 0.0132) which were used in all calculations. The final *R*_1_ was 0.0270 (*I* > 2σ(*I*)) and *wR*_2_ was 0.0716 (all data). Flack parameter = 0.09 (4).

Penicanesin E (**8**): Crystal Data for C_10_H_10_O_6_ (*M* = 226.18 g/mol): monoclinic, space group P2_1_/c (no. 14), *a* = 8.30853(9) Å, *b* = 14.86270(18) Å, *c* = 7.72506(8) Å, *β* = 97.8579(10)°, *V* = 944.989(18) Å^3^, *Z* = 4, *T* = 100.00(10) K, μ(Cu Kα) = 1.155 mm^−1^, *D*_calc_ = 1.590 g/cm^3^, 18,746 reflections measured (10.75° ≤ 2Θ ≤ 157.508°), 2007 unique (*R*_int_ = 0.0443, R_sigma_ = 0.0214) which were used in all calculations. The final *R*_1_ was 0.0365 (*I* > 2σ(*I*)) and *wR*_2_ was 0.0983 (all data).

4′-Demethoxyisogriseofulvin (**13**): Crystal Data for C_16_H_15_ClO_5_ (*M* = 322.73 g/mol): orthorhombic, space group P2_1_2_1_2_1_ (no. 19), *a* = 11.3451(2) Å, *b* = 16.9711(3) Å, *c* = 38.8580(6) Å, *V* = 7481.7(2) Å^3^, *Z* = 20, *T* = 100.00(10) K, μ(Cu Kα) = 2.462 mm^−1^, *D*_calc_ = 1.433 g/cm^3^, 73,432 reflections measured (4.548° ≤ 2Θ ≤ 157.614°), 15,741 unique (*R*_int_ = 0.0862, *R*_sigma_ = 0.0645) which were used in all calculations. The final *R*_1_ was 0.0948 (*I* > 2σ(*I*)) and *wR*_2_ was 0.2488 (all data). Flack parameter = 0.034 (7). Flack parameter = 0.09 (4).

### ECD calculations

Please see S1.1 in Supporting Information for details of the quantum chemical ECD calculation of **2** and **4**.

### Cytotoxic activity assay

For cell viability, NCI-H1975 cells were seeded in 96-well plates at 2,000 cells per well (optimum density for growth) in a total volume of 100 μL of media containing 10% serum. Serially diluted compounds in 50 μL of media were added to the cells 24 h later. After 3 days of incubation, Cell Counting Kit-8 reagents (Dojindo, Japan) were added, and luminescence was measured according to the manufacturer’s instructions. All experiments were repeated three times. The data are presented as percentage of viable cells with vehicle-treated cells set as 100. The estimated in vitro IC_50_ values were calculated by using GraphPad Prism 9 software.

## Conclusion

In summary, the chemical study of *P. canescens* L1 resulted in the identification of seven novel polyketides labelled as penicanesols A–G (**1**–**7**), and penicanesol A (**1**) was a rare dimer derived from phthalan derivatives among them. In the activity screen, compounds **1**–**15** and **21**–**34** showed different level of cytotoxic activities towards NCI-H1975. Compounds **9** and **13** had significant activities at a concentration of 20 *μ*M, and **13** (IC_50_ = 4.24 ± 0.13 μM) exhibited cytotoxicity superior to that of the positive drug gefitinib (IC_50_ = 12.99 ± 0.13 μM). These findings not only expand the structural diversity of the polyketides but also deepen our understanding of the chemical and bioactivity diversity of plant-derived fungus *P. canescens* L1*.*

## Supplementary Information

Additional file 1 The NMR of **1**–**34**, ECD and HR-ESI-MS spectra of new compounds, 1D NMR spectroscopic data for **7**–**34**, and ECD calculation for **2** and **4**.

## Data Availability

All data generated and analyzed during this study are included in this published article and its Additional file 1.
