# Supplementary material for: Structurally diverse polyketides and alkaloids produced by a plant-derived fungus Penicillium canescens L1
Source: Nat Prod Bioprospect. 2025 Apr 3;15(1):22. doi: 10.1007/s13659-025-00503-0 (PMC11968604; doi:10.1007/s13659-025-00503-0)

Supplementary Information

Structurally diverse polyketides and alkaloids produced by a plant-derived fungus *Penicillium canescens* L1

Wei-Ye Wu[a,#], Xun Wei[a,#], Qiong Liao[b], Yi-Fan Fu[a], Lei-Ming Wu[a], Lei Li[a], Shu-Qi Wu[a], Qing-Ren Lu[a], Fang-Yu Yuan[a], Dong Huang[a], Zhang-Hua Sun[c], Tao Yuan[d] and Gui-Hua Tang[a,]*

[a]School of Pharmaceutical Sciences, Sun Yat-sen University, Guangzhou 510006, China

[b]Laboratory Animal Center, Sun Yat-sen University, Guangzhou 510006, China

[c]Guangdong Provincial Key Laboratory of Utilization and Conservation of Food and Medicinal Resources in Northern Region, Shaoguan University, Shaoguan 512005, China

[d]The Laboratory of Effective Substances of Jiangxi Genuine Medicinal Materials, College of Life Sciences, Jiangxi Normal University, Nanchang, 330022, China

*Correspondence: tanggh5@mail.sysu.edu.cn (G-H Tang)

Contents

S1. Experimental Section ······························ p5

S1.1. ECD calculation of **2** and **4**

S2. Spectroscopic data of compounds listed in Figures or shown in Tables ·············· p9–23

Figure S1.2. Experimental ECD spectra of **1–5** and **7**

Table S2.1. The ^{1}H NMR (400 MHz) and ^{13}C NMR (100 MHz) data of **7** and **8** in CD_3OD

Table S2.2. The ^{1}H NMR (400 MHz) and ^{13}C NMR (100 MHz) data of **9** in acetone-d_6

Table S2.3. The ^{1}H NMR and ^{13}C NMR data of **10** (in $CDCl_3$) and **15** (in pyridine-d_5)

**Table S2.4.** The ^{1}H NMR and ^{13}C NMR data of **11** and **12**
**Table S2.5.** The ^{1}H NMR and ^{13}C NMR data of **13** and **14** in $CDCl_3$
**Table S2.6.** The ^{1}H NMR (400 MHz) and ^{13}C NMR (100 MHz) data of **16** and **17** in CD_3OD
**Table S2.7.** The ^{1}H NMR (400 MHz) and ^{13}C NMR (100 MHz) data of **18** (in CD_3OD) and **19** (in $CDCl_3$)
**Table S2.8.** The ^{1}H NMR (400 MHz) and ^{13}C NMR (100 MHz) data of **20** in $CDCl_3$
**Table S2.9.** The ^{1}H NMR (400 MHz) and ^{13}C NMR (100 MHz) data of **21** in CD_3OD
**Table S2.10.** The ^{1}H NMR (400 MHz) and ^{13}C NMR (100 MHz) data of **22** and **23** in CD_3OD
**Table S2.11.** The ^{1}H NMR (400 MHz) and ^{13}C NMR (100 MHz) data of **24** (in $CDCl_3$) and **25** (in acetone-d_6)
**Table S2.12.** The ^{1}H NMR (400 MHz) and ^{13}C NMR (100 MHz) data of **26** and **27** in $CDCl_3$
**Table S2.13.** The ^{1}H NMR (400 MHz) and ^{13}C NMR (100 MHz) data of **28** in $CDCl_3$
**Table S2.14.** The ^{1}H NMR and ^{13}C NMR data of **29** and **30** (in $CDCl_3$)
**Table S2.15.** The ^{1}H NMR (400 MHz) and ^{13}C NMR (100 MHz) data of **31** (in pyridine-d_5) and **32** (in $CDCl_3$ + CD_3OD)
**Table S2.16.** The ^{1}H NMR and ^{13}C NMR data of **33** (in $CDCl_3$) and **34** (in CD_3OD)

S3. 1D and 2D NMR spectra of compounds **1–34**·····································p24–72

**S3.1.** ^{1}H NMR spectrum of **1** in pyridine-d_5	**S3.2.** ^{13}C NMR and DEPT spectra of **1** in pyridine-d_5
**S3.3.** HSQC spectrum of **1** in pyridine-d_5	**S3.4.** HMBC spectrum of **1** in pyridine-d_5
**S3.5.** ^{1}H NMR spectrum of **2** in CD_3OD	**S3.6.** ^{13}C NMR and DEPT spectra of **2** in CD_3OD
**S3.7.** HSQC spectrum of **2** in CD_3OD	**S3.8.** HMBC spectrum of **2** in CD_3OD
**S3.9.** ^{1}H NMR spectrum of **3** in $CDCl_3$	**S3.10.** ^{1}H NMR and DEPT spectra of **3** in $CDCl_3$
**S3.11.** HMBC spectrum of **3** in $CDCl_3$	
**S3.12.** ^{1}H NMR spectrum of **4** in in CD_3OD	**S3.13.** ^{13}C NMR and DEPT spectra of **4** in CD_3OD
**S3.14.** HSQC spectrum of **4** in CD_3OD	**S3.15.** ^{1}H−^{1}H COSY spectrum of **4** in CD_3OD

**S3.16.** HMBC spectrum of **4** in CD_3OD	
**S3.17.** ^1H NMR spectrum of **5** in $CDCL_3$	**S3.18.** ^{13}C NMR and DEPT spectra of **5** in $CDCL_3$
**S3.19.** HSQC spectrum of **5** in $CDCL_3$	**S3.20.** $^1H-^1H$ COSY spectrum of **5** in $CDCL_3$
**S3.21.** HMBC spectrum of **5** in $CDCL_3$	**S3.22.** NOESY spectrum of **5** in $CDCL_3$
**S3.23.** ^1H NMR spectrum of **6** in $CDCl_3$	**S3.24.** ^{13}C NMR and DEPT spectra of **6** in $CDCl_3$
**S3.25.** HSQC spectrum of **6** in $CDCl_3$	**S3.26.** $^1H-^1H$ COSY spectrum of **6** in $CDCl_3$
**S3.27.** HMBC spectrum of **6** in $CDCl_3$	
**S3.28.** ^1H NMR spectrum of **7** in CD_3OD	**S3.29.** ^{13}C NMR and DEPT spectra of **7** in CD_3OD
**S3.30.** ^1H NMR spectrum of **8** in CD_3OD	**S3.31.** ^{13}C NMR and DEPT spectra of **8** in CD_3OD
**S3.32.** ^1H NMR spectrum of **9** in CD_3COCD_3	**S3.33.** ^{13}C NMR and DEPT spectra of **9** in CD_3COCD_3
**S3.34.** ^1H NMR spectrum of **10** in $CDCl_3$	**S3.35.** ^{13}C NMR and DEPT spectra of **10** in $CDCl_3$
**S3.36.** HSQC spectrum of **10** in $CDCl_3$	**S3.37.** $^1H-^1H$ COSY spectrum of **10** in $CDCl_3$
**S3.38.** HMBC spectrum of **10** in $CDCl_3$	
**S3.39.** ^1H NMR spectrum of **11** in $CDCl_3$	**S3.40.** ^{13}C NMR and DEPT spectra of **11** in $CDCl_3$
**S3.41.** ^1H NMR spectrum of **12** in $CDCl_3$	**S3.42.** ^{13}C NMR spectrum of **12** in $CDCl_3$
**S3.43.** ^1H NMR spectrum of **12** in CD_3OD	**S3.44.** ^{13}C NMR and DEPT spectra of **12** in CD_3OD
**S3.45.** ^1H NMR spectrum of **13** in $CDCl_3$	**S3.46.** ^{13}C NMR and DEPT spectra of **13** in $CDCl_3$
**S3.47.** HSQC spectrum of **13** in $CDCl_3$	**S3.48.** $^1H-^1H$ COSY spectrum of **13** in $CDCl_3$
**S3.49.** HMBC spectrum of **13** in $CDCl_3$	
**S3.50.** ^1H NMR spectrum of **14** in $CDCl_3$	**S3.51.** ^{13}C NMR and DEPT spectra of **14** in $CDCl_3$
**S3.52.** ^1H NMR spectrum of **15** in pyridine-d_5	**S3.53.** ^{13}C NMR and DEPT spectra of **15** in pyridine-d_5
**S3.54.** ^1H NMR spectrum of **16** in CD_3OD	**S3.55.** ^{13}C NMR and DEPT spectra of **16** in CD_3OD
**S3.56.** ^1H NMR spectrum of **17** in CD_3OD	**S3.57.** ^{13}C NMR and DEPT spectra of **17** in CD_3OD
**S3.58.** ^1H NMR spectrum of **18** in CD_3OD	**S3.59.** ^{13}C NMR and DEPT spectra of **18** in CD_3OD

**S3.60.** ^{1}H NMR spectrum of **19** in $CDCl_3$	**S3.61.** ^{13}C NMR spectrum of **19** in $CDCl_3$
**S3.62.** ^{1}H NMR spectrum of **20** in $CDCl_3$	**S3.63.** ^{13}C NMR and DEPT spectra of **20** in $CDCl_3$
**S3.64.** HSQC spectrum of **20** in $CDCl_3$	**S3.65.** HMBC spectrum of **20** in $CDCl_3$
**S3.66.** ^{1}H NMR spectrum of **21** in CD_3OD	**S3.67.** ^{13}C NMR and DEPT spectra of **21** in CD_3OD
**S3.68.** ^{1}H NMR spectrum of **22** in CD_3OD	**S3.69.** ^{13}C NMR and DEPT spectra of **22** in CD_3OD
**S3.70.** ^{1}H NMR spectrum of **23** in CD_3OD	**S3.71.** ^{13}C NMR and DEPT spectra of **23** in CD_3OD
**S3.72.** ^{1}H NMR spectrum of **24** in $CDCl_3$	**S3.73.** ^{13}C NMR and DEPT spectra of **24** in $CDCl_3$
**S3.74.** HSQC spectrum of **24** in $CDCl_3$	**S3.75.** ^{1}H−^{1}H COSY spectrum of **24** in $CDCl_3$
**S3.76.** HMBC spectrum of **24** in $CDCl_3$	
**S3.77.** ^{1}H NMR spectrum of **25** in CD_3COCD_3	**S3.78.** ^{13}C NMR spectrum of **25** in CD_3COCD_3
**S3.79.** ^{1}H NMR spectrum of **26** in $CDCl_3$	**S3.80.** ^{13}C NMR and DEPT spectra of **26** in $CDCl_3$
**S3.81.** ^{1}H NMR spectrum of **27** in $CDCl_3$	**S3.82.** ^{13}C NMR and DEPT spectra of **27** in $CDCl_3$
**S3.83.** ^{1}H NMR spectrum of **28** in $CDCl_3$	**S3.84.** ^{13}C NMR spectrum of **28** in $CDCl_3$
**S3.85.** ^{1}H NMR spectrum of **29** in $CDCl_3$	**S3.86.** ^{13}C NMR spectrum of **29** in $CDCl_3$
**S3.87.** ^{1}H NMR spectrum of **30** in $CDCl_3$	**S3.88.** ^{13}C NMR and DEPT spectra of **30** in $CDCl_3$
**S3.89.** HMBC spectrum of **30** in $CDCl_3$	
**S3.90.** ^{1}H NMR spectrum of **31** in pyridine-d_5	**S3.91.** ^{13}C NMR and DEPT spectra of **31** in pyridine-d_5
**S3.92.** ^{1}H NMR spectrum of **32** in CD_3OD + $CDCl_3$	**S3.93.** ^{13}C NMR and DEPT spectra of **32** in CD_3OD + $CDCl_3$
**S3.94.** ^{1}H NMR spectrum of **33** in $CDCl_3$	**S3.95.** ^{13}C NMR and DEPT spectra of **33** in $CDCl_3$
**S3.96.** ^{1}H NMR spectrum of **34** in CD_3OD	**S3.97.** ^{13}C NMR and DEPT spectra of **34** in CD_3OD

S4. HR-ESI-MS Spectra of New Compounds 1–7··p73–76

**S4.1.** HR-ESI-MS spectrum of **1**	**S4.2.** HR-ESI-MS spectrum of **2**
**S4.3.** HR-ESI-MS spectrum of **3**	**S4.4.** HR-ESI-MS spectrum of **4**

**S4.5.** HR-ESI-MS spectrum of **5**	**S4.6.** HR-ESI-MS spectrum of **6**
**S4.7.** HR-ESI-MS spectrum of **7**	

S1. Experimental Section

S1.1. ECD calculations of **2** and **4**

The absolute configurations of **2** and **4** was determined by quantum chemical calculations of their theoretical ECD spectra. One of the two enantiomers for each compound, (3*R*)-**2** and **(**3*R***)-4** were arbitrary chosen for theoretical studies. Conformational analyses were first carried out via Monte Carlo searching using molecular mechanism with MMFF force field in the *Spartan 18* program.[1] The results showed one lowest energy conformer for **2**, and eleven for **4** within an energy window of 2.5 Kcal/mol. These conformers were reoptimized using DFT at the B3LYP/6-31G(d) level in gas phase using the Gaussian 09 program.[2] One conformer of **2** (Figure S1.1), and three conformers of **4** (Figure S1.2), whose relative Gibbs free energies in the range of 0–2.0 Kcal/mol (Table S1.1) were refined and considered for next step. All the reoptimized conformers mentioned above for **2** and **4** were applied for theoretical ECD calculation. The energies, oscillator strengths, and rotational strengths of the 60 electronic excitations were calculated using the TD-DFT methodology at the rb3lyp/6-311+g(d,p) level in gas phase (Table S1.2). To get the final ECD spectrum of each compound, the simulated spectra of the lowest energy conformers were averaged according to the Boltzmann distribution theory and their relative Gibbs free energy (ΔG) ($\sigma = 0.30$ eV for **2** and **4**). Boltzmann statistics were used for simulations of the ECD spectra of the molecules using SpecDis 1.64. [3] The theoretical ECD curves of (3*S*)-**2** and (3*S*)-**4** were obtained by directly reverse those of (3*R*)-**2** and **(**3*R***)-4**, respectively.

References

(1) *Spartan 18*; Wavefunction Inc.:Irvine, CA.

(2) *Gaussian 09*, Revision A.1, Frisch, M. J.; Trucks, G. W.; Schlegel, H. B.; Scuseria, G. E.; Robb, M. A.; Cheeseman, J. R.; Scalmani, G.; Barone, V.; Mennucci, B.; Petersson, G. A.; Nakatsuji, H.; Caricato, M.; Li, X.; Hratchian, H. P.; Izmaylov, A. F.; Bloino, J.; Zheng, G.; Sonnenberg, J. L.; Hada, M.; Ehara, M.; Toyota, K.; Fukuda, R.; Hasegawa, J.; Ishida, M.; Nakajima, T.; Honda, Y.; Kitao, O.; Nakai, H.; Vreven, T.; Montgomery, Jr., J. A.; Peralta, J. E.; Ogliaro, F.; Bearpark, M.; Heyd, J. J.; Brothers, E.; Kudin, K. N.; Staroverov, V. N.; Kobayashi, R.; Normand, J.; Raghavachari, K.; Rendell, A.; Burant, J. C.; Iyengar, S. S.; Tomasi, J.; Cossi, M.; Rega, N.; Millam, J. M.; Klene, M.; Knox, J. E.; Cross, J. B.; Bakken, V.; Adamo, C.; Jaramillo, J.; Gomperts, R.; Stratmann, R. E.; Yazyev, O.; Austin, A. J.; Cammi, R.; Pomelli, C.; Ochterski, J. W.; Martin, R. L.; Morokuma, K.; Zakrzewski, V. G.; Voth, G. A.; Salvador, P.; Dannenberg, J. J.; Dapprich, S.; Daniels, A. D.; Farkas, Ö.; Foresman, J. B.; Ortiz, J. V.; Cioslowski, J.; Fox, D. J. Gaussian, Inc., Wallingford CT, 2009.

(3) Bruhn, T.; Schaumlöffel, A.; Hemberger, Y.; Bringmann, G. Chirality 2013, 25, 243−249.

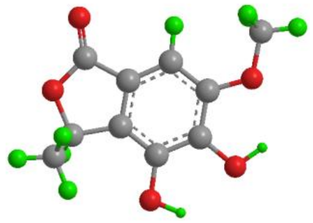

2C1

Figure S1.1. B3LYP/6-31G(d) optimized lowest energy conformer for **2**.

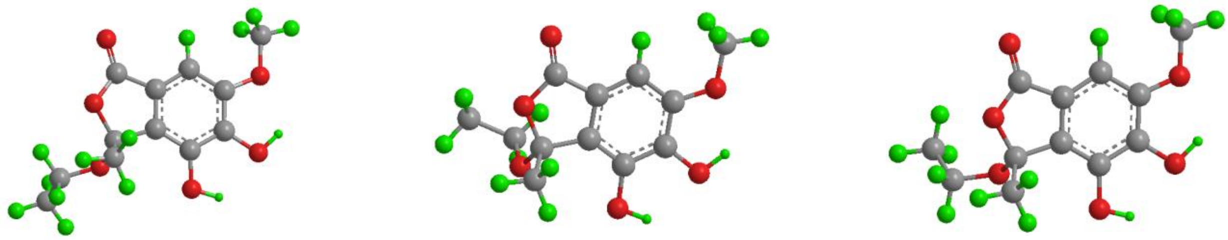

4C1 **4C2** **4C3**

Figure S1.2. B3LYP/6-31G(d) optimized lowest energy conformers for **4**.

Table S1.1. Energy (298.15 K) analysis for **2** and **4**

Conf.	G (Hartree)	ΔG (Kcal/mol)	Boltzmann Distribution
**2C1**	−763.4302574	0	1
**4C1**	−917.3143224	0	0.8513
**4C2**	−917.3115542	1.247614	0.1034
**4C3**	−917.3123342	1.737072	0.0452

ECD data

ECD spectrum of each conformation is simulated according to the overlapping Gaussian functions expressed as:

$$\Delta\varepsilon(E)=\frac{1}{2.296\times10^{-39}\sqrt{\pi}\sigma}\sum_{i}^{A}\Delta E_i \mathrm{R}_i e^{[-(E-\Delta Ei)^2/\sigma^2]}$$

Where σ is half the bandwidth at 1/e peak height and expressed in energy units. The parameters ΔE_i and R_i are the excitation energies and rotational strengths for the transition i, respectively.

The above function is converted to Δε, λ (wavelength) correlations as:

$$\Delta\varepsilon(\lambda)=\frac{1}{2.296\times10^{-39}\sqrt{\pi}\sigma}\sum_{i}^{A}\Delta E_i \mathrm{R}_i e^{[-(1240/\lambda-\Delta Ei)^2/\sigma^2]}$$

and then simulation was accomplished by using the Excel 2003 and the Origin 9.1 software.

To get the final spectra, all the simulated spectra of conformations of each compound were averaged according to their energy and the Boltzmann distribution theory expressed as:

$$\frac{N_i^*}{N} = \frac{g_i e^{-\varepsilon_i / k_B T}}{\sum g_i e^{-\varepsilon_i / k_B T}}$$

Table S1.2. Calculated ECD data of **2** (**2C1**) and **4** (**4C1–4C3**) in gas phase

State	**2C1**		**4C1**		**4C2**		**4C3**	
	Excitation energies(eV)	Rotatory Strengths*	Excitation energies(eV)	Rotatory Strengths*	Excitation energies(eV)	Rotatory Strengths*	Excitation energies(eV)	Rotatory Strengths*
1	4.6056	0.3461	4.5285	-2.1202	4.5059	-3.629	4.5167	-2.7396
2	4.7949	11.3961	4.6897	-23.4225	4.6643	-38.8668	4.6770	-13.3046
3	4.8137	-18.4548	4.7506	18.5819	4.7447	41.7498	4.7394	12.8089
4	5.0575	6.2076	5.0740	0.8727	5.0980	-0.2459	5.0828	1.5149
5	5.3673	0.8321	5.4333	2.4814	5.1688	-6.2347	5.2925	-2.3361
6	5.7795	-21.2551	5.4522	-0.3074	5.4608	-0.9457	5.4618	-0.6194
7	5.8473	-1.3084	5.7264	8.152	5.7140	1.6	5.7174	12.5854
8	6.0198	-25.9547	5.8340	-6.8678	5.8583	-4.5791	5.8375	-6.8248
9	6.0429	12.6649	6.0524	-28.2734	6.0531	-97.8893	6.0540	-34.0025
10	6.1217	32.1549	6.0917	-1.4214	6.0771	47.9788	6.1141	-6.2731
11	6.2465 eV	-1.6746	6.1337	0.7836	6.1754	0.0261	6.1354	13.242
12	6.2673	-10.834	6.2449	5.3156	6.2029	9.1523	6.2150	10.6719
13	6.3431	21.7909	6.2931	0.2392	6.3137	-2.1931	6.2296	-2.0387
14	6.4272	-5.3873	6.3877	-3.9796	6.3548	27.9456	6.4116	0.2076
15	6.4780	-0.5923	6.4065	-0.536	6.4151	-2.3333	6.4193	-4.3486
16	6.6030	0.4544	6.5075	0.2176	6.4288	-8.7219	6.4670	-6.3578
17	6.6501	-12.3401	6.5434	2.2303	6.4633	0.5691	6.4875	-3.006
18	6.7366	-3.4694	6.5493	-5.6688	6.5474	0.8669	6.5582	-0.4682
19	6.7811	-4.0328	6.6385	0.053	6.5895	-6.8394	6.6420	-0.9315
20	6.8885	3.6618	6.7096	2.3408	6.6592	-4.9095	6.7210	3.1059
21	6.9295	14.0668	6.7542	0.0951	6.6917	0.1264	6.7328	-0.1966
22	6.9486	0.0372	6.7729	7.8012	6.7859	-1.3597	6.8184	34.9043
23	6.9892	-2.7428	6.8241	17.226	6.7951	12.2269	6.8241	-7.0395
24	7.0507	-10.5719	6.8433	-3.5175	6.9119	1.8044	6.8421	1.6598
25	7.0670	-3.3566	6.8653	-8.6229	6.9425	14.4208	6.8647	-11.0635

26	7.1265	10.3455	6.9181	1.789	6.9516	1.657	6.9217	-3.7704
27	7.1421	0.1472	6.9678	1.6264	7.0207	3.4198	6.9384	14.4709
28	7.1790	-1.2228	7.0278	-1.0417	7.0395	3.6219	7.0242	-13.85
29	7.2063	4.2343	7.0911	-8.3048	7.1022	-12.9854	7.0463	2.0819
30	7.2933	2.2285	7.1030	4.7898	7.1635	19.8018	7.1031	0.2828
31	7.3009	-3.5204	7.1281	2.4654	7.1682	-3.8701	7.1177	4.2111
32	7.4420	1.3941	7.1612	2.7735	7.2235	6.7004	7.1629	0.6879
33	7.4595	-10.137	7.2873	11.5621	7.2928	2.9987	7.2100	-8.479
34	7.4862	-3.2165	7.2876	-3.2518	7.2986	-2.8236	7.2521	3.5999
35	7.5315	10.4528	7.3284	-0.212	7.3072	0.7212	7.3188	-0.5216
36	7.5494	0.4294	7.3362	2.7105	7.3431	0.8391	7.3298	20.5217
37	7.5794	13.4189	7.3535	-1.4868	7.3950	-3.4666	7.3560	-1.3923
38	7.6410	1.8144	7.3626	-2.8933	7.4208	-2.8889	7.3626	-8.7974
39	7.6551 eV	-7.2447	7.3751	1.9365	7.4249	16.4008	7.3751	-2.8182
40	7.6967	-0.5776	7.3946	-5.3383	7.4457	-1.7618	7.3829	-2.0065
41	7.7126	10.0202	7.4406	18.9424	7.4540	-9.5382	7.4625	2.6047
42	7.7554	1.5688	7.4958	17.693	7.4745	14.3321	7.4666	4.1263
43	7.7634	-2.4252	7.5462	1.4057	7.5030	-0.5789	7.4820	4.1058
44	7.7733	0.5407	7.5558	-8.1509	7.5313	1.0622	7.5016	11.2152
45	7.7909	-2.541	7.6170	-5.3063	7.5825	-14.0139	7.5221	-15.349
46	7.8071	-0.775	7.6214	-1.5379	7.6226	11.583	7.6011	6.735
47	7.8593	2.8497	7.6262	6.3569	7.6258	-1.7248	7.6251	-1.0914
48	7.8910	-1.9999	7.6408	1.6292	7.6361	-4.1555	7.6306	-0.5601
49	7.9664	-10.7464	7.6502	-12.2473	7.6448	3.4884	7.6722	-12.1533
50	7.9830	1.3781	7.6548	5.7524	7.6488	-3.1421	7.7357	-0.7972
51	8.0054	3.575	7.6877	-6.316	7.6783	2.3171	7.7486	2.9649
52	8.0136	2.6232	7.7392	0.7723	7.7089	-37.5077	7.7726	1.3953
53	8.0315	-3.0096	7.8060	-4.4232	7.7680	-0.7602	7.7814	4.7361
54	8.0643	-0.9957	7.8249	-2.5081	7.7778	6.1458	7.7864	-21.4318
55	8.0995	-0.1068	7.8339	-6.469	7.7948	8.0739	7.8124	-8.3044
56	8.1532	2.9184	7.8357	2.2279	7.8194	3.4374	7.8340	-3.271
57	8.2150	-1.4588	7.8439	-4.8979	7.8584	-0.9803	7.8465	-1.2995
58	8.2340	7.6138	7.8563	-3.7804	7.8737	1.3413	7.8528	6.798
59	8.2515	-12.1491	7.8796	-11.2606	7.8882	2.7283	7.8611	0.071
60	8.2698	1.7783	7.8861	-2.4836	7.9027	-2.639	7.8802	-1.4046

* R(velocity) 10**-40 erg-esu-cm

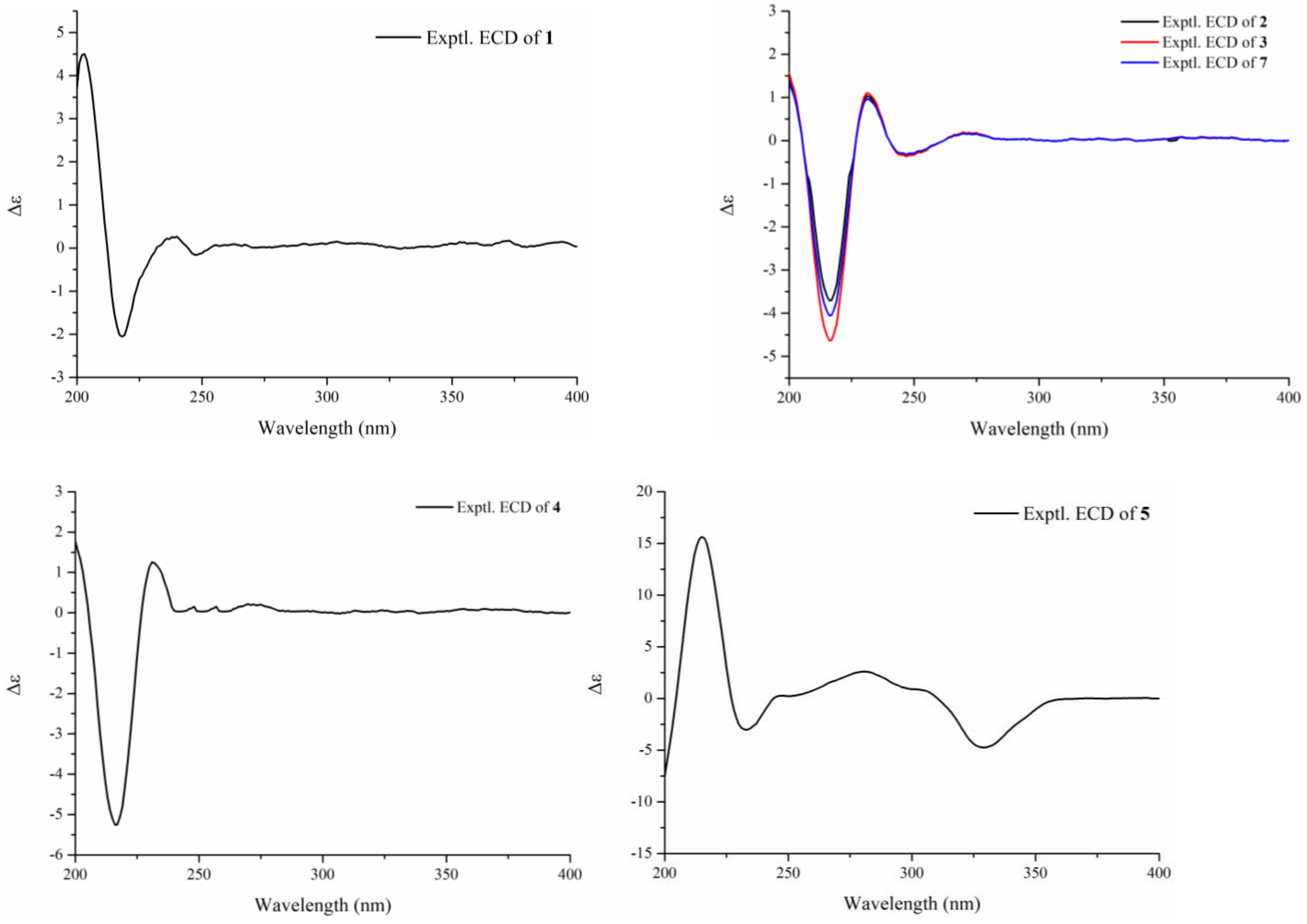

Figure S1.2. Experimental ECD spectra of **1–5** and **7**.

S2. Spectroscopic data of new compounds listed in Tables or shown in Figures

Table S2.1. The ^{1}H NMR (400 MHz) and ^{13}C NMR (100 MHz) data of **7** and **8** in CD_3OD

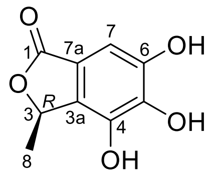

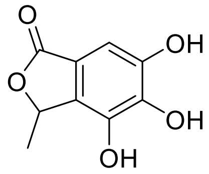

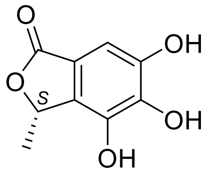

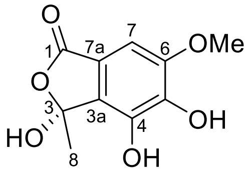

Position	Penicanesol G (**7**) $[\alpha]_{D}^{25}$ = +58.9	4,5,6-Trihydroxy-3-methylphthalide[a] $[\alpha]_{D}^{25}$ = 0	(*S*)-4,5,6-Trihydroxy-3-methylphthalide[b] $[\alpha]_{D}^{25}$ = −25.9	Penicanesin E (**8**)
	δ_H, multi. (*J* in Hz)	δ_C, type	δ_H, multi. (*J* in Hz)	δ_C, type
1		173.9, C		171.6, C
3	5.50, q (6.5)	78.1, CH		106.9, C
3a		132.6, C		131.6, C
4		141.3, C		141.8, C
5		141.0, C		141.8, C
6		148.7, C		151.6, C
7	6.78, s	102.9, CH	6.91, s	99.2, CH
7a		116.8, C		117.9, C
8	1.59, d (6.5)	19.7, CH_3	1.88, s	25.0, CH_3
6-OMe			3.91, s	56.9, CH_3

[a]See the ref.: *Journal of Natural Products* **2004**, 67(12), 2086–2089; [a]See the ref.: *Angewandte Chemie International Edition* **2024**, 63(6), e202316741.

Table S2.2. The ^{1}H NMR (400 MHz) and ^{13}C NMR (100 MHz) data of **9** in acetone-d_6

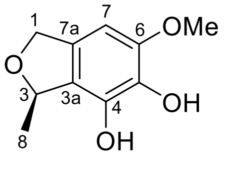

Curvulol (**9**)

Position	δ_H, multi. (*J* in Hz)	δ_C, type	Position	δ_H, multi. (*J* in Hz)	δ_C, type
1	a 4.97, ddd (11.5, 2.6, 0.6); b 4.83, ddd (11.5, 1.6, 0.9)	73.0, CH_2	6		149.2, C
3	5.28, qdd (6.2, 2.6, 1.6)	80.0, CH	7	6.40, s	96.4, CH
3a		123.3, C	7a		130.9, C
4		140.6, C	8	1.44, d (6.2)	21.5, CH_3
5		133.4, C	6-OMe	3.79, s	56.6, CH_3

Table S2.3. The ^{1}H NMR and ^{13}C NMR data of **10** (in $CDCl_3$) and **15** (in pyridine-d_5)

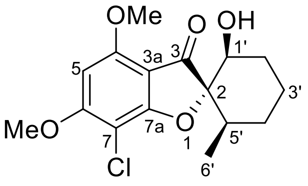

Penigriseofulvin E (**10**)

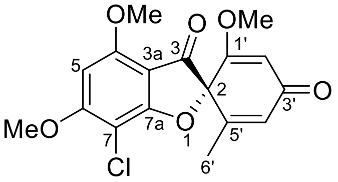

Dehydrogriseofulvin (**15**)

Position	Penigriseofulvin E (**10**)		Dehydrogriseofulvin (**15**)	
	δ_H, multi. (J in Hz)[a]	δ_C, type	δ_H, multi. (J in Hz)[b]	δ_C, type
2		96.9, C		89.7, C
3		196.7, C		189.7, C
3a		108.2, C		105.2, C
4		157.0, C		159.4, C
5	6.06, s	88.9, CH	6.32, s	91.5, CH
6		163.8, C		165.9, C
7		96.8, C		98.0, C
7a		169.2, C		170.5, C
1′	4.01, br s	74.8, CH		168.5, C
2′	a 2.29, m; b 1.88, m	29.1, CH_2	6.01, s	104.8, CH
3′	a 1.86, m; b 1.39, m	23.3, CH_2		186.6, C
4′	a 2.02, m; b 1.54, m	28.5, CH_2	6.43, s	130.3, CH
5′	2.03, m	38.7, CH		147.9, C
6′	0.80, d (6.3)	14.8, CH_3	1.83, s	16.9, CH_3
4-OMe	3.94, s	56.3, CH_3	3.91, s	57.8, CH_3
6-OMe	3.99, s	56.9, CH_3	3.83, s	57.1, CH_3
1′-OMe			3.46, s	56.9, CH_3

[a] Measured at 500 MHz for ^{1}H NMR, 125 MHz for ^{13}C NMR; [b] Measured at 400 MHz for ^{1}H NMR, 100 MHz for ^{13}C NMR.

Table S2.4. The ^{1}H NMR and ^{13}C NMR data of **11** and **12**

Position	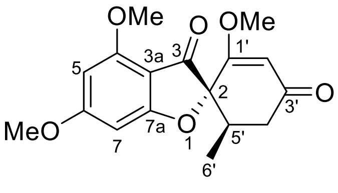 Dechlorogriseofulvin (**11**)		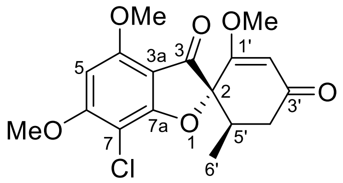 Griseofulvin (**12**)			
	δ_H, multi. (J in Hz)[a]	δ_C, type	δ_H, multi. (J in Hz)[b]	δ_C, type	δ_H, multi. (J in Hz)[c]	δ_C, type
2		90.1, C		90.8, C		91.8, C
3		192.7, C		192.6, C		193.9, C
3a		104.9, C		105.2, C		105.3, C
4		159.2, C		157.9, C		159.6, C
5	6.21, d (1.2)	88.7, CH	6.12, s	89.6 CH	6.42, s	91.5, CH
6		171.5, C		164.7, C		166.8, C
7	6.02, d (1.2)	93.5, CH		97.3, C		97.8, C
7a		176.2, C		169.6, C		170.8, C
1′		170.5, C		170.9 C		173.6 C
2′	5.51, s	104.5, CH	5.52, s	104.9, CH	5.62, s	105.8, CH
3′		197.5, C		197.1, C		199.6, C
4′	a 3.03, dd (16.6, 13.7)<br>b 2.37, dd (16.6, 4.5)	40.2, CH_2	a 3.00, dd (16.7, 13.4)<br>b 2.41, dd (16.7, 4.7)	40.1, CH_2	a 2.87, m, overlapped<br>b 2.42, dd (15.0, 2.9)	40.8, CH_2
5′	2.73, m	36.7, CH	2.82, m,	36.5, CH	2.84, m, overlapped	37.5, CH
6′	0.94, d (6.7)	14.4, CH_3	0.94, d (6.7)	14.3, CH_3	0.91, d (6.4)	14.4, CH_3
4-OMe	3.88, s	56.7, CH_3	4.02, s	57.1, CH_3	4.06, s	57.9, CH_3
6-OMe	3.88, s	56.25, CH_3	3.96, s	56.8, CH_3	3.98, s	57.7, CH_3
1′-OMe	3.60,	56.23, CH_3	3.60, s	56.5, CH_3	3.69, s	57.0, CH_3

[a] Measured at 400 MHz for ^{1}H NMR, 100 MHz for ^{13}C NMR in $CDCl_3$; [b] Measured at 500 MHz for ^{1}H NMR, 125 MHz for ^{13}C NMR in $CDCl_3$; [c] Measured at 400 MHz for ^{1}H NMR, 100 MHz for ^{13}C NMR in CD_3OD.

Table S2.5. The ^{1}H NMR and ^{13}C NMR data of **13** and **14** in $CDCl_3$

Position	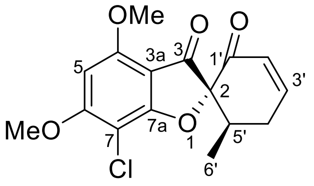Demethoxyisogriseofulvin (**13**)		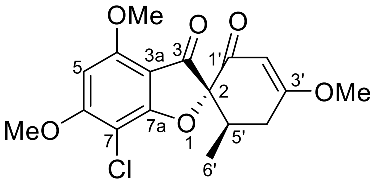Isogriseofulvin (**14**)	
	δ_H, multi. (J in Hz)[a]	δ_C, type	δ_H, multi. (J in Hz)[b]	δ_C, type
2		96.0, C		95.3, C
3		190.9, C		191.9, C
3a		105.1, C		105.3, C
4		157.9, C		157.9, C
5	6.09, s	89.7, CH	6.07, s	89.6, CH
6		164.6, C		164.6, C
7		96.0, C		97.5, C
7a		169.6, C		169.9, C
1′		189.6, C		188.9, C
2′	6.09, dd (10.2, 2.1)	126.7, CH	5.42, d (1.3)	99.8, CH
3′	7.15, ddd (10.2, 5.7, 2.1)	152.5, CH		179.1, C
4′	a 3.04, ddt (19.2, 11.1, 2.1);<br>b 2.51, dt (19.2, 5.7)	31.3, CH_2	a 3.17, ddd (17.6, 12.0, 1.3);<br>b 2.45, dd (17.6, 5.7)	33.1, CH_2
5′	2.84, m	37.1, CH	2.83, m	35.3, CH
6′	1.02, d (6.7)	14.7, CH_3	1.01, d (6.7)	14.6, CH_3
4-OMe	4.00, s	57.1, CH_3	3.99, s	57.1, CH_3
6-OMe	3.92, s	56.4, CH_3	3.90, s	56.44, CH_3
3′-OMe			3.76, s	56.41, CH_3

[a] Measured at 500 MHz for ^{1}H NMR, 125 MHz for ^{13}C NMR; [b] Measured at 400 MHz for ^{1}H NMR, 100 MHz for ^{13}C NMR.

Table S2.6. The ^{1}H NMR (400 MHz) and ^{13}C NMR (100 MHz) data of **16** and **17** in CD_3OD

Position	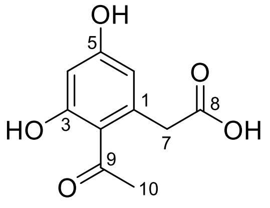 Curvulin acid (**16**)		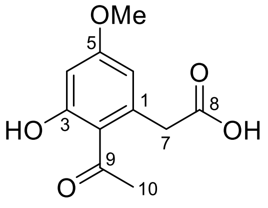 *O*-Methylcurvulinic acid (**17**)	
	δ_H, multi. (J in Hz)	δ_C, type	δ_H, multi. (J in Hz)	δ_C, type
1		138.0, C		137.6, C
2		120.5, C		121.8, C
3		161.1, C		160.4, C
4	6.20, d (2.2)	102.7, CH	6.34, d (2.3)	101.1, CH
5		161.9, C		163.6, C
6	6.27, d (2.2)	112.1, CH	6.36, d (2.3)	110.7, CH
7	3.65, br s	40.8, CH_2	3.69, br s	40.6, CH_2
8		175.3, C		175.2, C
9		206.2, C		206.3, C
10	2.52, s	32.4, CH_3	2.53, s	32.3, CH_3
5-OMe			3.78, s	55.8, CH_3

Table S2.7. The ^{1}H NMR (400 MHz) and ^{13}C NMR (100 MHz) data of **18** (in CD_3OD) and **19** (in $CDCl_3$)

Position	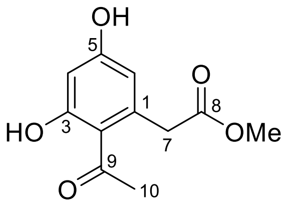 Methyl curvulinate (**18**)		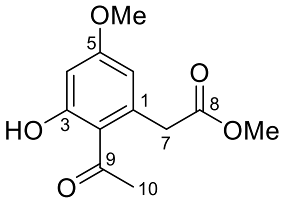 Methyl 2-(2-acetyl-3-hydroxy-5-methoxyphenyl)acetate (**19**)	
	δ_H, multi. (J in Hz)	δ_C, type	δ_H, multi. (J in Hz)	δ_C, type
1		137.7, C		136.8, C
2		120.5, C		115.7, C
3		161.1, C		164.1, C
4	6.19, d (2.3)	102.8, CH	6.29, s	100.5, CH
5		161.9, C		165.9, C
6	6.28, d (2.3)	112.2, CH	6.33, s	112.7, CH
7	3.65, s	40.7, CH_2	3.86, s	41.7, CH_2
8		174.1, C		171.5, C
9		205.8, C		203.5, C
10	2.51, s	32.5, CH_3	2.56, s	32.0, CH_3
5-OMe			3.77, s	55.5, CH_3
8-OMe	3.65, s	52.3, CH_3	3.70, s	52.4, CH_3
3-OH			12.71, s	

Table S2.8. The ^{1}H NMR (400 MHz) and ^{13}C NMR (100 MHz) data of **20** in $CDCl_3$

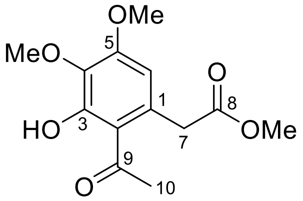

Methyl 2-acetyl-3-hydroxy-4,5-dimethoxybenzeneacetate (**20**)

Position	δ_H, multi. (J in Hz)	δ_C, type	Position	δ_H, multi. (J in Hz)	δ_C, type
1		130.3, C	8		172.1, C
2		119.9, C	9		202.5, C
3		151.2, C	10	2.60, s	32.3, CH_3
4		134.8, C	3-OH	7.72, s	
5		154.0, C	4-OMe	3.90, s	61.0, CH_3
6	6.34, s	107.7, CH	5-OMe	3.89, s	56.0, CH_3
7	3.77, s	40.2, CH_2	8-OMe	3.70, s	52.2, CH_3

Table S2.9. The ^{1}H NMR (400 MHz) and ^{13}C NMR (100 MHz) data of **21** in CD_3OD

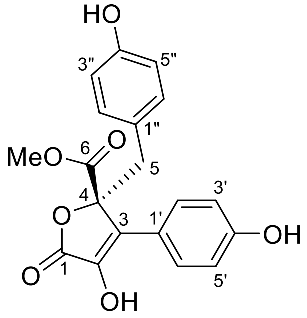

Butyrolactone II (**21**)

Position	δ_H, multi. (*J* in Hz)	δ_C, type	Position	δ_H, multi. (*J* in Hz)	δ_C, type
1		170.3, C	1′		123.1, C
2		139.8, C	2′/6′	7.60, d (8.3)	130.4, CH
3		129.2, C	3′/5′	6.88, d (8.3)	116.6, CH
4		86.8, C	4′		159.3, C
5	3.47, s	39.5, CH_2	1″		125.2, C
6		171.5, C	2″/6″	6.65, d (8.1)	132.5, CH
6-OMe	3.79, s	53.9, CH_3	3″/5″	6.53, d (8.1)	115.6, CH
			4″		157.5, C

Table S2.10. The ^{1}H NMR (400 MHz) and ^{13}C NMR (100 MHz) data of **22** and **23** in CD_3OD

Position	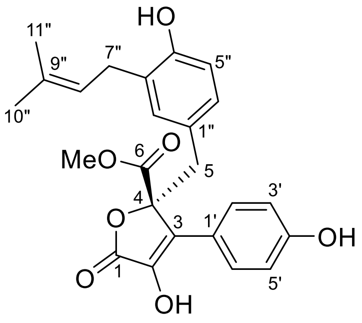<br>Butyrolactone I (**22**)		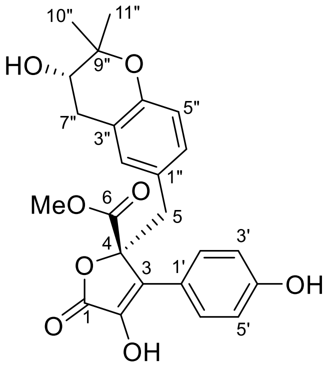<br>Butyrolactone-V (**23**)	
	δ_H, multi. (J in Hz)	δ_C, type	δ_H, multi. (J in Hz)	δ_C, type
1		170.3, C		170.3, C
2		139.7, C		139.7, C
3		129.2, C		126.1, C
4		86.8, C		86.7, C
5	3.43, q (14.6);	39.6, CH_2	3.45, m	39.5, CH_2
6		171.6, C		171.5, C
1′		123.1, C		123.1, C
2′/6′	7.59, d (8.9)	130.4, CH	7.56, dd (8.9, 1.1)	130.36, CH
3′/5′	6.87, d (8.9)	116.6, CH	6.86, dd, (8.9, 1.1)	116.6, CH
4′		159.3, C		159.4, C
1″		125.0, C		126.1 C
2″	6.41, d (2.0)	132.4, CH	6.48, d (2.2)	132.9, CH
3″		128.4, C		120.5, C
4″		155.1, C		153.4, C
5″	6.49, d (8.1)	115.0, CH	6.47, d (8.4)	117.2, CH
6″	6.54, dd (8.1, 2.0)	129.8, CH	6.54, dd (8.4, 2.2)	130.43, CH
7″	3.08, d (7.0)	28.7, CH_2	2.51, ddd (16.8, 11.4, 7.5); 2.79, td (16.8, 5.4)	32.0, CH_2
8″	5.07, tt (7.0, 1.1)	123.6, CH	3.65, m	70.4, CH
9″		133.0, C		78.0, C
10″	1.67, s	25.9, CH_3	1.26, s	25.8, CH_3
11″	1.57, s	17.8, CH_3	1.17, s	20.9, CH_3
6-OMe	3.78, s	53.8, CH_3	3.78, s	53.9, CH_3

Table S2.11. The ^{1}H NMR (400 MHz) and ^{13}C NMR (100 MHz) data of **24** (in $CDCl_3$) and **25** (in acetone-d_6)

Position	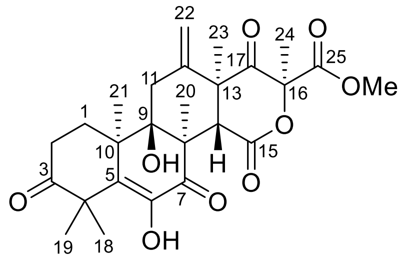<br>Terretonin (**24**)		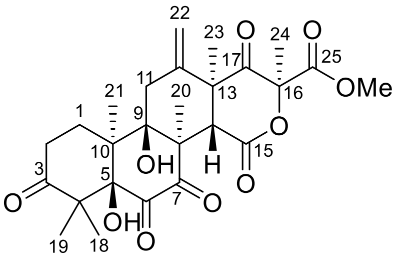<br>Terretonin B (**25**)	
	δ_H, multi. (J in Hz)	δ_C, type	δ_H, multi. (J in Hz)	δ_C, type
1	α 1.76, dd (13.9, 8.4);<br>β 2.36, m	28.3, CH_2	α 1.91, ddd (13.2, 7.5, 2.5));<br>β 2.62, m	27.4, CH_2
2	α 2.52, m;<br>β 2.71, dd (19.0, 8.6)	32.8, CH_2	α 2.37, ddd (16.1, 6.4, 2.5);<br>β 2.71, 2.87, ddd (16.1, 11.4, 7.5)	33.5, CH_2
3		214.2, C		211.8, C
4		48.0, C		53.0, C
5		131.7, C		88.4, C
6		138.8, C		202.0, C
7		197.1, C		201.5, C
8		52.5, C		59.7, C
9		77.7, C		84.5, C
10		43.3, C		48.3, C
11	α 2.97, d (14.3);<br>β 2.27, d (14.3)	35.0, CH_2	α 3.46, dt (14.9, 1.8);<br>β 2.54, d (14.9)	35.8, CH_2
12		139.9, C		140.4 C
13		49.6, C		49.7, C
14	3.54, s	44.7, CH	4.02, s	44.5, CH
15		167.9, C		168.0, C
16		85.7, C		86.4 C
17		201.6, C		202.8, C
18	1.47, s	21.4, CH_3	1.50, s	21.4, CH_3
19	1.47, s	23.6, CH_3	1.15, s	23.7, CH_3
20	1.93, s	20.0, CH_3	1.86, s	17.2, CH_3
21	1.21, s	18.7, CH_3	1.63, s	21.1, CH_3
22	a 5.08, s;<br>b 5.47, s	117.3, CH_2	a 5.45, br s；<br>b 5.04, br s	116.9, CH_2
23	1.44, s	23.7, CH_3	1.44, s	24.9, CH_3
24	1.72, s	21.4, CH_3	1.64, s	21.9, CH_3
25		168.7, C		168.9, C
25-OMe	3.80, s	53.9, CH_3	3.77, s	54.0, CH_3

Table S2.12. The ^{1}H NMR (400 MHz) and ^{13}C NMR (100 MHz) data of **26** and **27** in $CDCl_3$

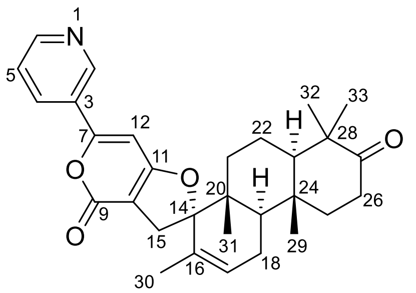

Decaturin D (**26**)

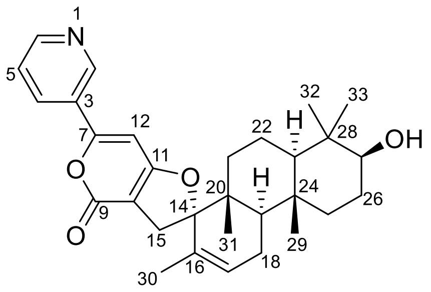

Decaturin E (**27**)

Position	Decaturin D (**26**)		Decaturin E (**27**)	
	δ_H, multi. (J in Hz)	δ_C, type	δ_H, multi. (J in Hz)	δ_C, type
2	8.98, s	146.9, CH	9.02, s	147.1, CH
3		127.6, C		127.8, C
4	8.08, d (7.8)	133.2, CH	8.14, d (8.1)	133.4, CH
5	7.36, dd (7.7, 3.6)	123.7, CH	7.40, dd (8.1, 4.5)	123.8, CH
6	8.62, d (3.6)	151.5, CH	8.66, d (4.5)	151.6, CH
7		160.8, C		161.1, C
9		170.2, C		170.4, C
10		102.0, C		102.2, C
11		160.2, C		160.2, C
12	6.61, s	93.8, CH	6.66, s	94.1, CH
14		100.8, C		101.3, C
15	a 3.06, d (16.2);<br>b 2.91, d (16.2)	28.4, CH_2	a 3.10, d (16.2);<br>b 2.94, d (16.2)	28.5, CH_2
16		131.4, C		131.3, C
17	5.67, br s	128.4, CH	5.70, br s	128.8, CH
18	2.03, m	23.2, CH_2	2.03, m	23.2, CH_2
19	1.73, dd (13.0, 7.4)	46.9, CH	1.58, m	47.8, CH
20		40.9, C		41.0, C
21	a 1.54, m;<br>b 1.42, m	31.7, CH_2	a 1.56, m;<br>b 1.28, m	32.4, CH_2
22	1.51, m	19.0, CH_2	a 1.52, m;<br>b 1.41, m	17.9, CH_2
23	1.37, m	54.5, CH	0.84, dd (11.2)	54.7, CH
24		36.5, C		36.9, C
25	a 1.90, ddd (13.3, 7.3, 3.9);<br>b 1.52, m	38.7, CH_2	a 1.07, td (12.4, 4.3);<br>b 1.60, m	38.3, CH_2
26	a 2.51, ddd (15.8, 10.6, 7.4);<br>b 2.37, ddd (15.8, 6.6, 3.6)	34.0, CH_2	1.48, m	27.3, CH_2
27		217.0, C	3.24, dd (11.2, 4.6)	78.9, CH
28		47.3, C		38.9, C
29	1.03, s	15.5, CH_3	0.98, s	15.7, CH_3
30	1.65, s	18.5, CH_3	1.68, s	18.7, CH_3
31	0.94, s	16.0, CH_3	0.96, s	15.8, CH_3
32	1.01, s	21.4, CH_3	0.79, s	16.5, CH_3
33	1.03, s	26.5, CH_3	0.93, s	28.2, CH_3

Table S2.13. The ^{1}H NMR (400 MHz) and ^{13}C NMR (100 MHz) data of **28** in $CDCl_3$

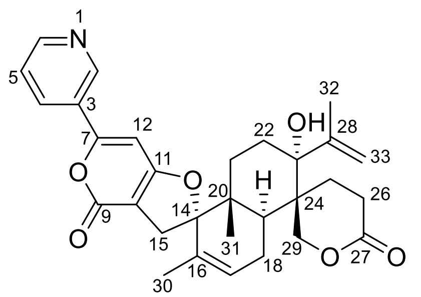

15-Deoxyoxalicine B (**28**)

Position	δ_H, multi. (J in Hz)	δ_C, type	Position	δ_H, multi. (J in Hz)	δ_C, type
2	8.95, br s	146.9, CH	19	2.67. dd (11.2, 6.0)	42.0, CH
3		127.62, C	20		40.3, C
4	8.08, d (7.9)	133.3, CH	21	a 1.49, d (14.6);<br>b 2.33, m	24.9, CH_2
5	7.39, dd (7.9, 4.5)	123.8, CH	22	a 1.29, d (13.2);<br>b 2.04, td (13.2, 4.0)	29.0, CH_2
6	8.63, d (4.5)	151.5, CH	23		76.3, C
7		160.8, C	24		44.1, C
9		170.3, C	25	a 1.61, dt (14.7, 4.2);<br>b 2.47, m	25.8, CH_2
10		102.0, C	26	a 2.23, m;<br>b 2.43, m	29.9, CH_2
11		160.3, C	27		173.5, C
12	6.61, s	93.8, CH	28		150.5, C
14		100.2, C	29	a 4.44, d (12.7);<br>b 4.36, d (12.7)	67.6, CH_2
15	a 3.09, d (16.2);<br>b 2.94, d (16.2)	28.5, CH_2	30	1.68, s	18.5, CH_3
16		131.7, C	31	0.91, s	15.9, CH_3
17	5.71, br s	127.56, CH	32	a 5.17, s;<br>b 5.05, s	115.3, CH_2
18	2.22, m	24.3, CH_2	33	1.87, s	21.7, CH_3

Table S2.14. The ^{1}H NMR and ^{13}C NMR data of **29** and **30** (in $CDCl_3$)

Position	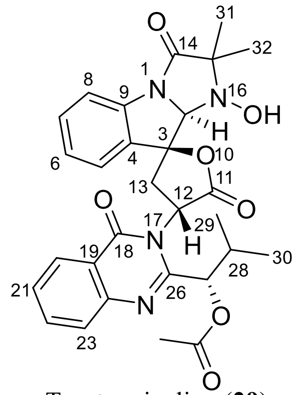 Tryptoquivaline (**29**)		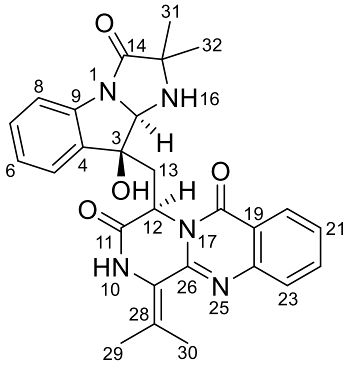 Quinadoline A (**30**)	
	δ_H, multi. (*J* in Hz)[a]	δ_C, type	δ_H, multi. (*J* in Hz)*b*	δ_C, type
2	5.02, s	87.8, CH	5.28, d (7.3)	78.4, CH
3		84.2, C		74.2, C
4		133.7, C		137.5, C
5	7.52, d (7.5)	124.2, CH	7.26, d (7.5)	124.1, CH
6	7.26, t (7.5)	125.4, CH	7.07, t (7.5)	125.3, CH
7	7.51, t (7.7)	131.8, CH	7.29, t (7.7)	130.2, CH
8	7.65, d (7.8)	115.8, CH	7.52, d (7.9)	115.9, CH
9		137.7, C		138.0, C
11		169.8, C		168.5, C
12	5.82, t (9.8)	54.9, CH	5.81, dd (7.7, 4.6)	51.3, CH
13	a 3.28, dd (13.5, 10.6); b 3.09, dd (13.5, 9.4)	33.8, CH_2	a 2.65, dd (14.6, 4.6); b 2.28, dd (14.6, 8.3)	40.2, CH_2
14		171.1, C		175.3, C
15		71.4, C		64.9, C
18		161.5, C		160.5, C
19		120.1, C		119.9, C
20	8.29, d (7.8)	126.7, CH	8.28, dd (8.1, 1.5)	127.0, CH
21	7.61, t (7.5)	127.8, CH	7.48, t (7.6)	127.5, CH
22	7.90, t (7.4)	135.4, CH	7.76, ddd (8.2, 7.0, 1.5)	134.9, CH
23	7.7.83, d (8.1)	128.0, CH	7.69, d (8.2)	127.6, CH
24		146.5, C		147.1, C
26		152.8, C		145.7, C
27	5.73, d (8.5)	76.9, CH		120.9, C
28	2.70, m	31.7, CH		132.7, C
29	1.08, d (6.7)	18.7, CH_3	2.01, s	22.0, CH_3
30	1.20, d (6.6)	18.3, CH_3	2.42, s	21.3, CH_3
31	1.57, s	17.1, CH_3	1.42, s	26.5, CH_3
32	1.60, s	22.9, CH_3	1.55, s	25.6, CH_3
27-OAc	2.27, s	170.8, C; 20.7, CH_3		
3-OH			4.92, s	
10-NH			8.56, s	

[a] Measured at 400 MHz for ^{1}H NMR, 100 MHz for ^{13}C NMR; [b] Measured at 500 MHz for ^{1}H NMR, 125 MHz for ^{13}C NMR.

Table S2.15. The ^{1}H NMR (400 MHz) and ^{13}C NMR (100 MHz) data of **31** (in pyridine-d_5) and **32** (in CDCl$_3$ + CD$_3$OD)

Position	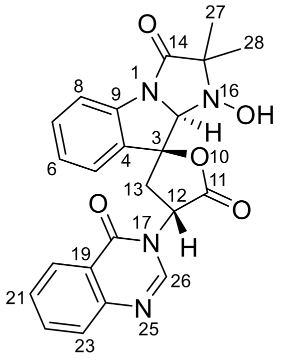 Tryptoquivaline L (**31**)		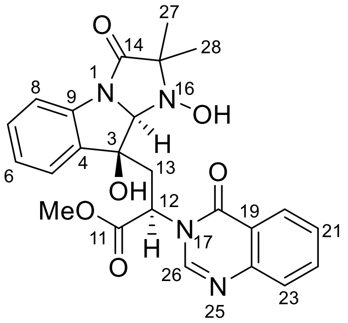 Tryptoquivaline R (**32**)	
	δ_H, multi. (J in Hz)	δ_C, type	δ_H, multi. (J in Hz)	δ_C, type
2	5.38, s	87.7, CH	5.01, s	85.9, CH
3		84.7, C		78.3, C
4		133.1, C		138.3, C
5	8.09, d (7.6)	127.08, CH	7.86, m, overlapped	125.5, CH
6	7.21, m, overlapped	126.0, CH	7.13, t (7.4)	126.3, CH
7	7.73, t (7.6)	132.5, CH	7.33, m, overlapped	130.9, CH
8	7.21, m, overlapped	116.0, CH	7.49, d (8.8)	116.3, CH
9		140.2, C		138.6, C
11		171.6, C		171.1, C
12	6.05, t (9.8)	58.8, CH	5.44, dd (6.8, 2.7)	60.0, CH
13	a 3.90, t (12.5, 8.9); b 3.25, dd (12.5, 8.2)	36.7, CH$_2$	a 2.99, dd (15.8, 2.7); b 2.90, dd (15.8, 6.7)	39.2, CH$_2$
14		172.8, C		174.6, C
15		71.7, C		71.7, C
18		161.2, C		162.3, C
19		122.9, C		122.9, C
20	8.45, d (7.9)	127.15, CH	8.26, dd (8.0, 1.5)	127.4, CH
21	7.46, m	128.5, CH	7.59, t (7.7)	128.5, CH
22	7.85, m, overlapped	135.3, CH	7.86, m, overlapped	135.9, CH
23	7.85, m, overlapped	128.1, CH	7.73, d (8.3)	127.8, CH
24		148.9, C		149.6, C
26	8.77, s	147.8, CH		148.9, C
27	1.61, s	16.6, CH$_3$	1.42, s	16.8, CH$_3$
28	1.42, s	23.5, CH$_3$	1.36, s	22.9, CH$_3$
11-OMe			3.74, s	53.6, CH$_3$
16-OH	11.19, s		8.44, s	

Table S2.16. The ^{1}H NMR and ^{13}C NMR data of **33** (in $CDCl_3$) and **34** (in CD_3OD)

Position	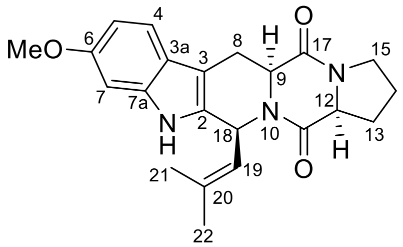 Fumitremorgin C (**33**)		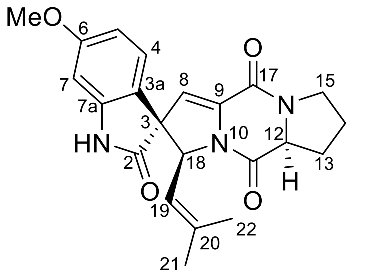 6-Methoxyspirotryprostatin B (**34**)	
	δ_H, multi. (*J* in Hz)[a]	δ_C, type	δ_H, multi. (*J* in Hz)[b]	δ_C, type
2		132.3, C		181.1, C
3		106.5 C		62.9, C
3a		120.9, C		120.3, C
4	7.44, d (8.6)	119.0, CH	7.02, d (8.3)	129.5, CH
5	6.81, dd (8.6, 2.2)	109.7, CH	6.56, dd (8.3, 1.9)	108.1, CH
6		156.7, C		162.4, C
7	6.86, d (2.2)	95.4, CH	6.50, br s	98.2, CH
7a		137.1, C		144.2, C
8	a 3.51, dd (15.9, 5.0); b 3.10, dd (15.9, 11.5)	22.1, CH_2	5.27, s	118.6, CH
9	4.19, dd (11.5, 5.0)	56.9, CH		138.9, C
11		169.7, C		164.9, C
12	4.12, t (8.2)	59.4, CH	4.45, dd (10.8, 6.0)	63.0, CH
13	a 2.41, m; b 2.23, m	28.7, CH_2	a 2.41, m; b 1.98, m	30.0, CH_2
14	a 2.06, m; b 1.95, m	23.2, CH_2	a 2.12, m; b 1.98, m	22.9, CH_2
15	3.64, m	45.6, CH_2	a 3.78, m; b 3.55, m	46.0, CH_2
17		165.9, C		156.9, C
18	4.90, d (9.5)	51.2, CH	5.27, s	65.3, CH
19	5.98, d (9.5)	124.3, CH	5.74, s	122.2, CH
20		134.2, C		139.0, C
21	1.65, s	25.9, CH_3	1.59, s	25.3, CH_3
22	2.00, s	18.3, CH_3	1.27, s	18.3, CH_3
6-OMe	3.84, s	55.9, CH_3	3.78, s	55.9, CH_3
1-NH	7.73, s			

[a] Measured at 500 MHz for ^{1}H NMR, 125 MHz for ^{13}C NMR; [b] Measured at 400 MHz for ^{1}H NMR, 100 MHz for ^{13}C NMR.

S3. 1D and 2D NMR spectra of **1**−**34**

S3.1. ^{1}H NMR spectrum of **1** in pyridine-d_5

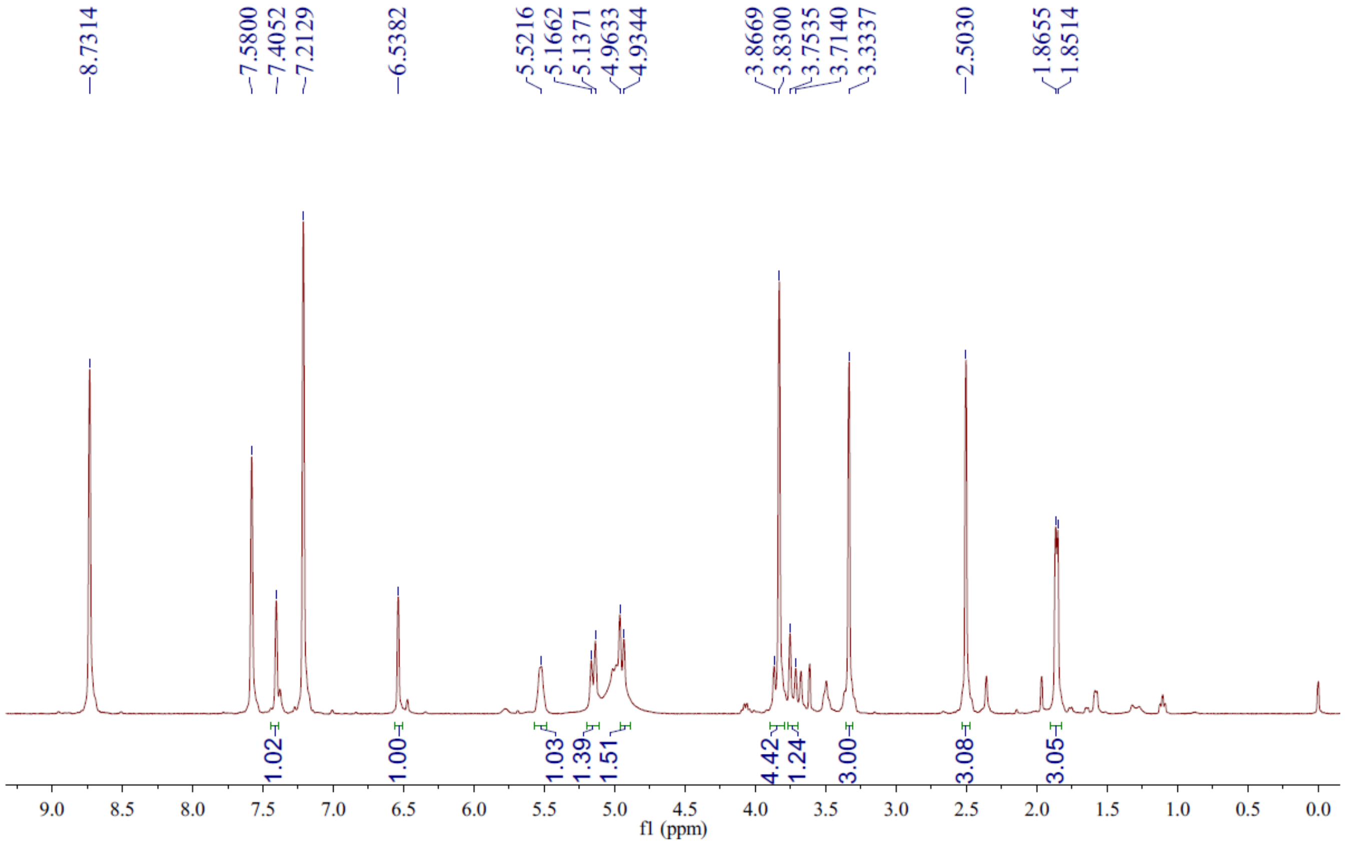

S3.2. ^{13}C NMR and DEPT spectra of **1** in pyridine-d_5

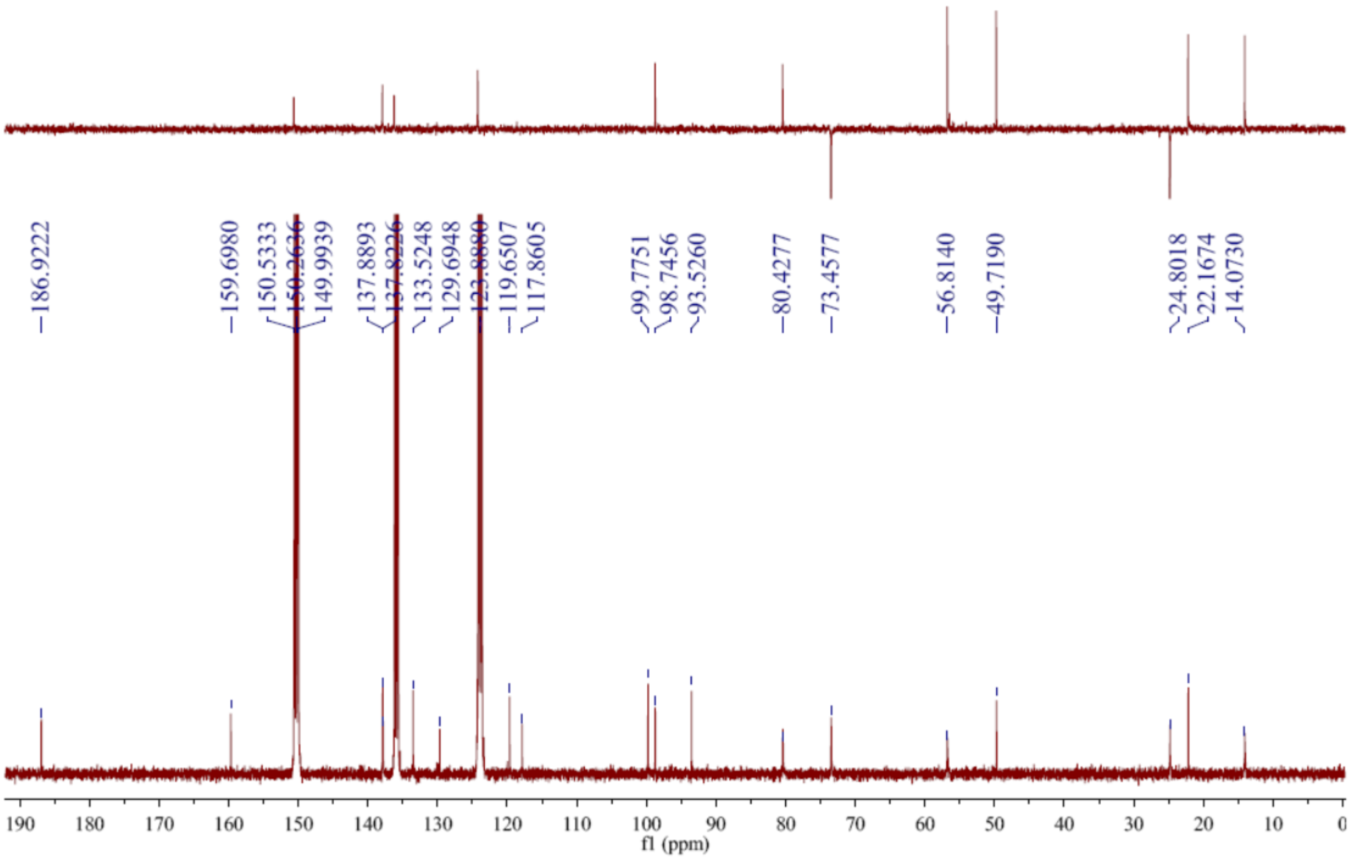

S3.3. HSQC spectrum of **1** in pyridine-d_5

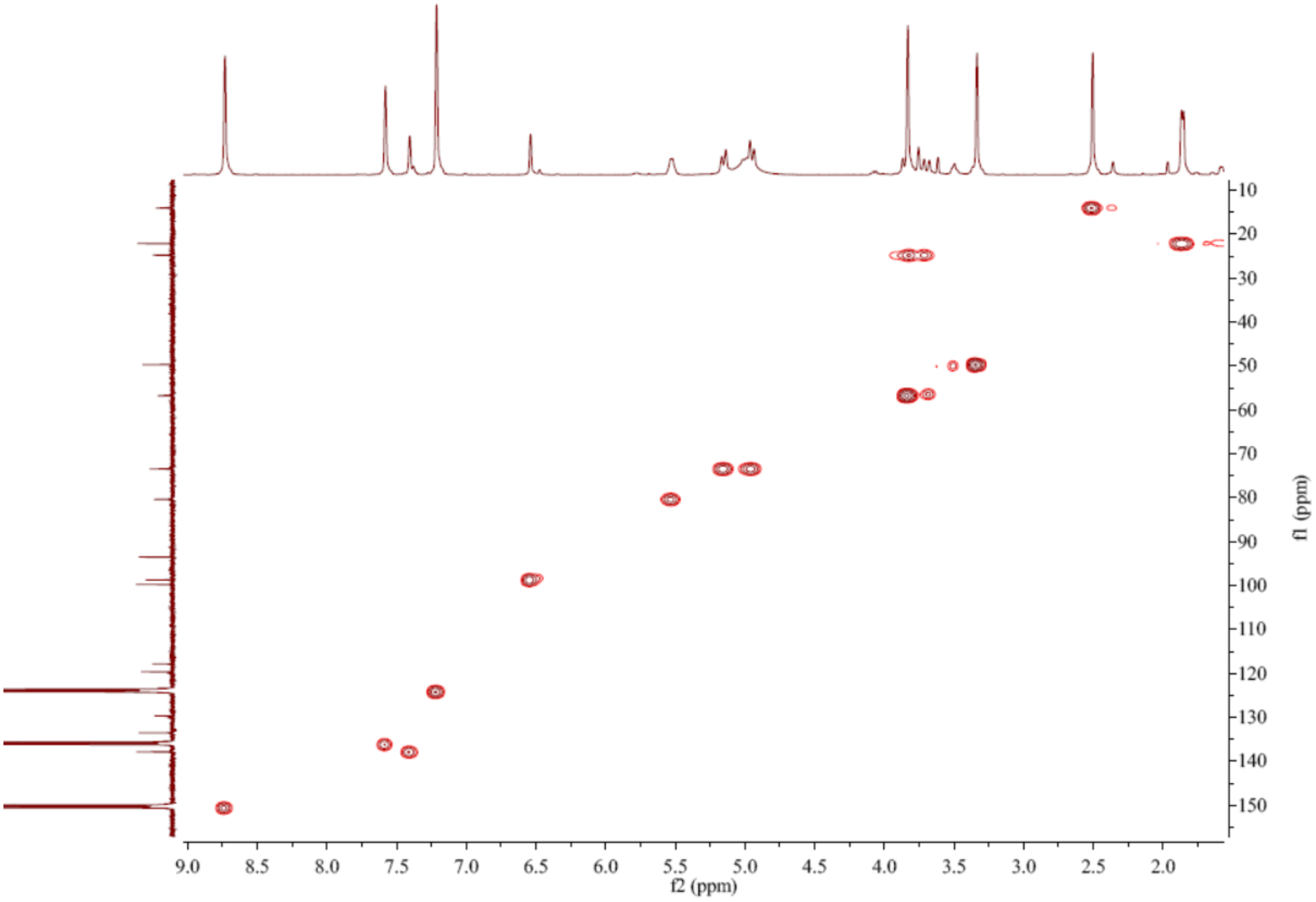

S3.4. HMBC spectrum of **1** in pyridine-d_5

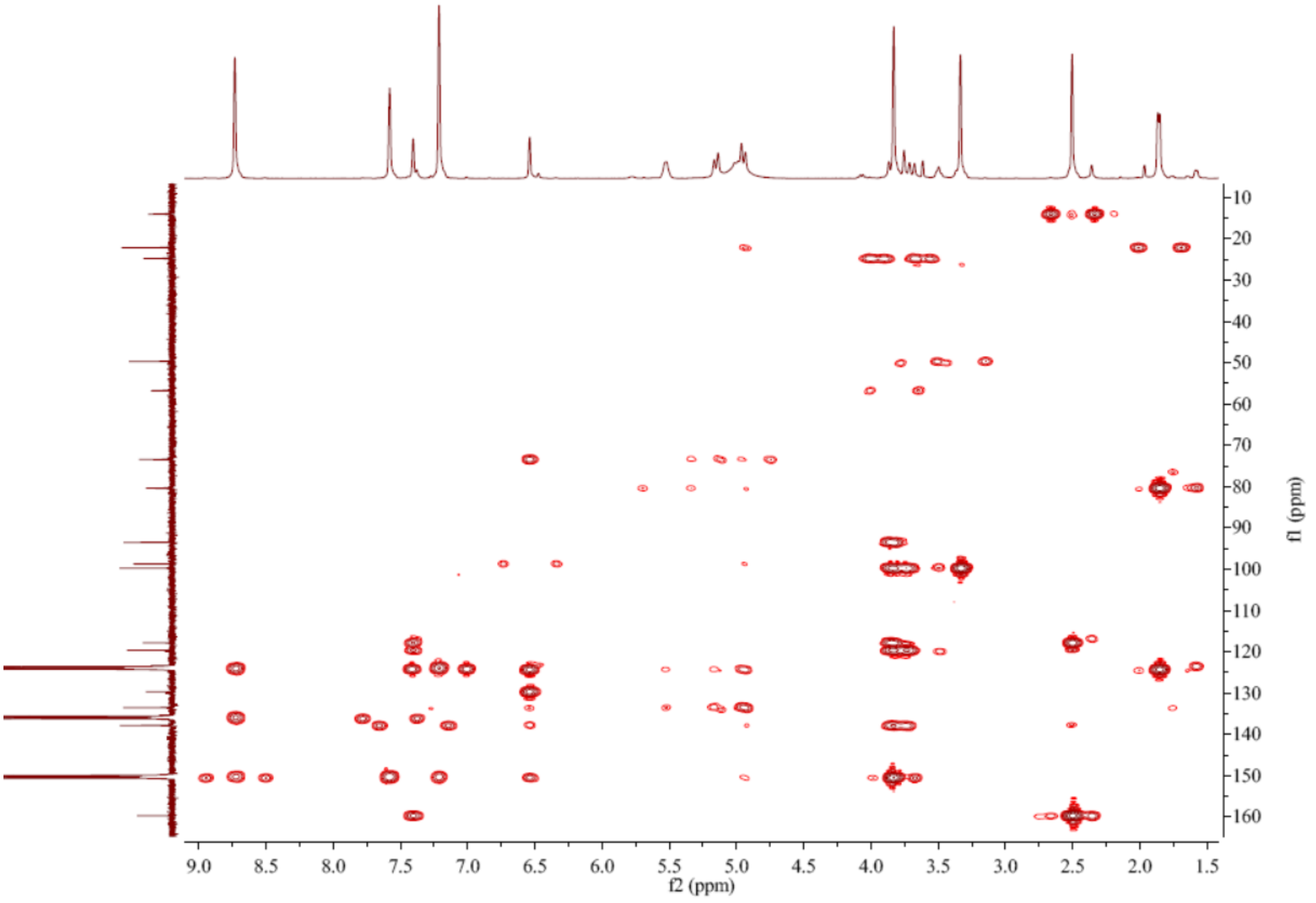

S3.5. ¹H NMR spectrum of **2** in CD_3OD

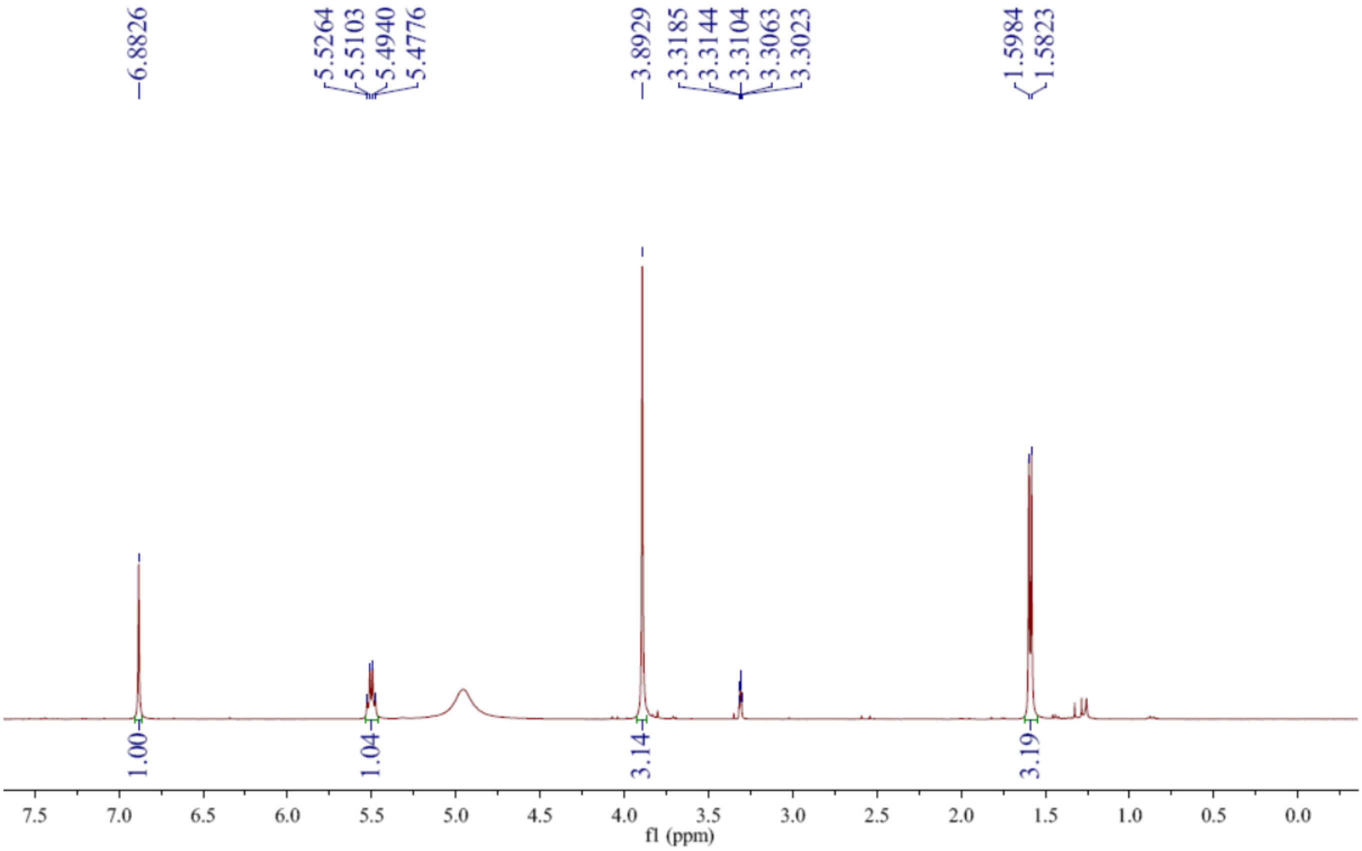

S3.6. ¹³C NMR and DEPT spectra of **2** in CD_3OD

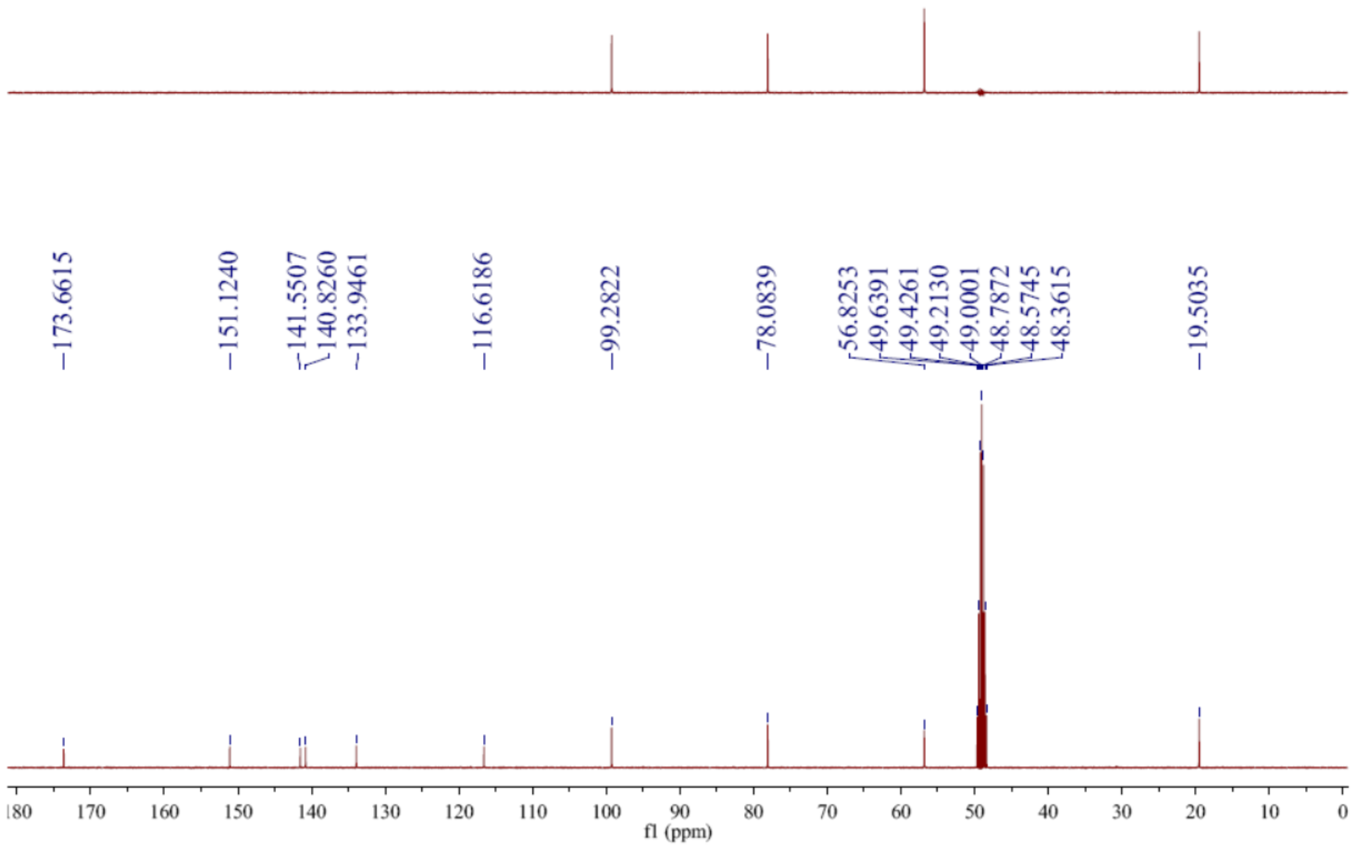

S3.7. HSQC spectrum of **2** in CD_3OD

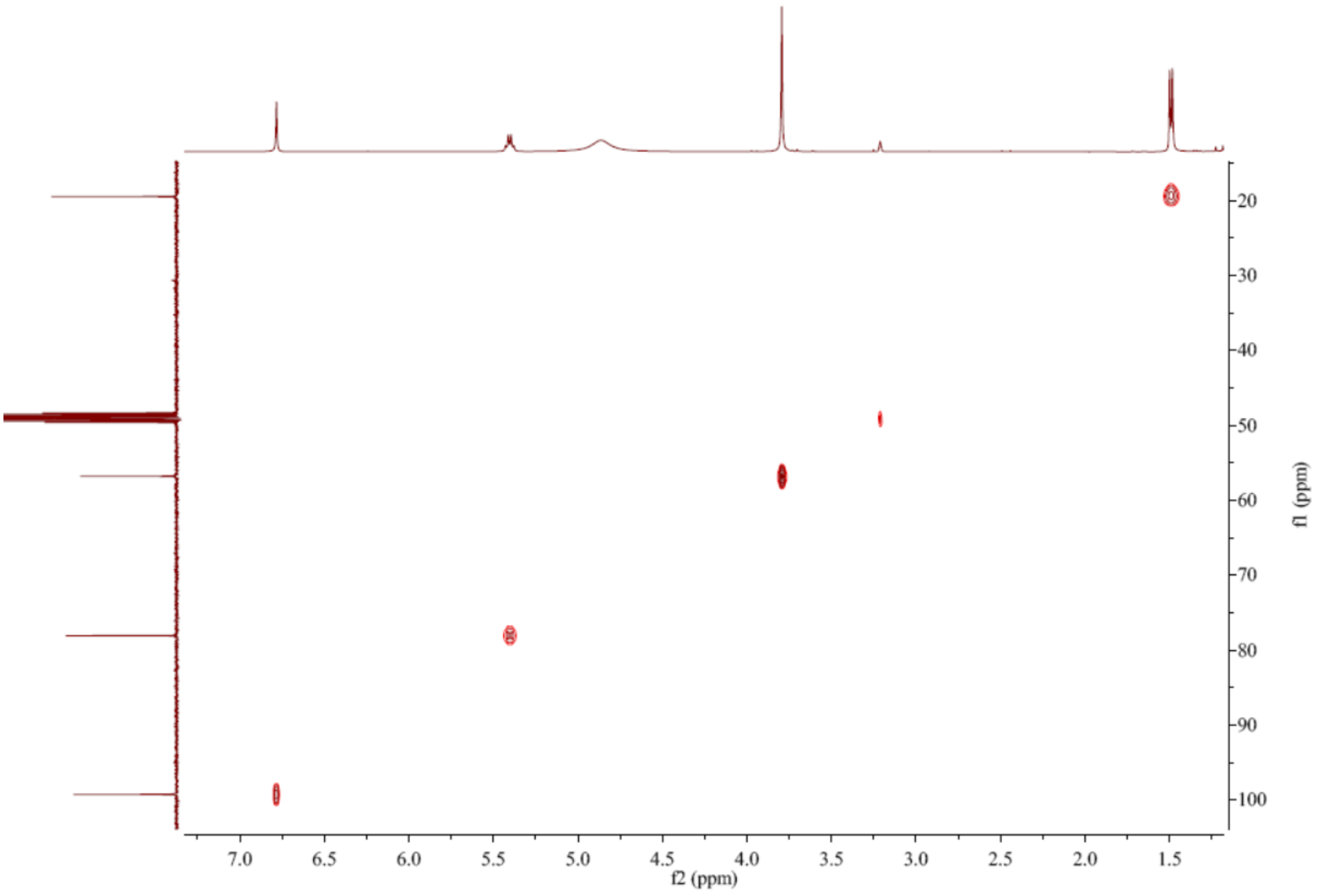

S3.8. HMBC spectrum of **2** in CD_3OD

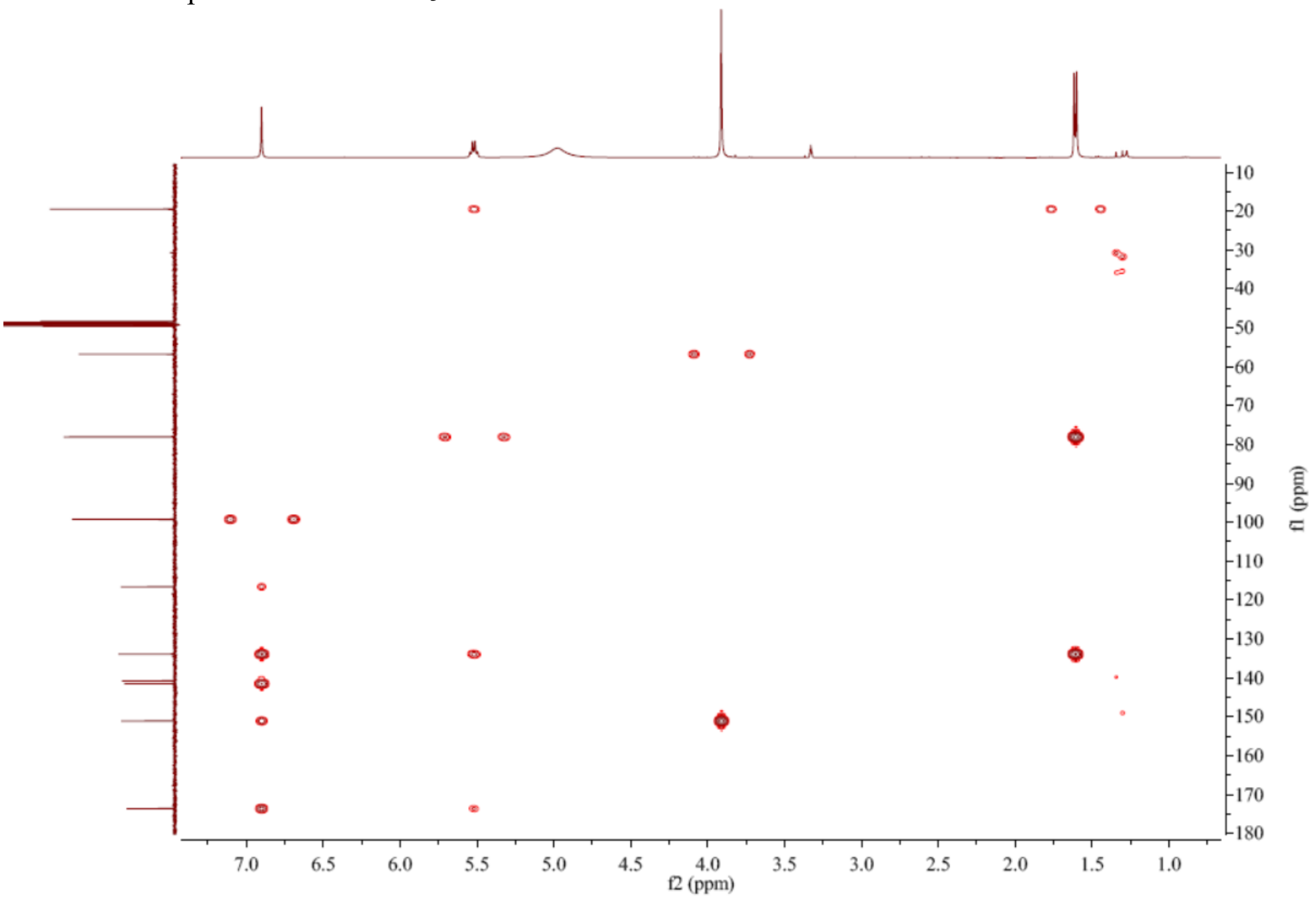

S3.9. ^{1}H NMR spectrum of **3** in $CDCl_3$

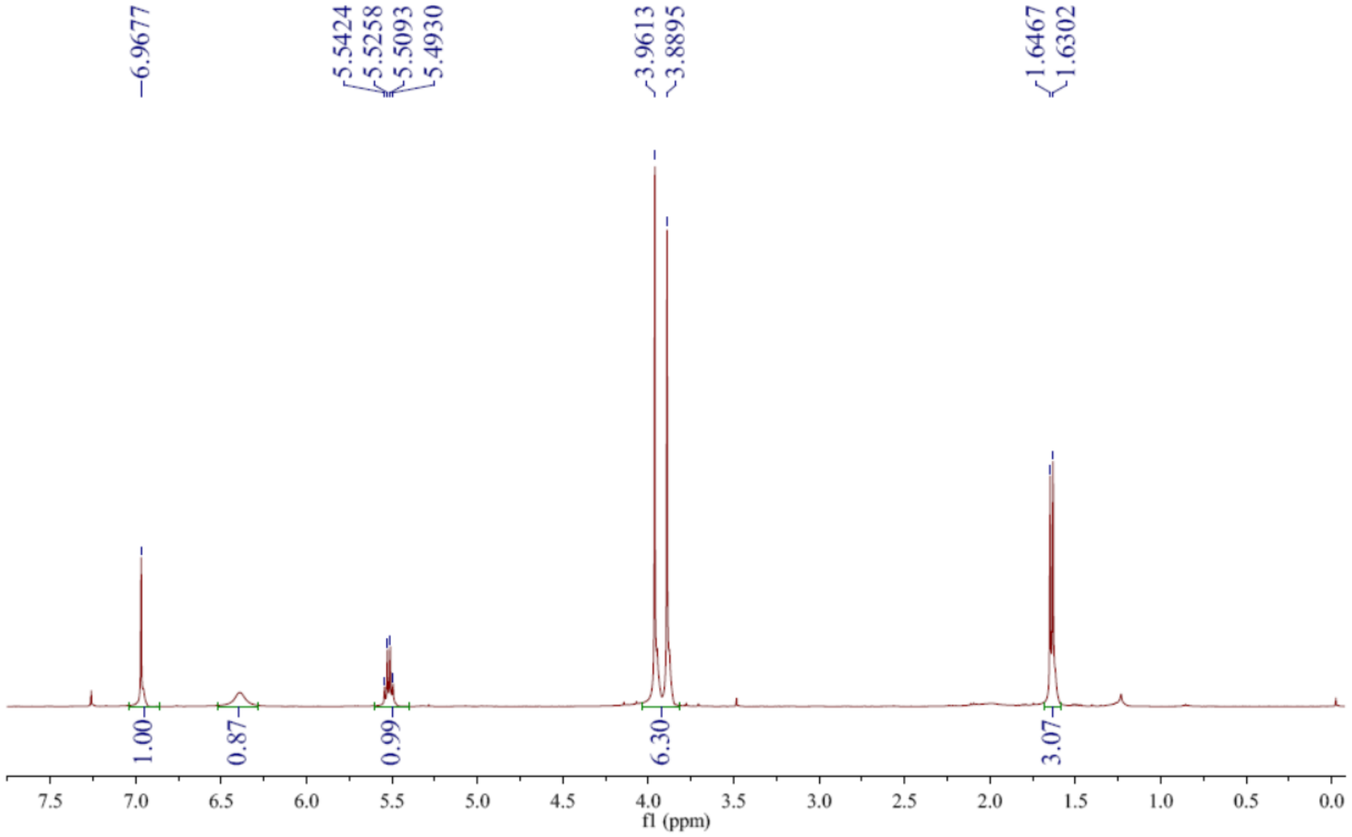

S3.10. ^{1}H NMR and DEPT spectra of **3** in $CDCl_3$

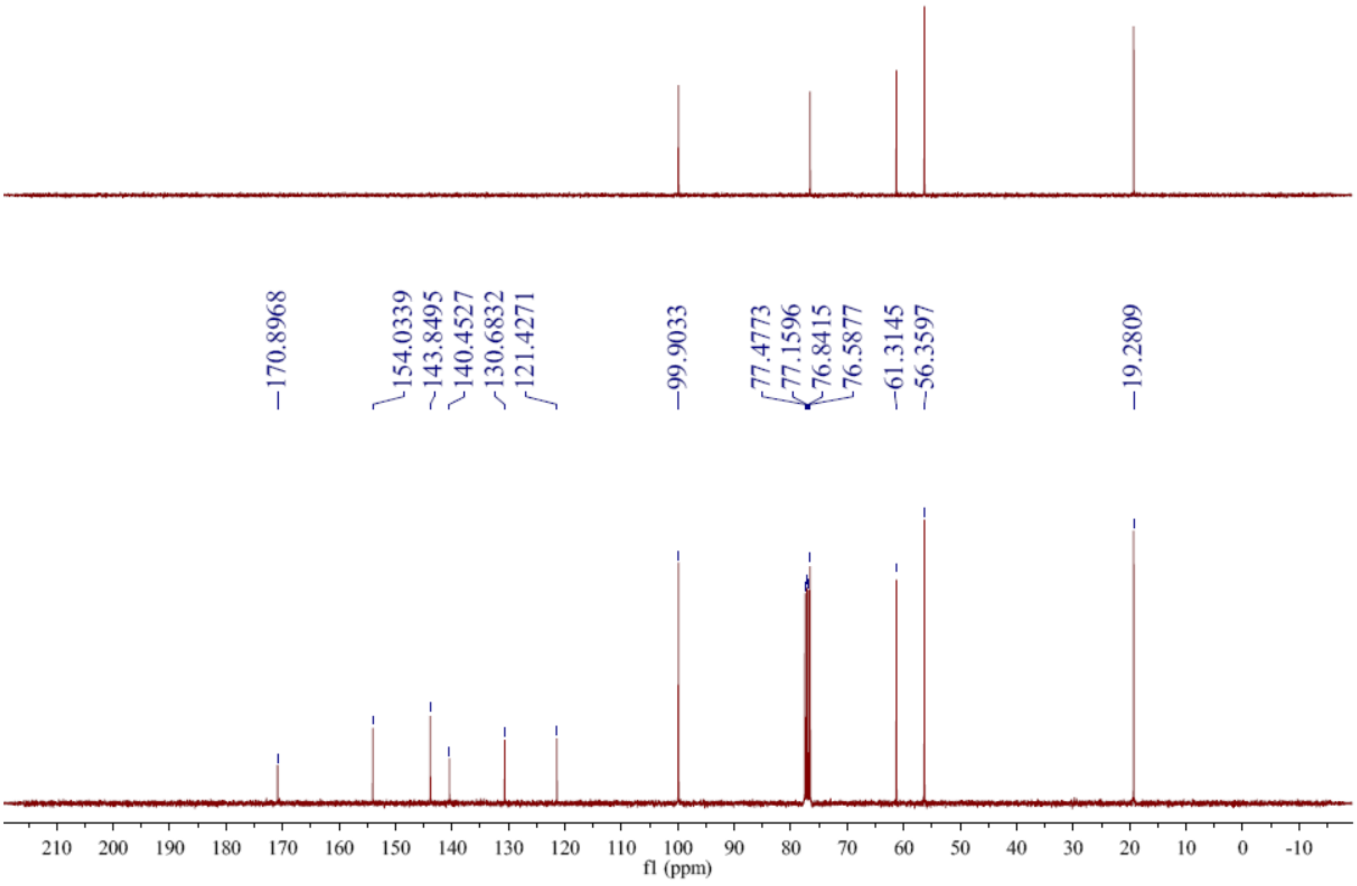

S3.11. HMBC spectrum of **3** in $CDCl_3$

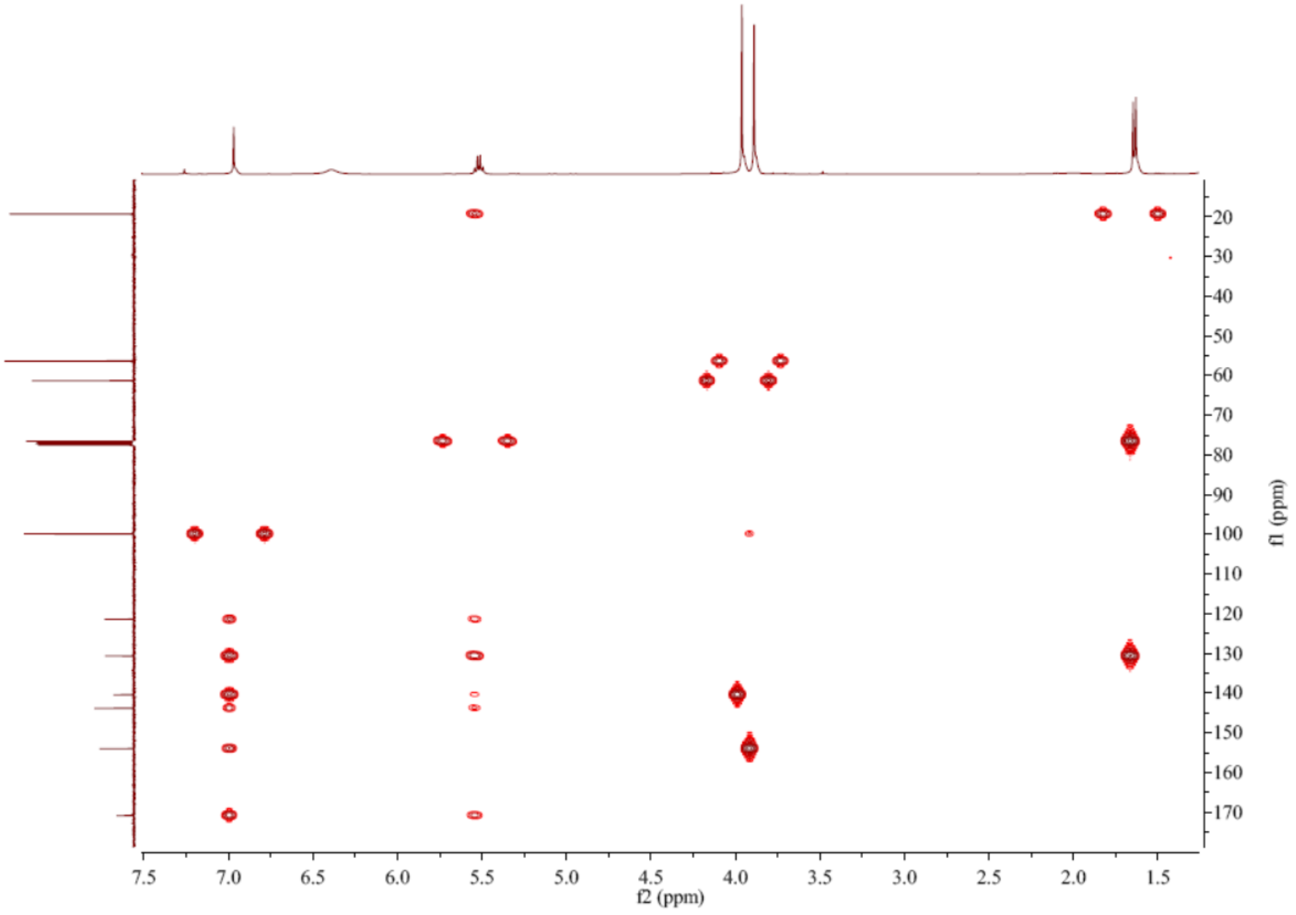

S3.12. ^{1}H NMR spectrum of **4** in in CD_3OD

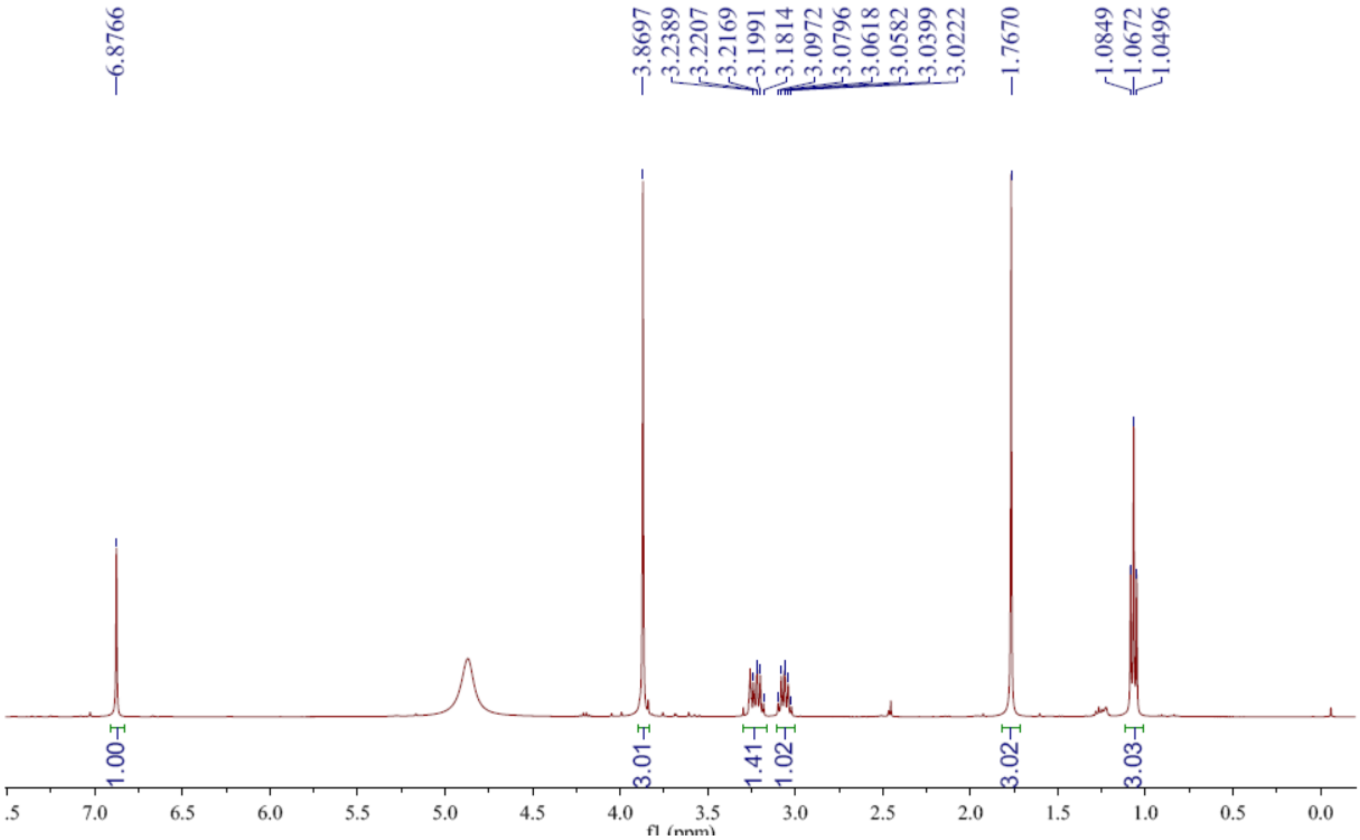

S3.13. ^{13}C NMR and DEPT spectra of **4** in CD_3OD

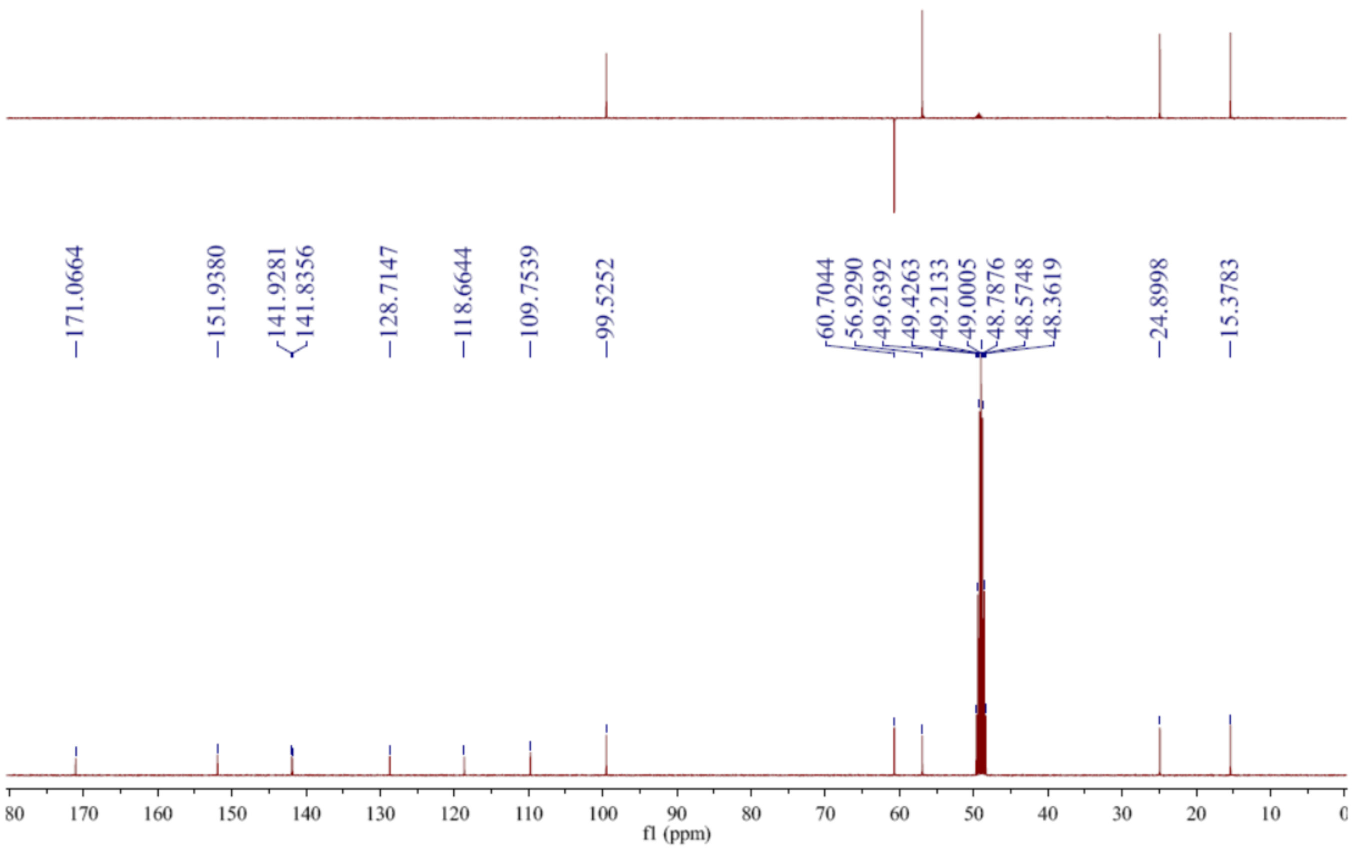

S3.14. HSQC spectrum of **4** in CD_3OD

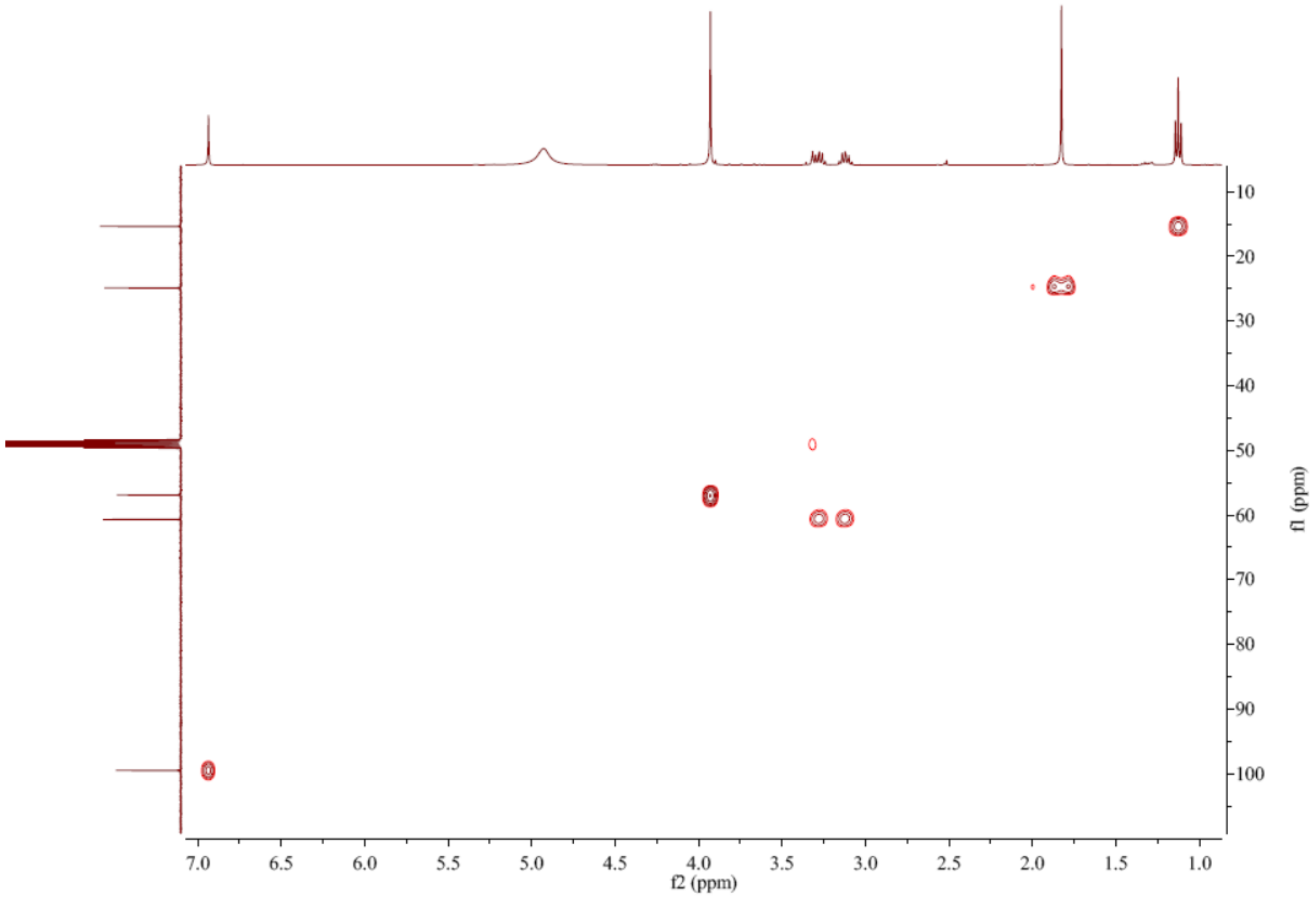

S3.15. $^{1}H-^{1}H$ COSY spectrum of **4** in CD_3OD

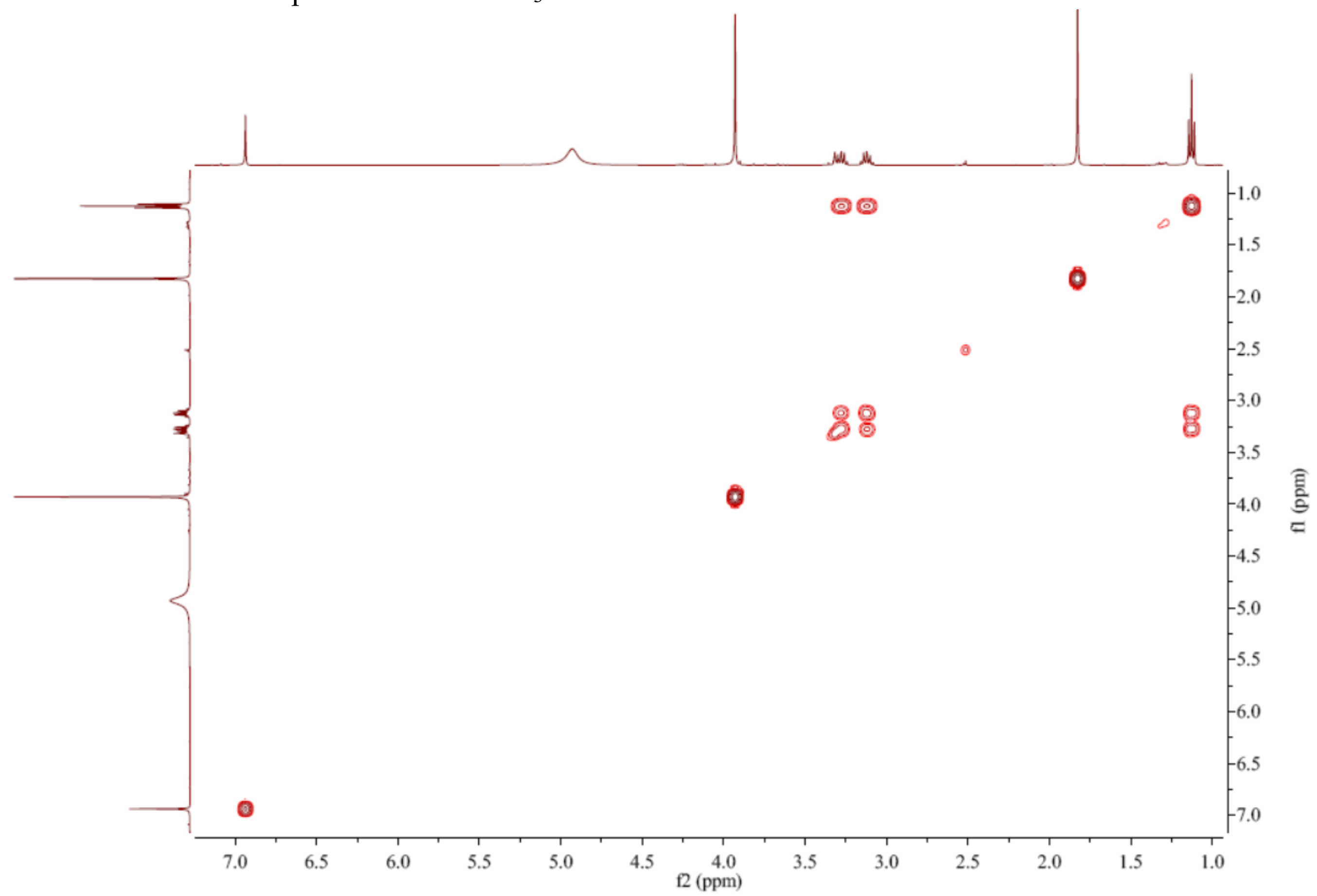

S3.16. HMBC spectrum of **4** in CD_3OD

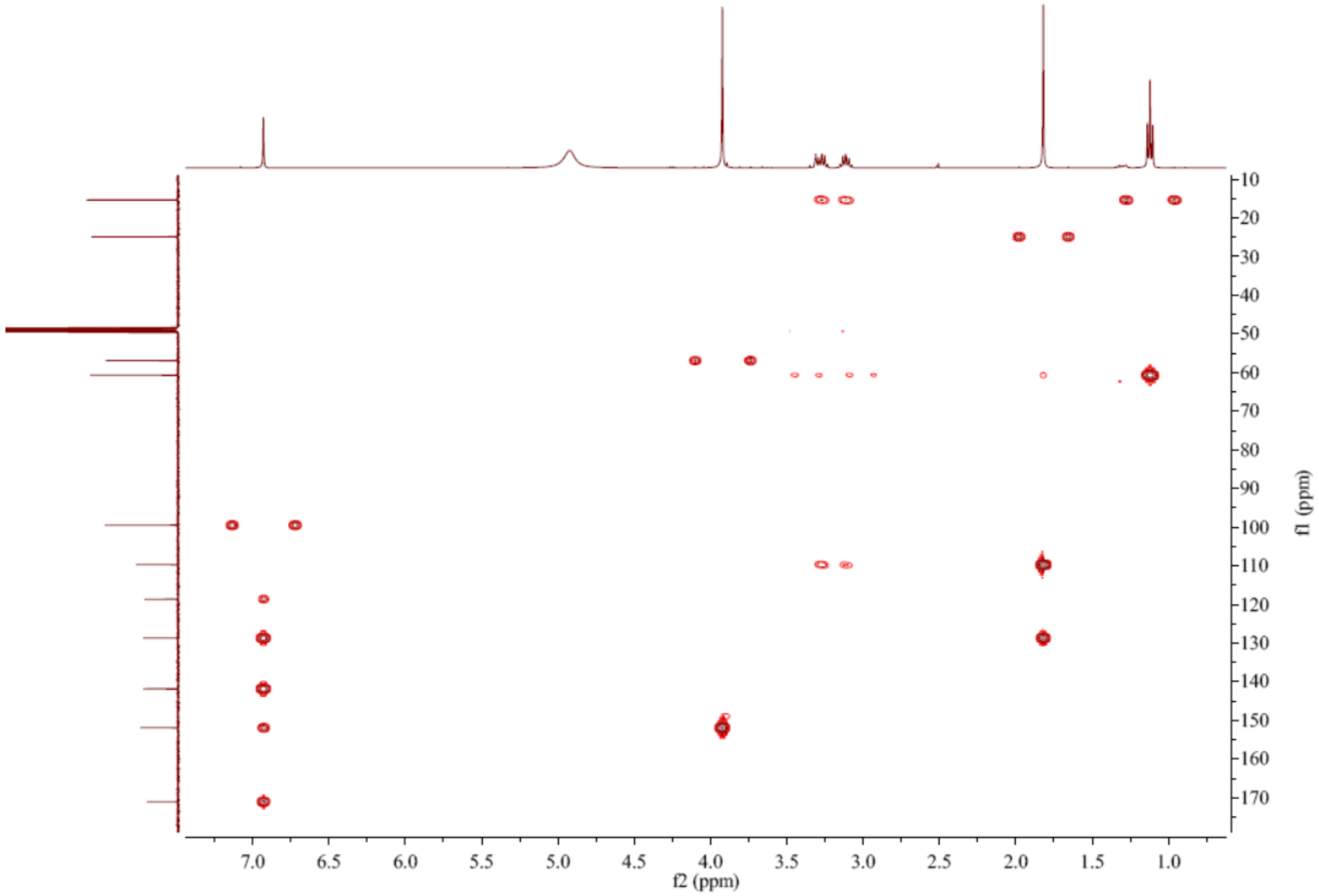

S3.17. ^{1}H NMR spectrum of **5** in $CDCL_3$

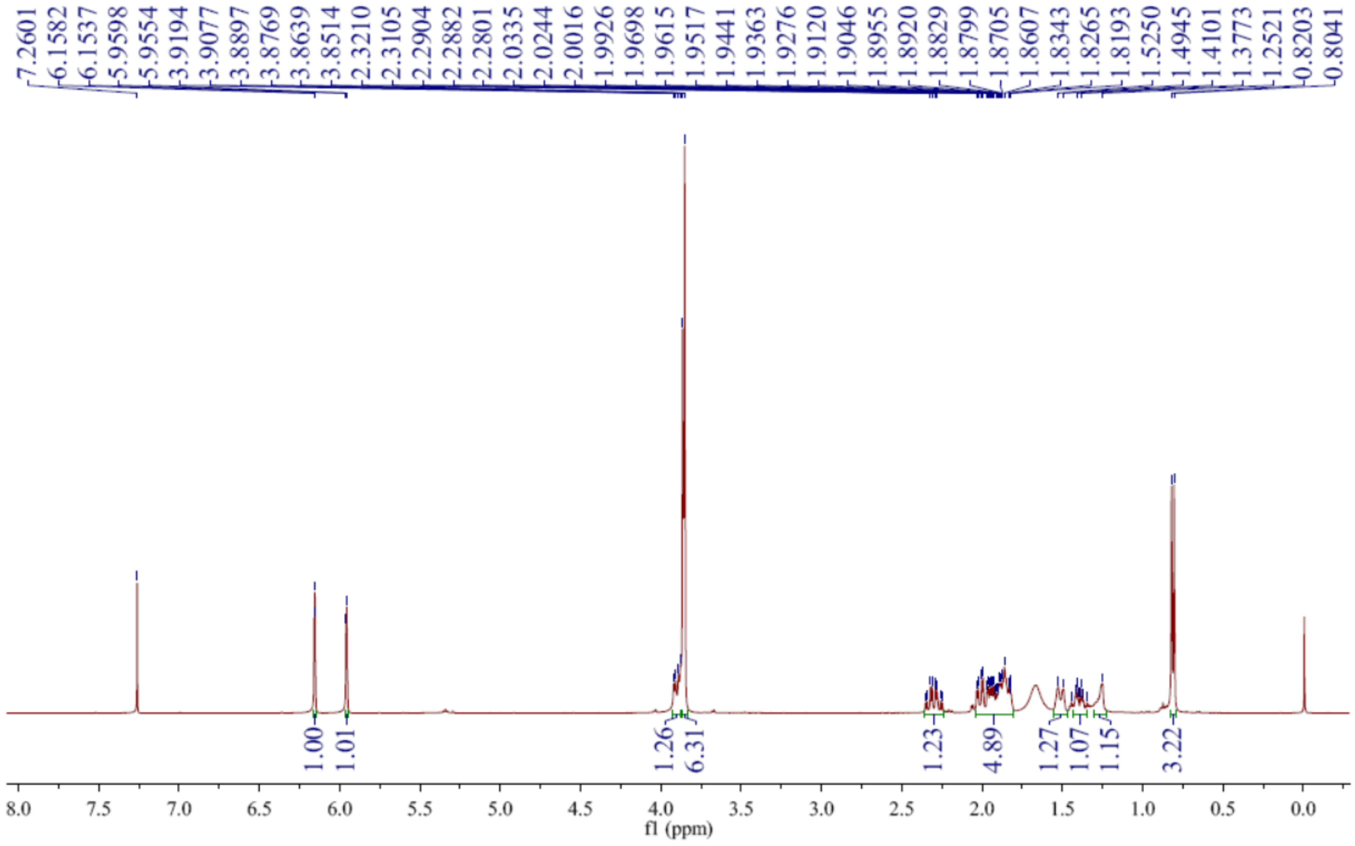

S3.18. ^{13}C NMR and DEPT spectra of **5** in $CDCL_3$

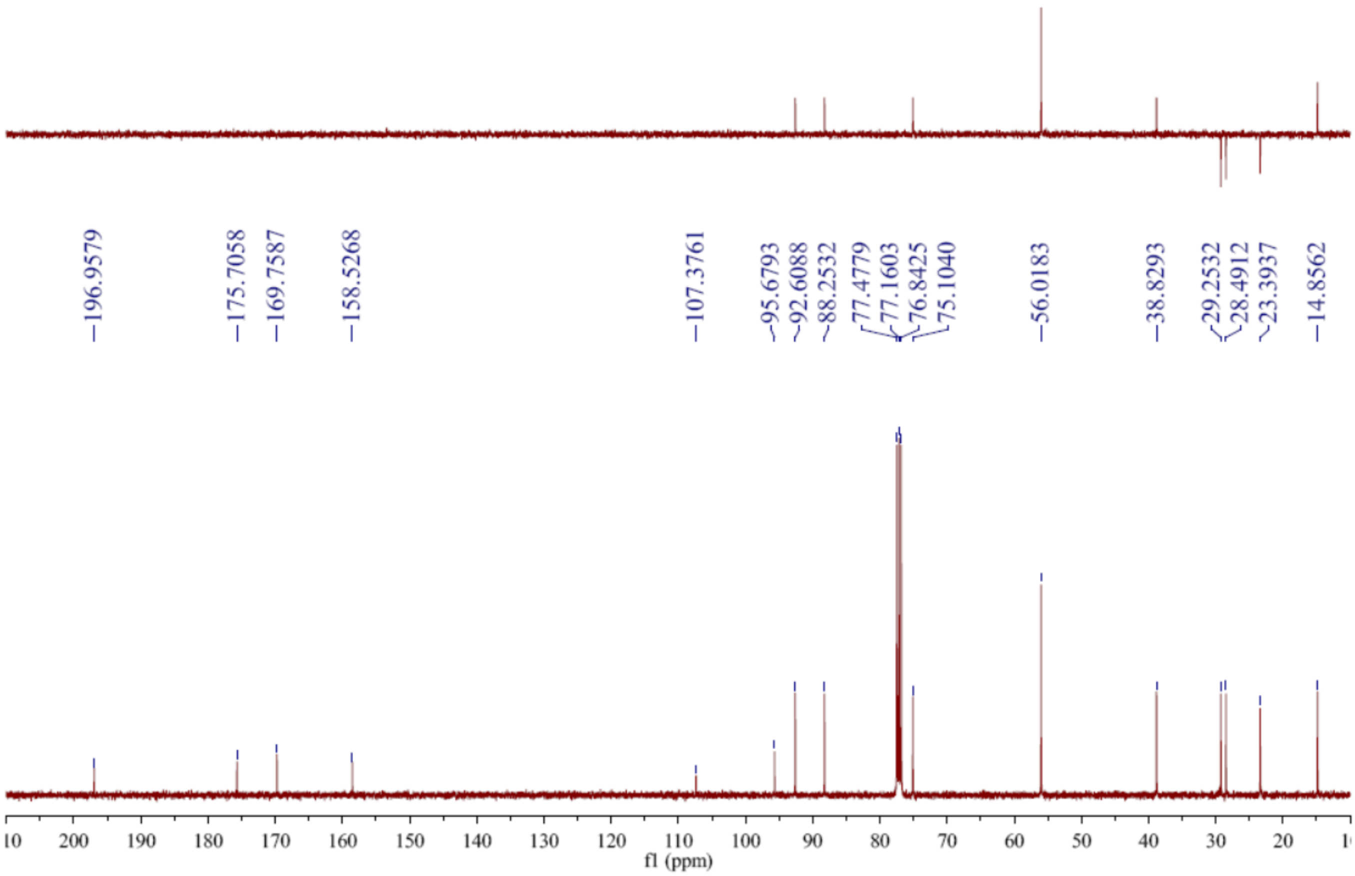

S3.19. HSQC spectrum of **5** in CDCL$_3$

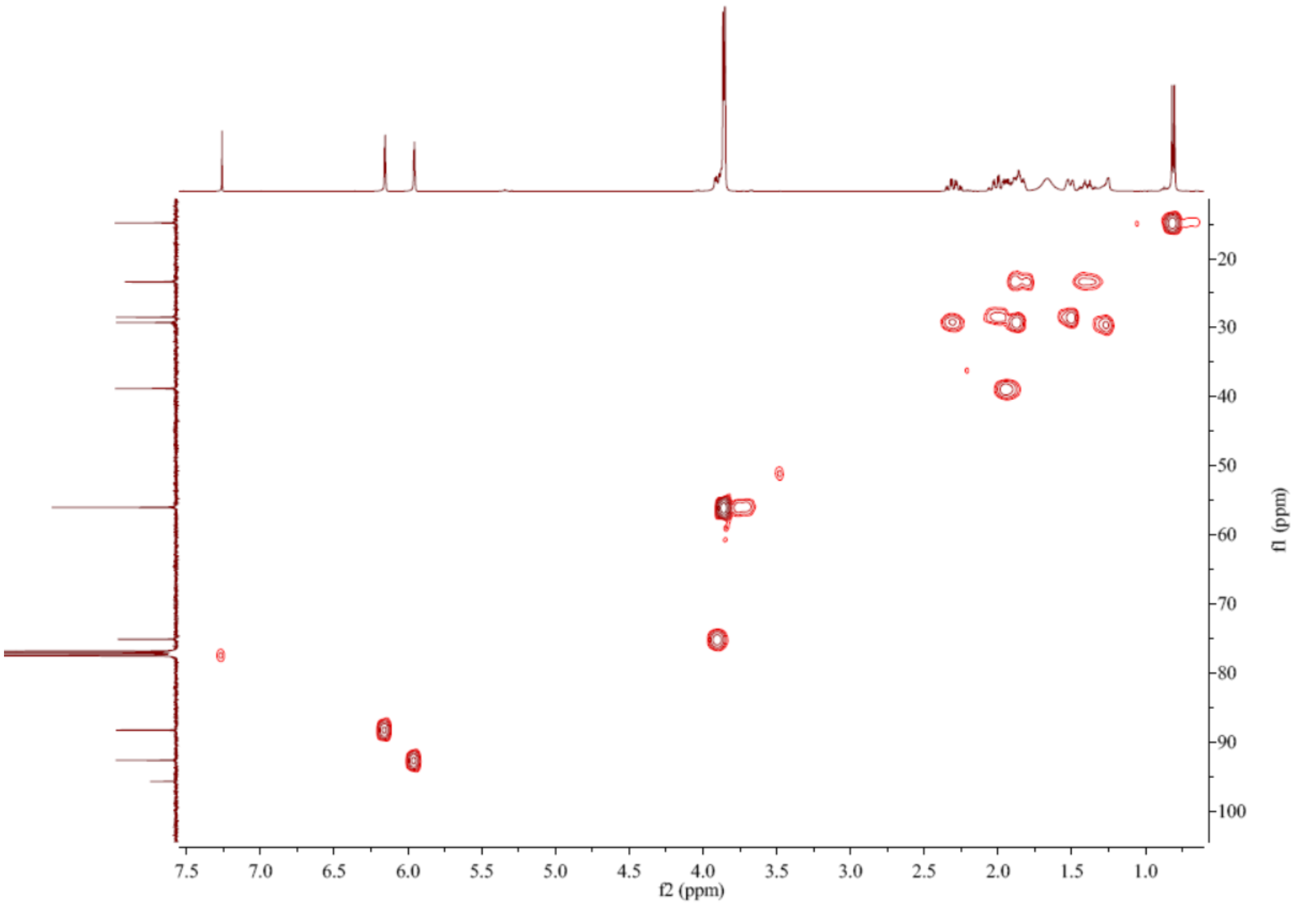

S3.20. $^1H-^1H$ COSY spectrum of **5** in CDCL$_3$

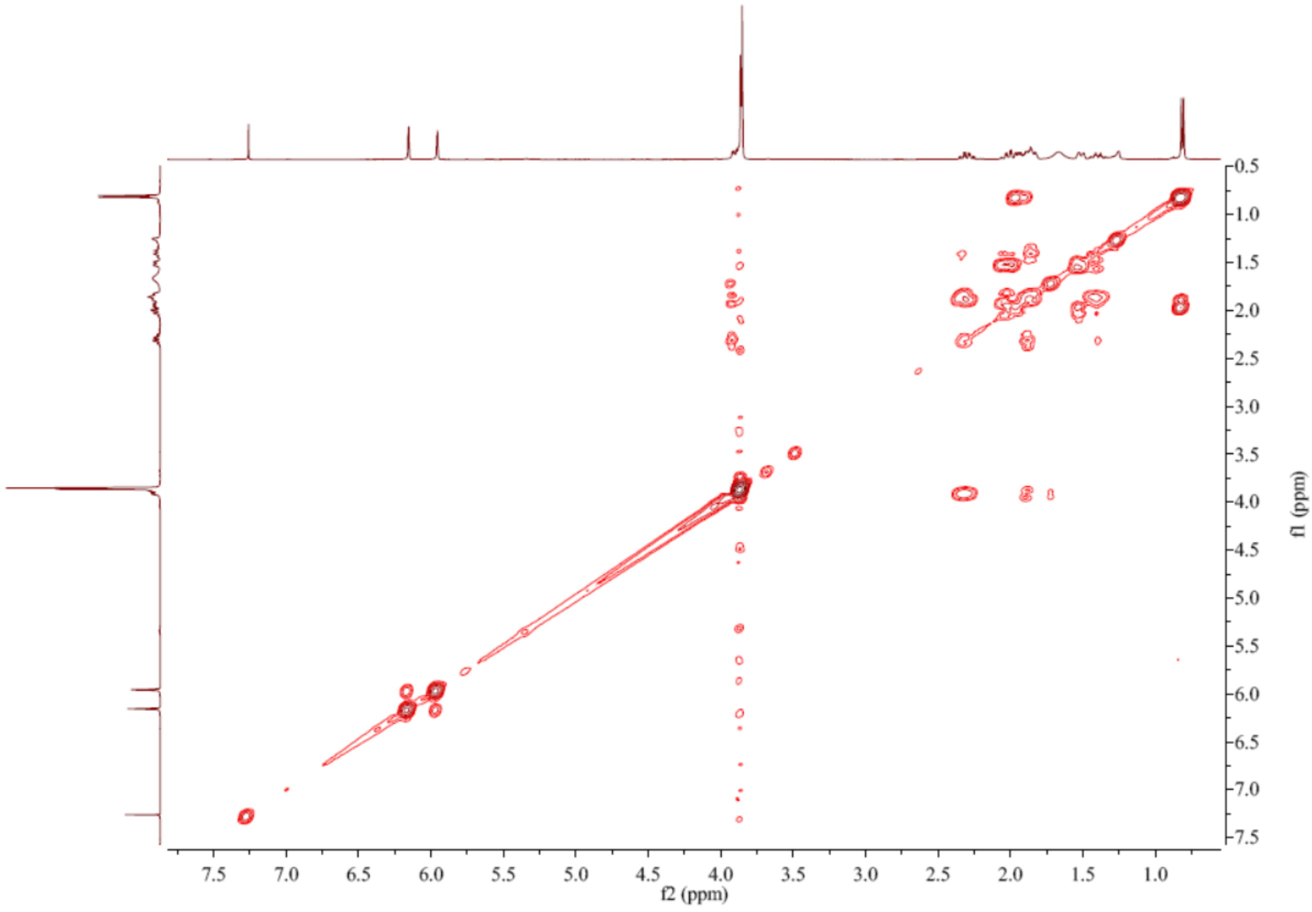

S3.21. HMBC spectrum of **5** in CDCL$_3$

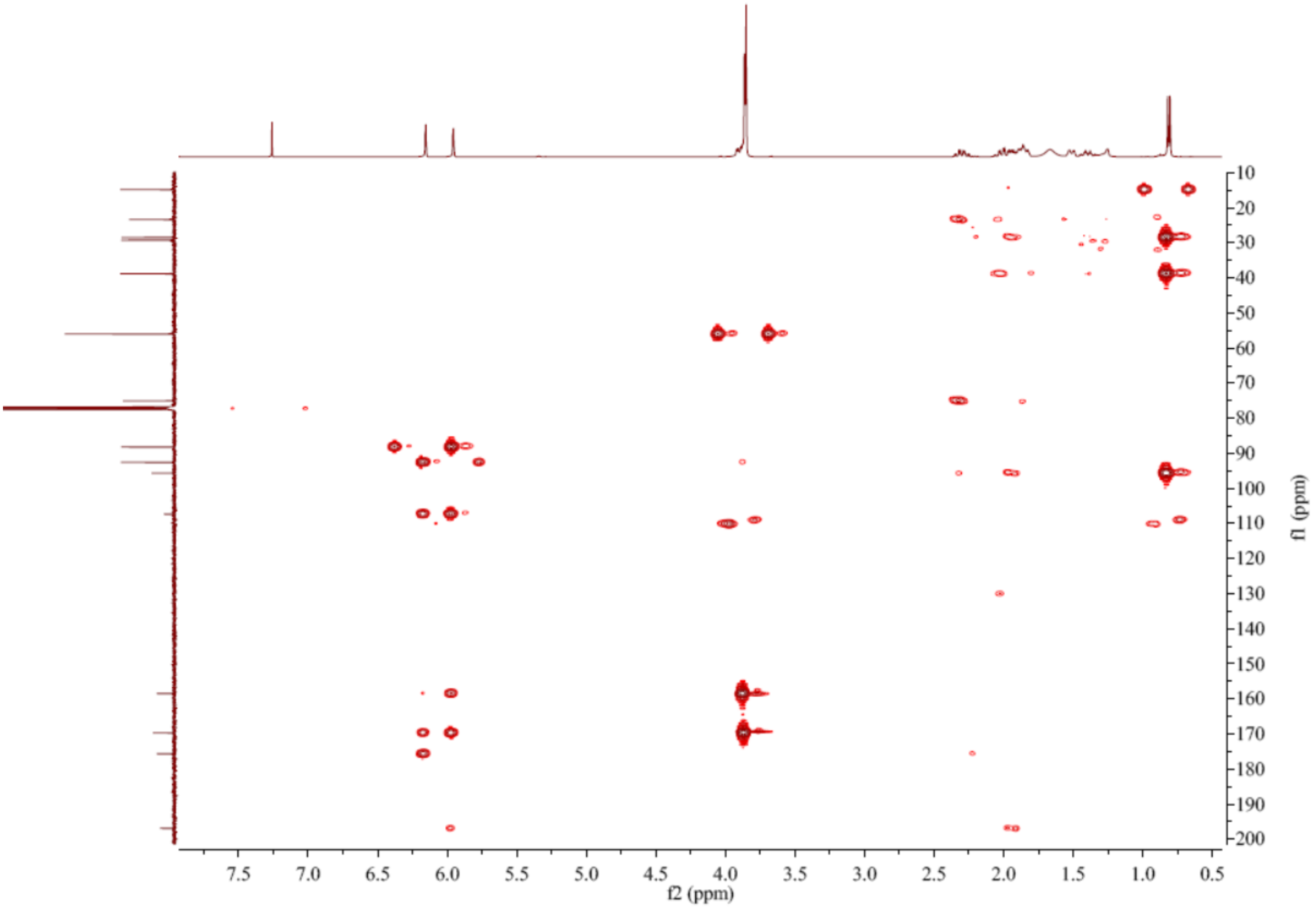

S3.22. NOESY spectrum of **5** in CDCL$_3$

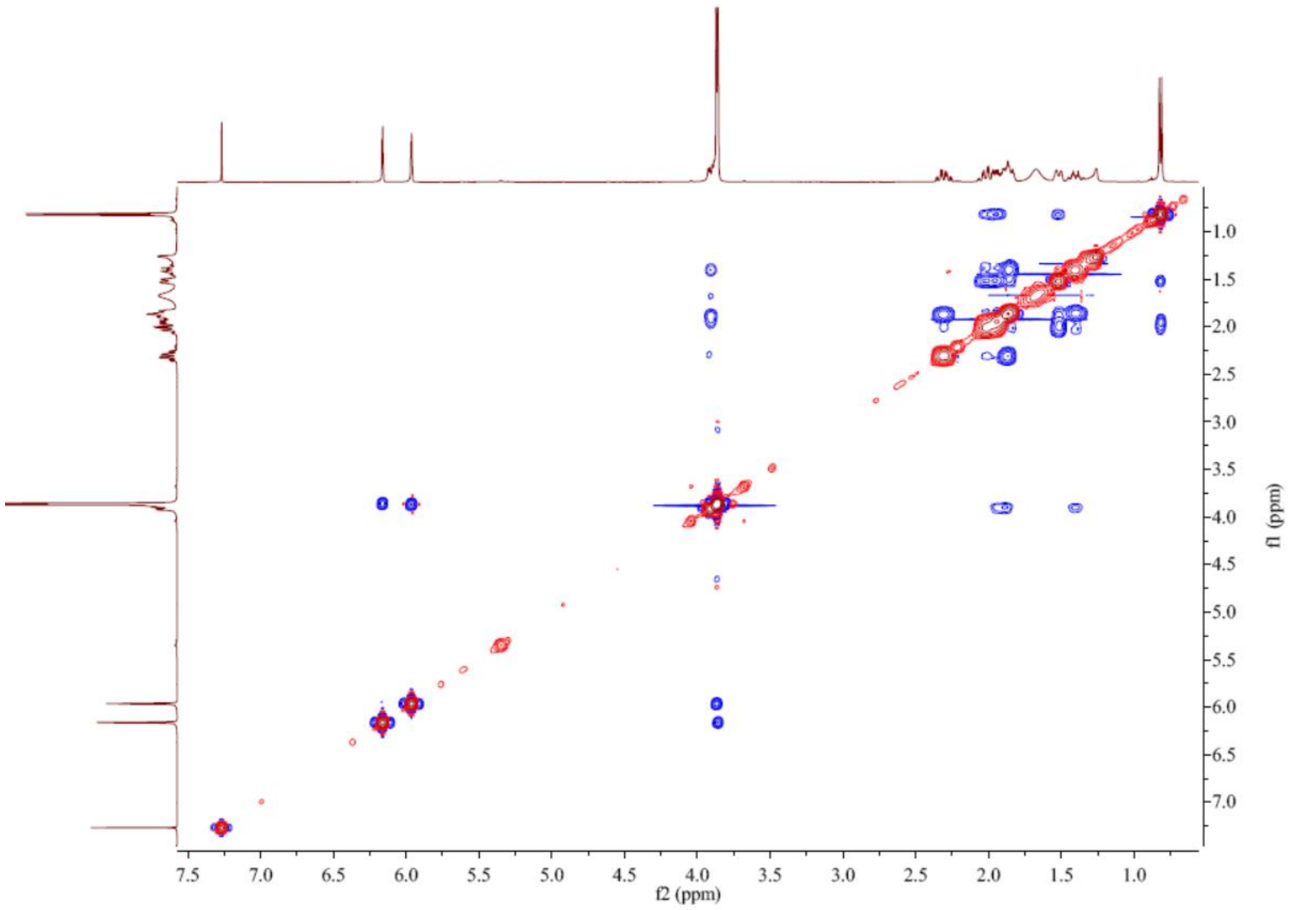

S3.23. ^{1}H NMR spectrum of **6** in $CDCl_3$

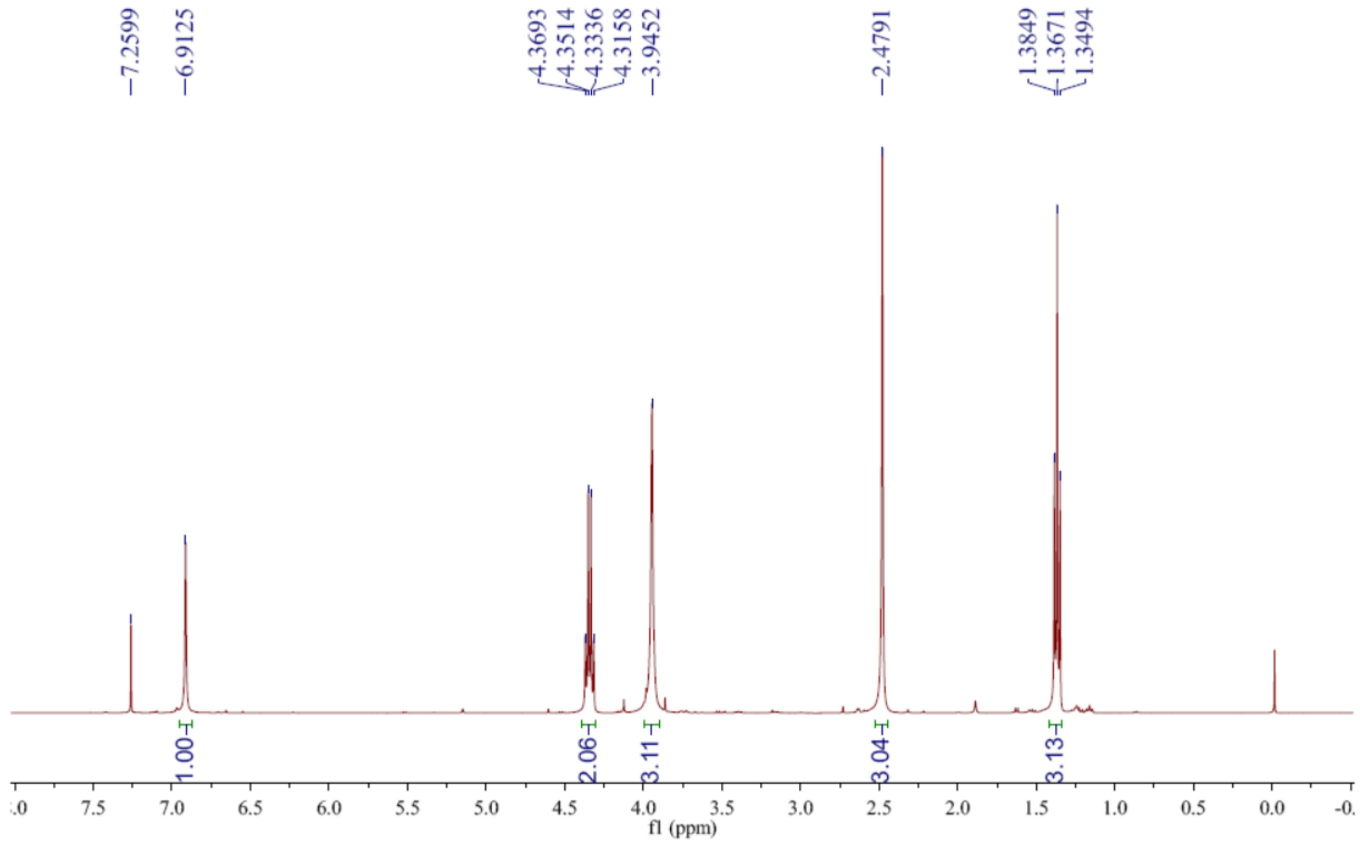

S3.24. ^{13}C NMR and DEPT spectra of **6** in $CDCl_3$

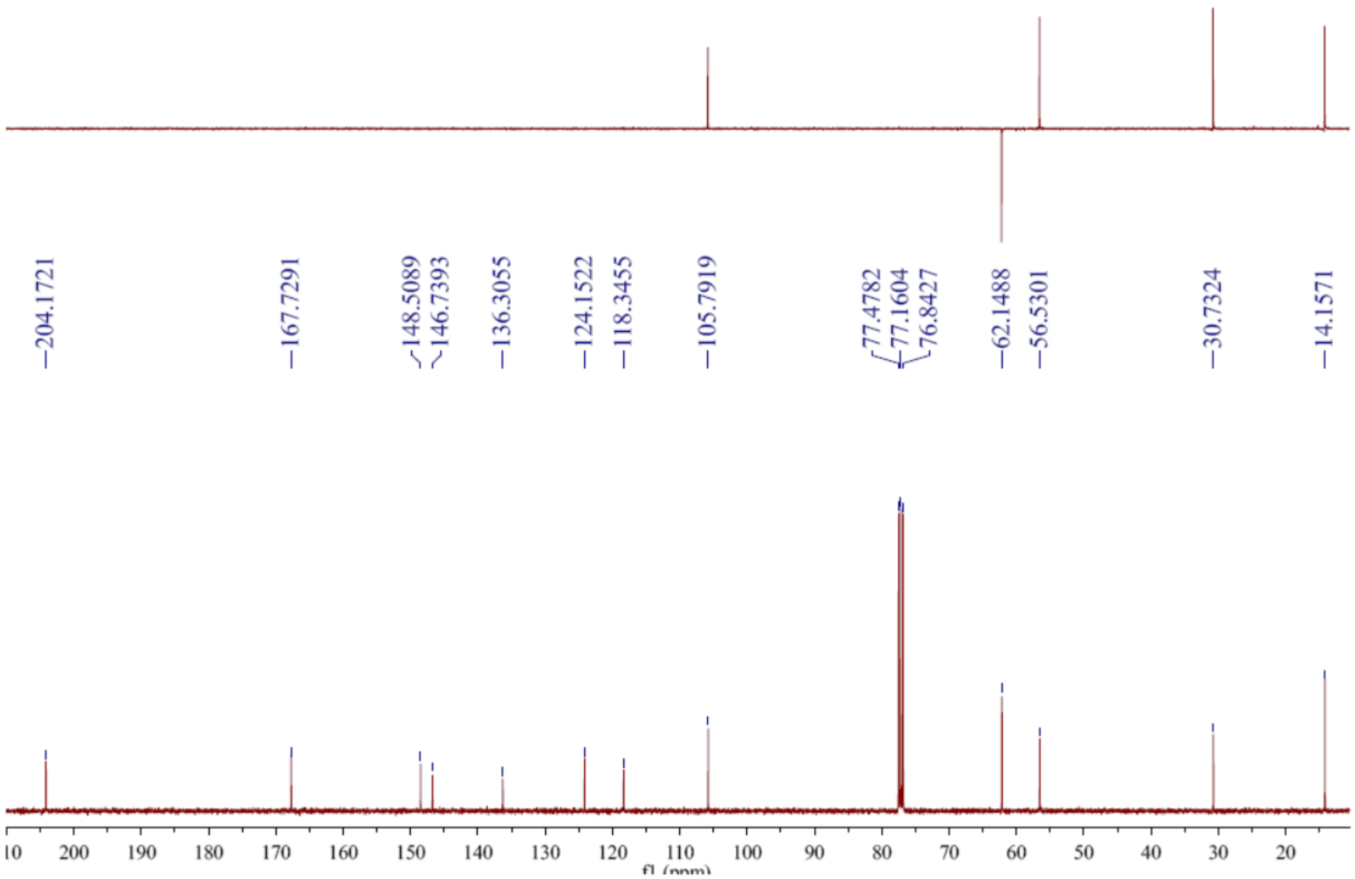

S3.25. HSQC spectrum of **6** in $CDCl_3$

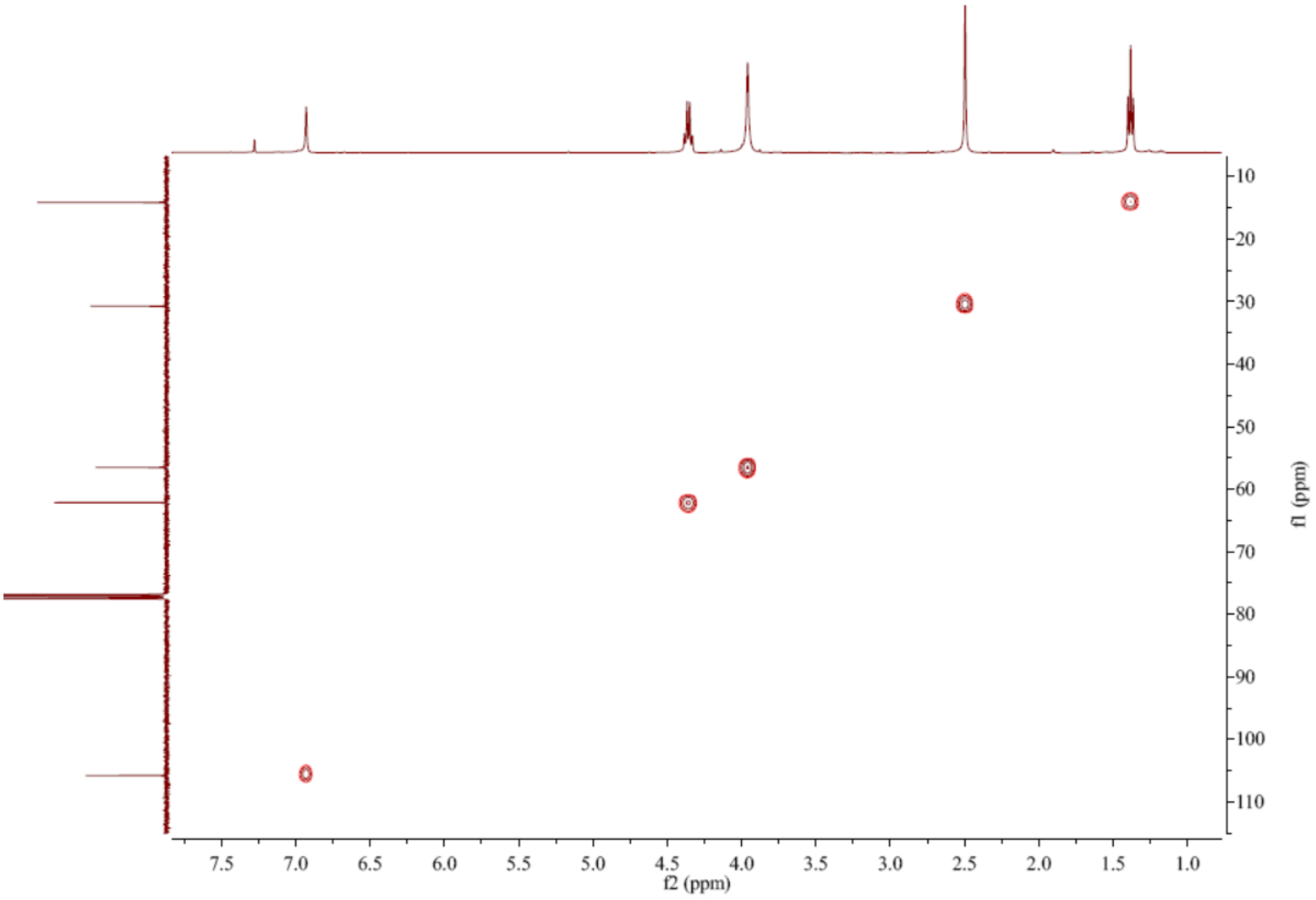

S3.26. $^1H-^1H$ COSY spectrum of **6** in $CDCl_3$

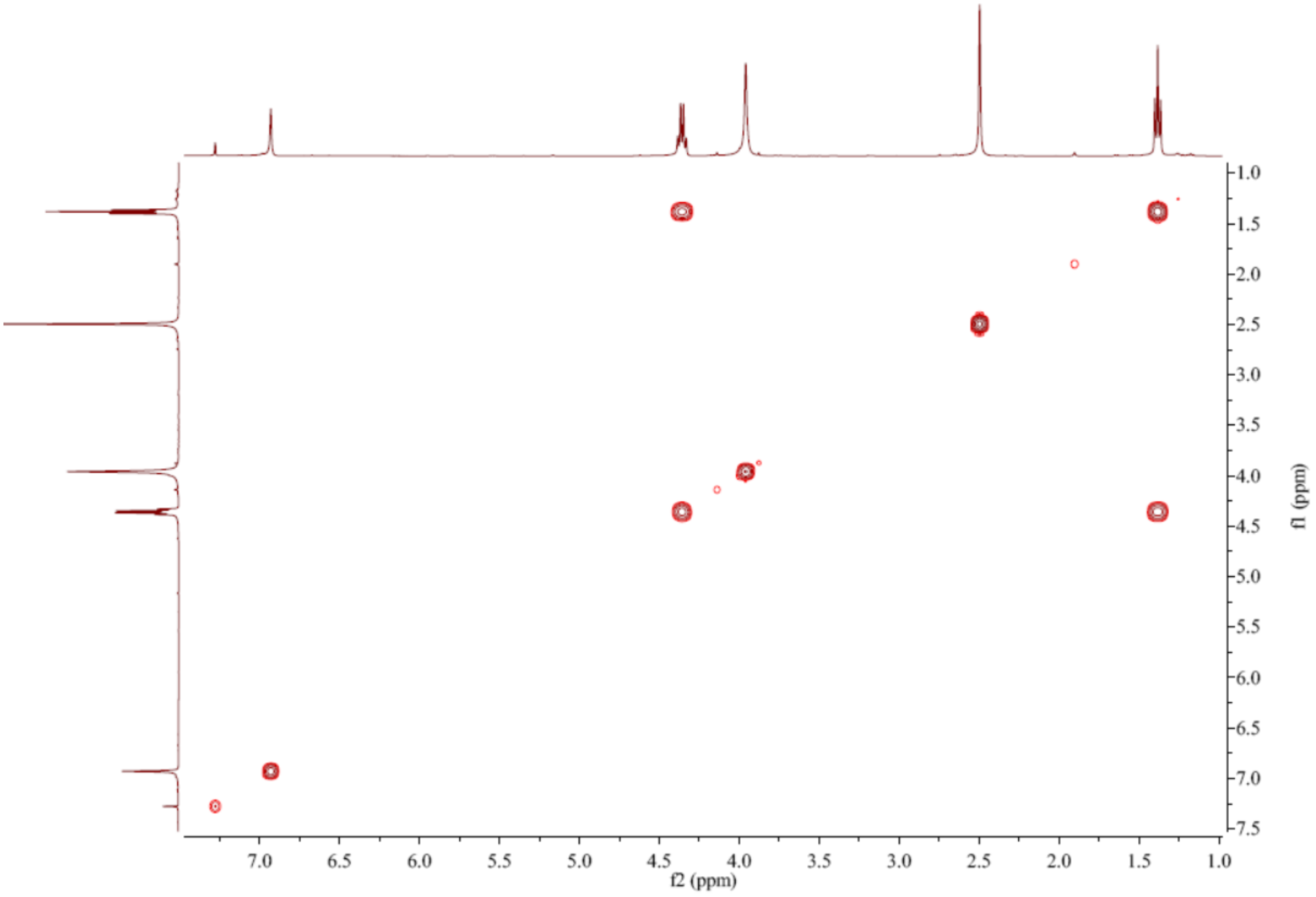

S3.27. HMBC spectrum of **6** in $CDCl_3$

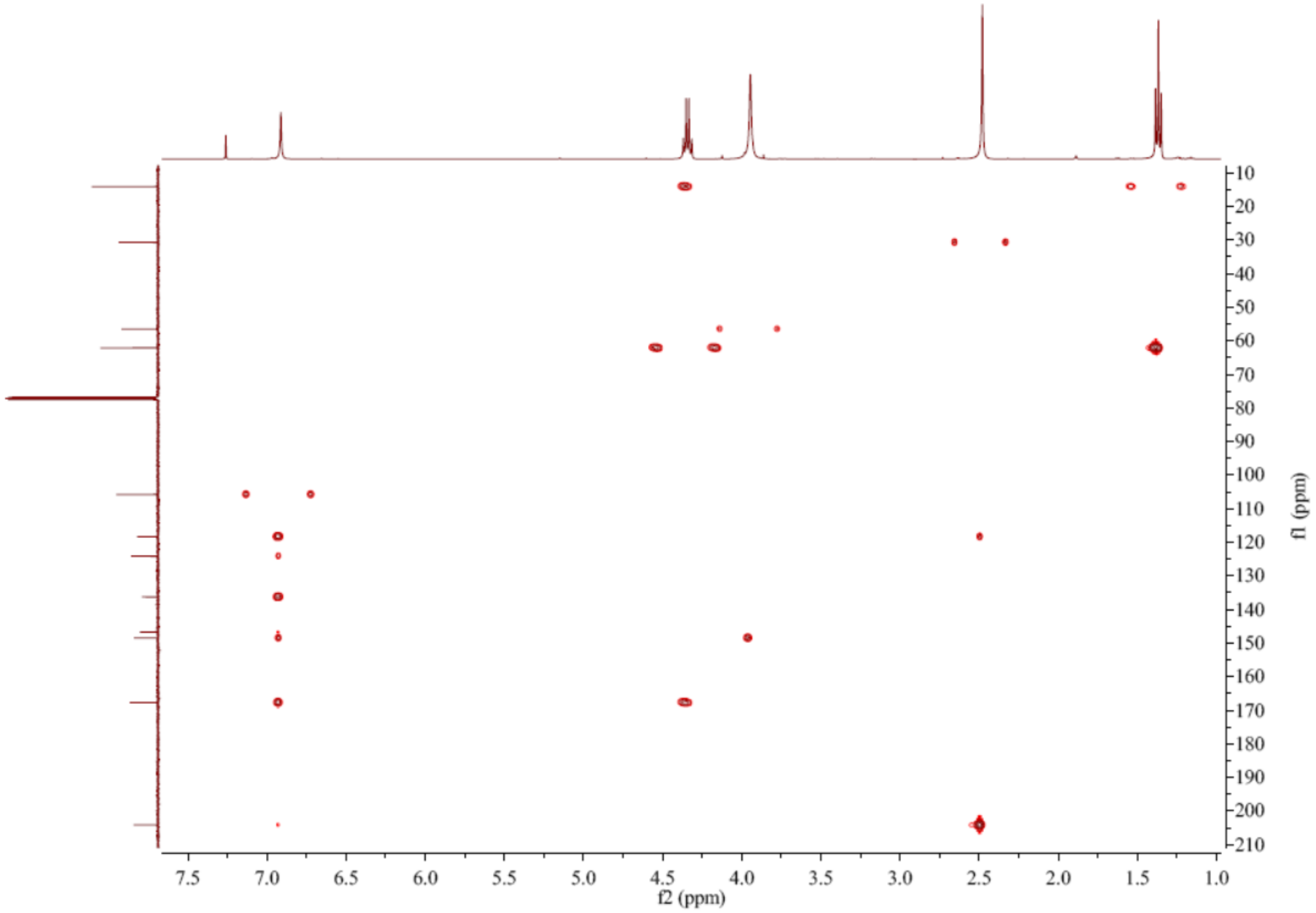

S3.28. ^{1}H NMR spectrum of **7** in CD_3OD

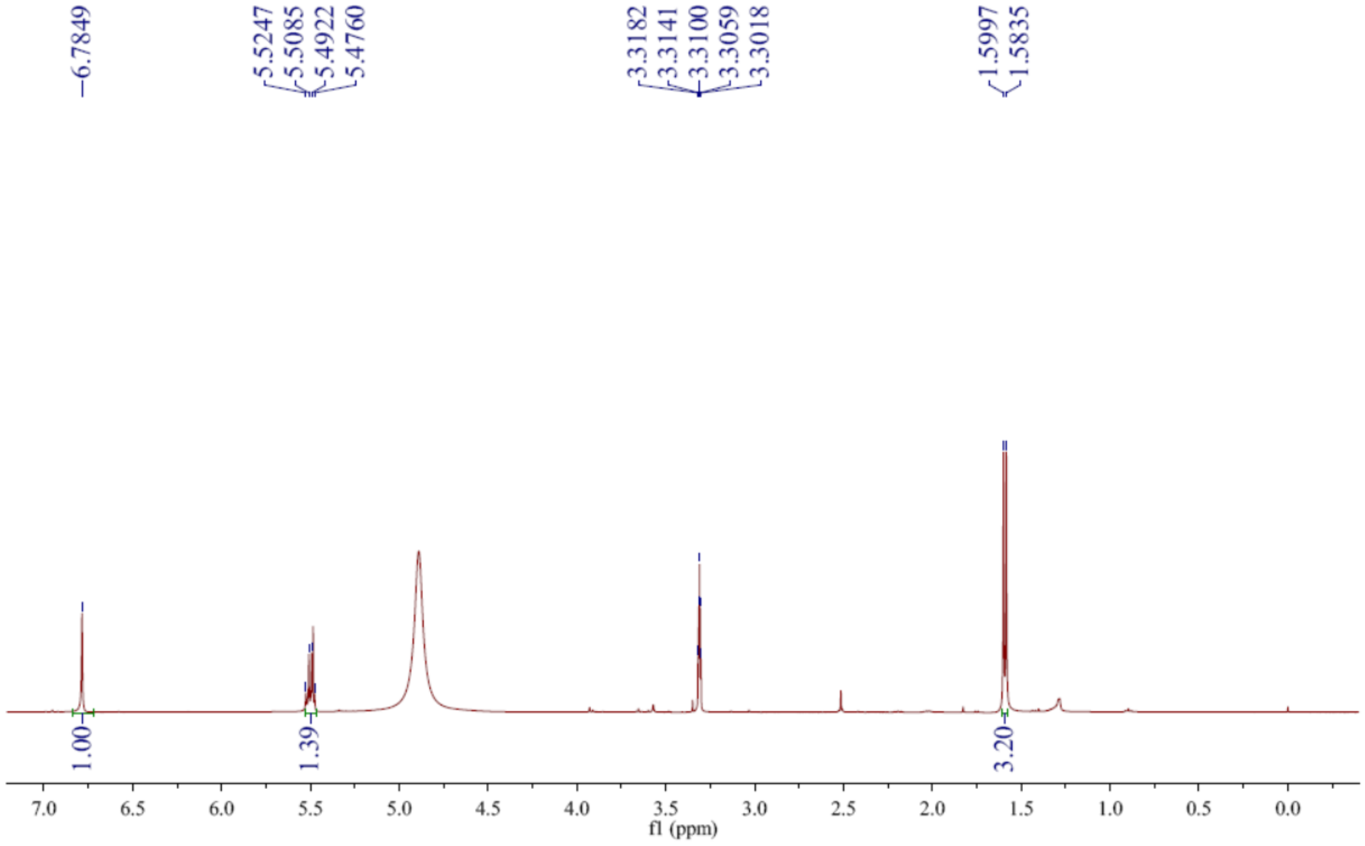

S3.29. ^{13}C NMR and DEPT spectra of **7** in CD_3OD

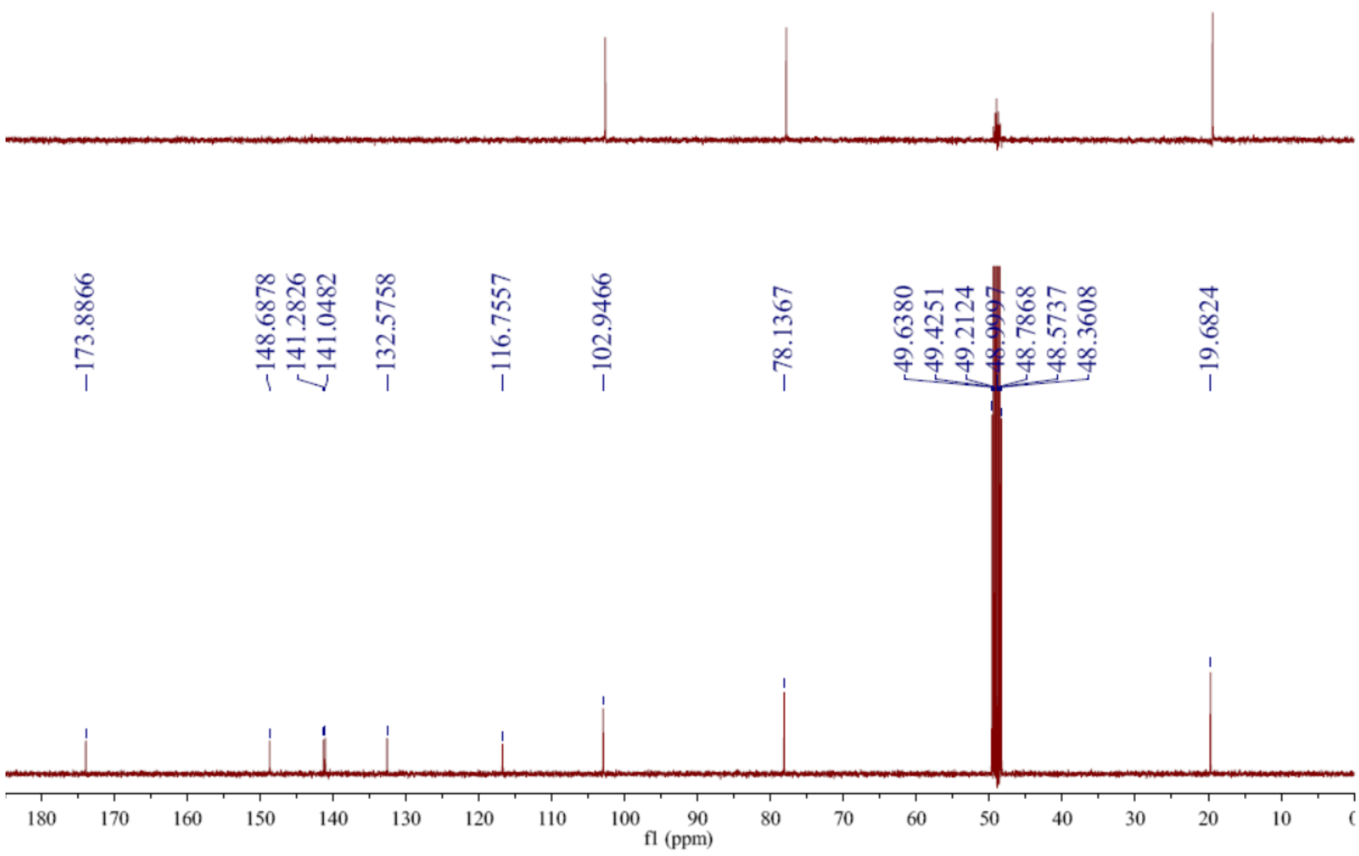

S3.30. ^{1}H NMR spectrum of **8** in CD_3OD

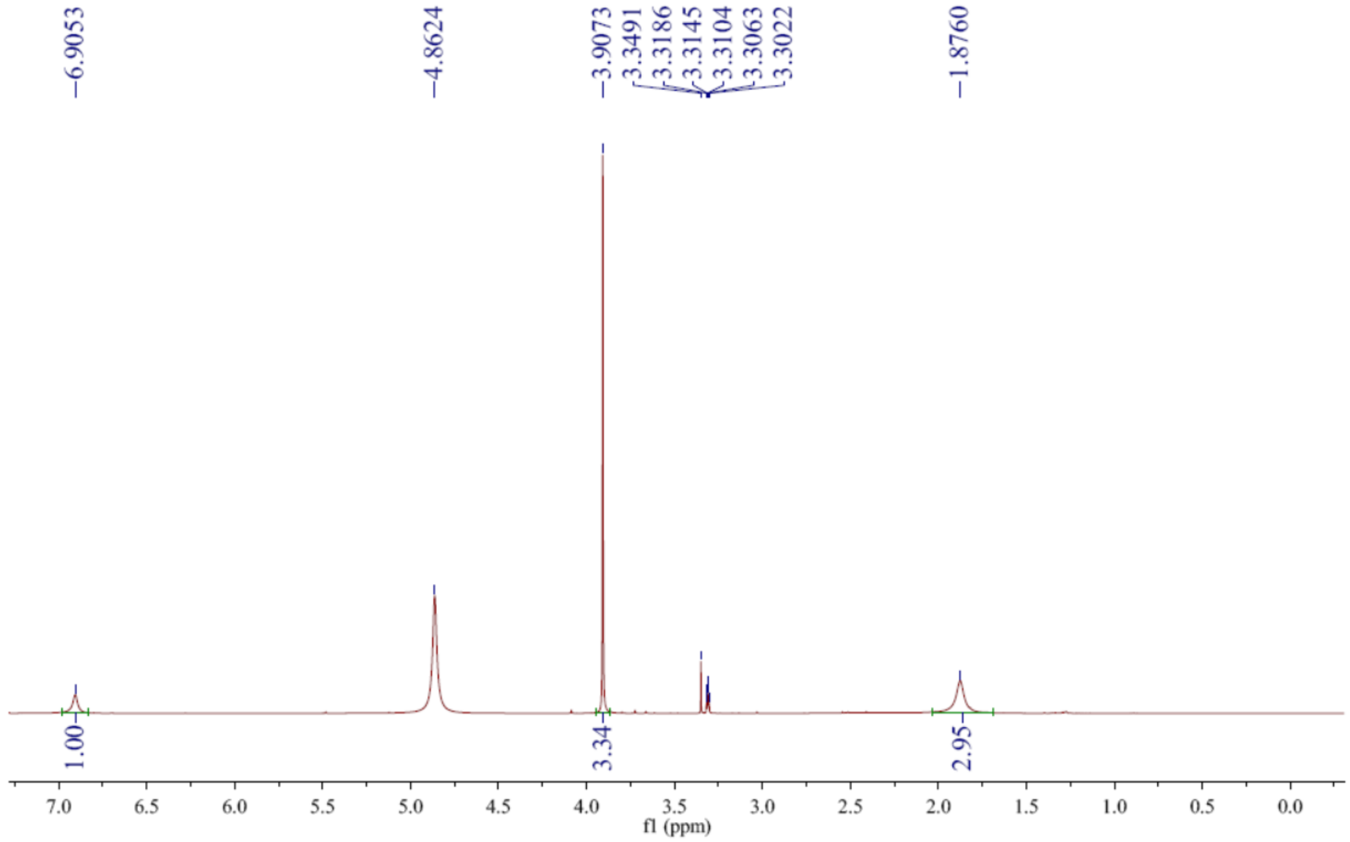

S3.31. ^{13}C NMR and DEPT spectra of **8** in CD_3OD

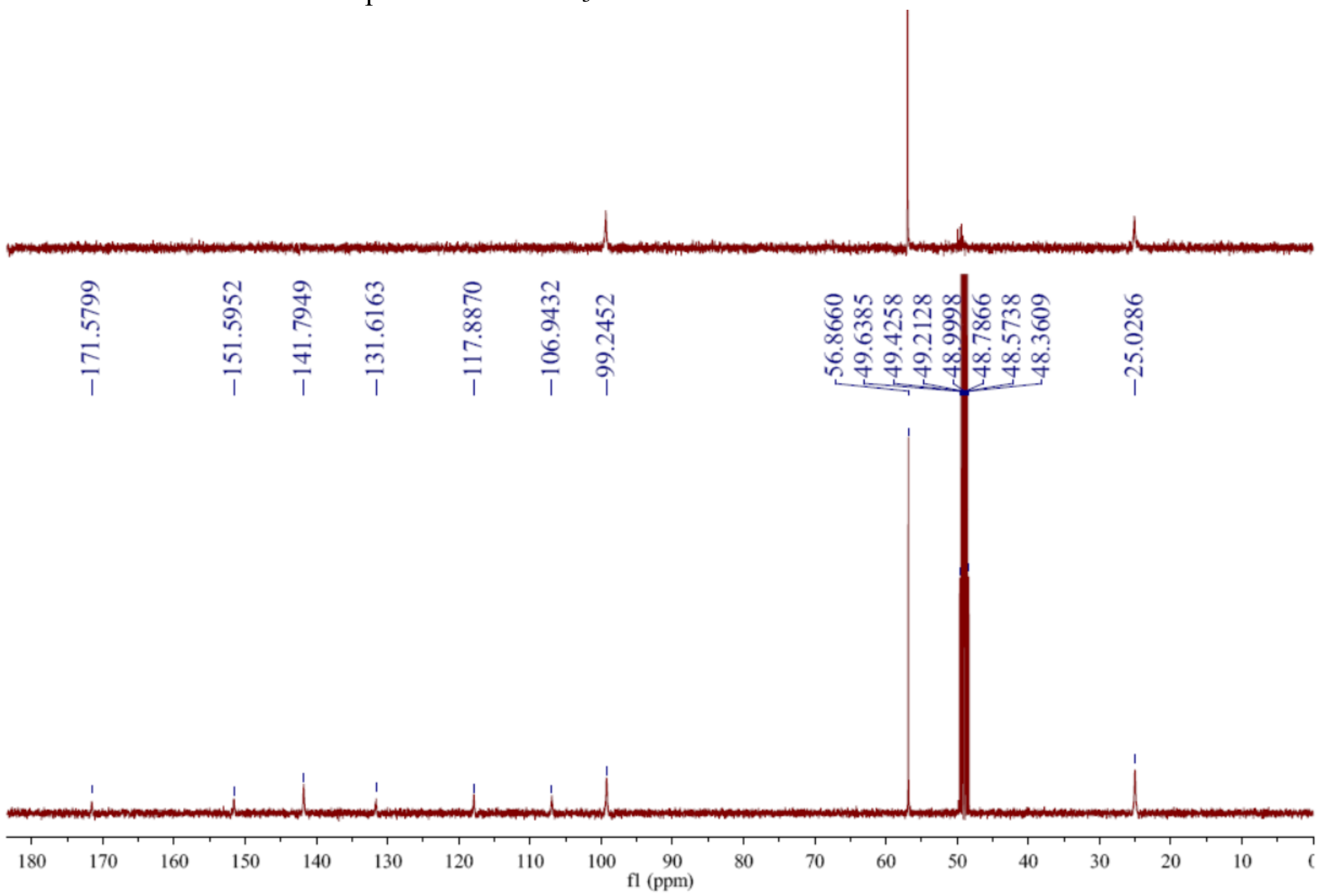

S3.32. ^{1}H NMR spectrum of **9** in CD_3COCD_3

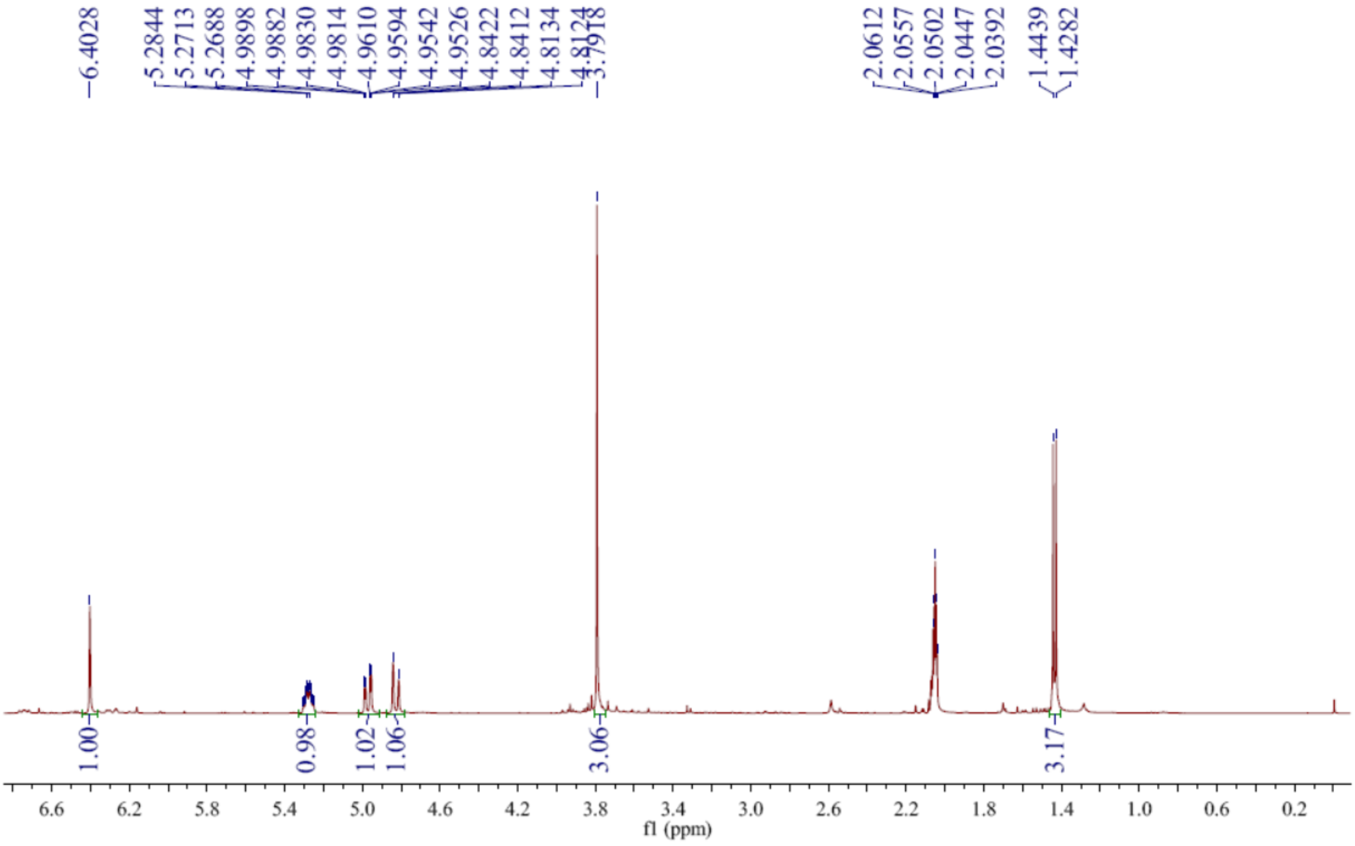

S3.33. ^{13}C NMR and DEPT spectra of **9** in CD_3COCD_3

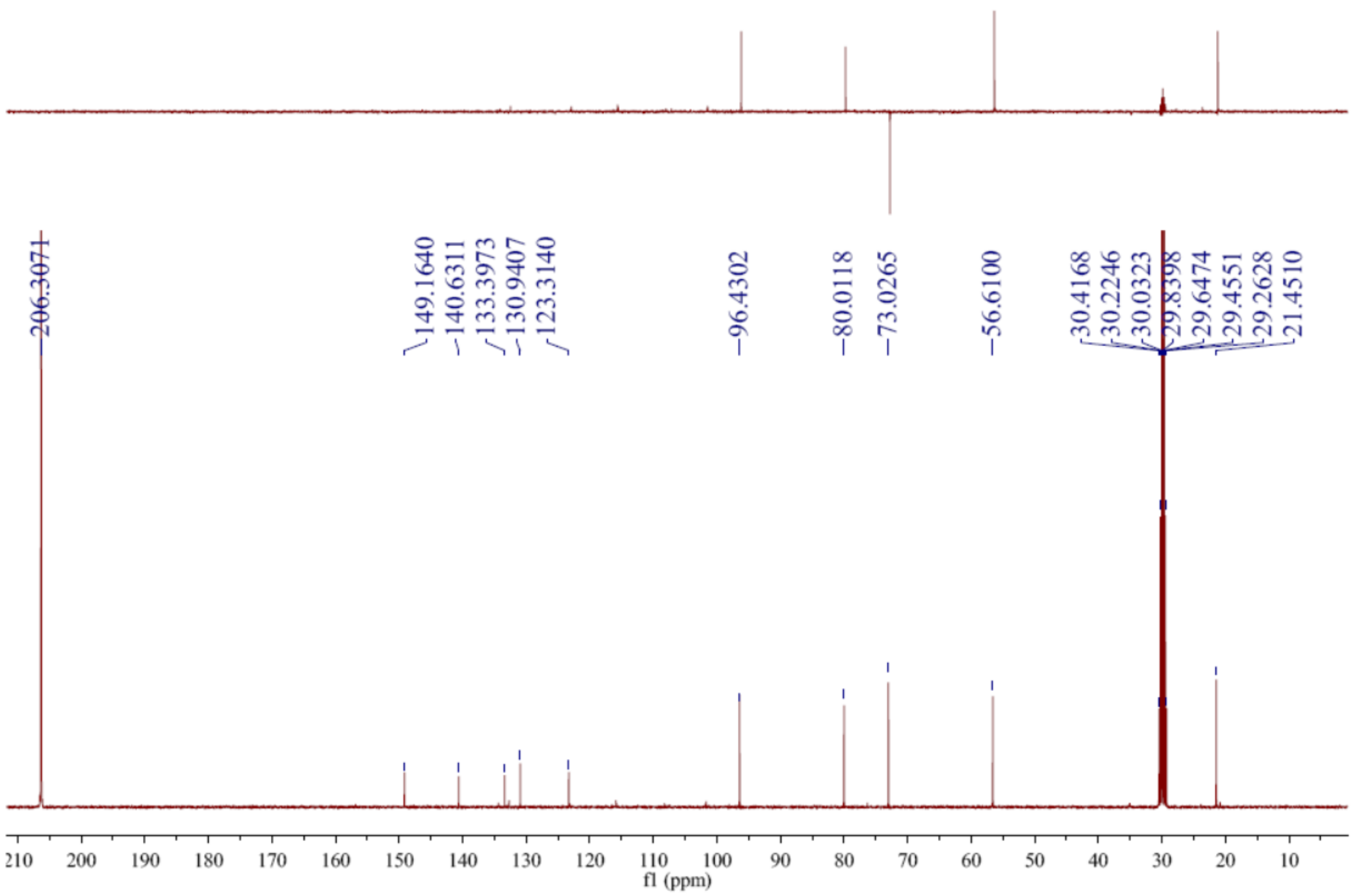

S3.34. ^{1}H NMR spectrum of **10** in $CDCl_3$

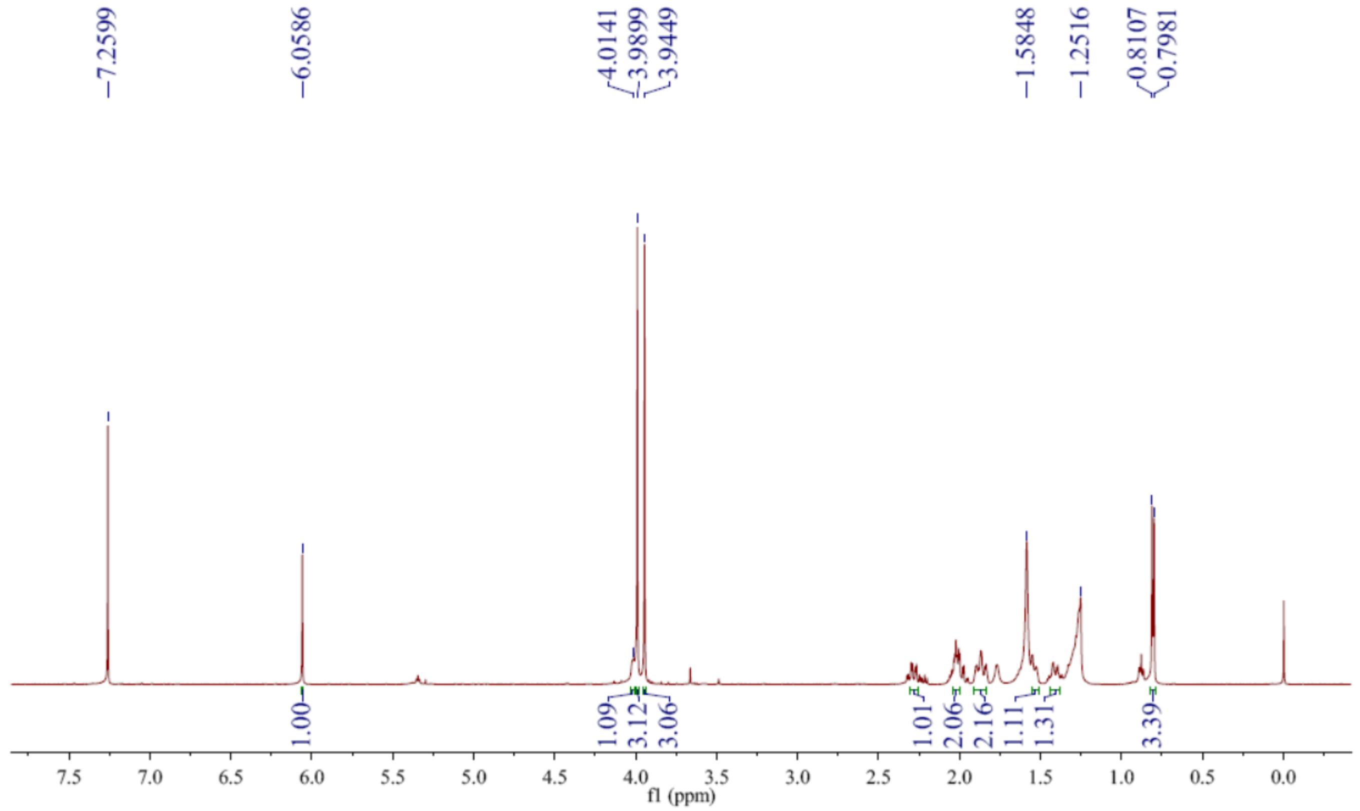

S3.35. ^{13}C NMR and DEPT spectra of **10** in $CDCl_3$

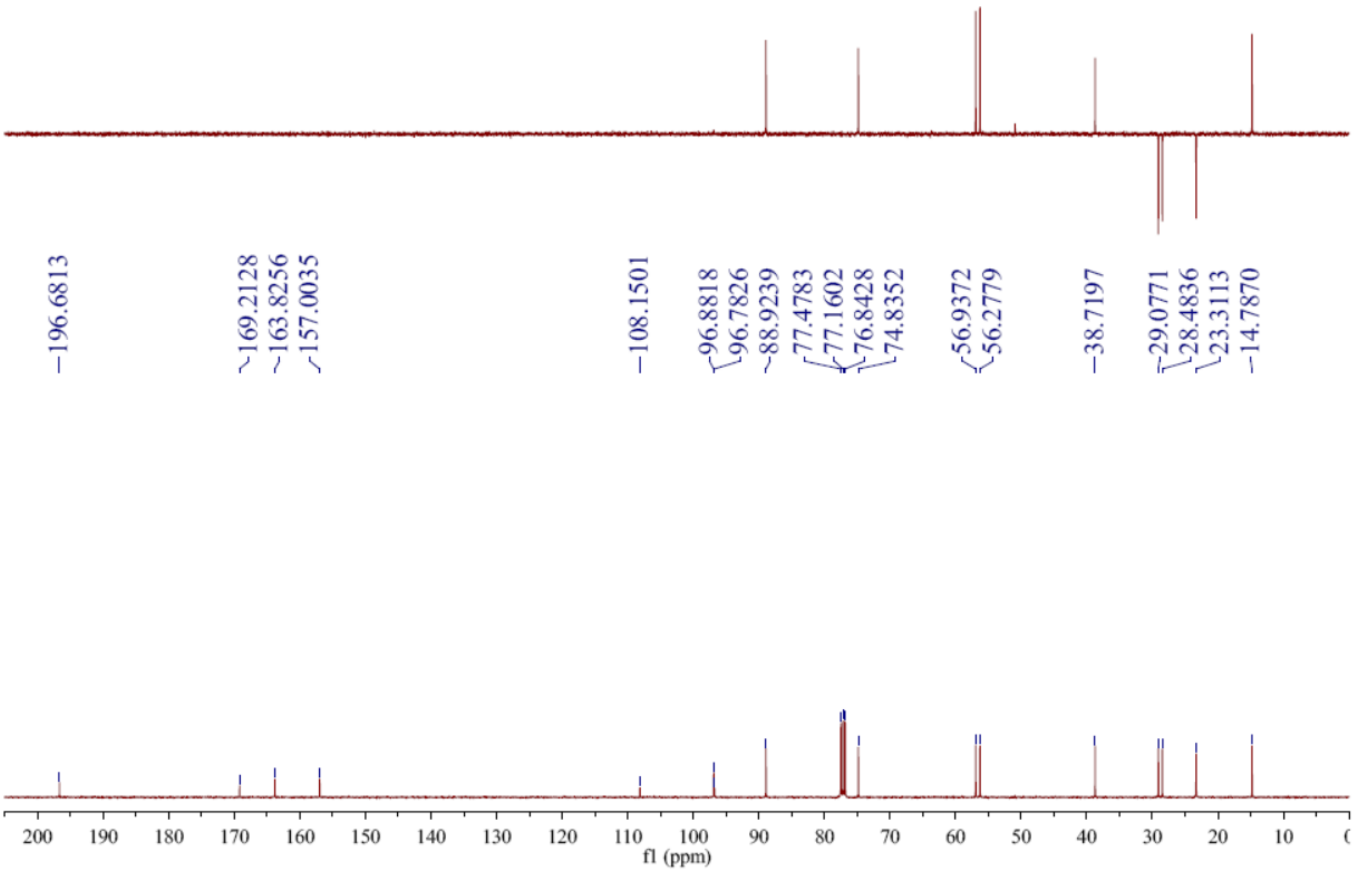

S3.36. HSQC spectrum of **10** in $CDCl_3$

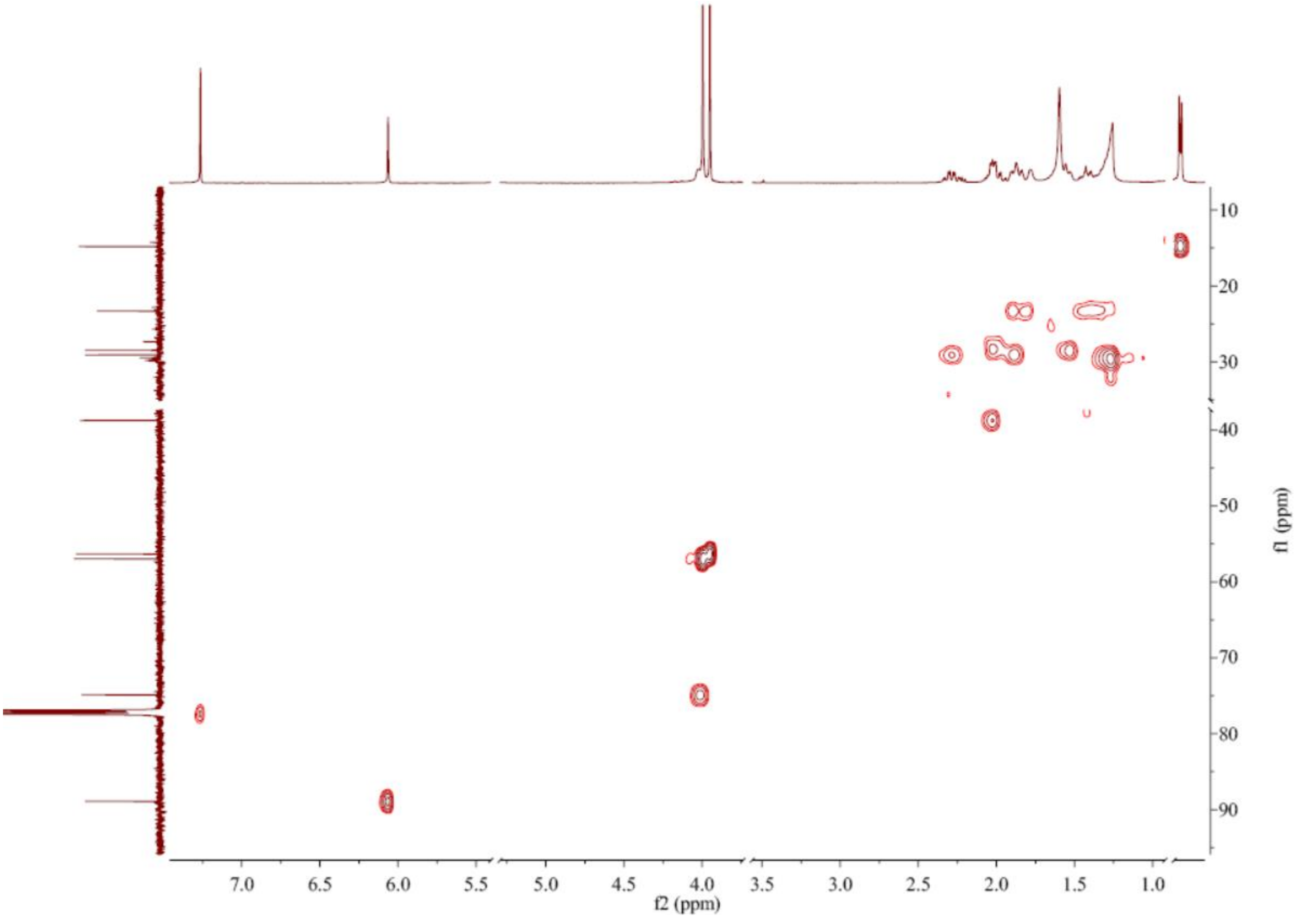

S3.37. $^{1}H-^{1}H$ COSY spectrum of **10** in $CDCl_3$

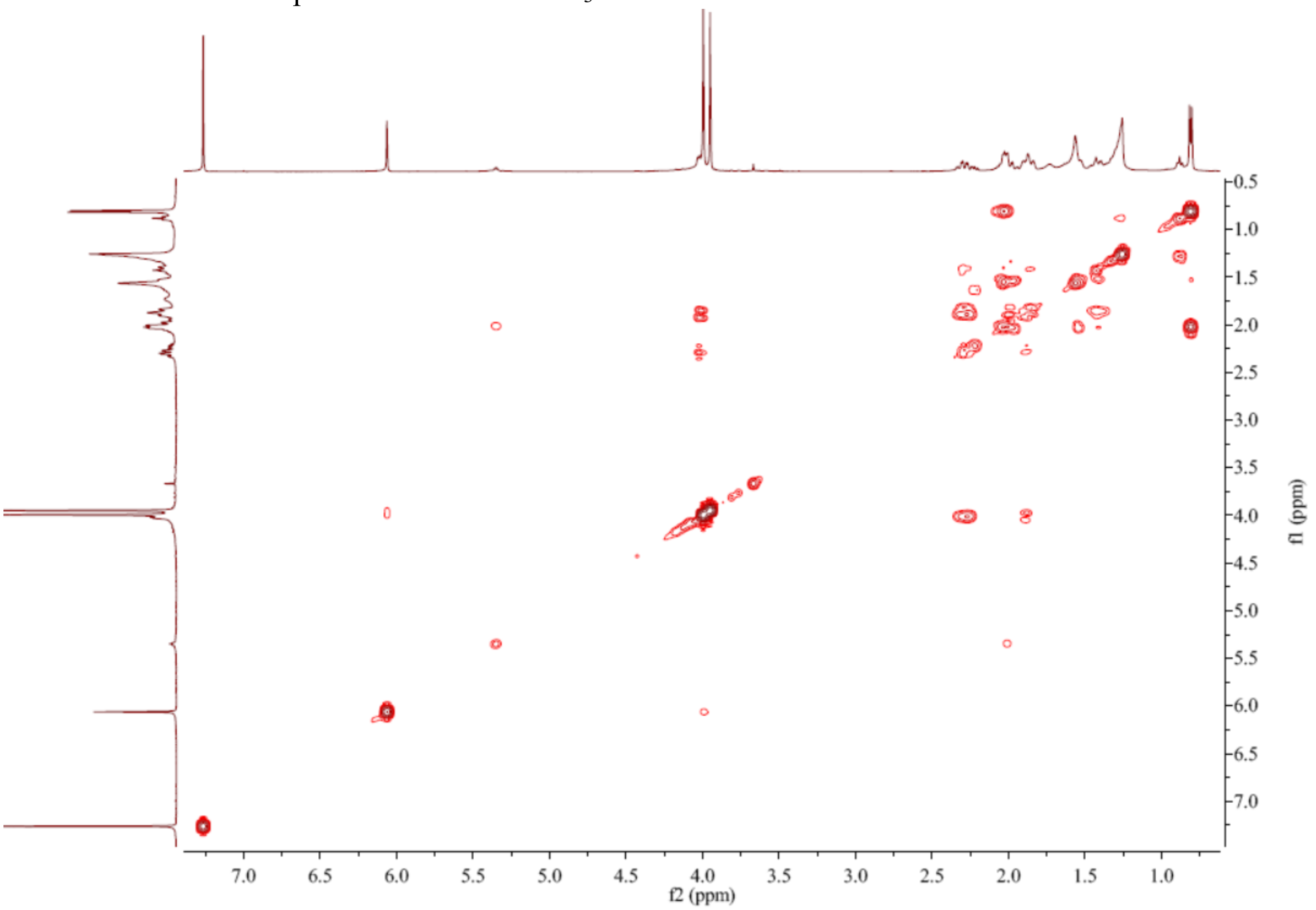

S3.38. HMBC spectrum of **10** in $CDCl_3$

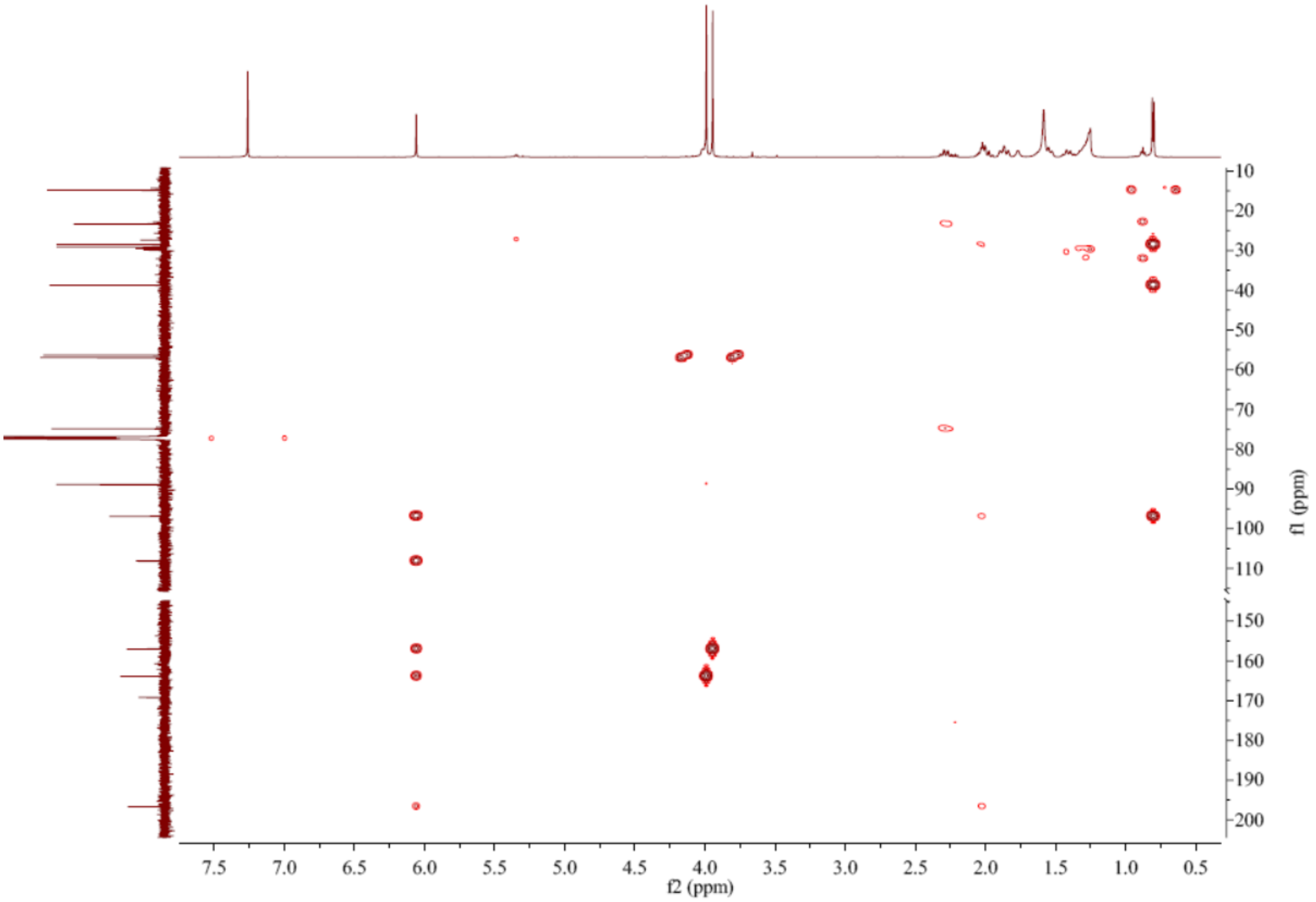

S3.39. ^{1}H NMR spectrum of **11** in $CDCl_3$

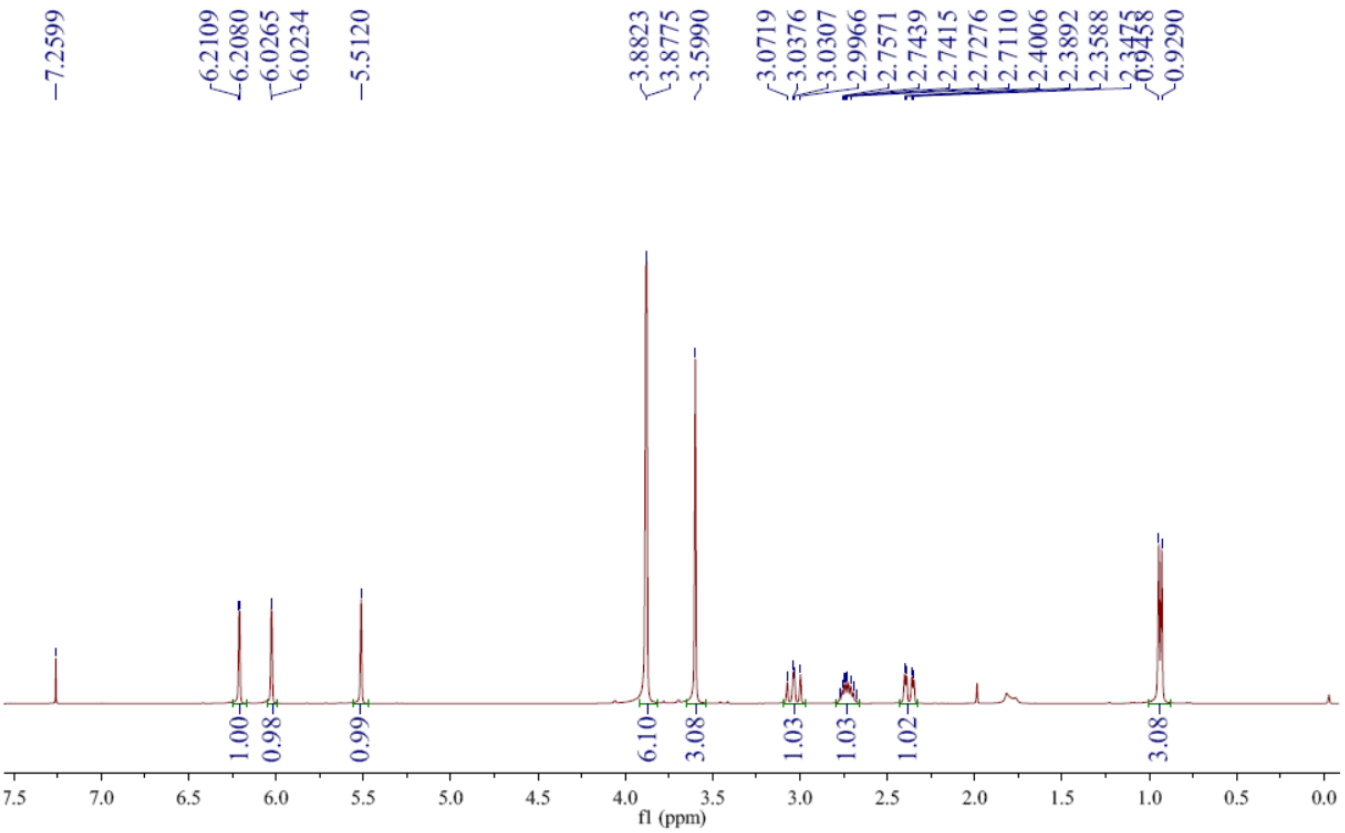

S3.40. ^{13}C NMR and DEPT spectra of **11** in $CDCl_3$

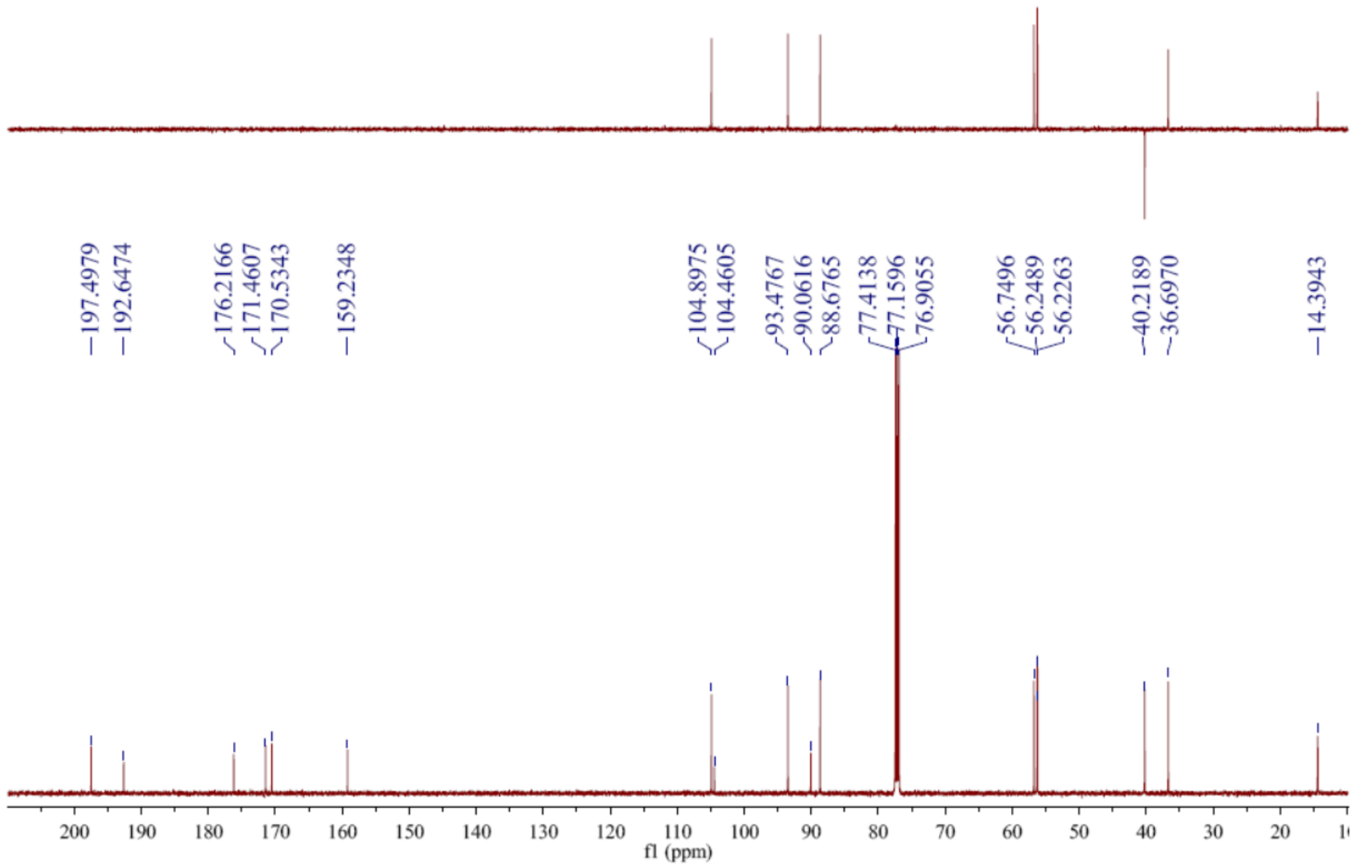

S3.41. ^{1}H NMR spectrum of **12** in $CDCl_3$

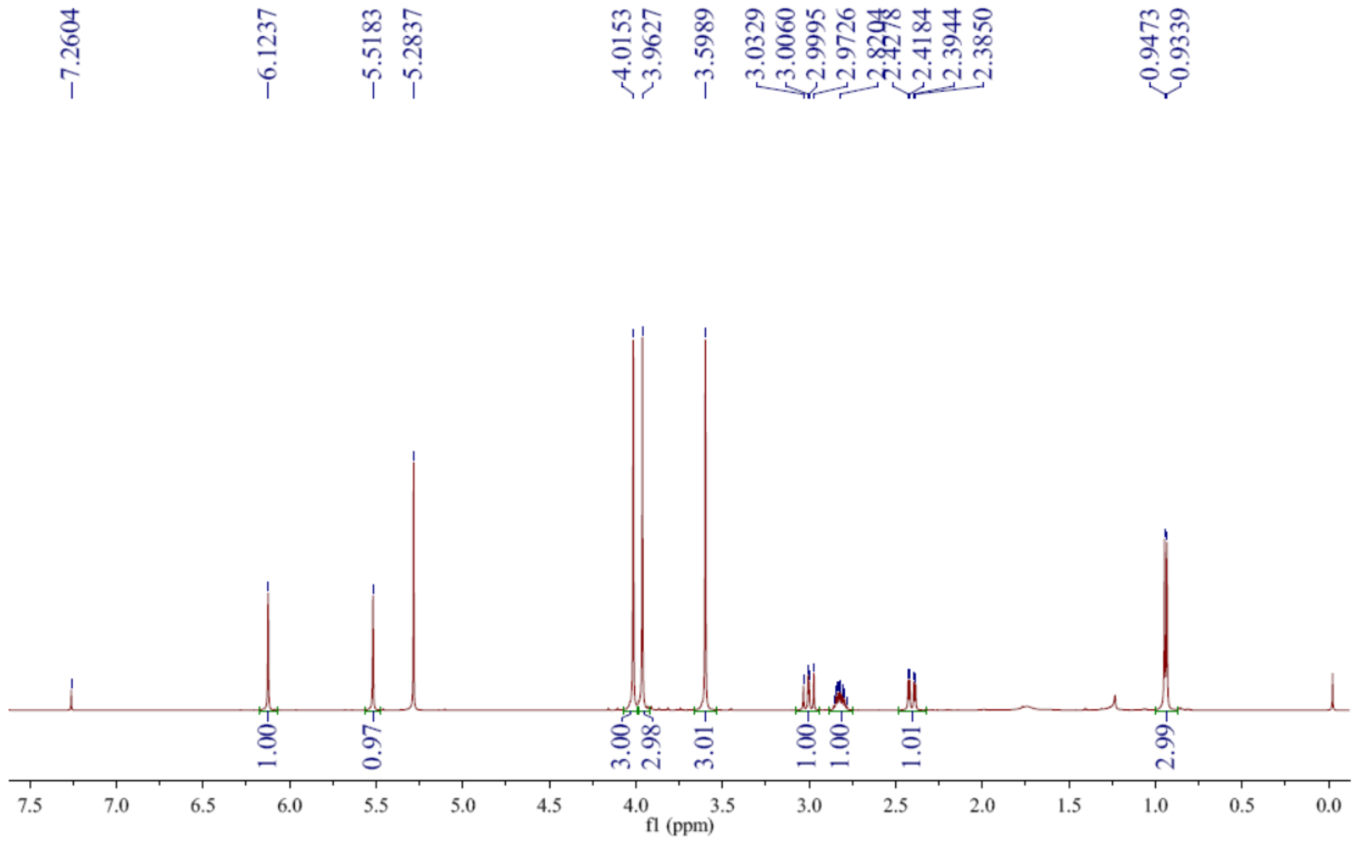

S3.42. ^{13}C NMR spectrum of **12** in $CDCl_3$

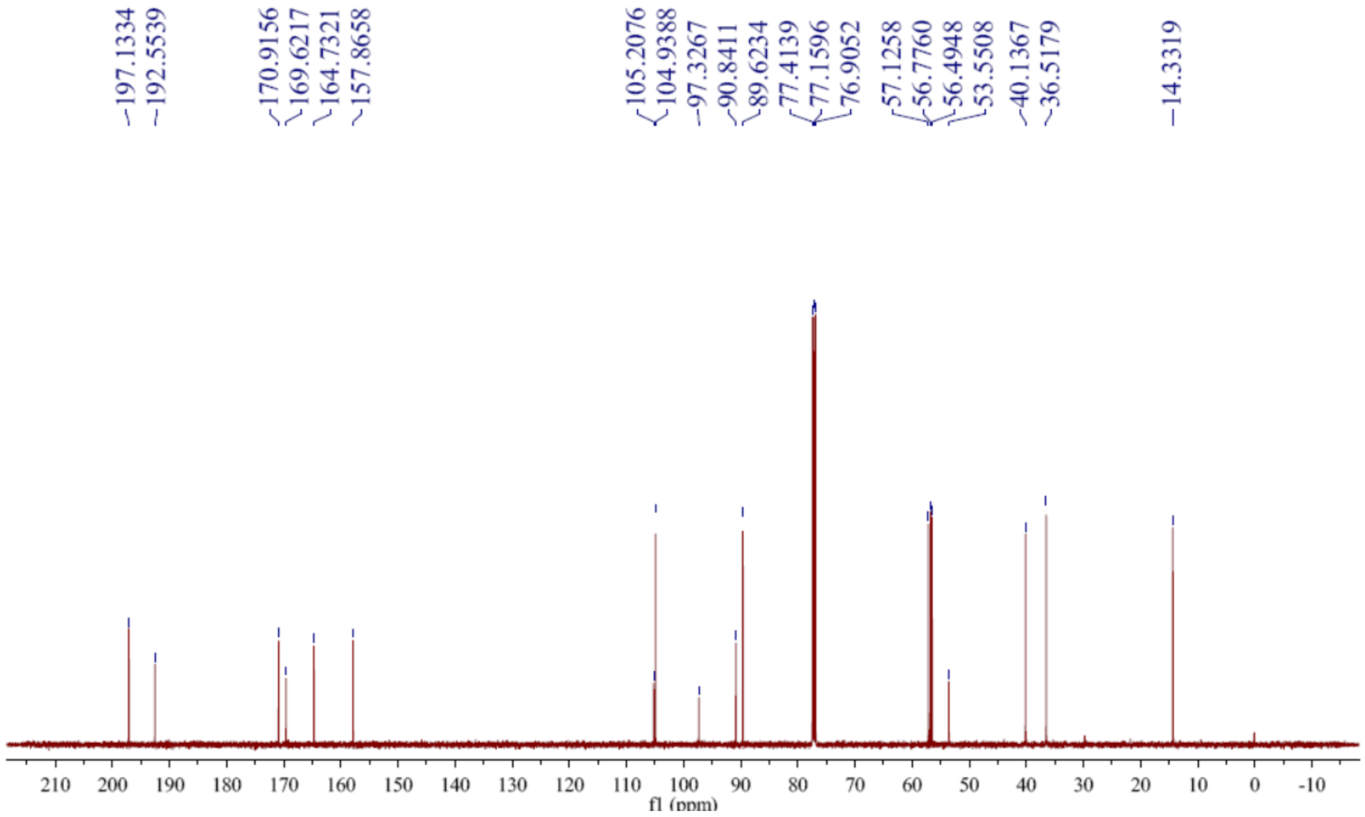

S3.43. ^{1}H NMR spectrum of **12** in CD_3OD

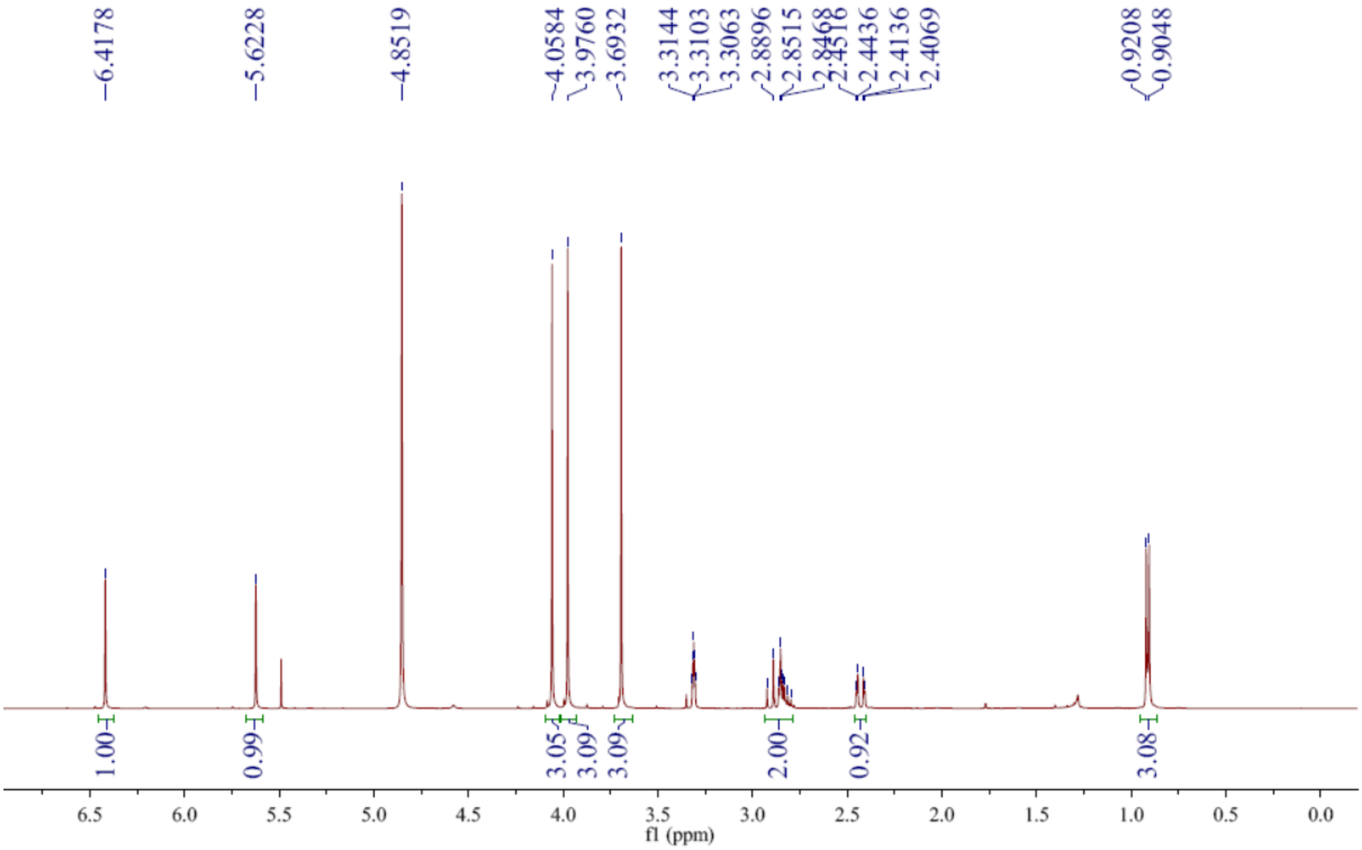

S3.44. ^{13}C NMR and DEPT spectra of **12** in CD_3OD

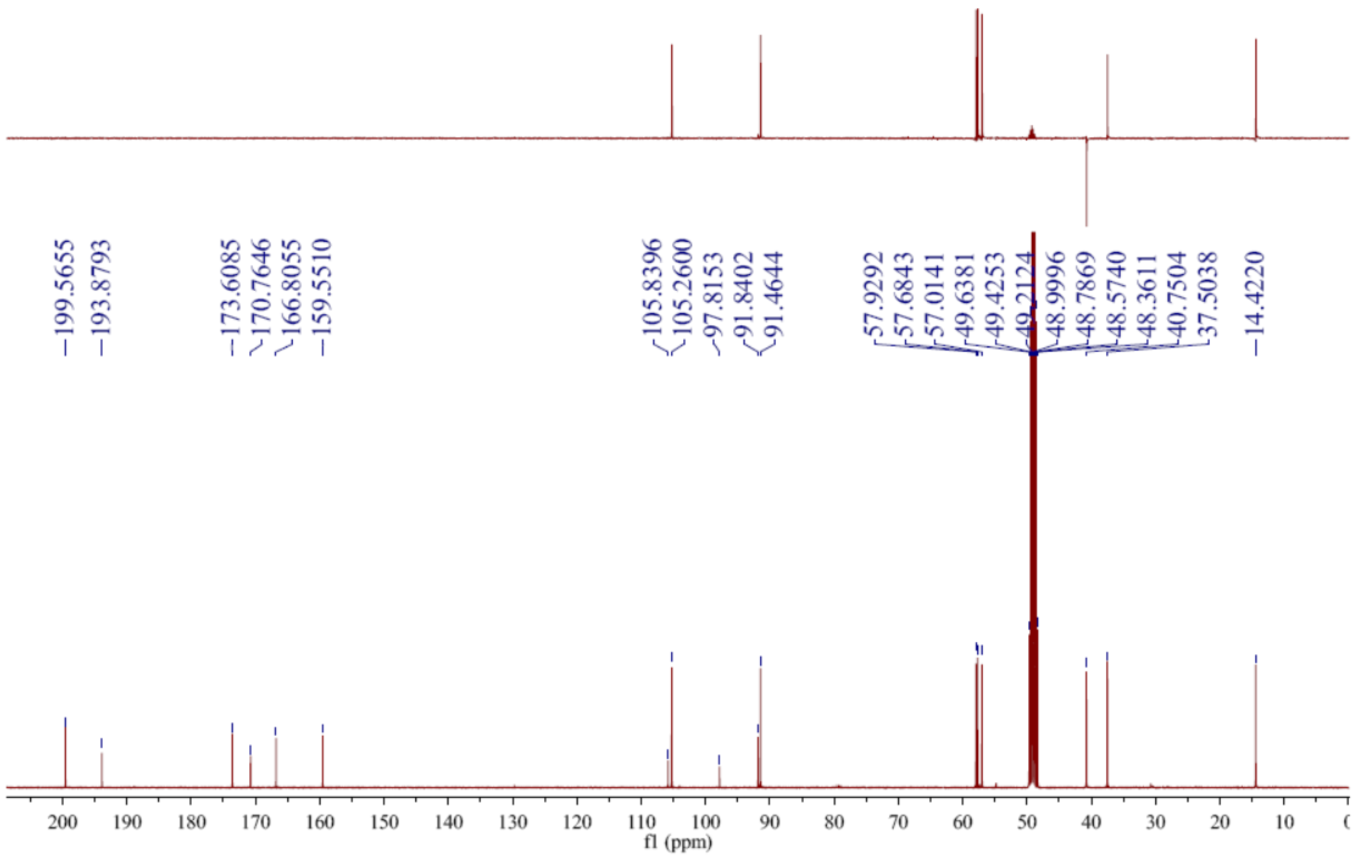

S3.45. ^{1}H NMR spectrum of **13** in $CDCl_3$

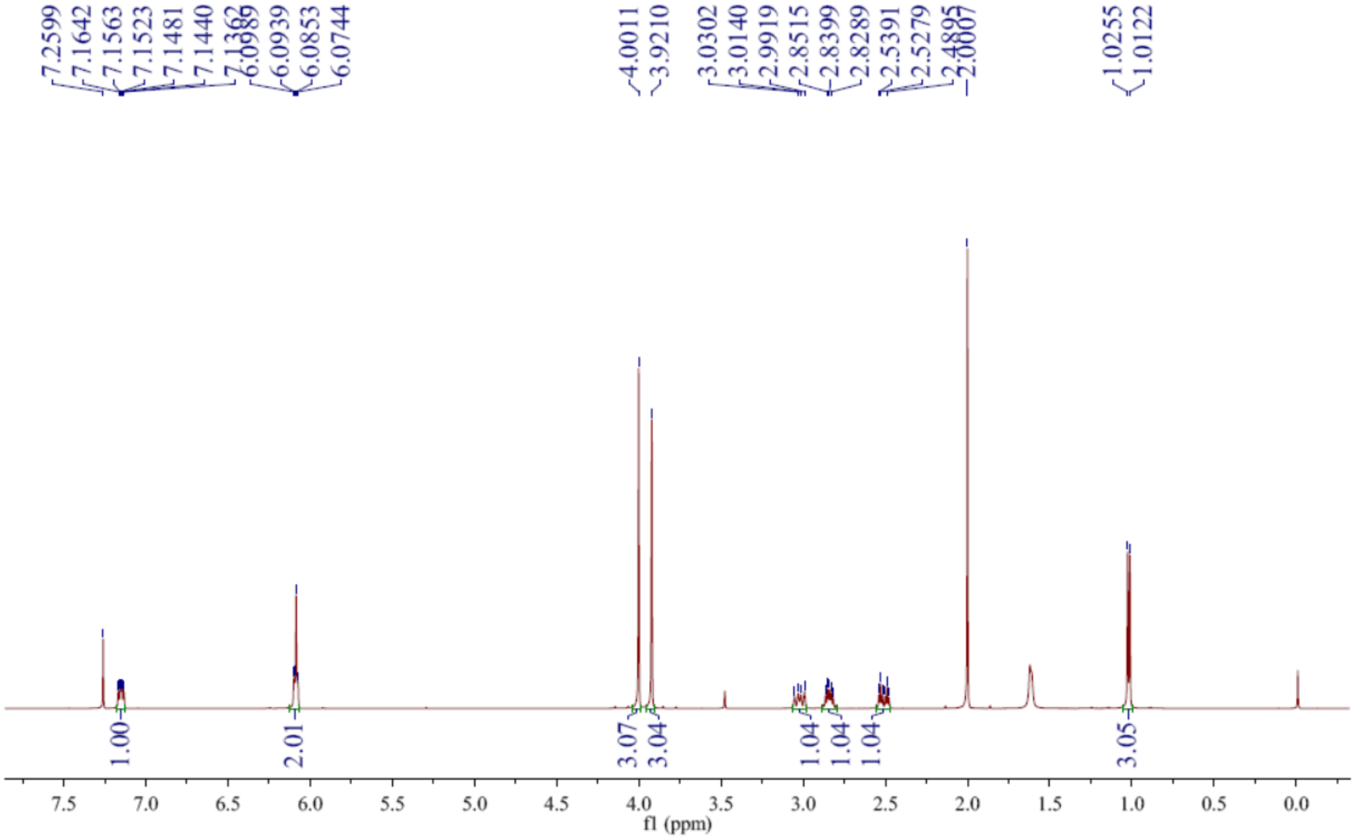

S3.46. ^{13}C NMR and DEPT spectra of **13** in $CDCl_3$

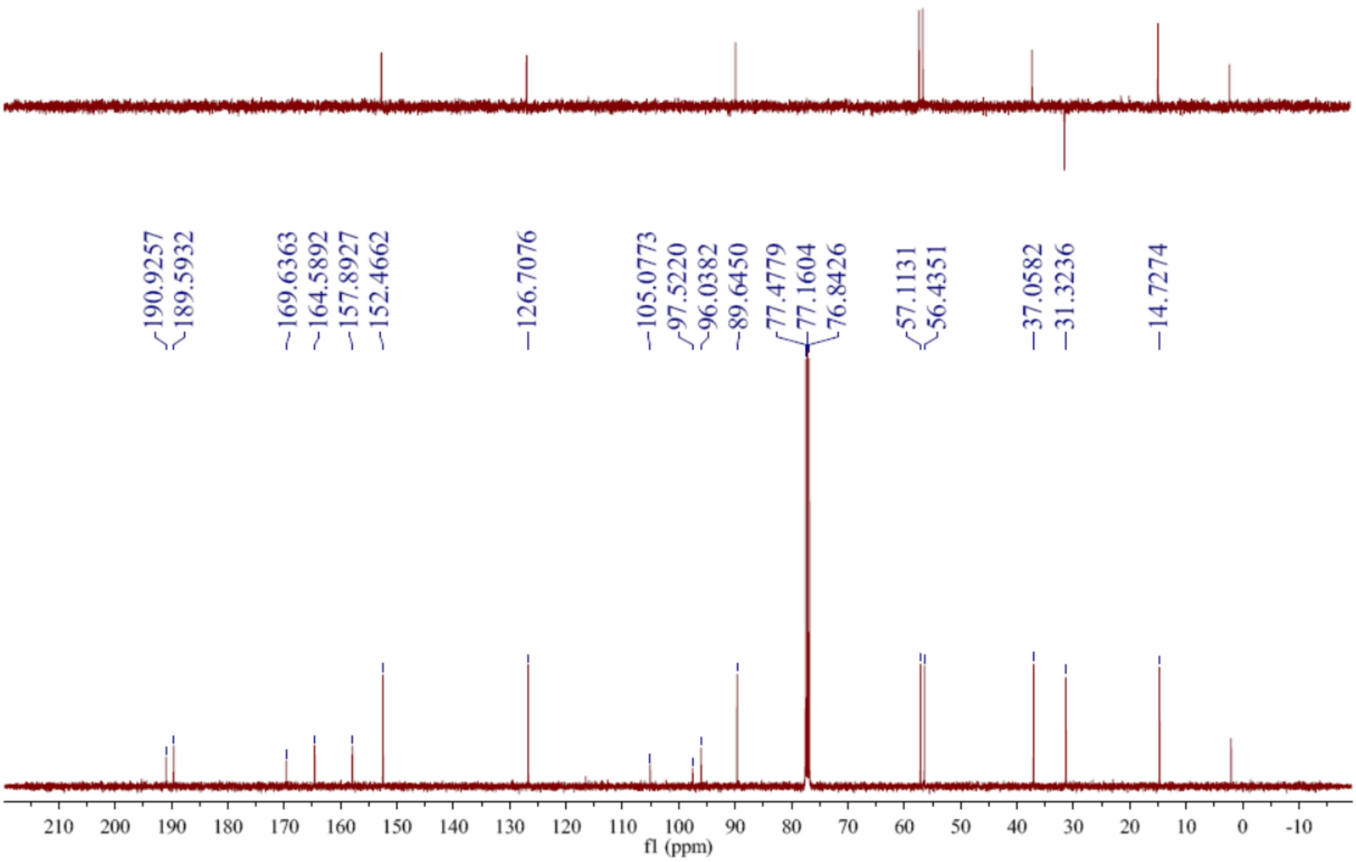

S3.47. HSQC spectrum of **13** in $CDCl_3$

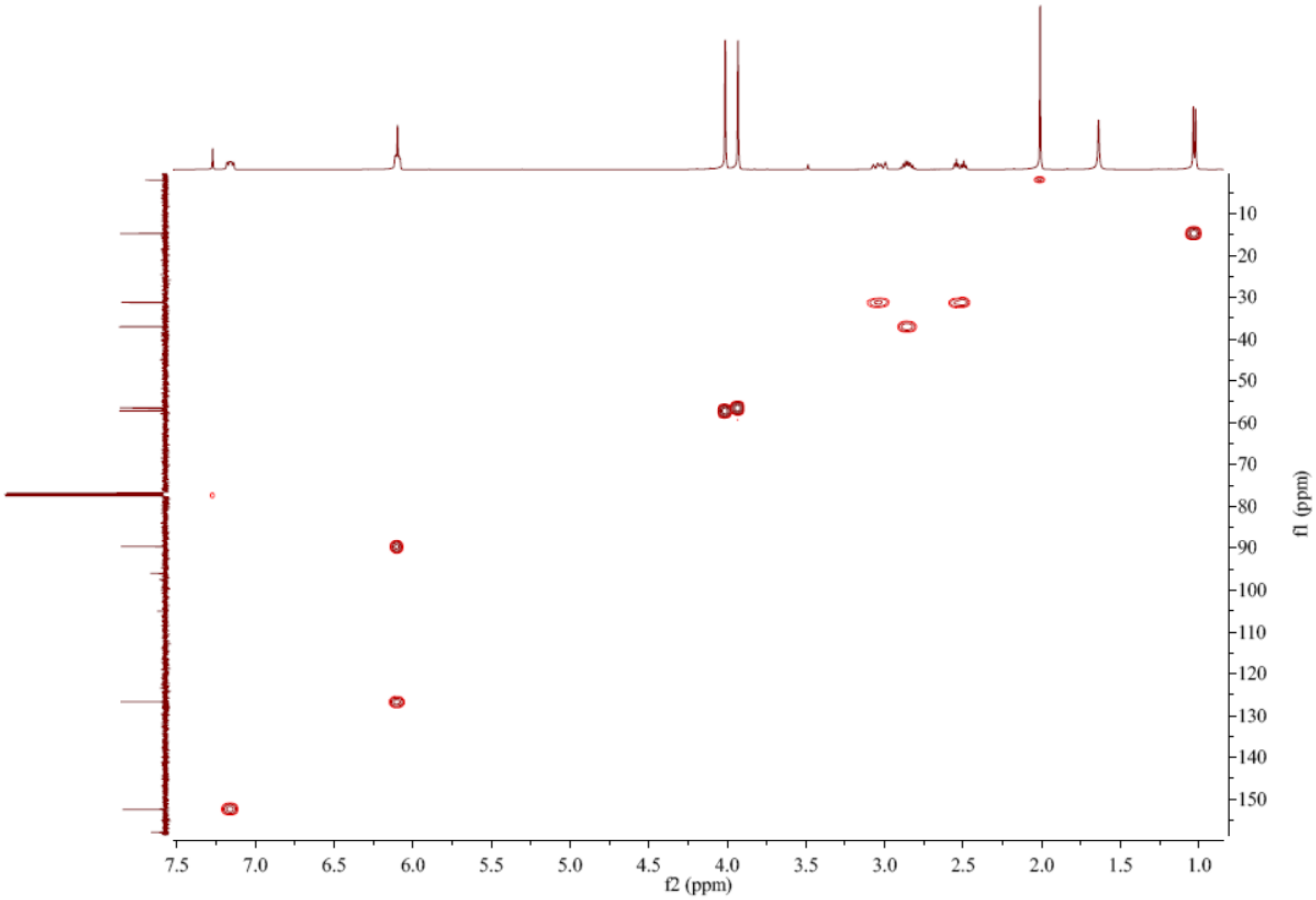

S3.48. $^1H-^1H$ COSY spectrum of **13** in $CDCl_3$

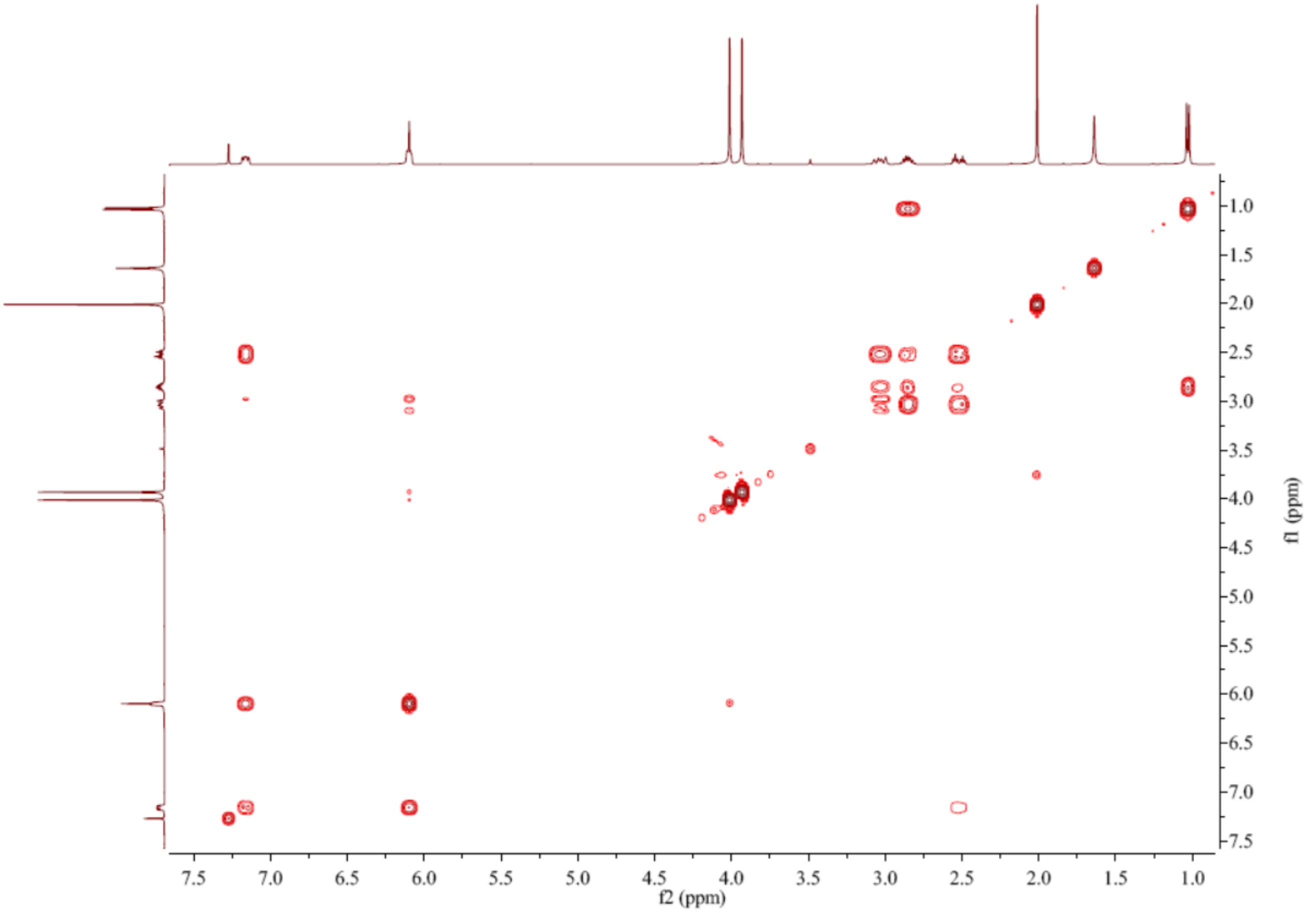

S3.49. HMBC spectrum of **13** in $CDCl_3$

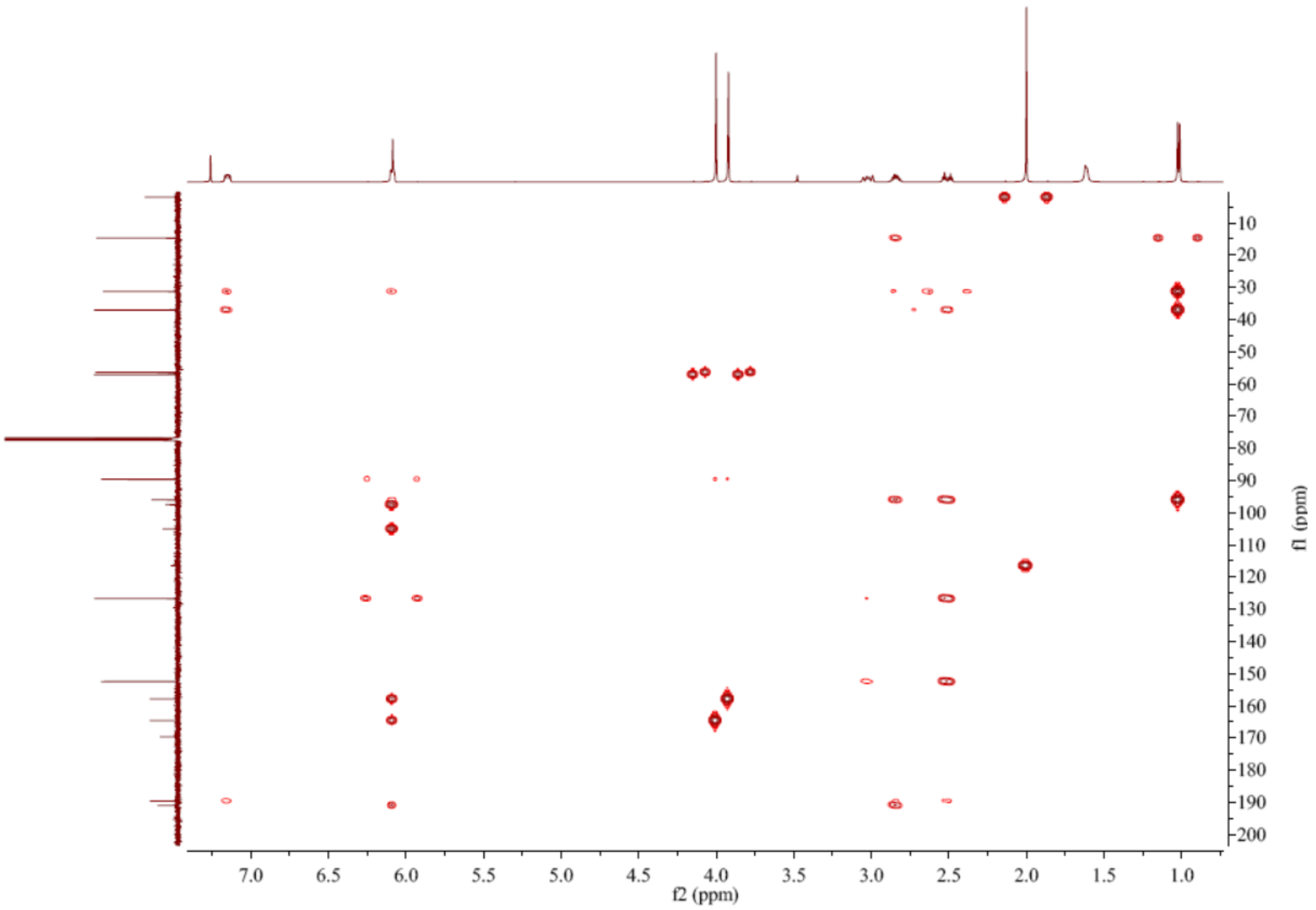

S3.50. ^{1}H NMR spectrum of **14** in $CDCl_3$

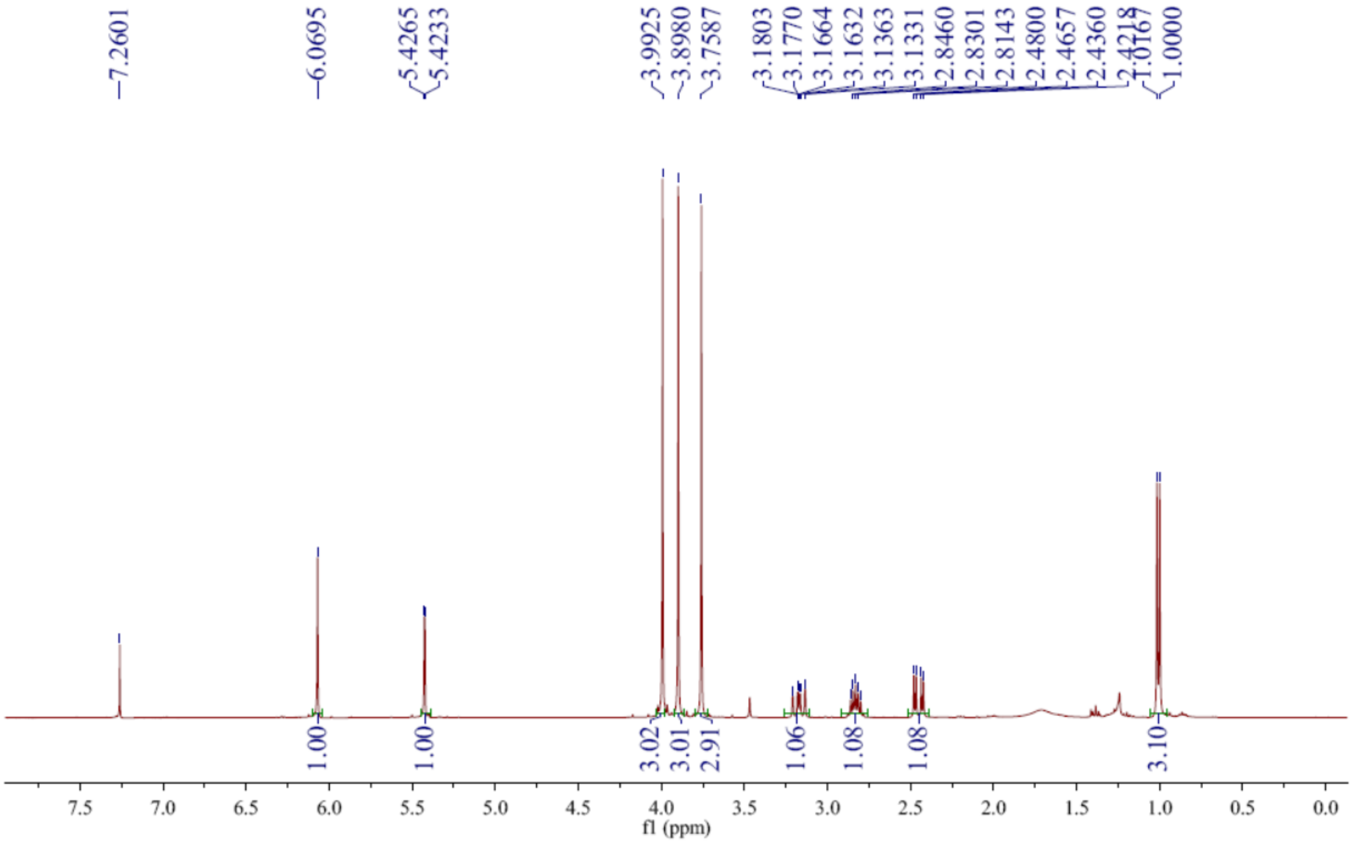

S3.51. ^{13}C NMR and DEPT spectra of **14** in $CDCl_3$

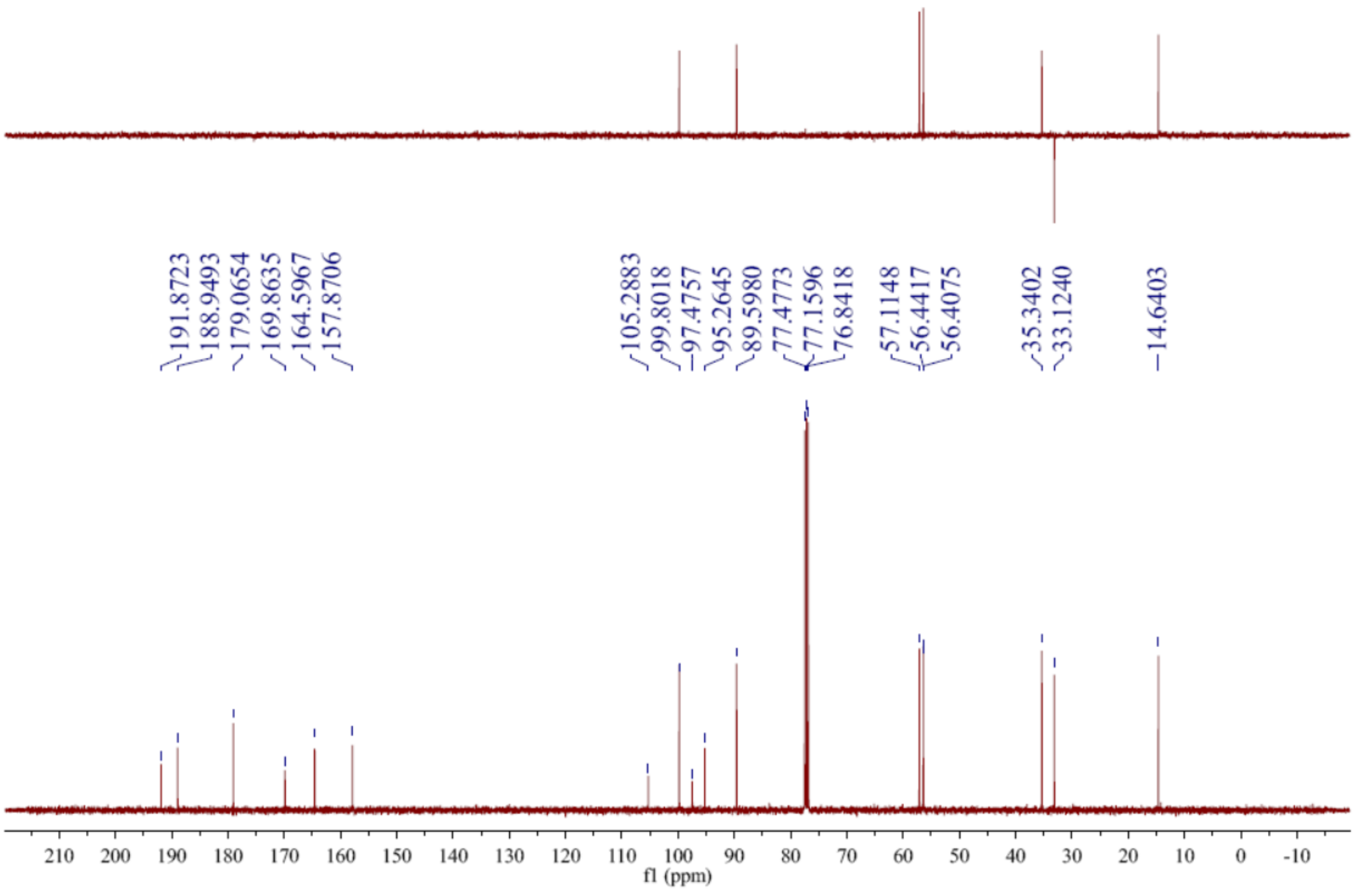

S3.52. ^{1}H NMR spectrum of **15** in pyridine-d_5

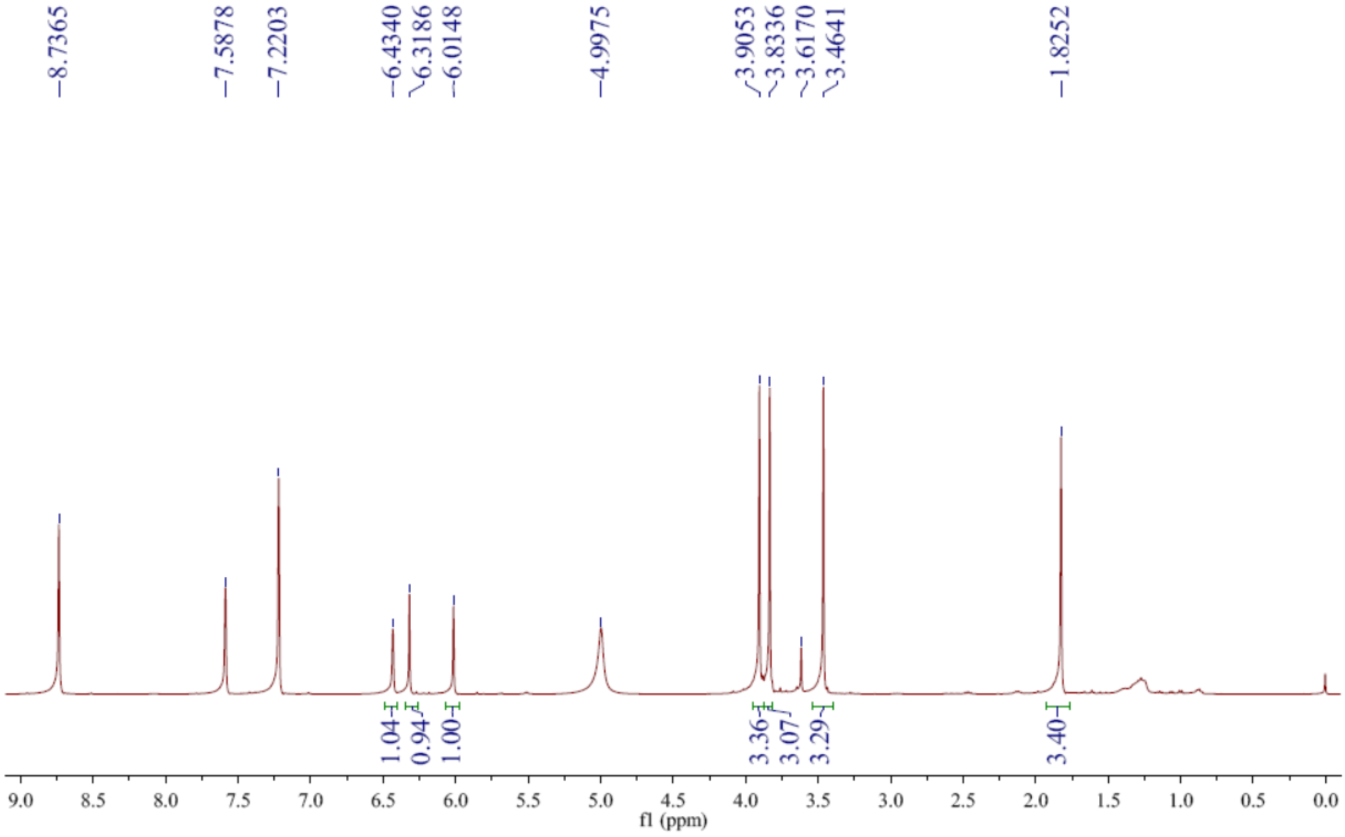

S3.53. ^{13}C NMR and DEPT spectra of **15** in pyridine-d_5

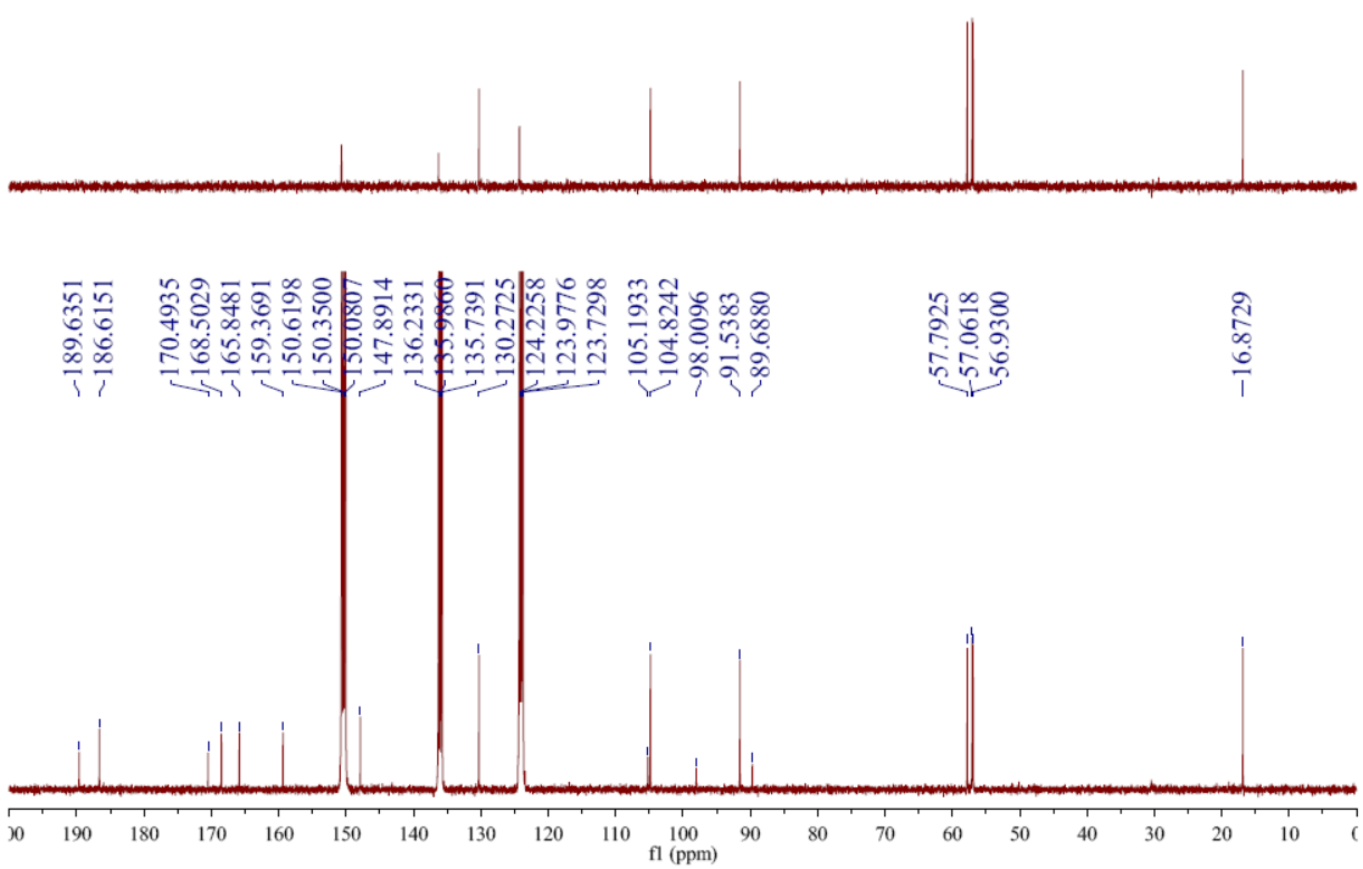

S3.54. ^{1}H NMR spectrum of **16** in CD_3OD

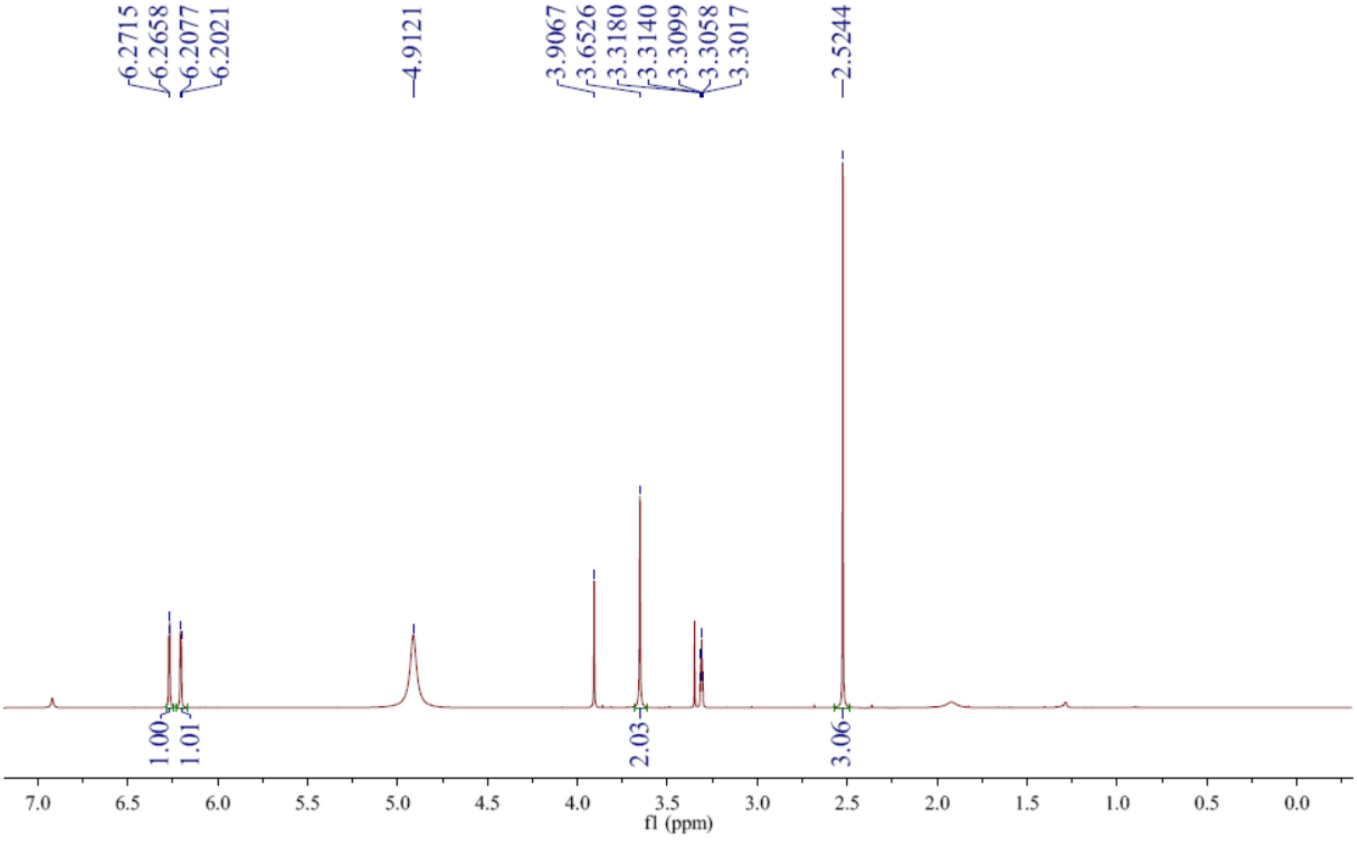

S3.55. ^{13}C NMR and DEPT spectra of **16** in CD_3OD

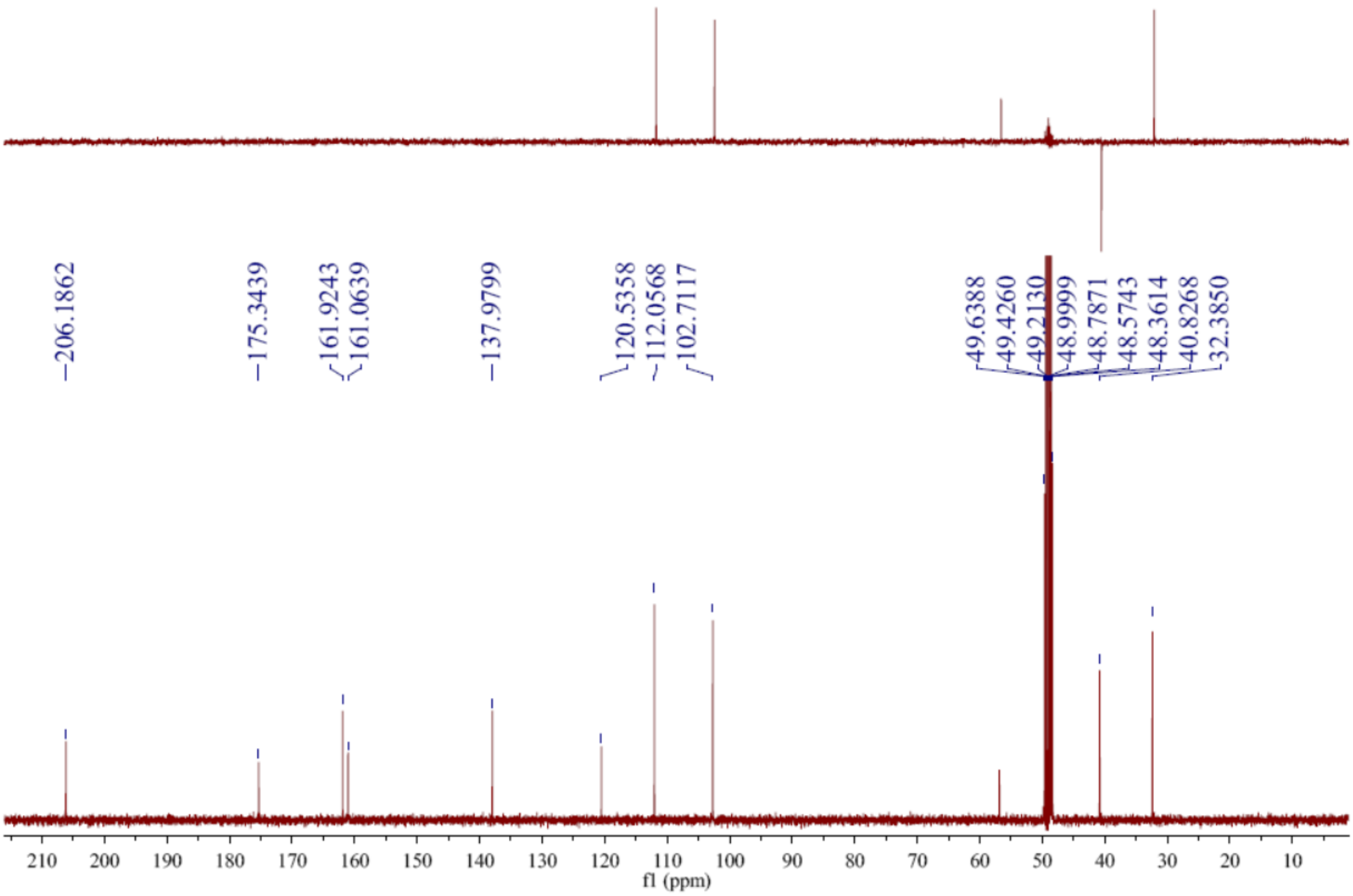

S3.56. ^{1}H NMR spectrum of **17** in CD_3OD

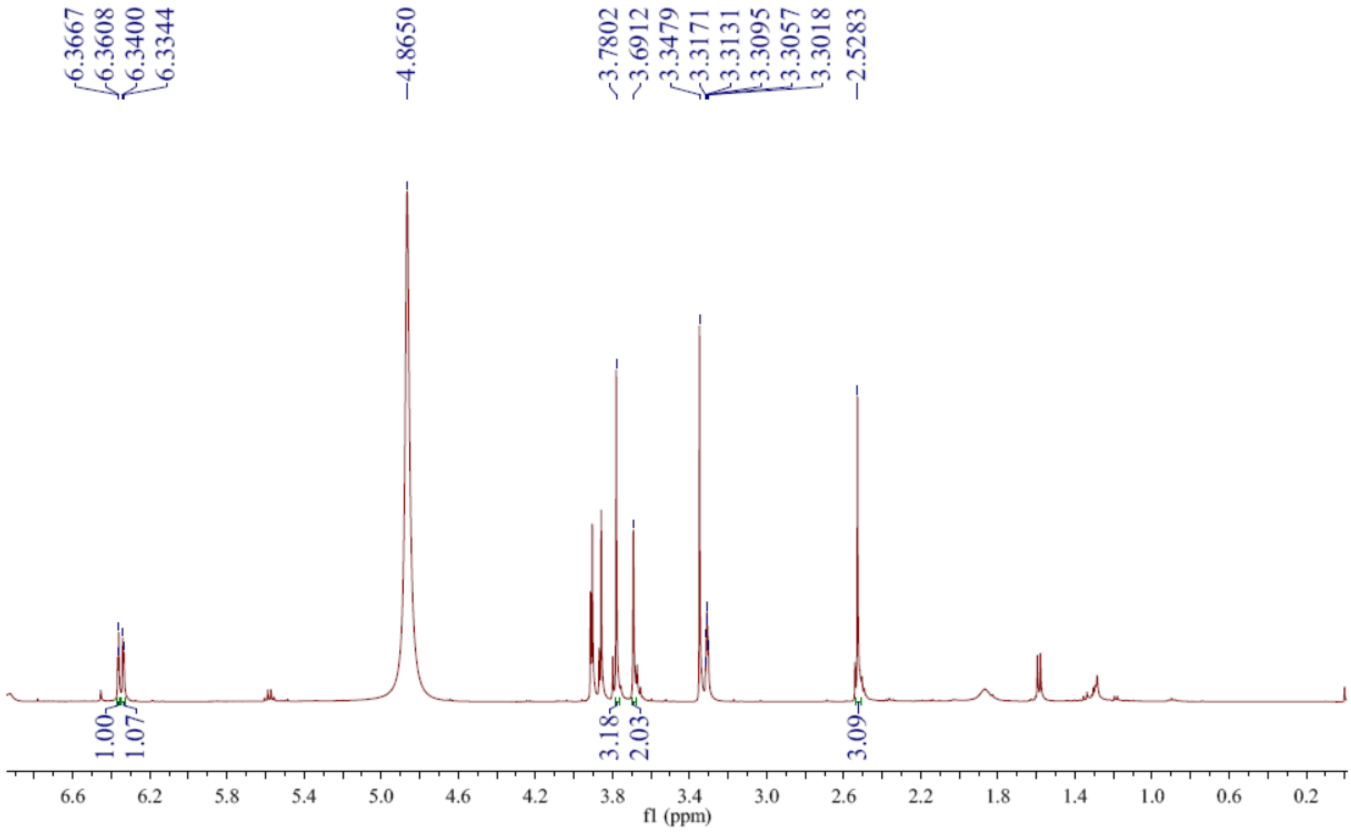

S3.57. ^{13}C NMR and DEPT spectra of **17** in CD_3OD

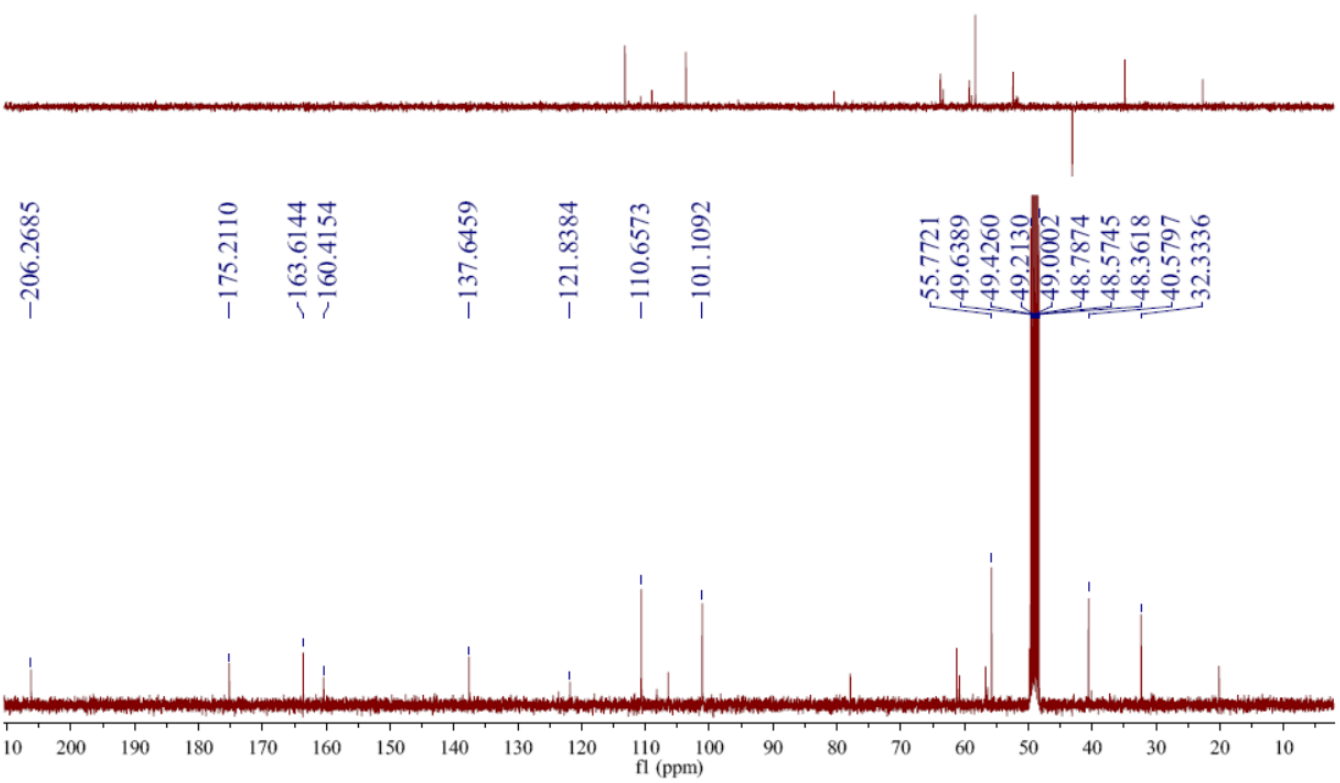

S3.58. ^{1}H NMR spectrum of **18** in CD_3OD

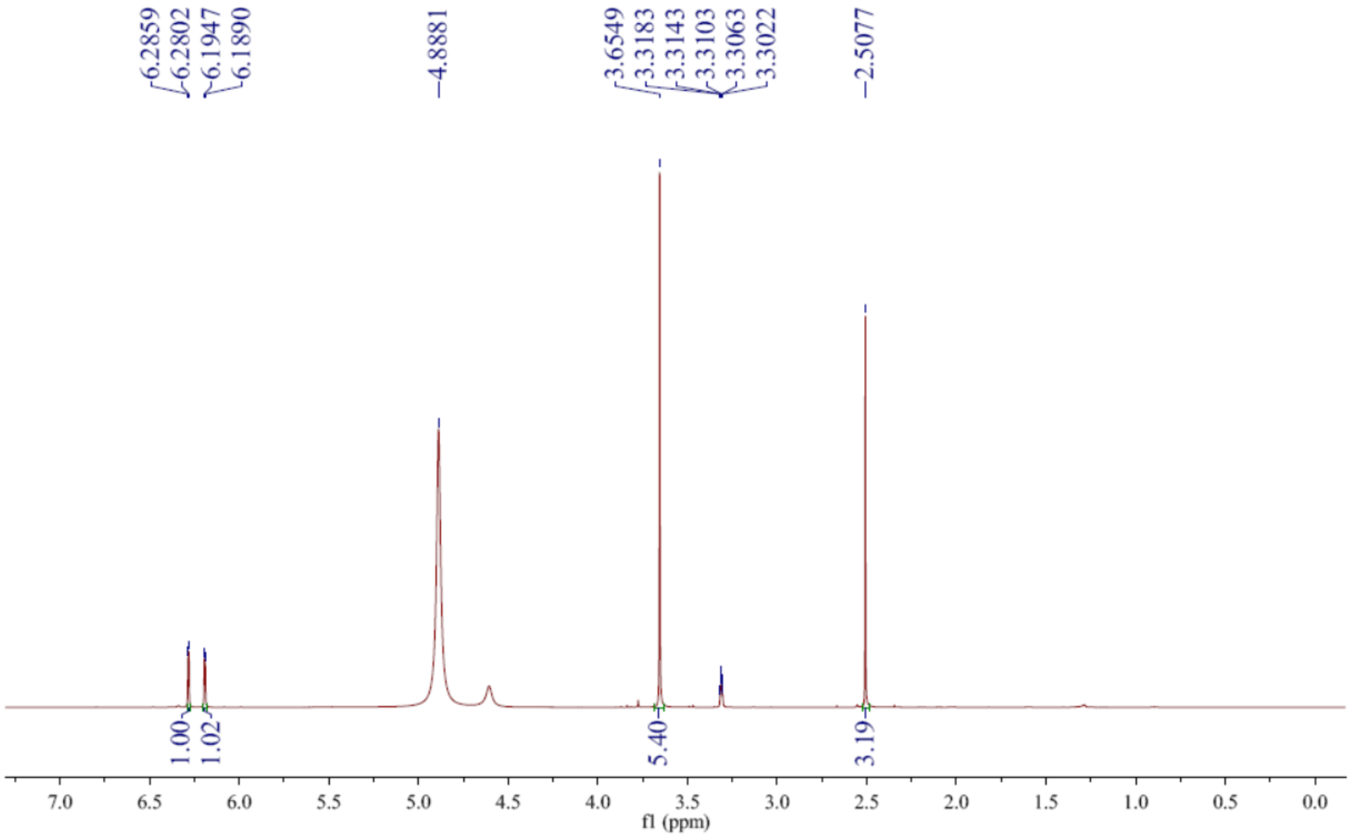

S3.59. ^{13}C NMR and DEPT spectra of **18** in CD_3OD

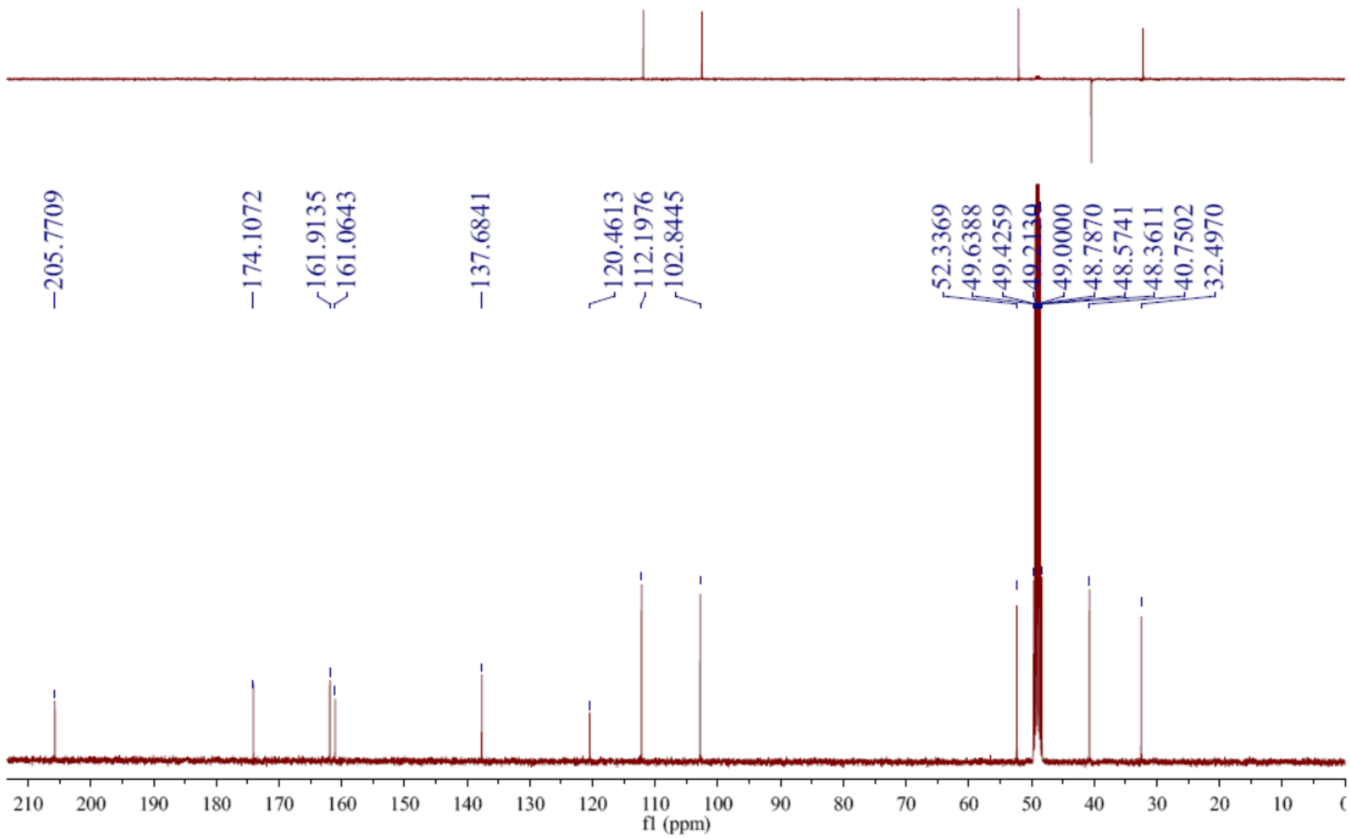

S3.60. ^{1}H NMR spectrum of **19** in $CDCl_3$

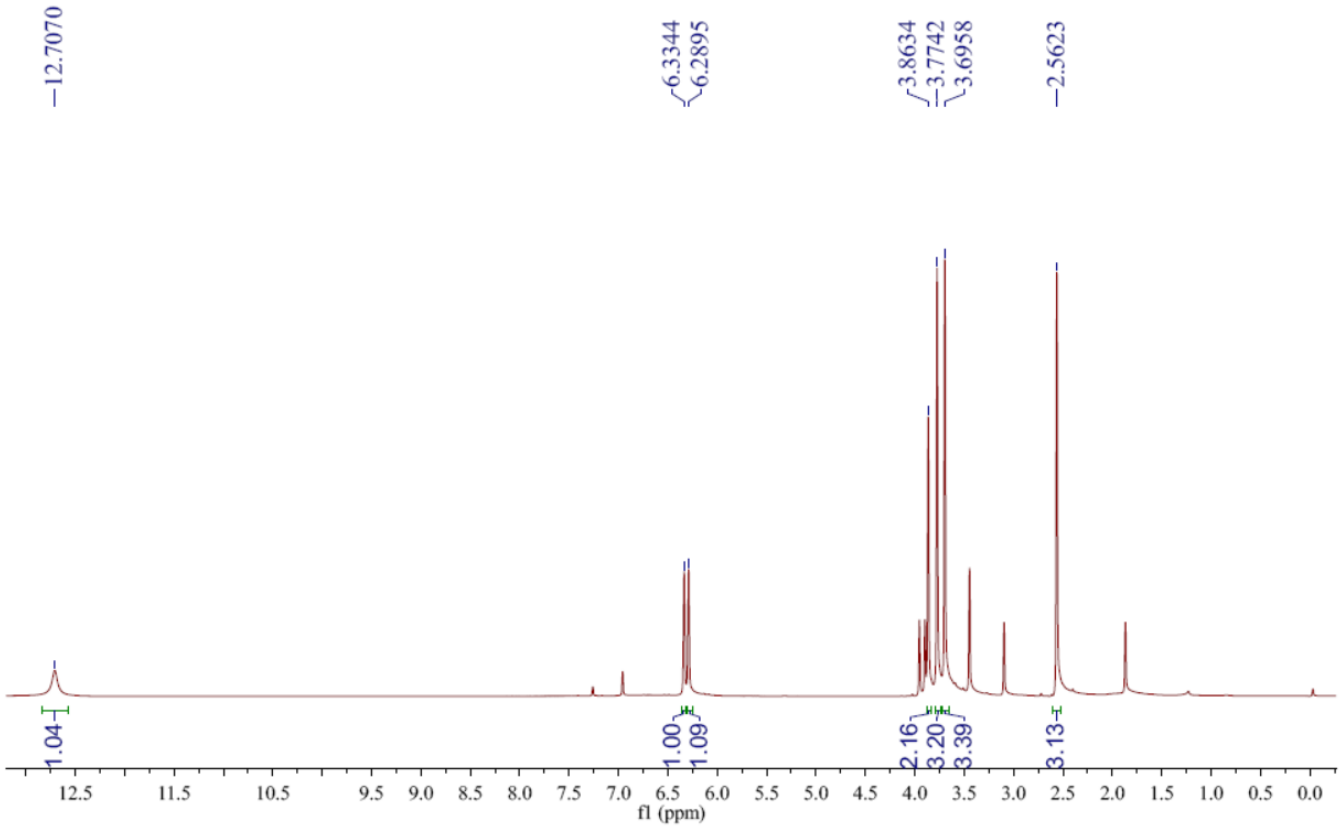

S3.61. ^{13}C NMR spectrum of **19** in $CDCl_3$

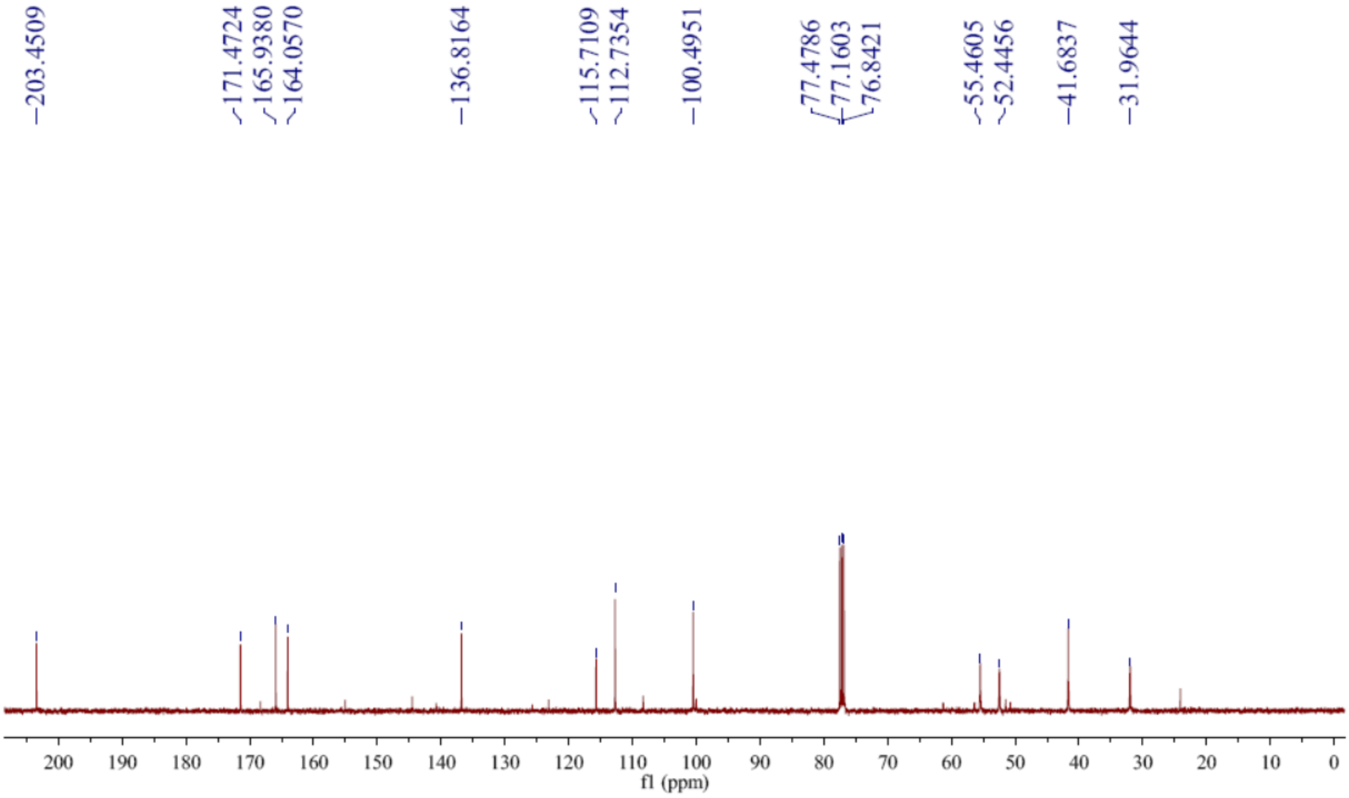

S3.62. ^{1}H NMR spectrum of **20** in $CDCl_3$

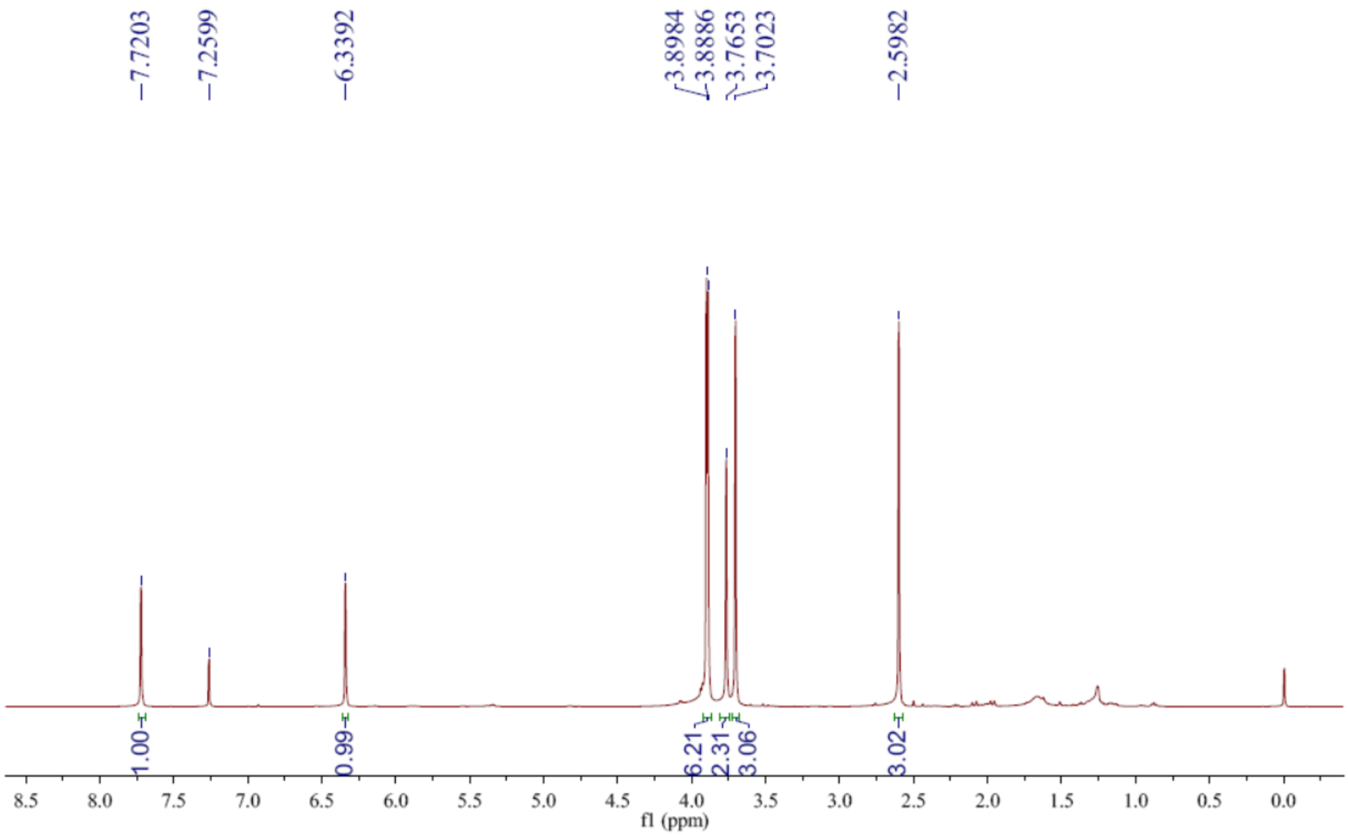

S3.63. ^{13}C NMR and DEPT spectra of **20** in $CDCl_3$

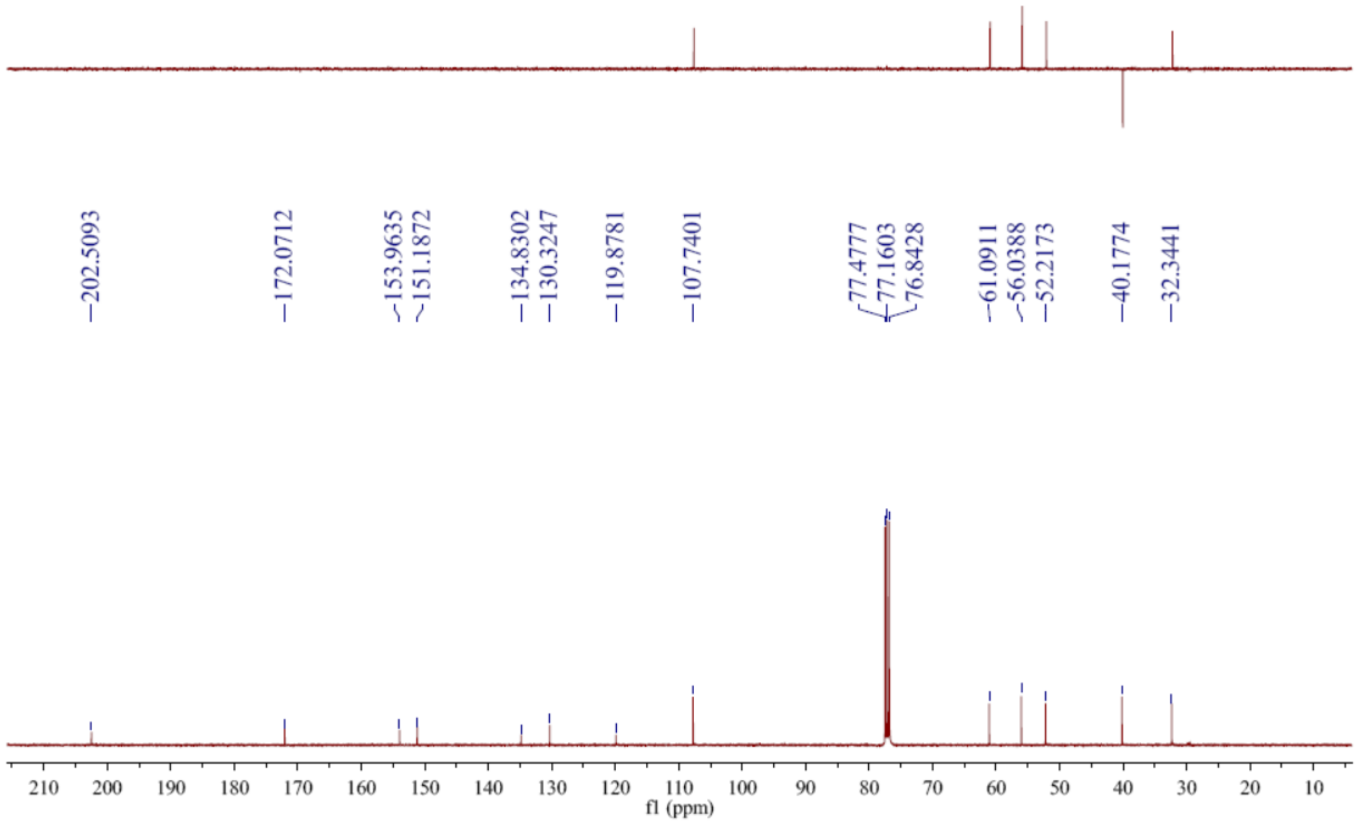

S3.64. HSQC spectrum of **20** in $CDCl_3$

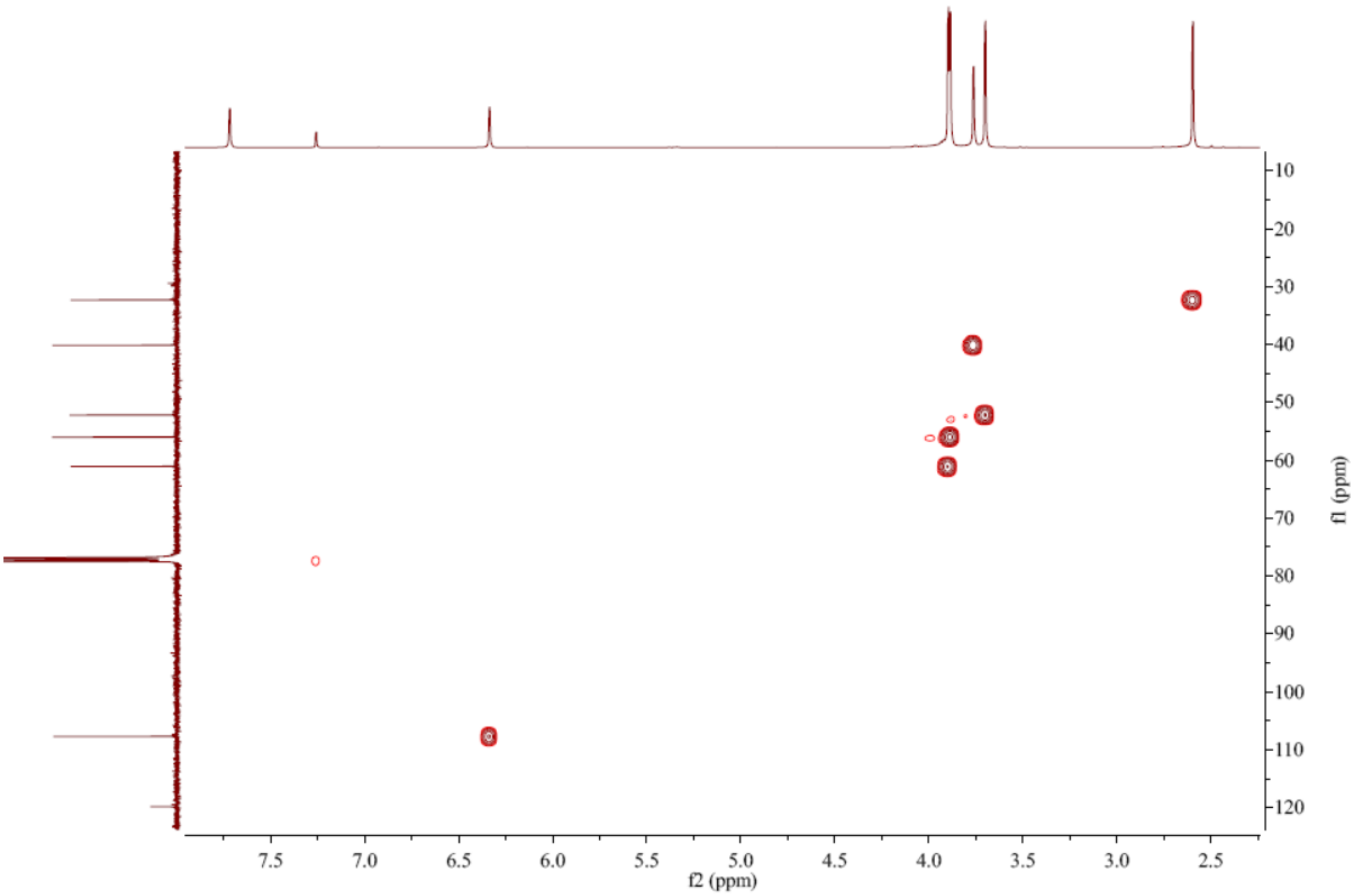

S3.65. HMBC spectrum of **20** in $CDCl_3$

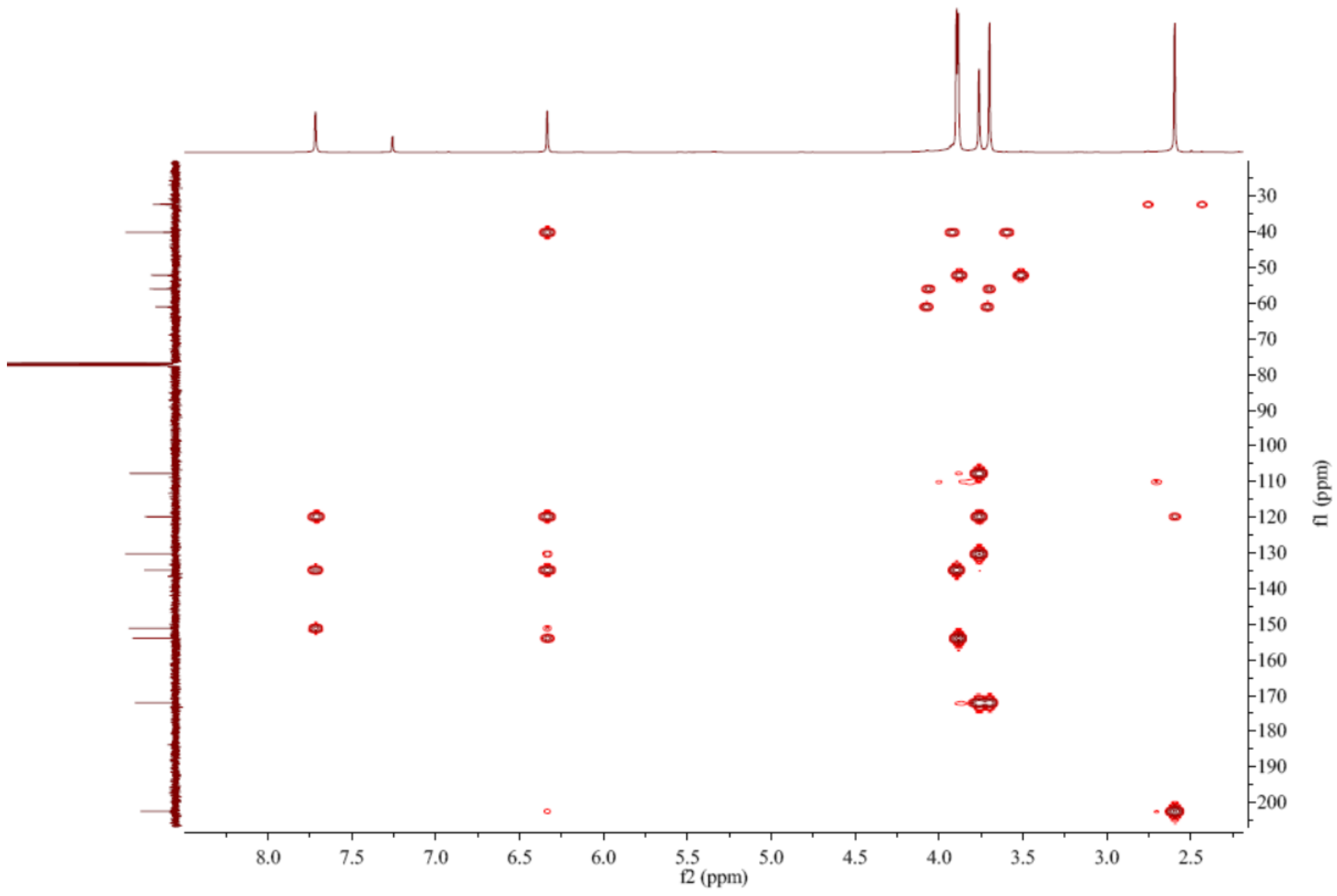

S3.66. ^{1}H NMR spectrum of **21** in CD_3OD

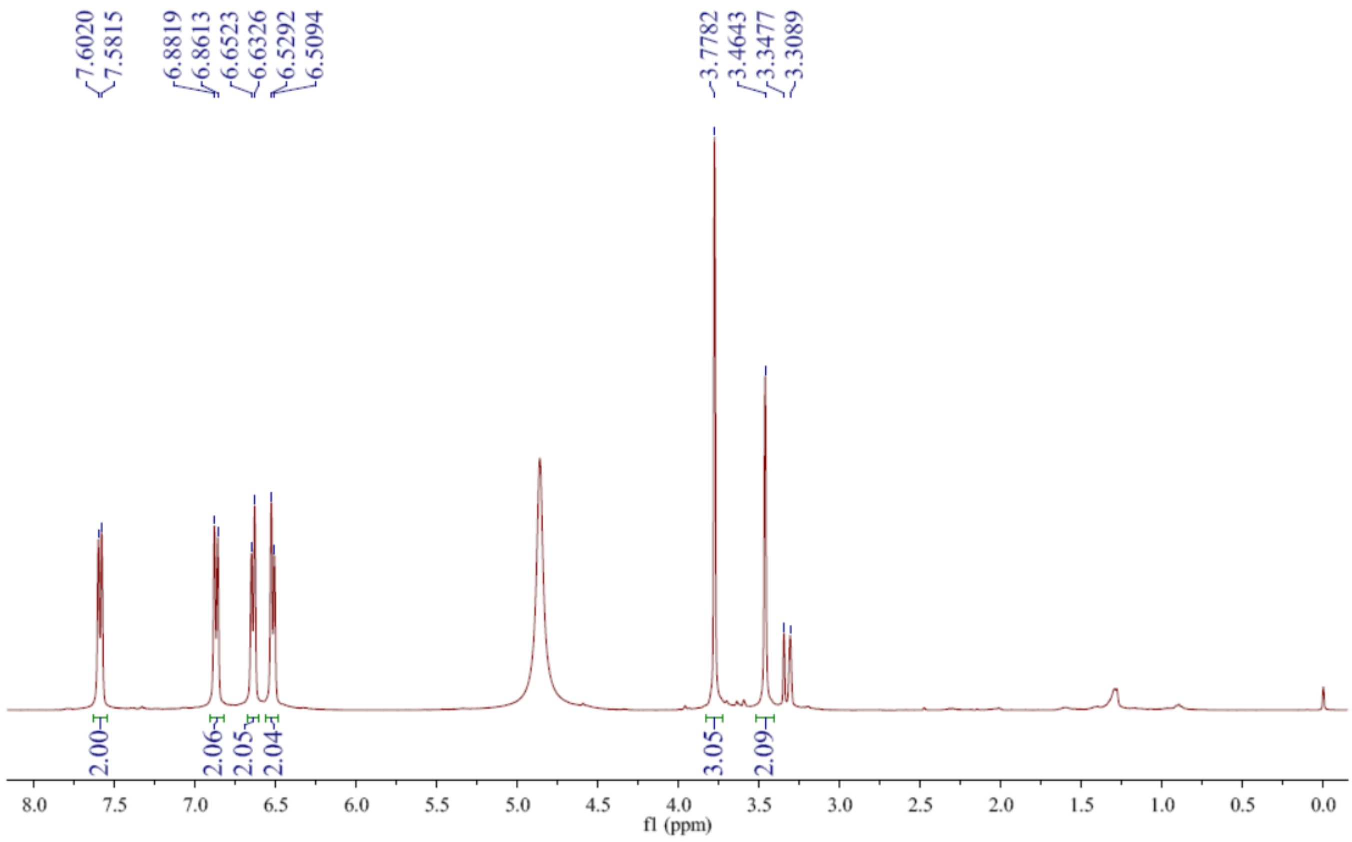

S3.67. ^{13}C NMR and DEPT spectra of **21** in CD_3OD

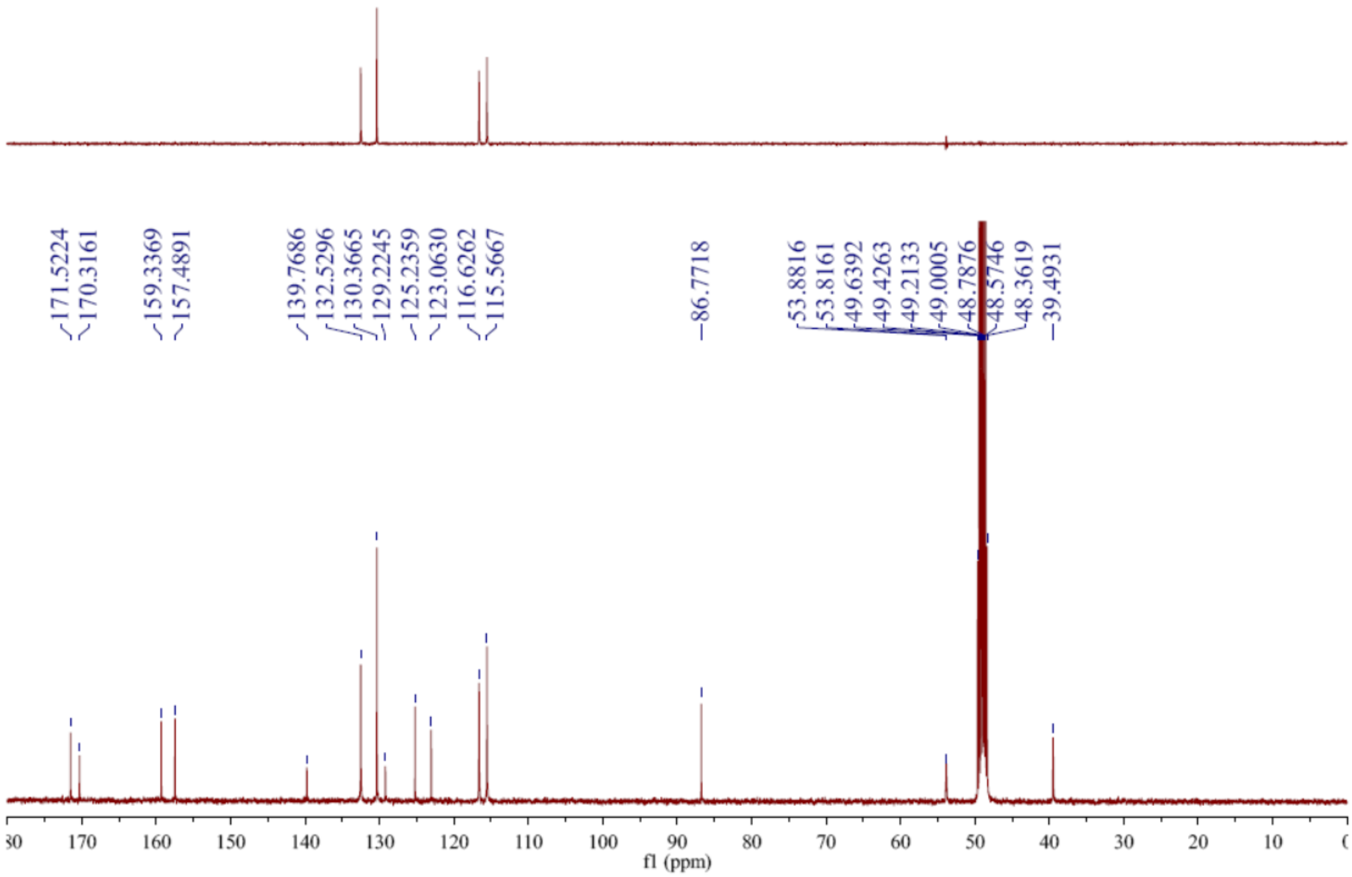

S3.68. ^1H NMR spectrum of **22** in CD_3OD

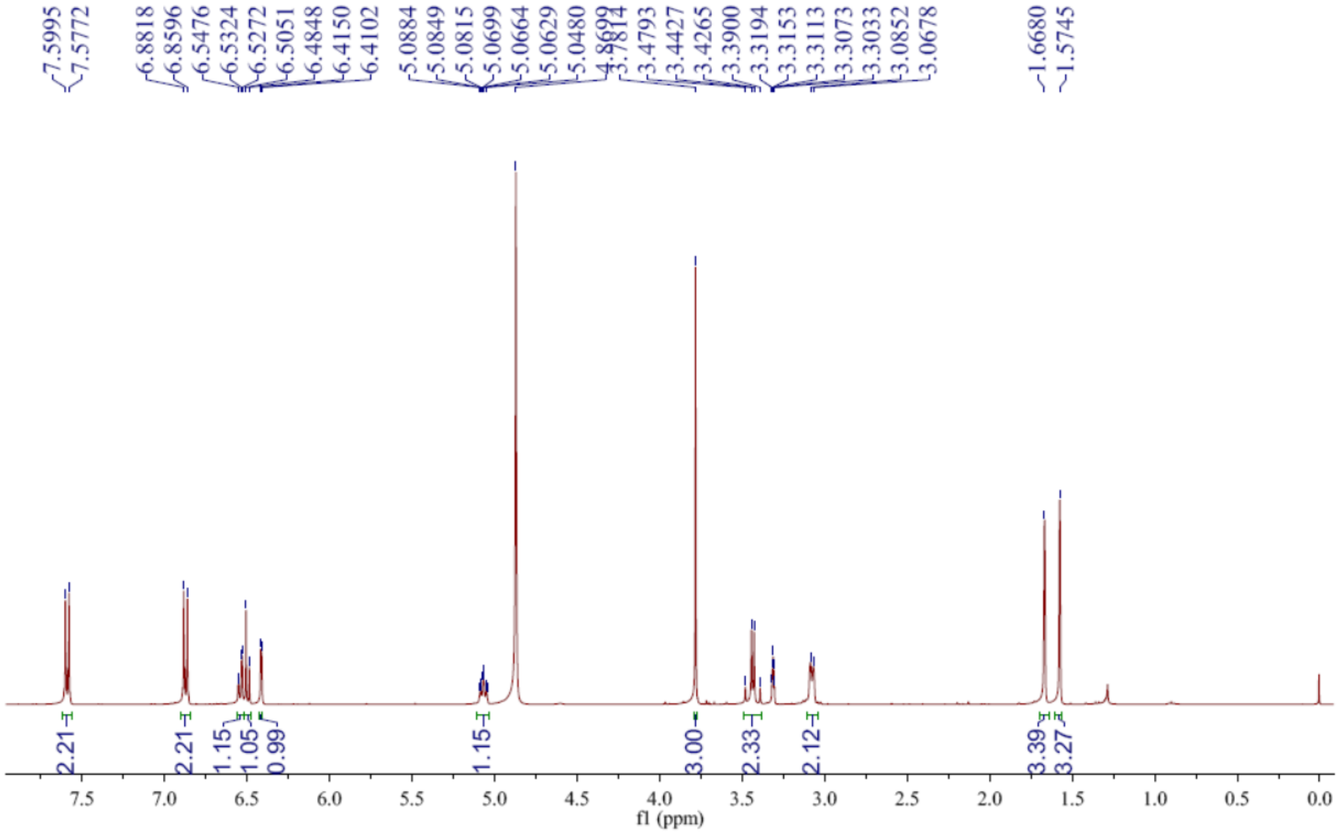

S3.69. ^{13}C NMR and DEPT spectra of **22** in CD_3OD

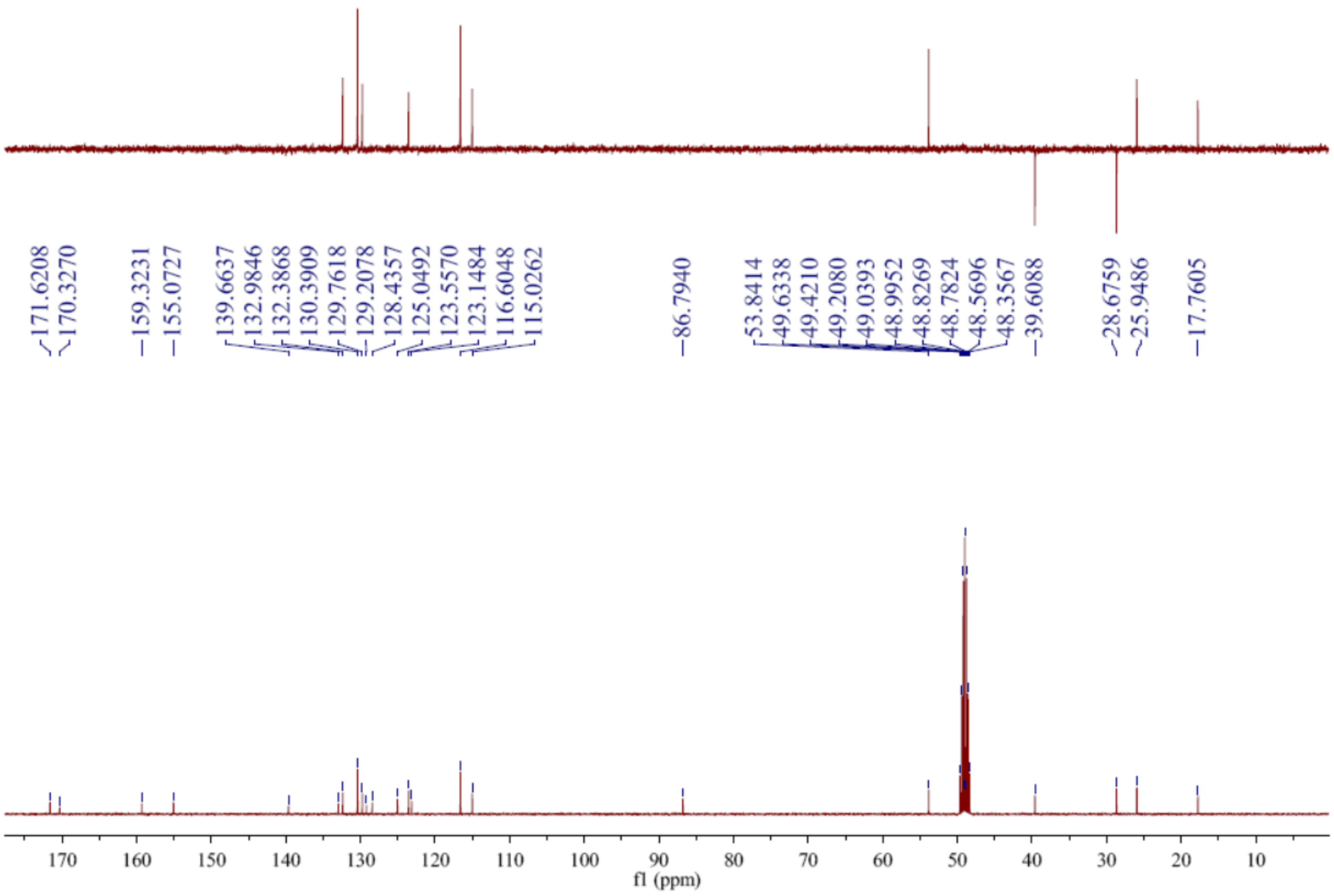

S3.70. ^{1}H NMR spectrum of **23** in CD_3OD

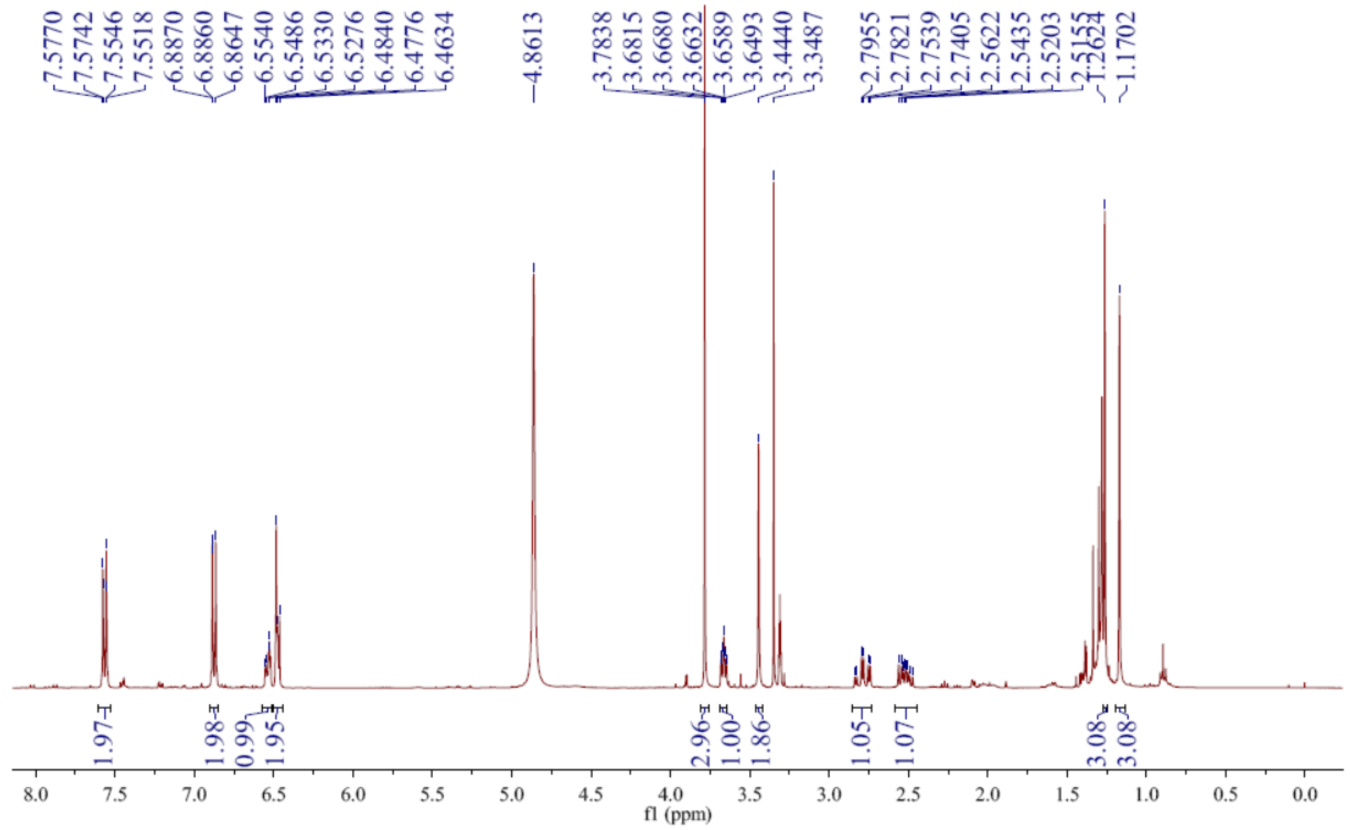

S3.71. ^{13}C NMR and DEPT spectra of **23** in CD_3OD

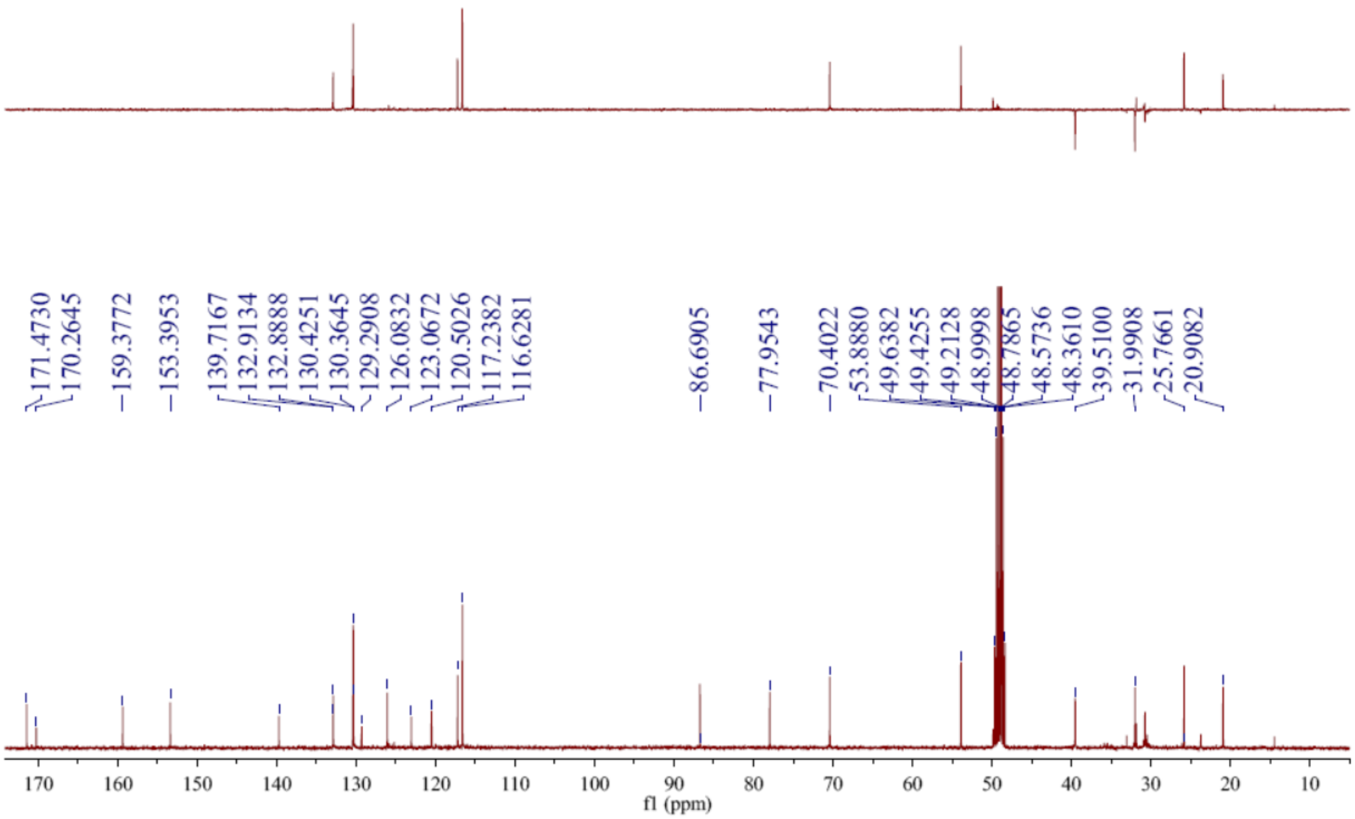

S3.72. ^{1}H NMR spectrum of **24** in $CDCl_3$

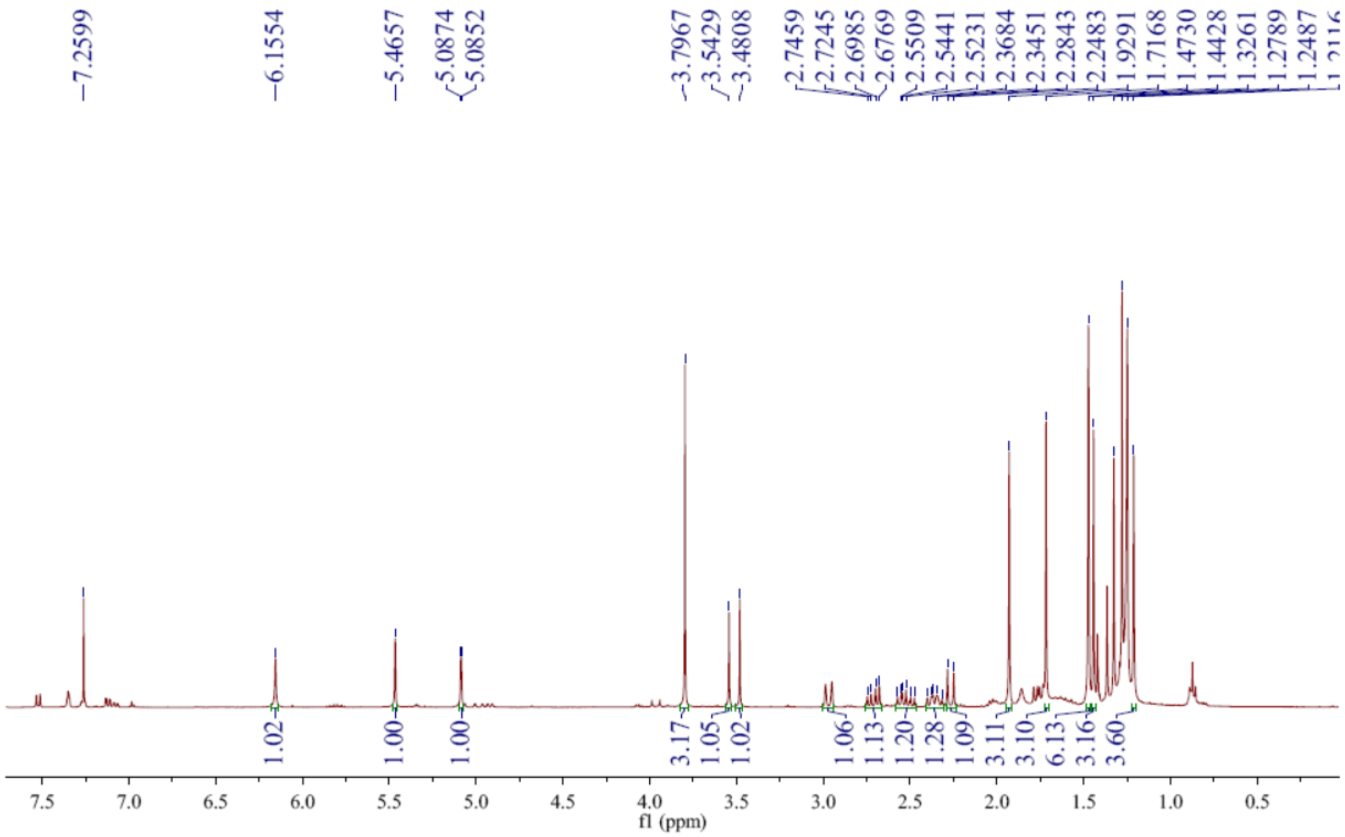

S3.73. ^{13}C NMR and DEPT spectra of **24** in $CDCl_3$

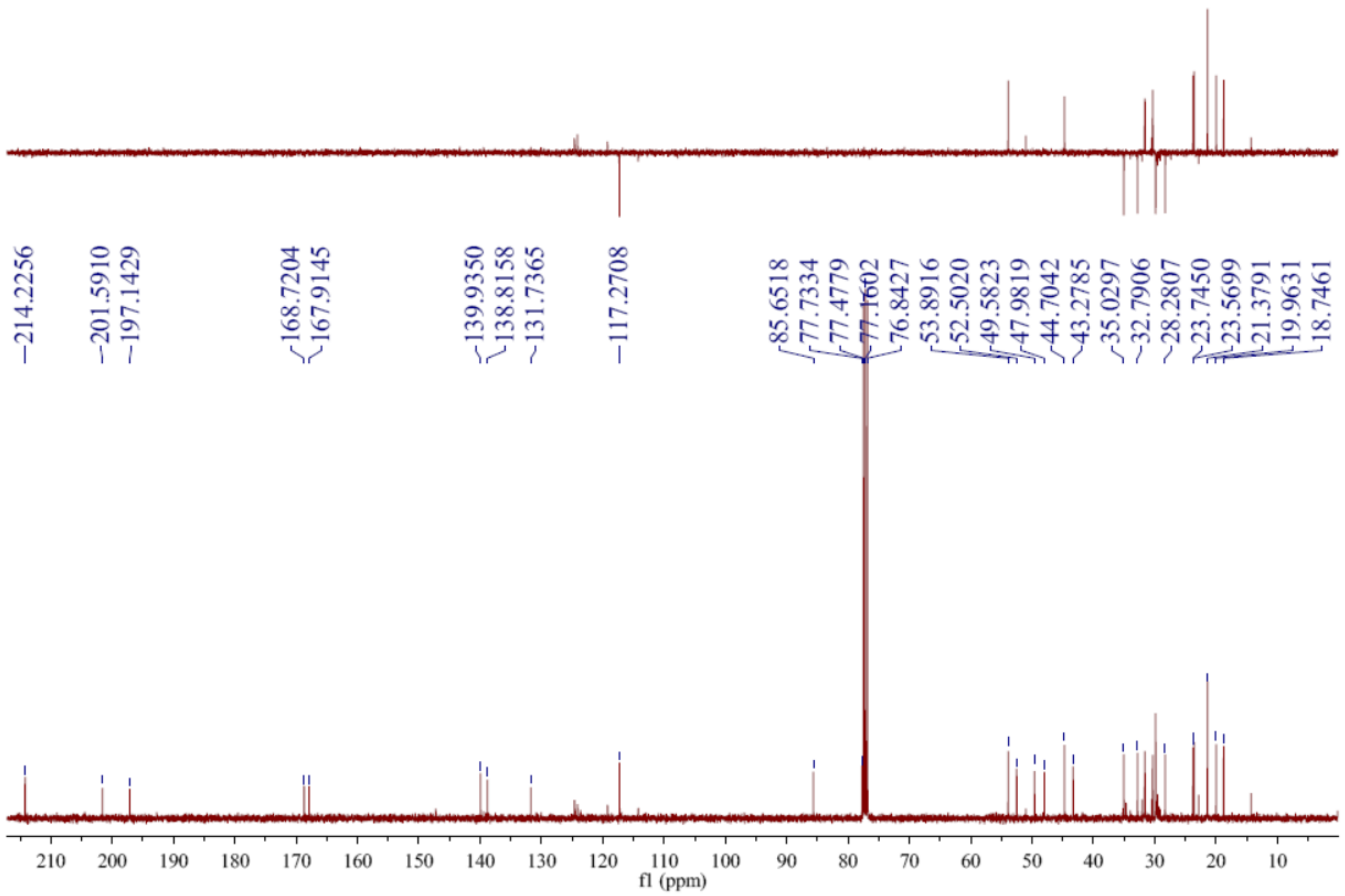

S3.74. HSQC spectrum of **24** in $CDCl_3$

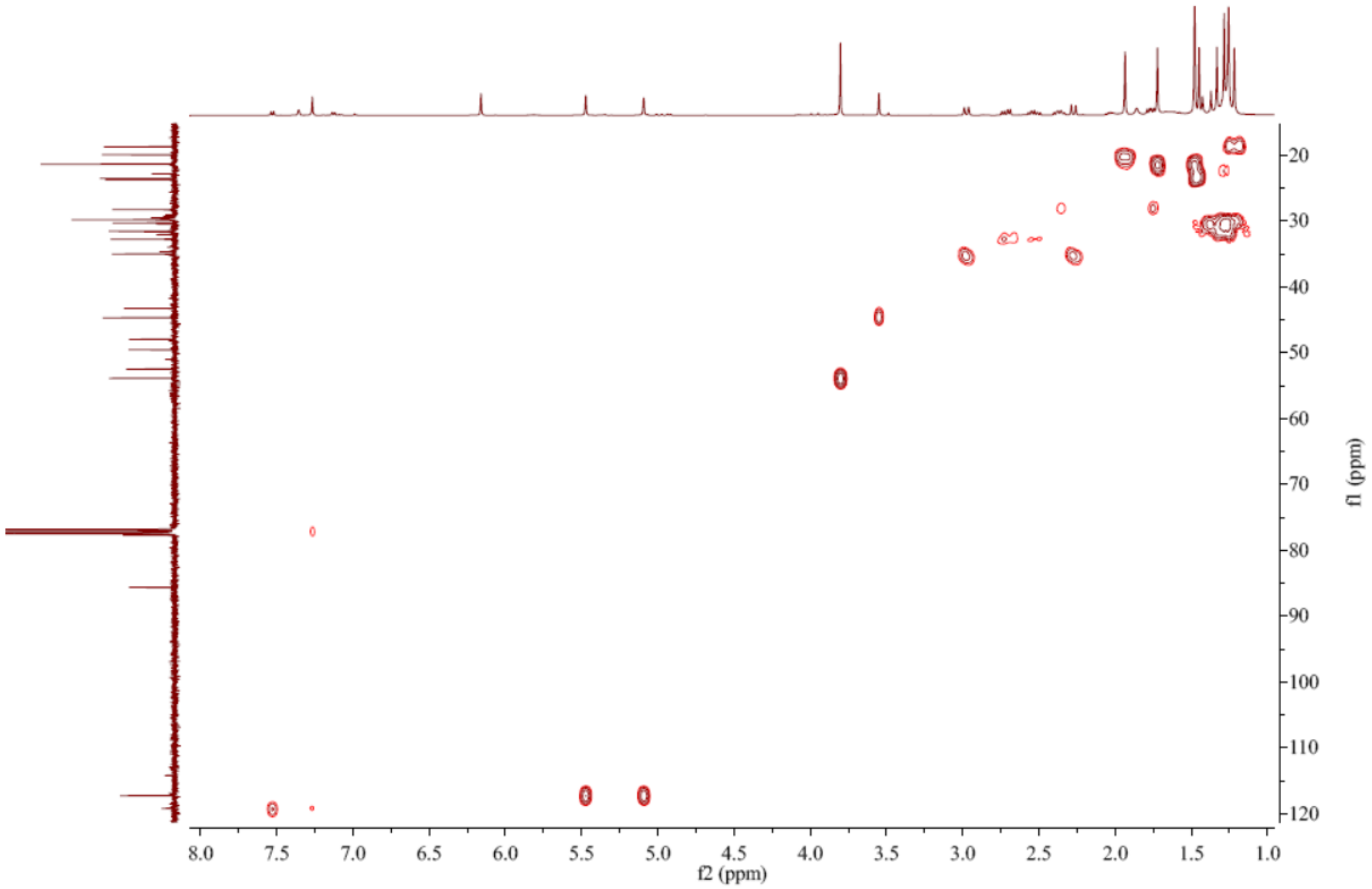

S3.75. $^1H-^1H$ COSY spectrum of **24** in $CDCl_3$

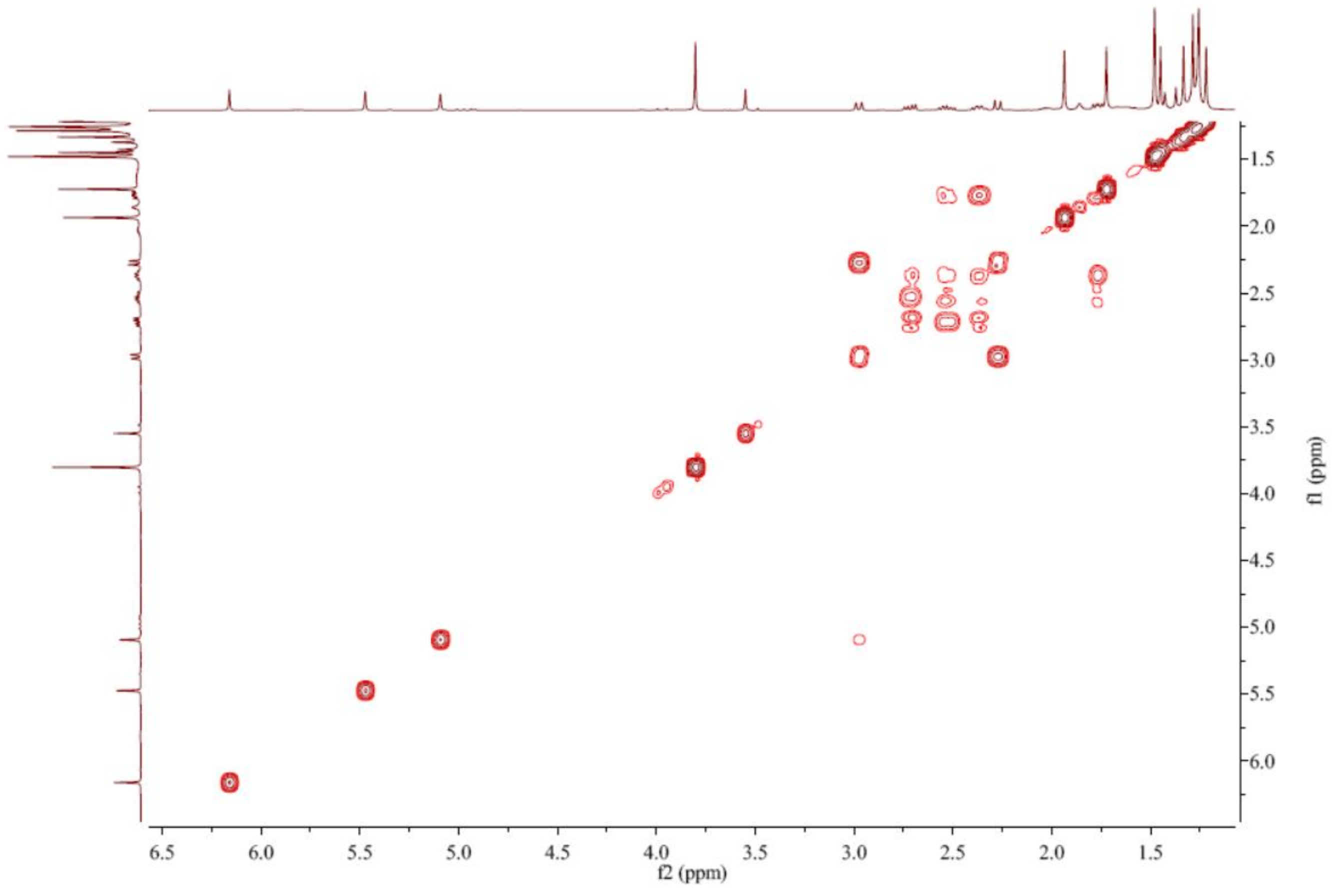

S3.76. HMBC spectrum of **24** in $CDCl_3$

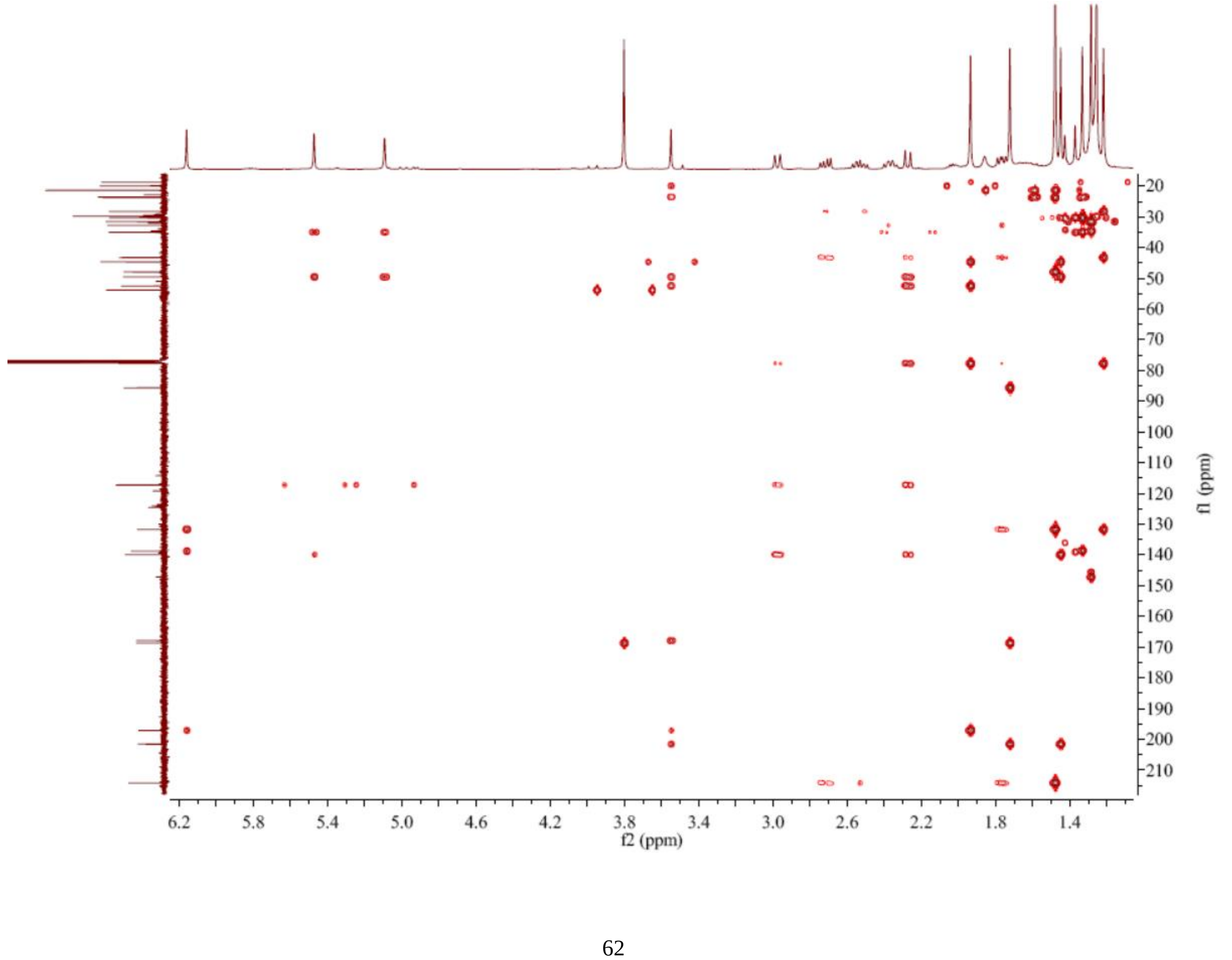

S3.77. ^{1}H NMR spectrum of **25** in CD_3COCD_3

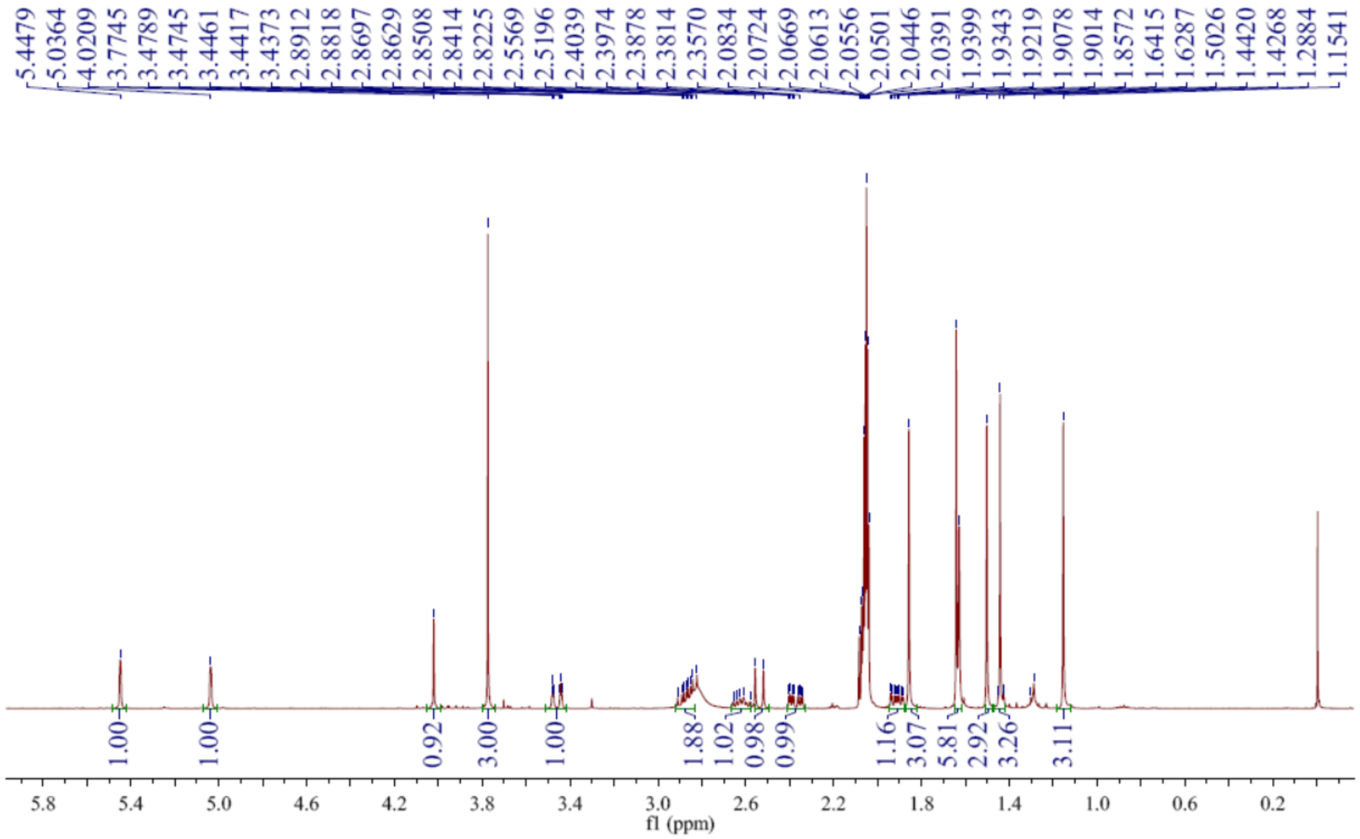

S3.78. ^{13}C NMR spectrum of **25** in CD_3COCD_3

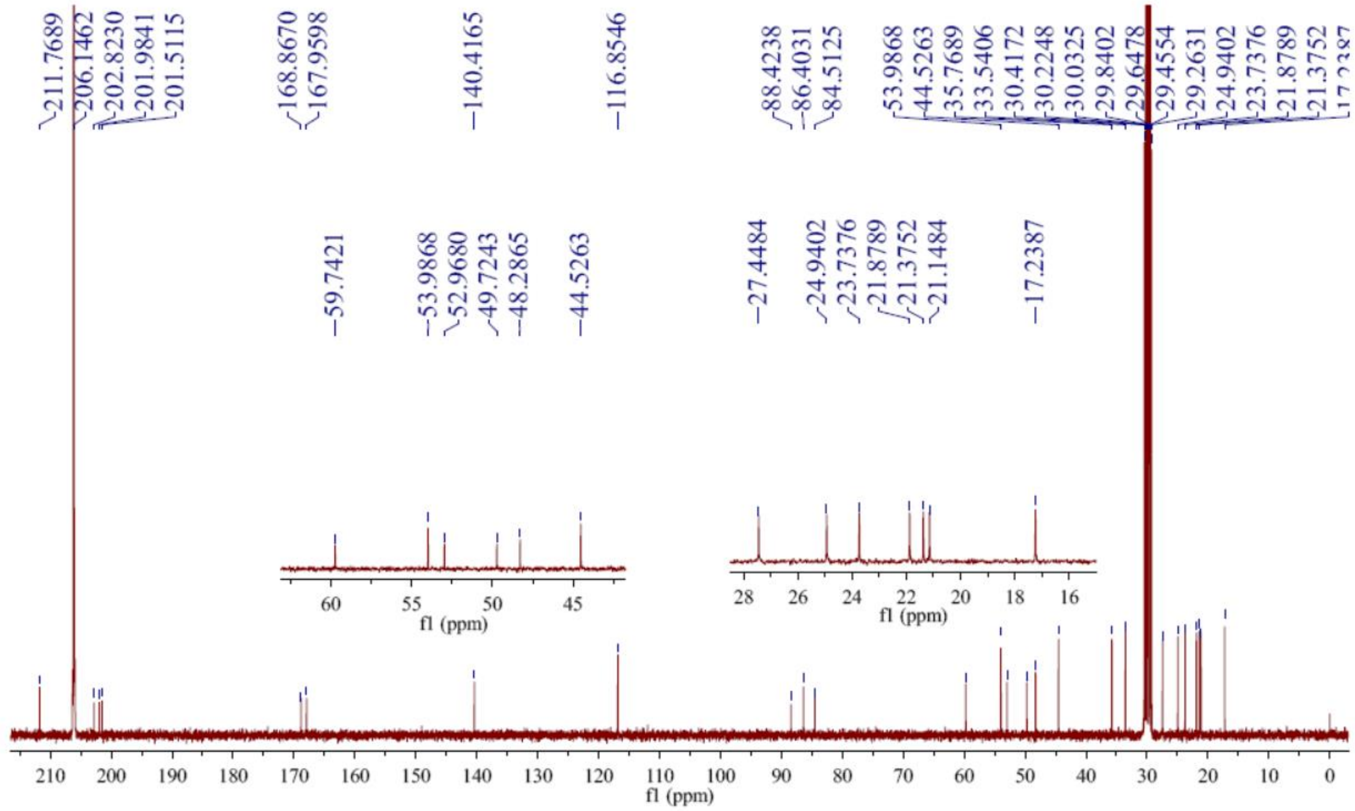

S3.79. ^{1}H NMR spectrum of **26** in $CDCl_3$

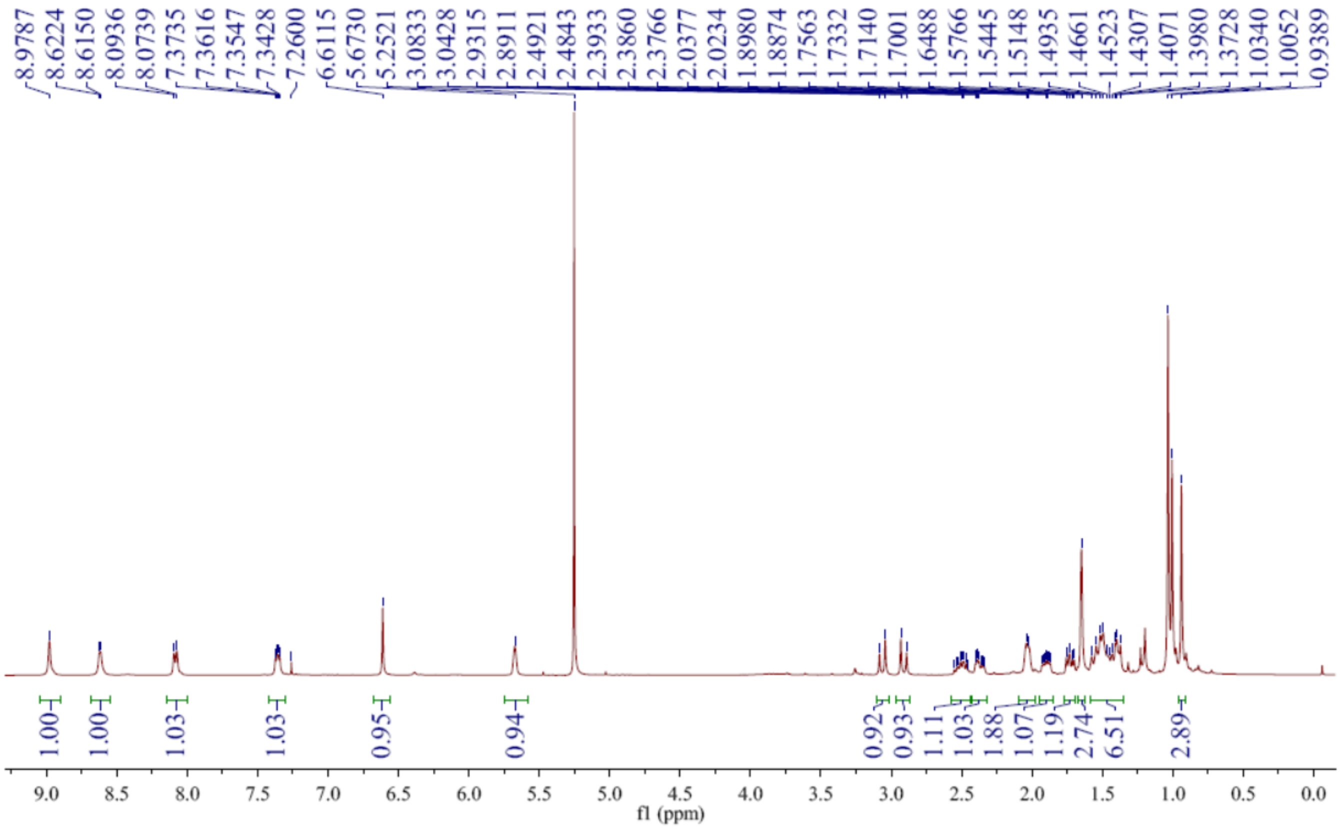

S3.80. ^{13}C NMR and DEPT spectra of **26** in $CDCl_3$

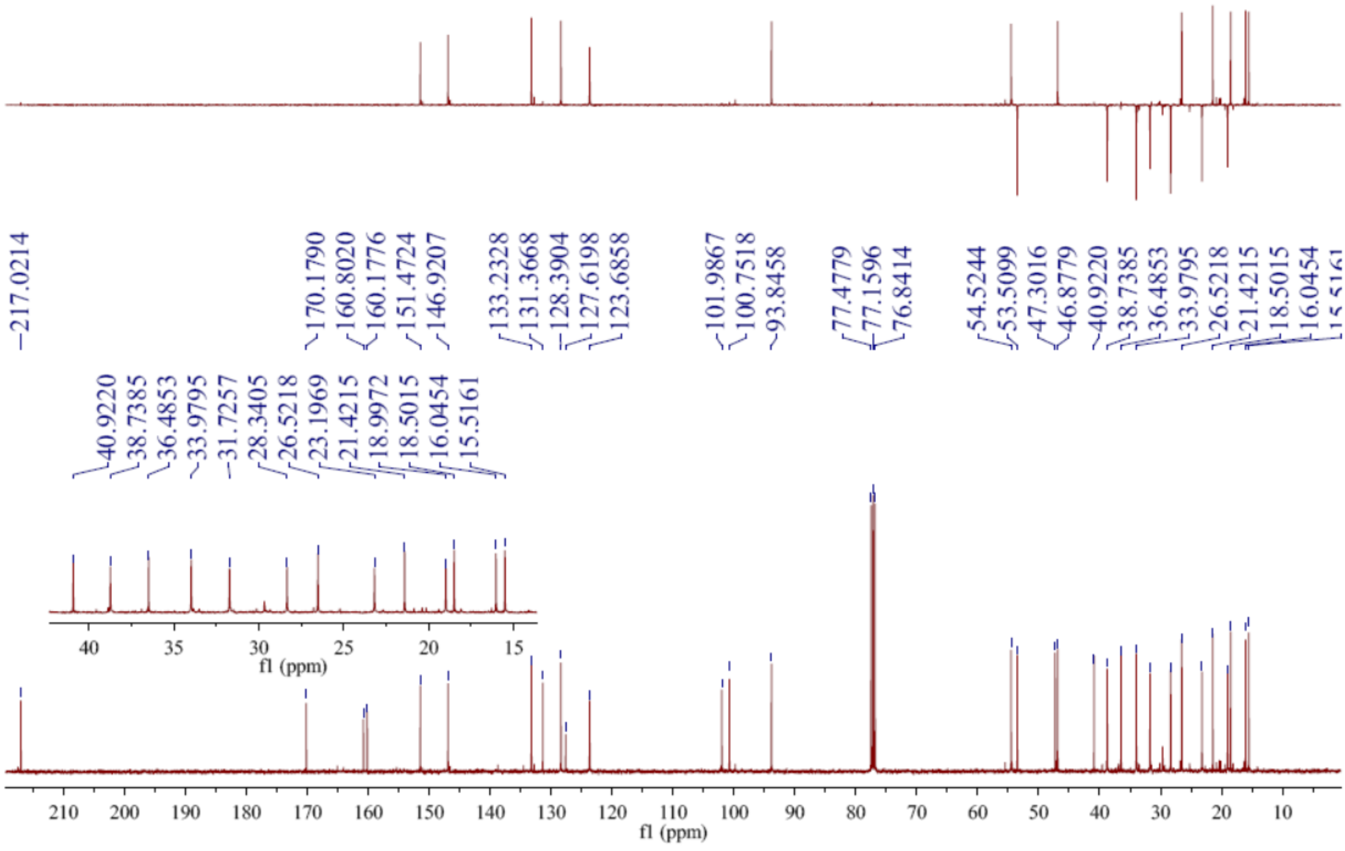

S3.81. ^{1}H NMR spectrum of **27** in $CDCl_3$

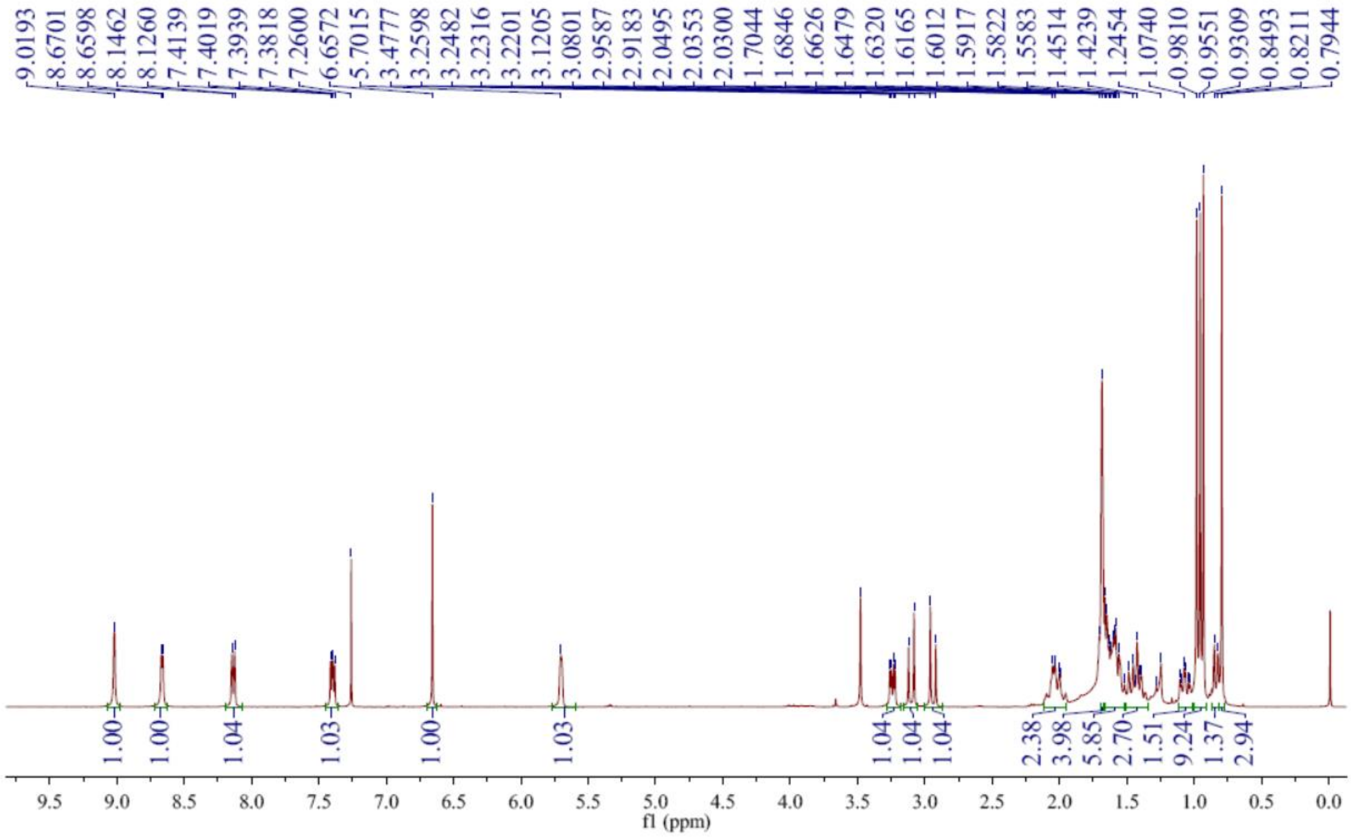

S3.82. ^{13}C NMR and DEPT spectra of **27** in $CDCl_3$

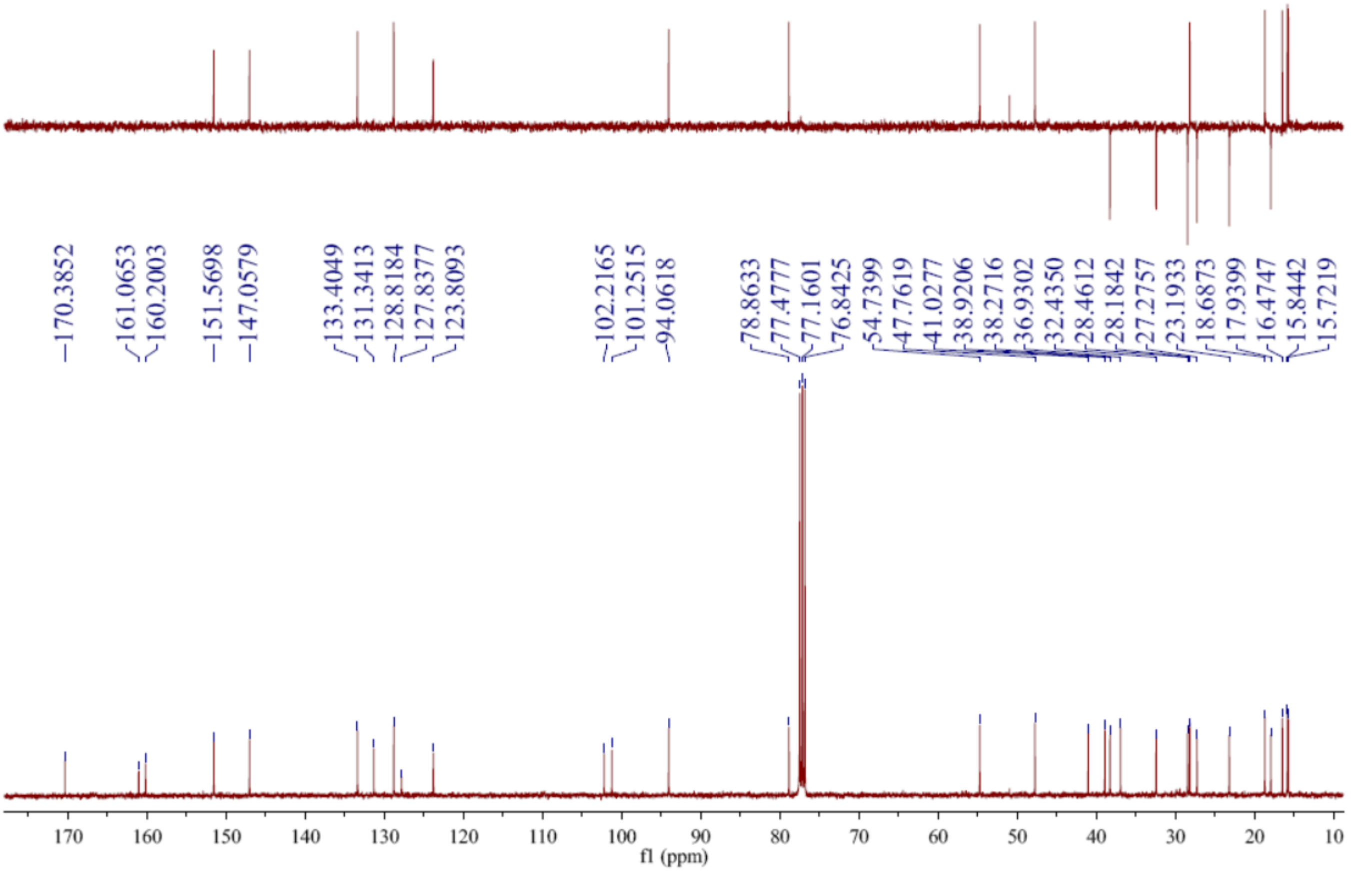

S3.83. ^{1}H NMR spectrum of **28** in $CDCl_3$

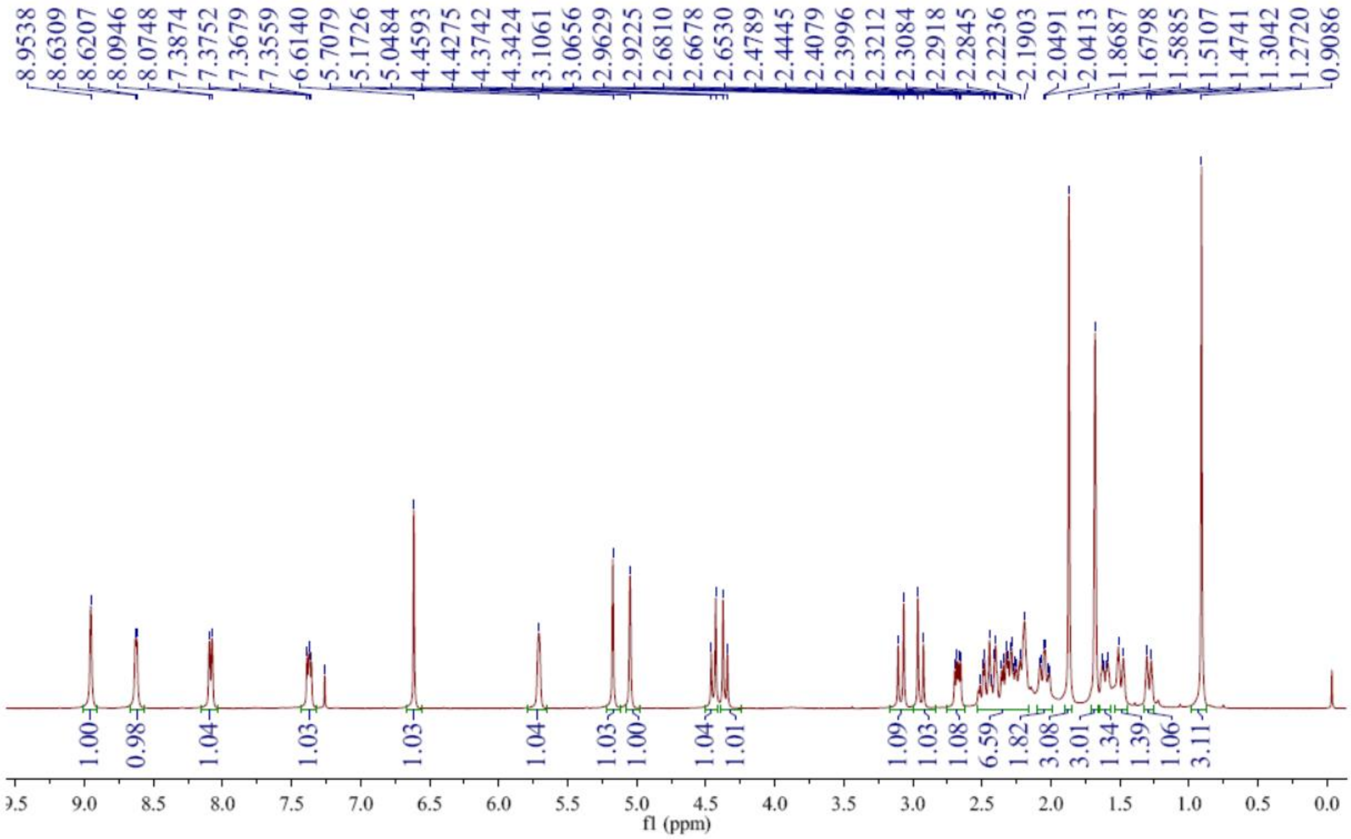

S3.84. ^{13}C NMR spectrum of **28** in $CDCl_3$

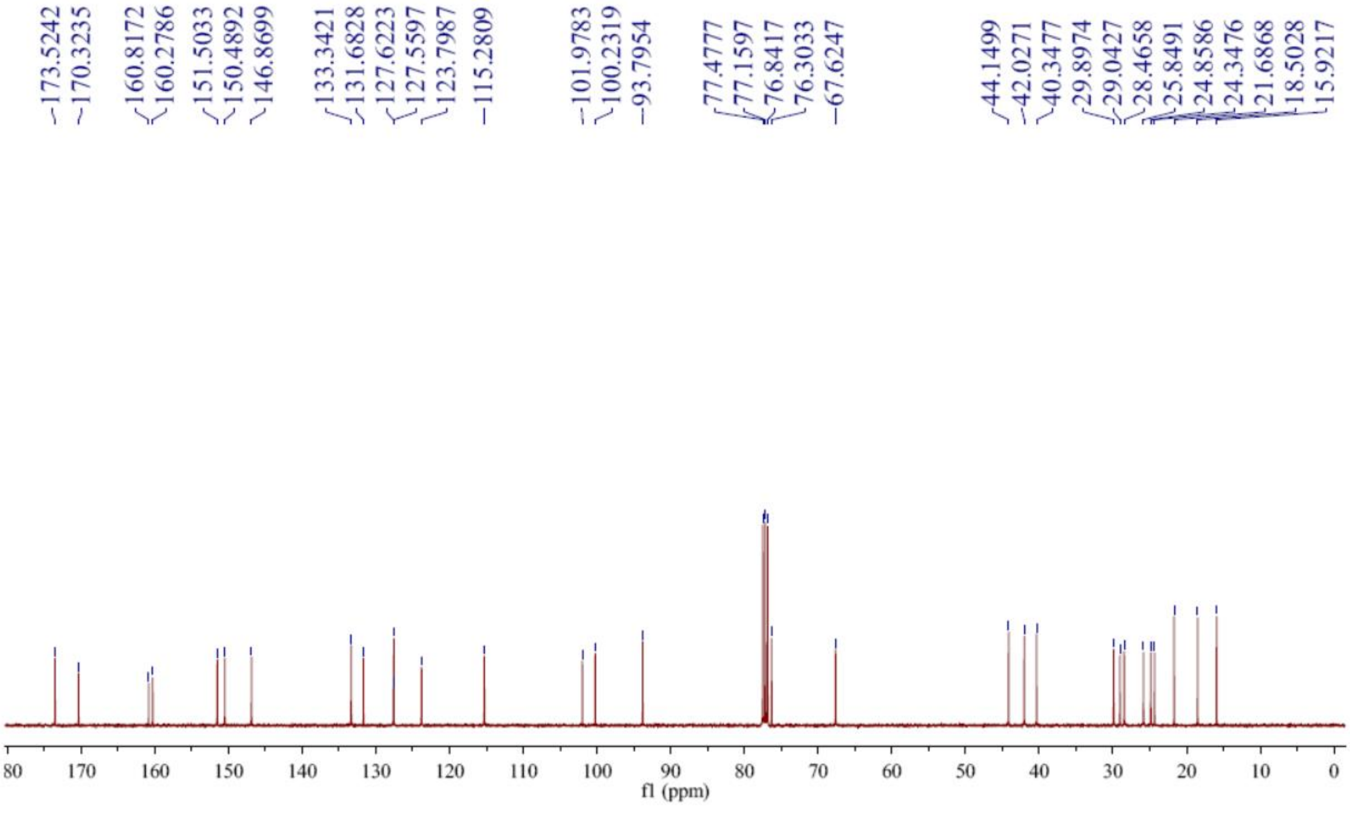

S3.85. ^{1}H NMR spectrum of **29** in $CDCl_3$

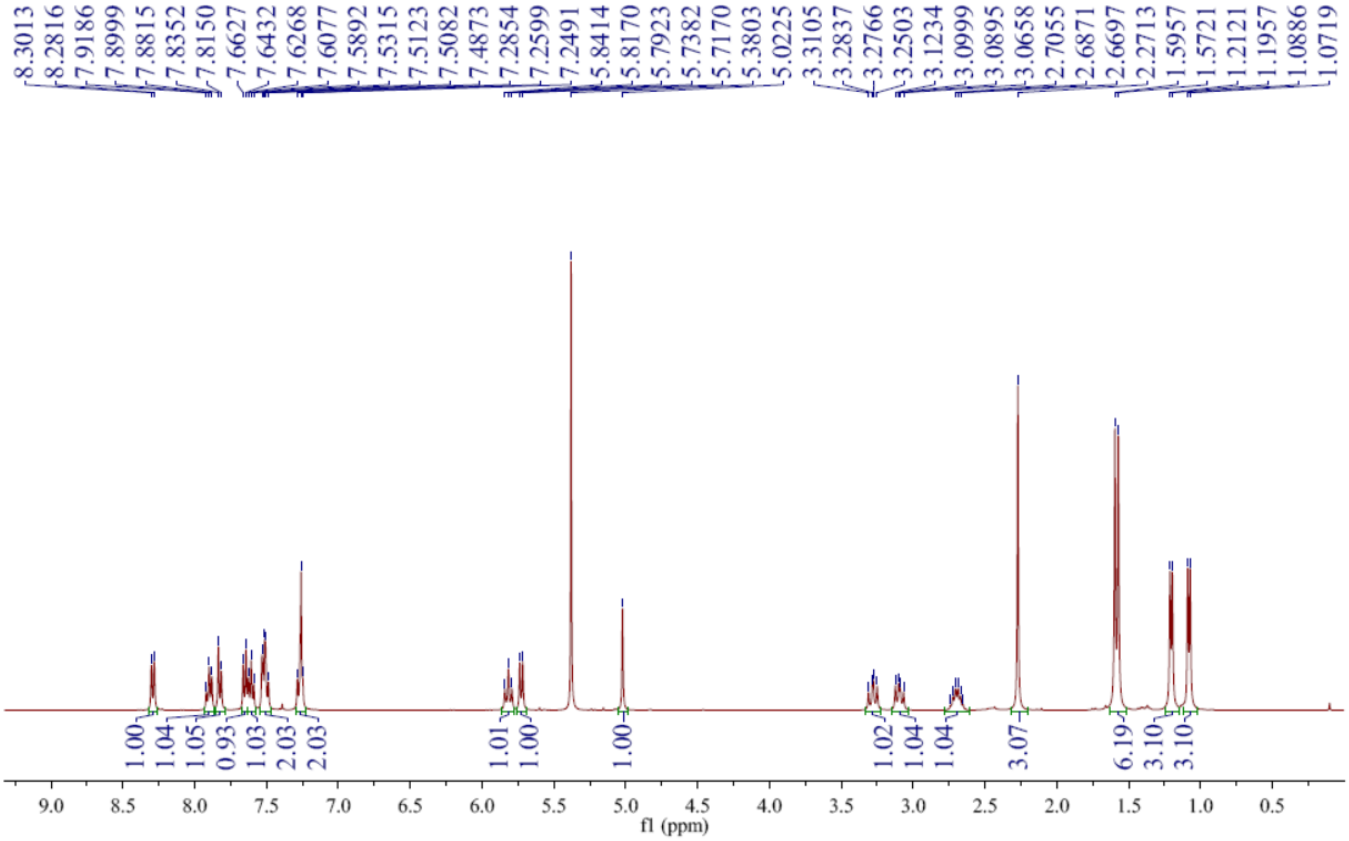

S3.86. ^{13}C NMR spectrum of **29** in $CDCl_3$

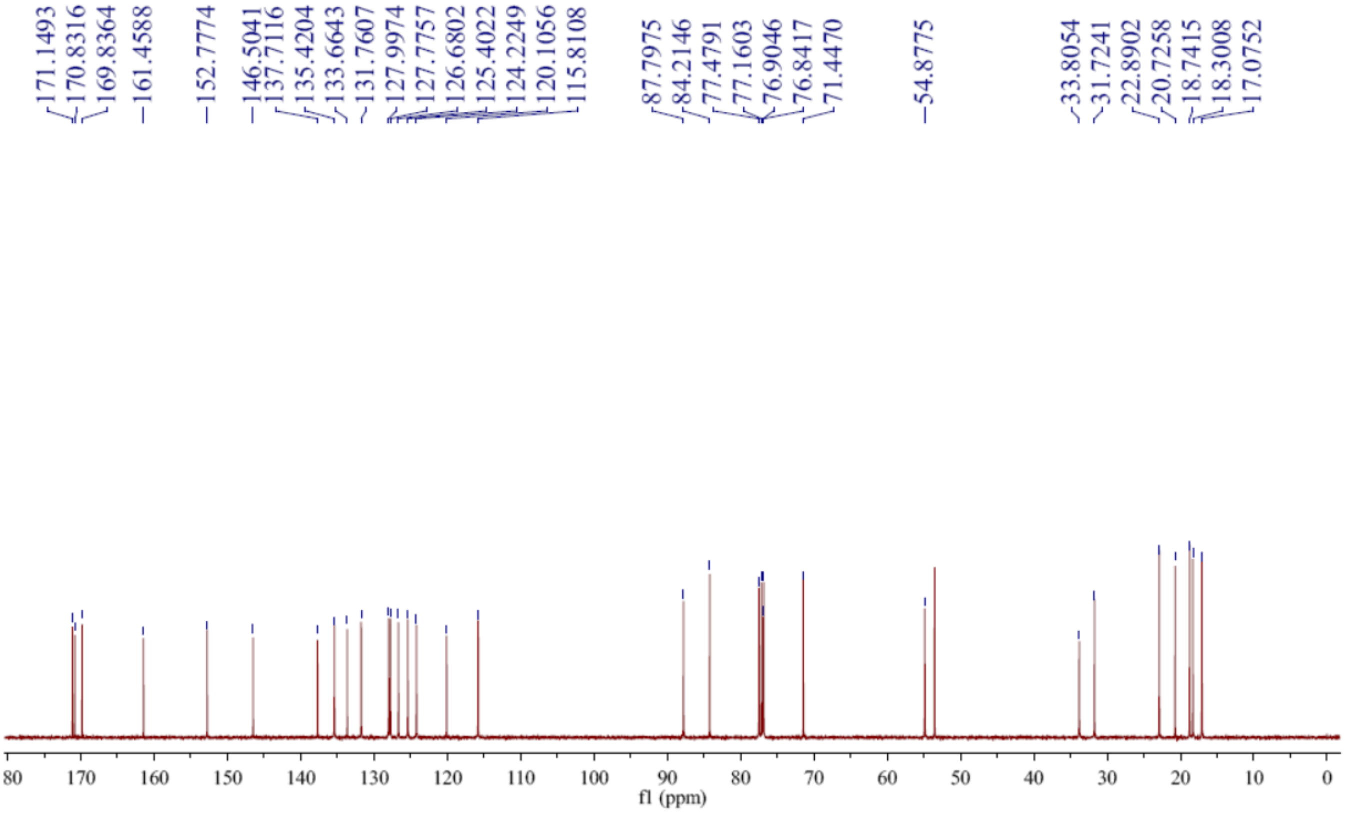

S3.87. ^{1}H NMR spectrum of **30** in $CDCl_3$

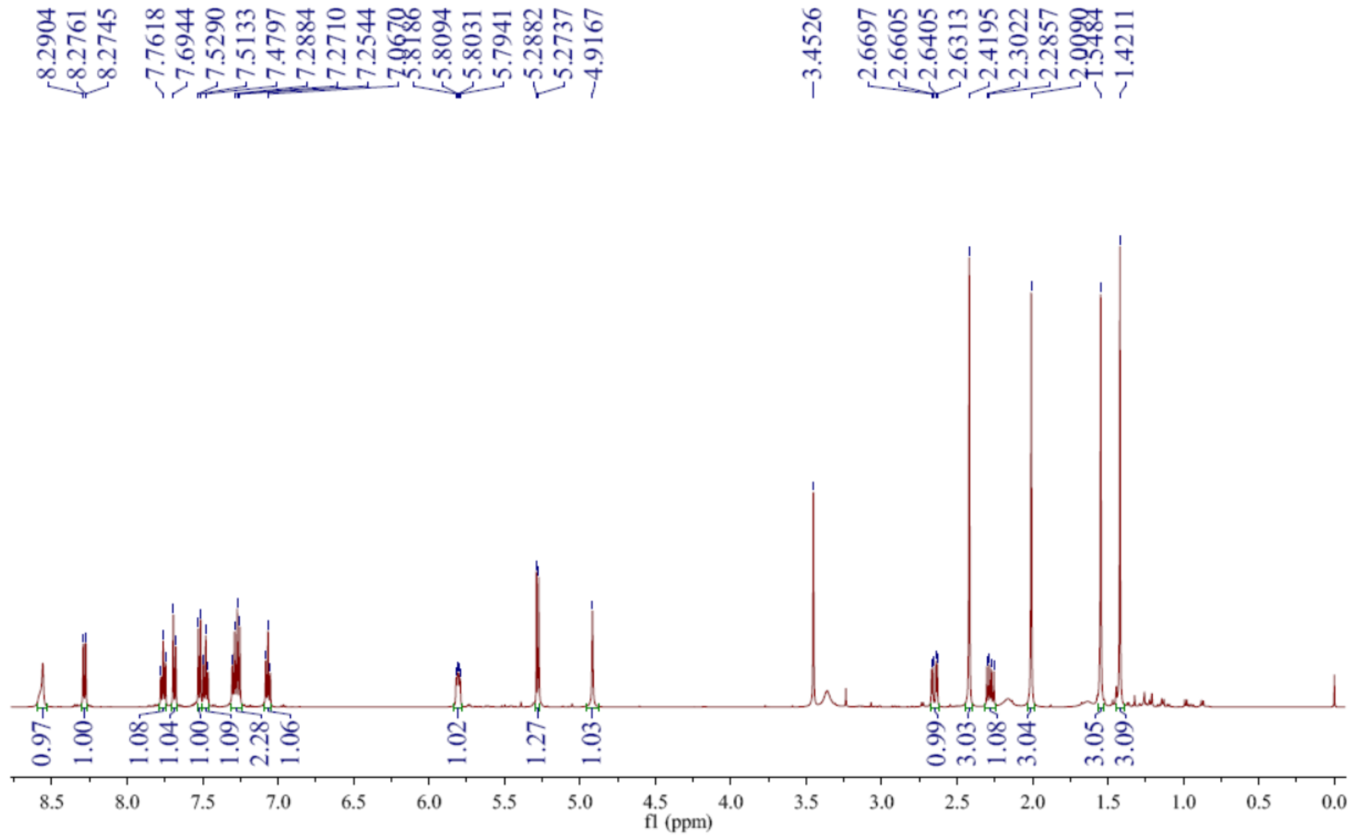

S3.88. ^{13}C NMR and DEPT spectra of **30** in $CDCl_3$

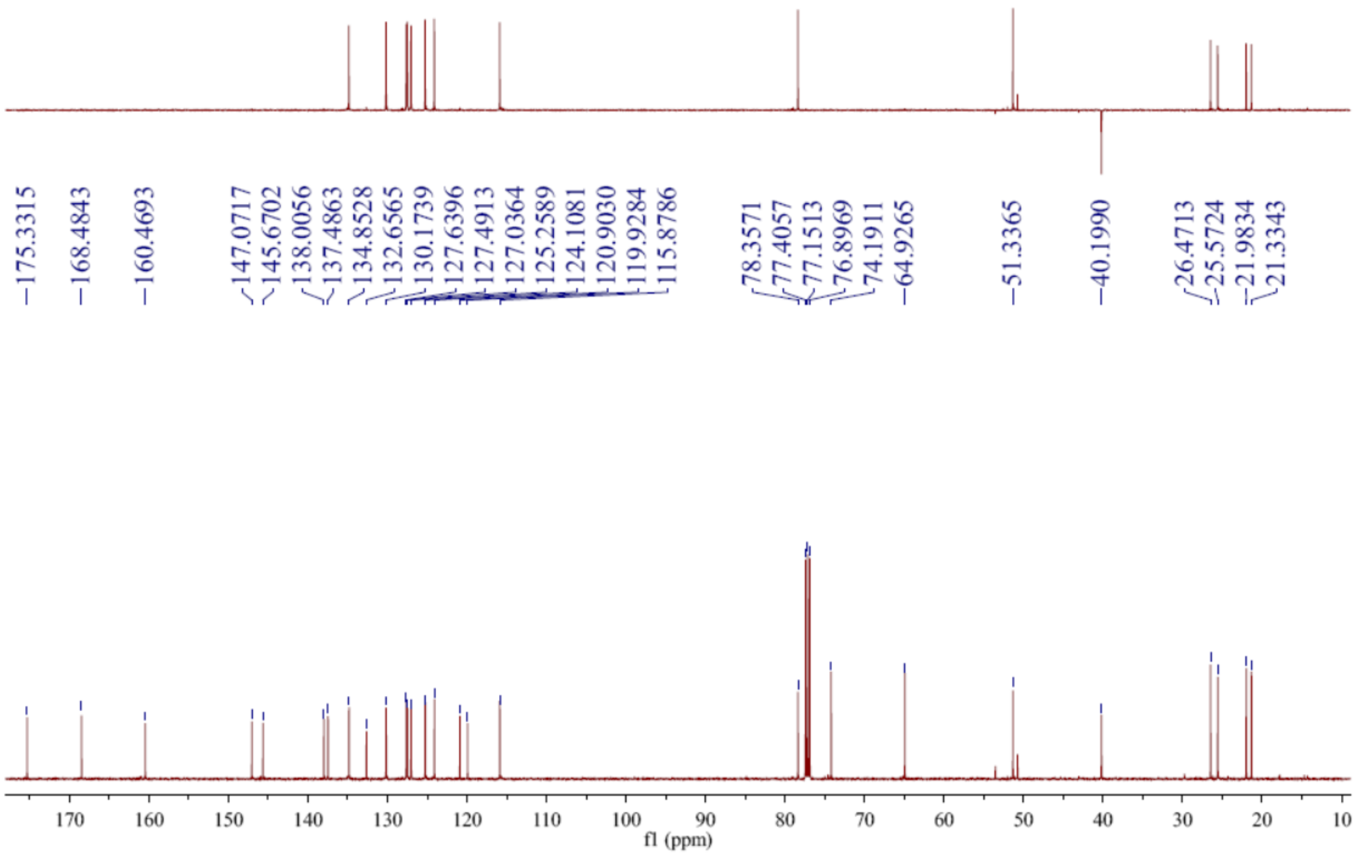

S3.89. HMBC spectrum of **30** in $CDCl_3$

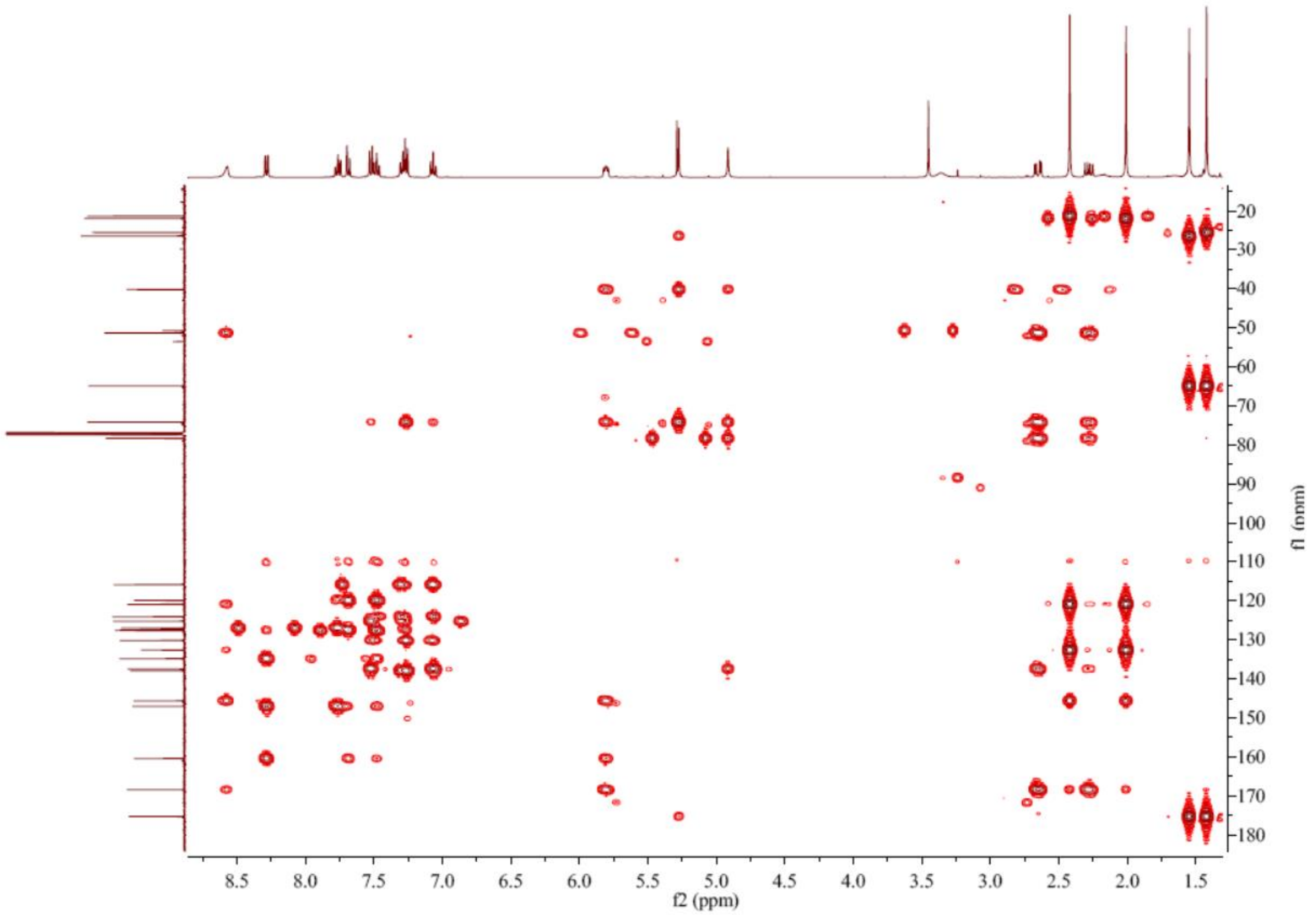

S3.90. ^{1}H NMR spectrum of **31** in pyridine-d_5

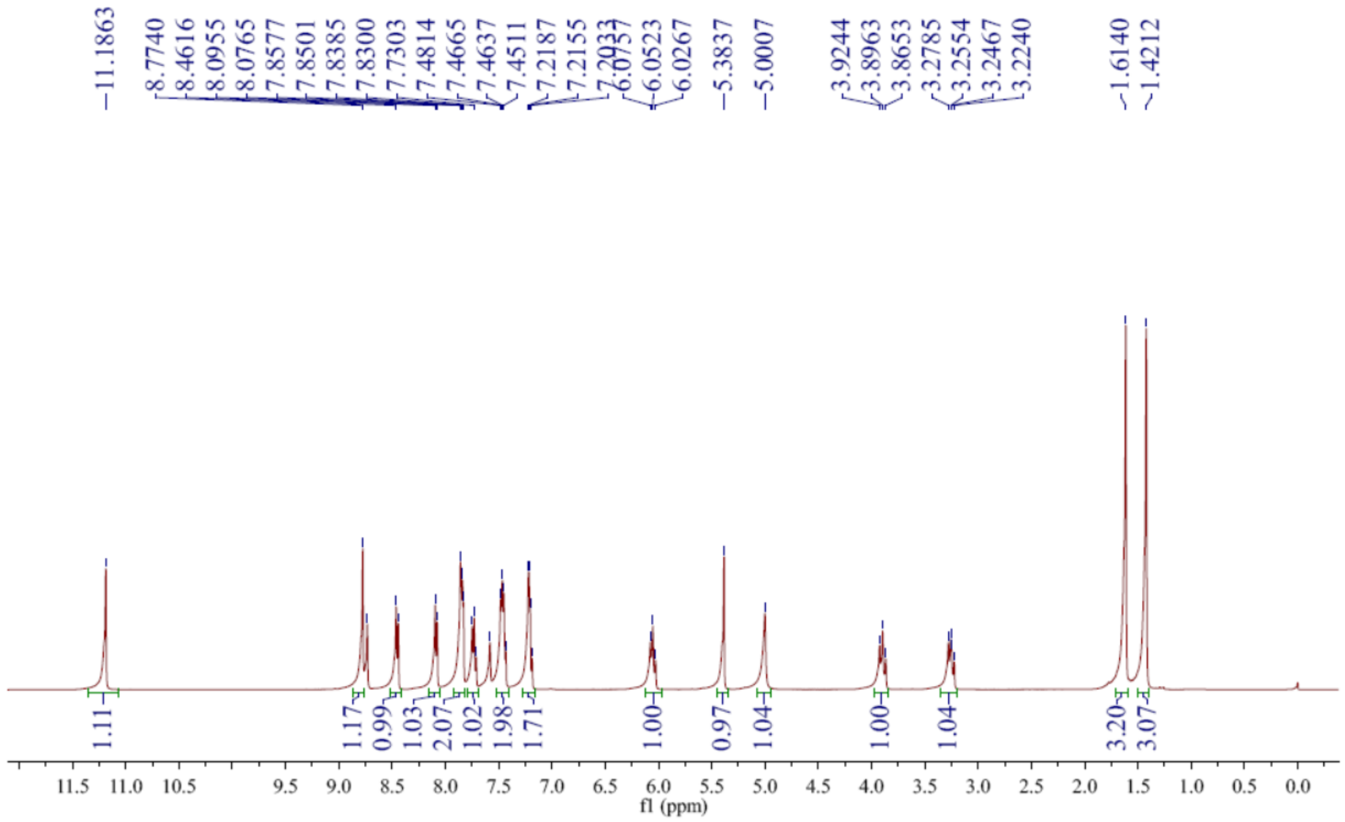

S3.91. ^{13}C NMR and DEPT spectra of **31** in pyridine-d_5

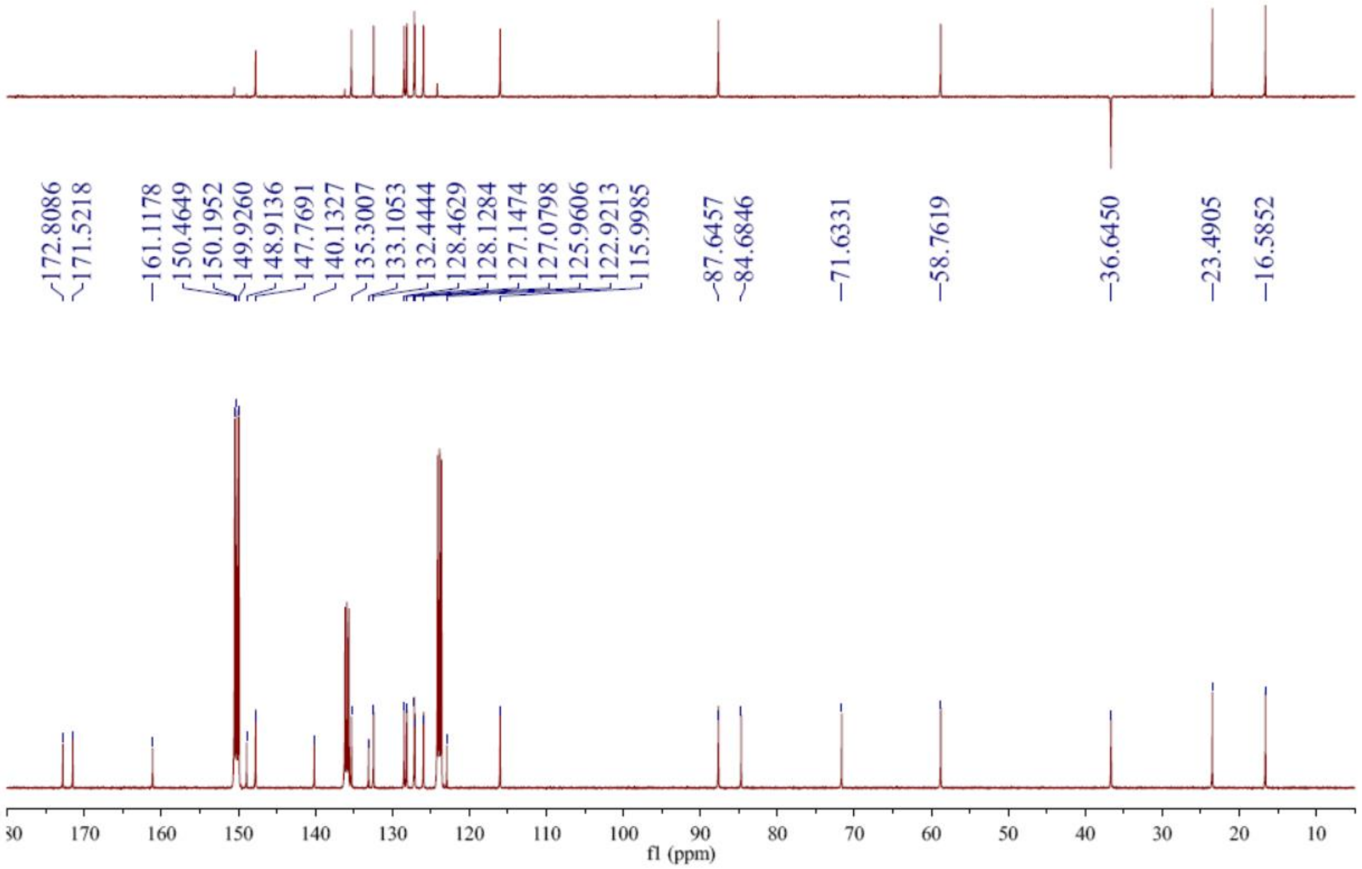

S3.92. ^{1}H NMR spectrum of **32** in CD_3OD + $CDCl_3$

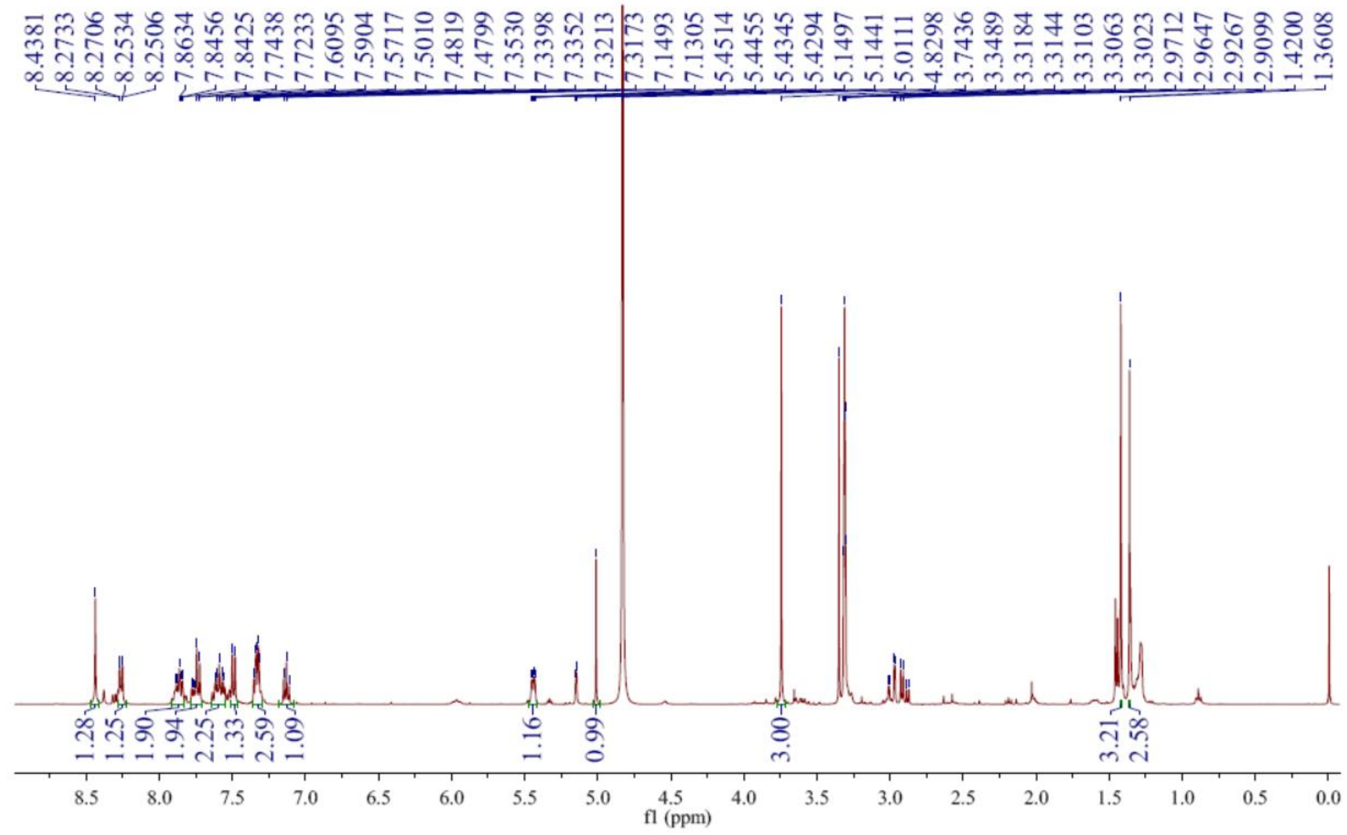

S3.93. ^{13}C NMR and DEPT spectra of **32** in $CD_3OD + CDCl_3$

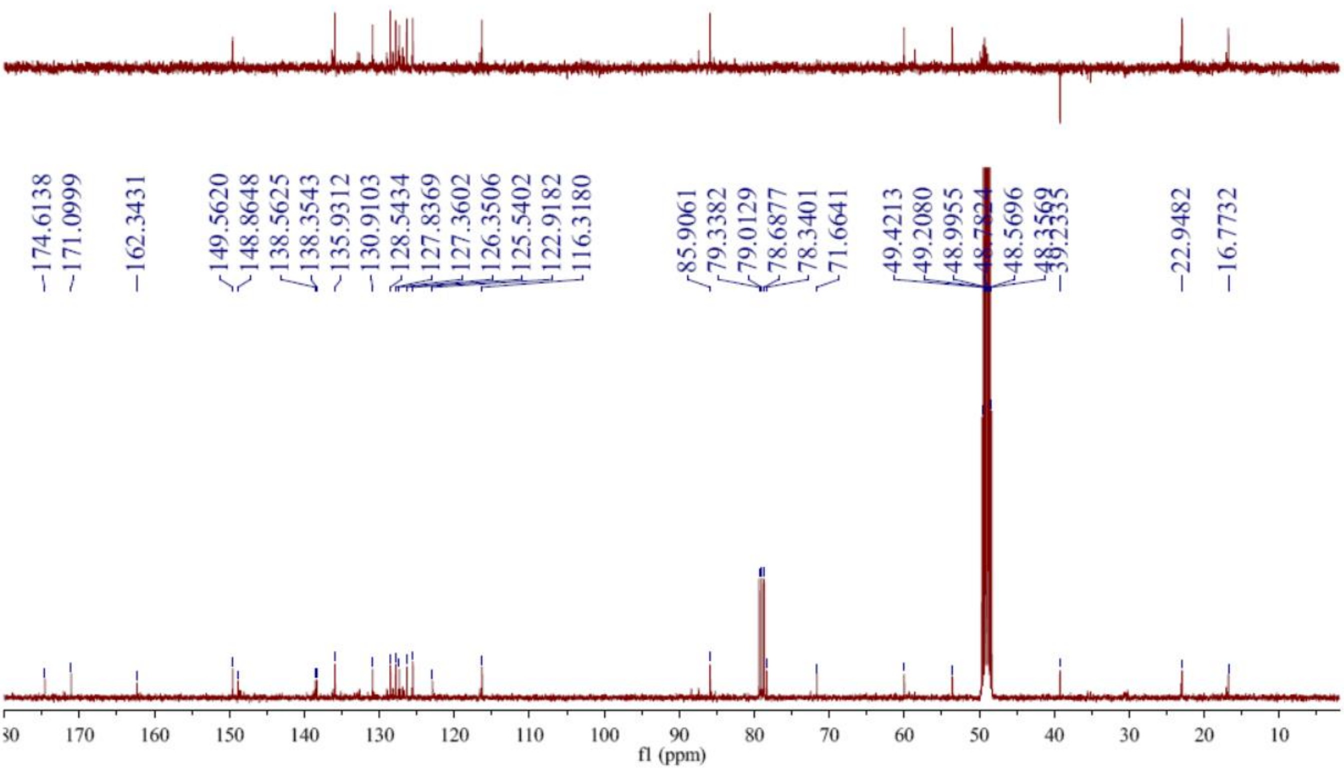

S3.94. ^{1}H NMR spectrum of **33** in $CDCl_3$

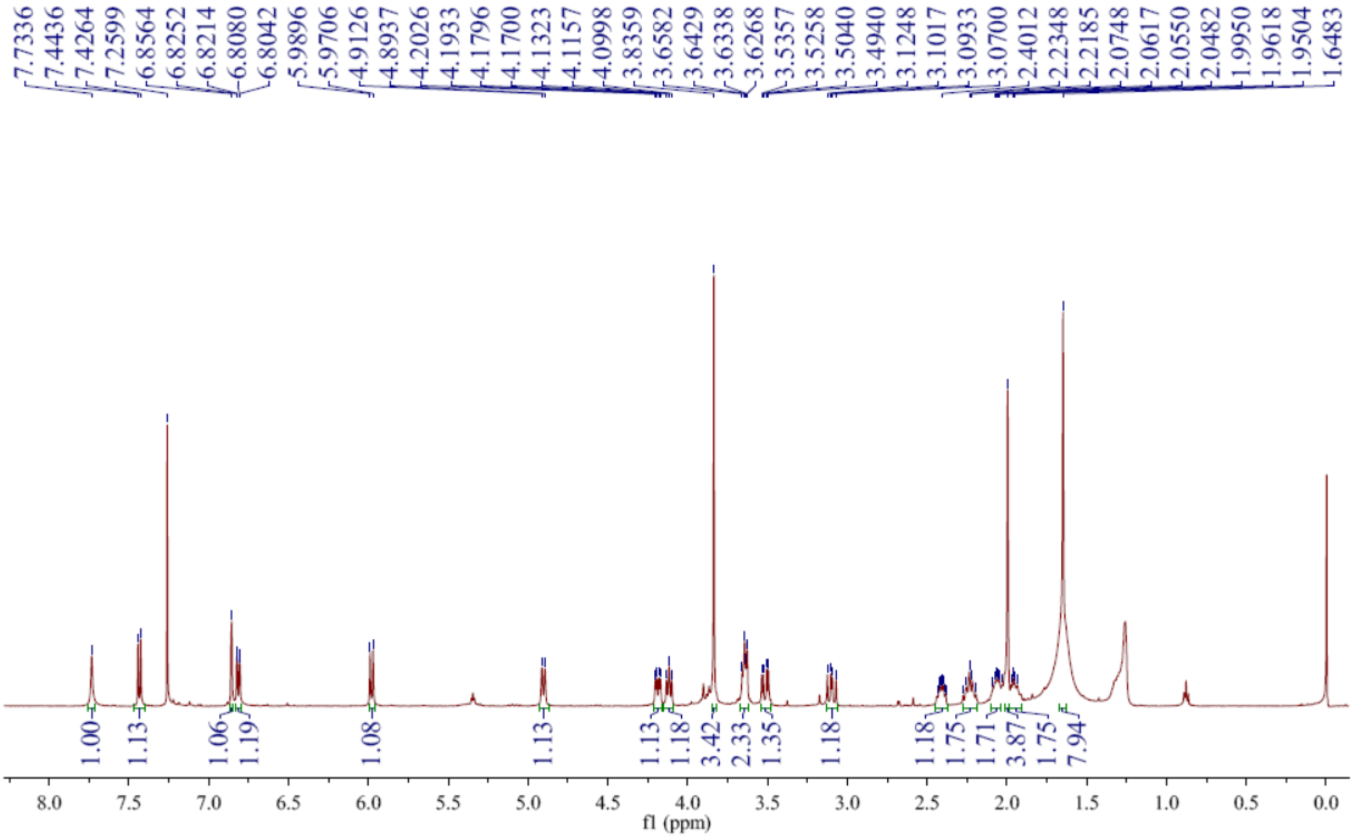

S3.95. ^{13}C NMR and DEPT spectra of **33** in $CDCl_3$

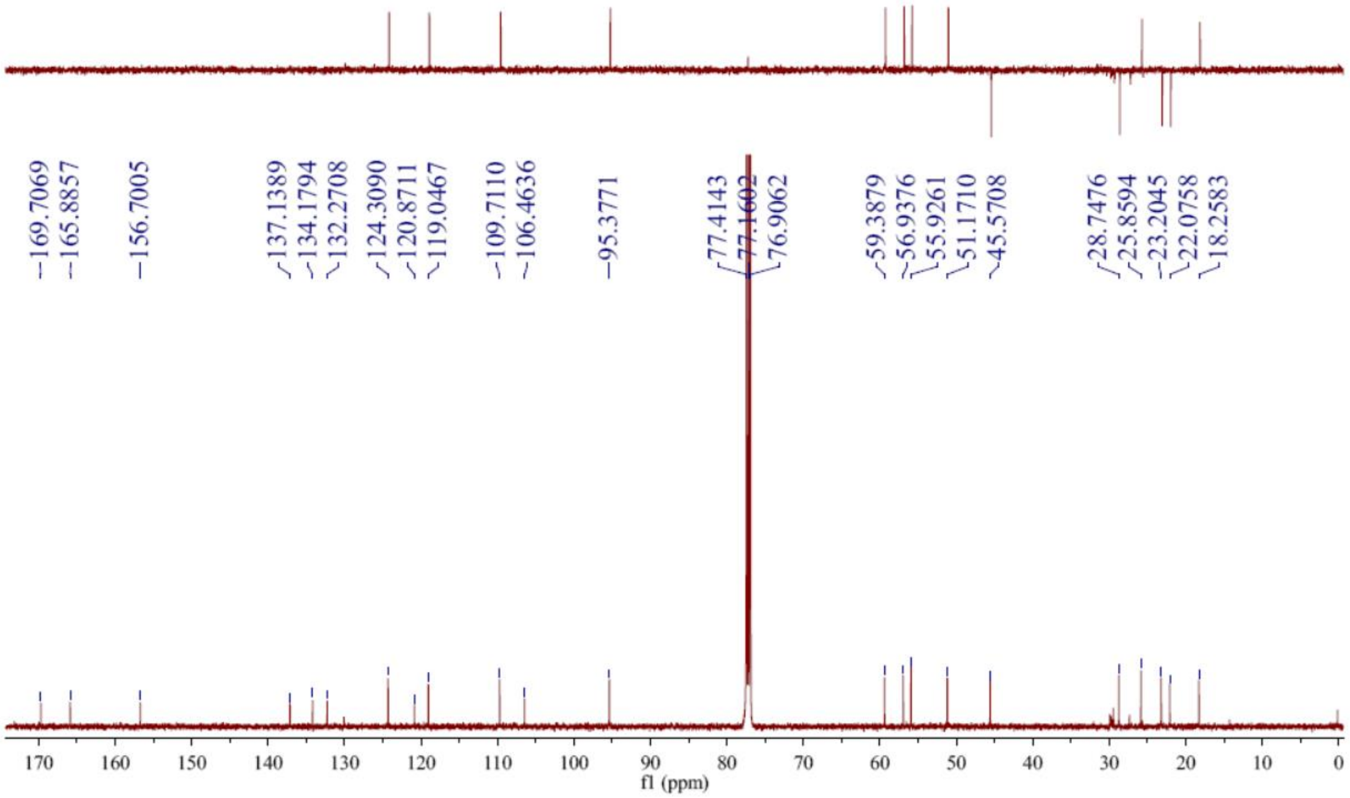

S3.96. ^{1}H NMR spectrum of **34** in CD_3OD

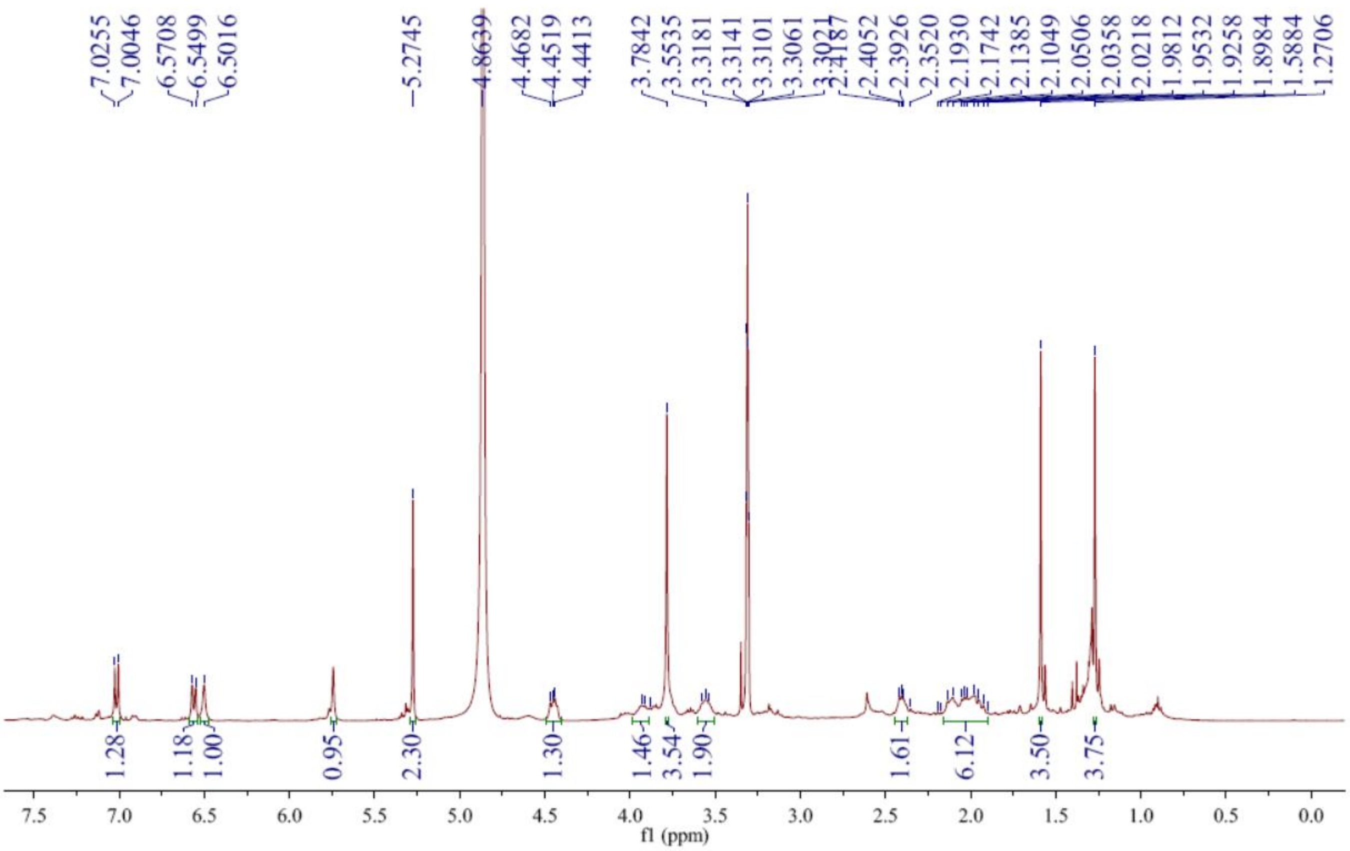

S3.97. ^{13}C NMR and DEPT spectra of **34** in CD_3OD

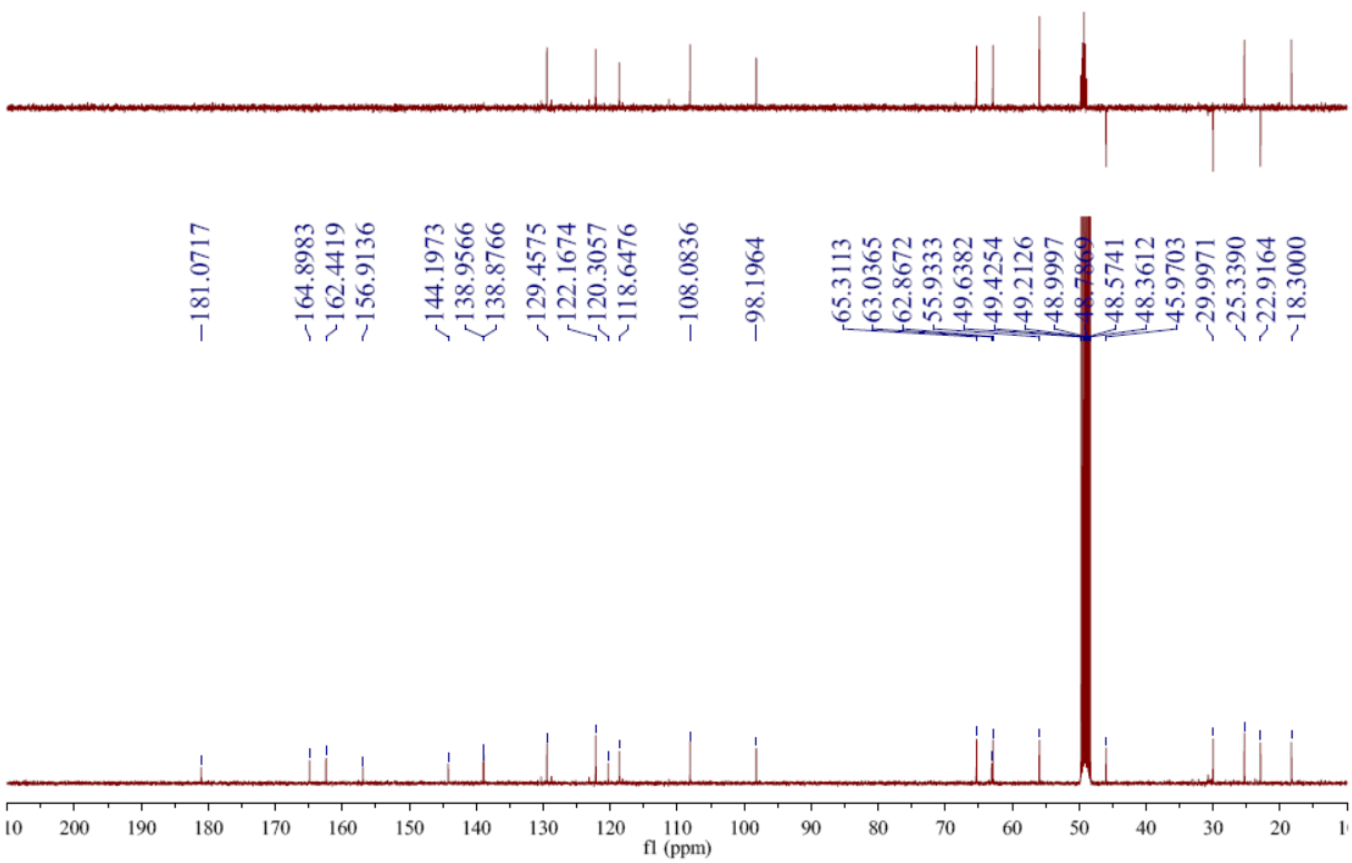

S4. HR-ESI-MS and IR spectra of **1–7**

S4.1. HR-ESI-MS spectrum of **1**

Measured region for 411.1055 m/z

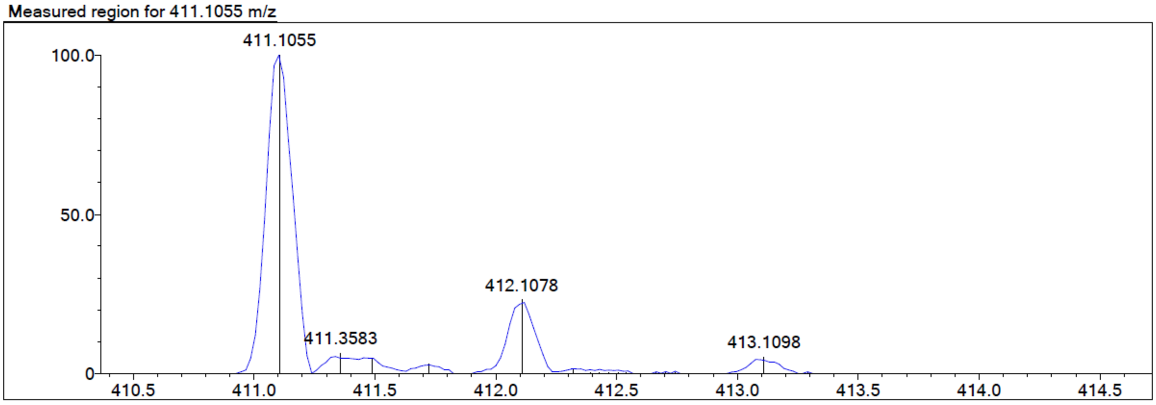

C20 H20 O8 [M+Na]+ : Predicted region for 411.1050 m/z

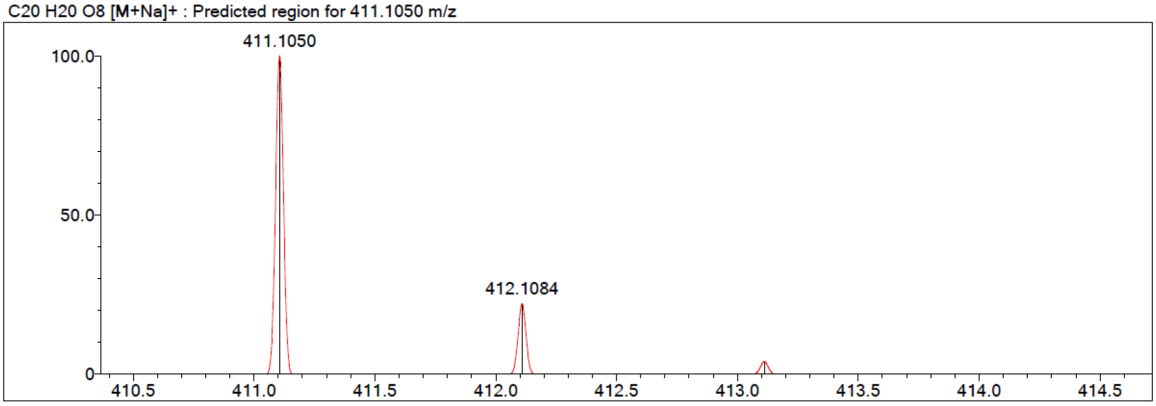

S4.2. HR-ESI-MS spectrum of **2**

Measured region for 233.0422 m/z

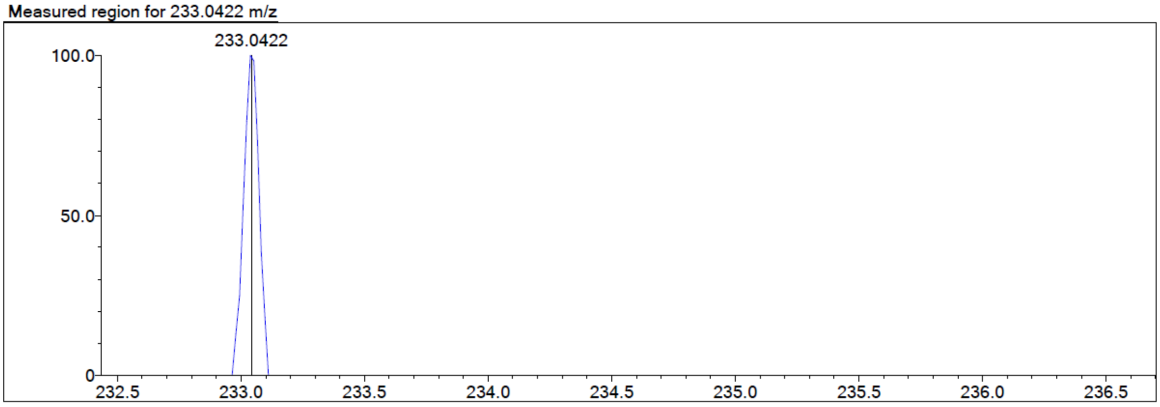

C10 H10 O5 [M+Na]+ : Predicted region for 233.0420 m/z

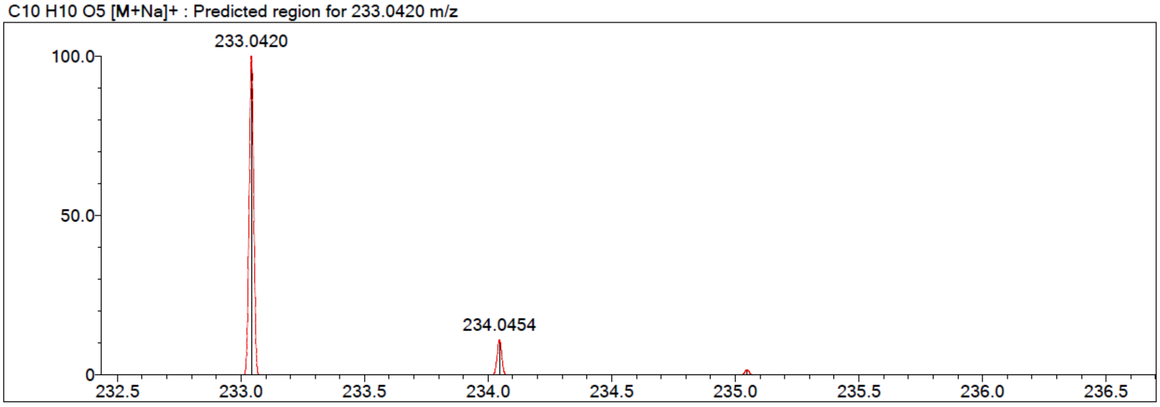

S4.3. HR-ESI-MS spectrum of **3**

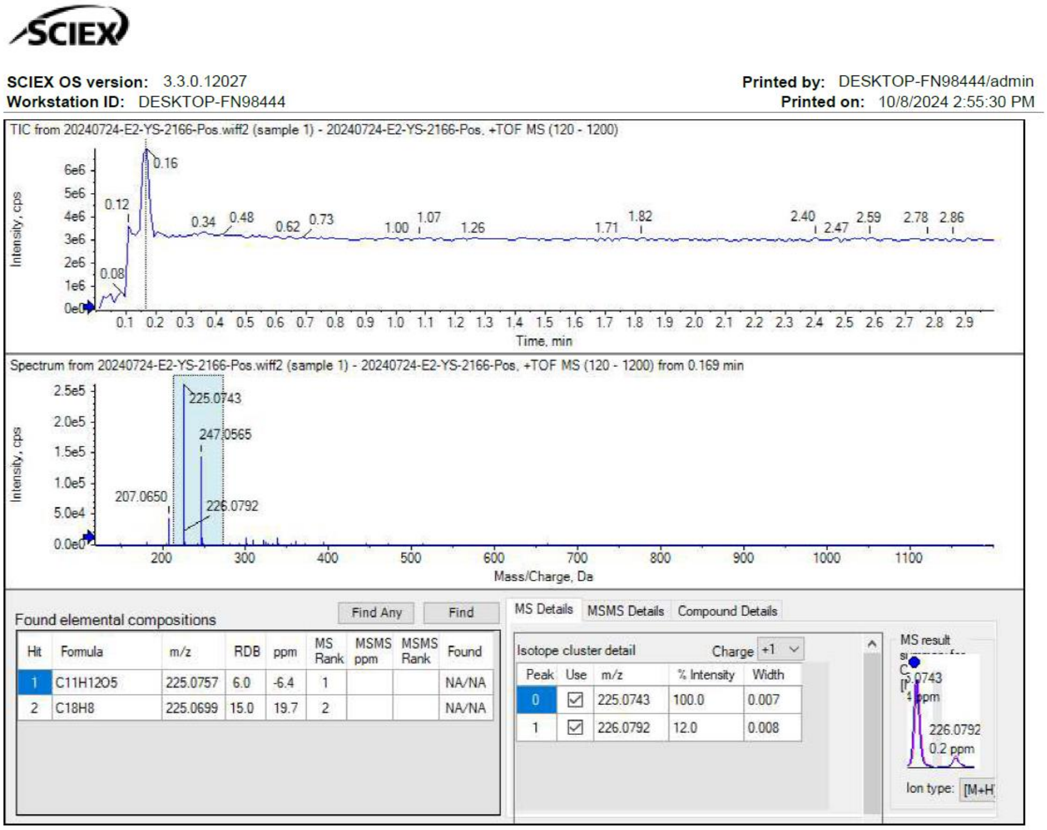

Hit	Formula	m/z	RDB	ppm	MS Rank	MSMS ppm	MSMS Rank	Found
1	C11H12O5	225.0757	6.0	-6.4	1			NA/NA
2	C18H8	225.0699	15.0	19.7	2			NA/NA

Peak	Use	m/z	% Intensity	Width
0	☑	225.0743	100.0	0.007
1	☑	226.0792	12.0	0.008

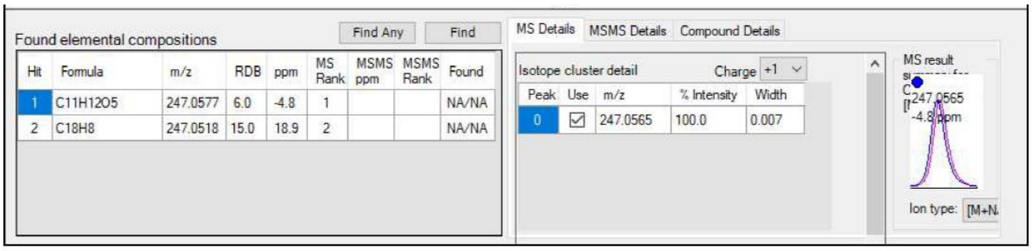

Hit	Formula	m/z	RDB	ppm	MS Rank	MSMS ppm	MSMS Rank	Found
1	C11H12O5	247.0577	6.0	-4.8	1			NA/NA
2	C18H8	247.0518	15.0	18.9	2			NA/NA

Peak	Use	m/z	% Intensity	Width
0	☑	247.0565	100.0	0.007

S4.4. HR-ESI-MS spectrum of **4**

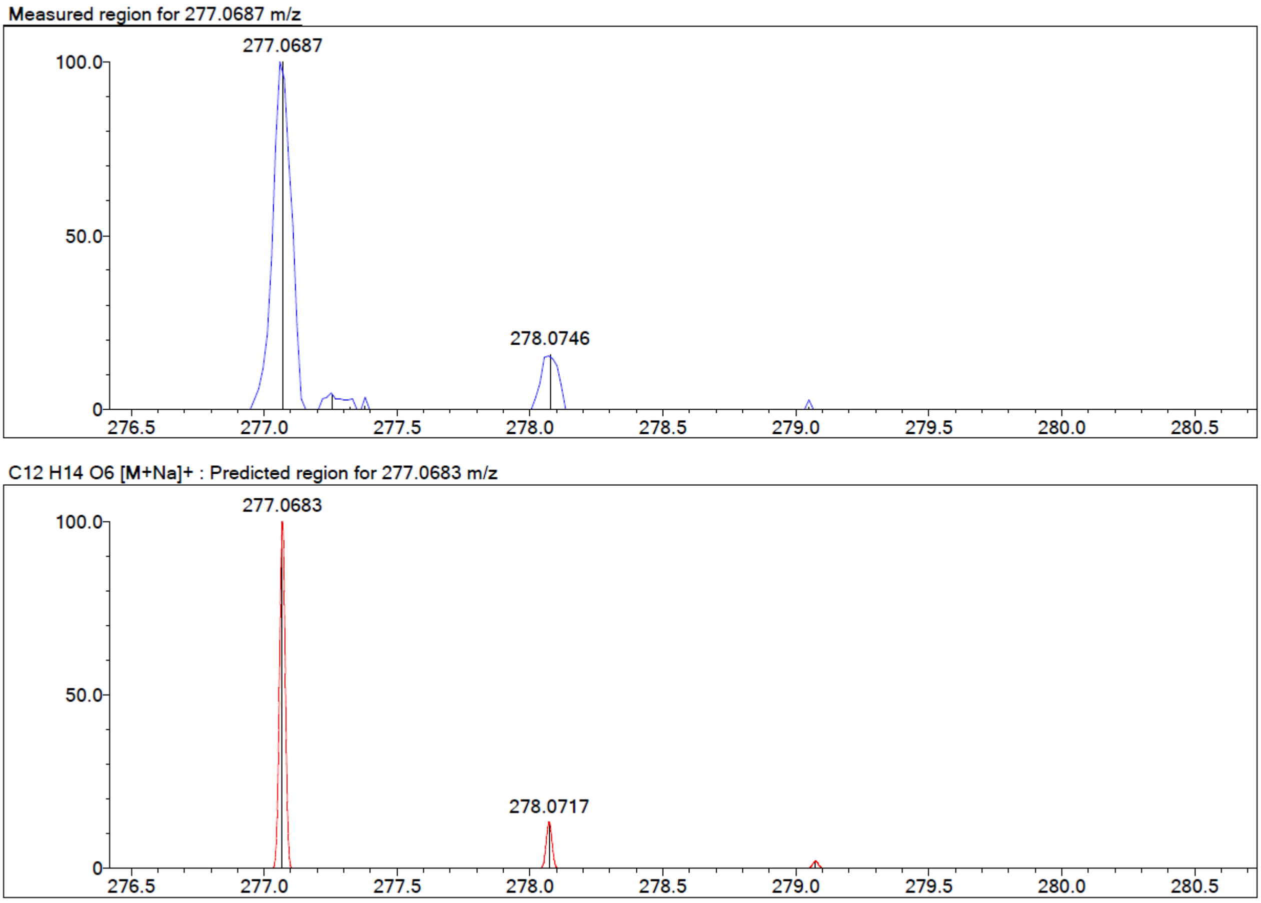

S4.5. HR-ESI-MS spectrum of **5**

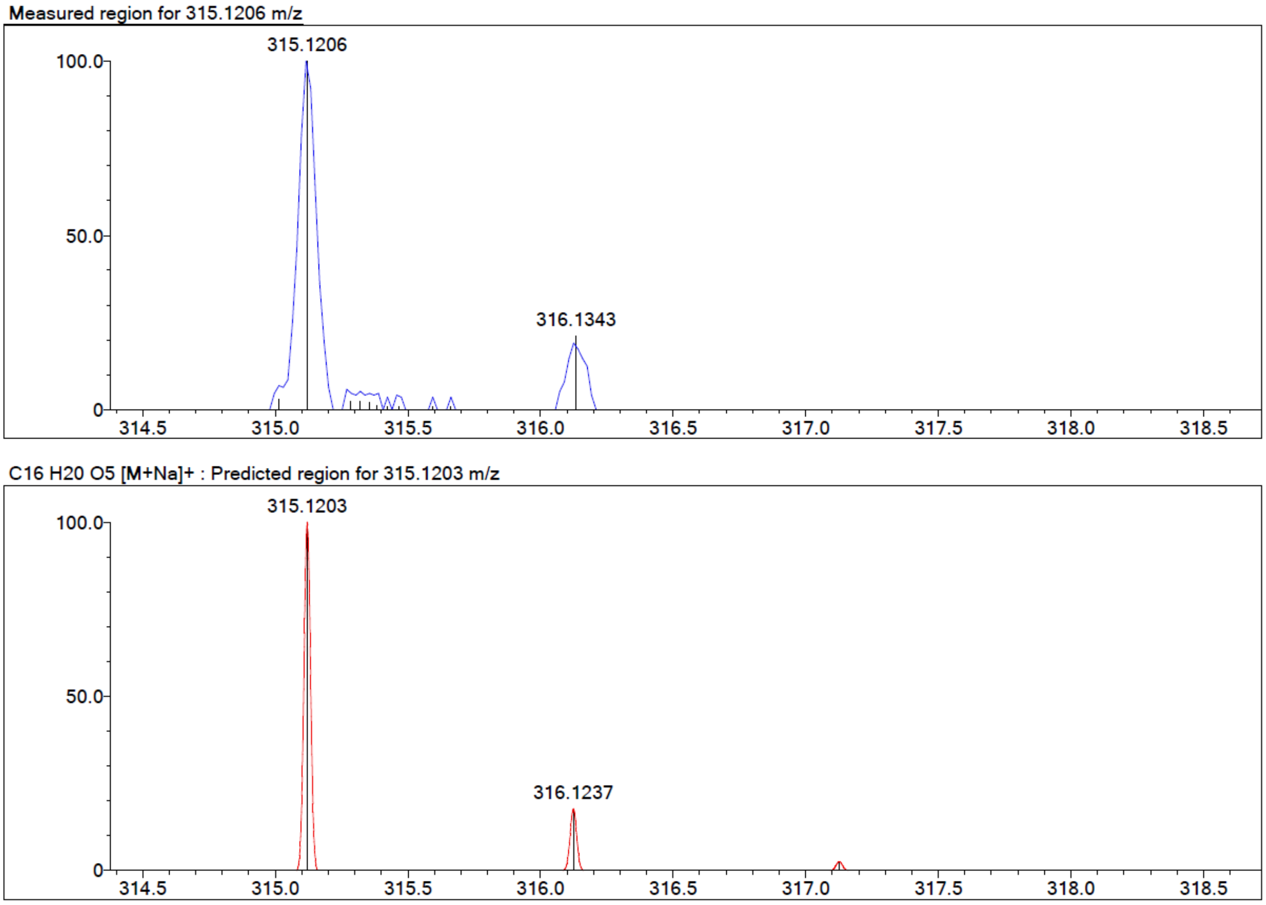

S4.6. HR-ESI-MS spectrum of **6**

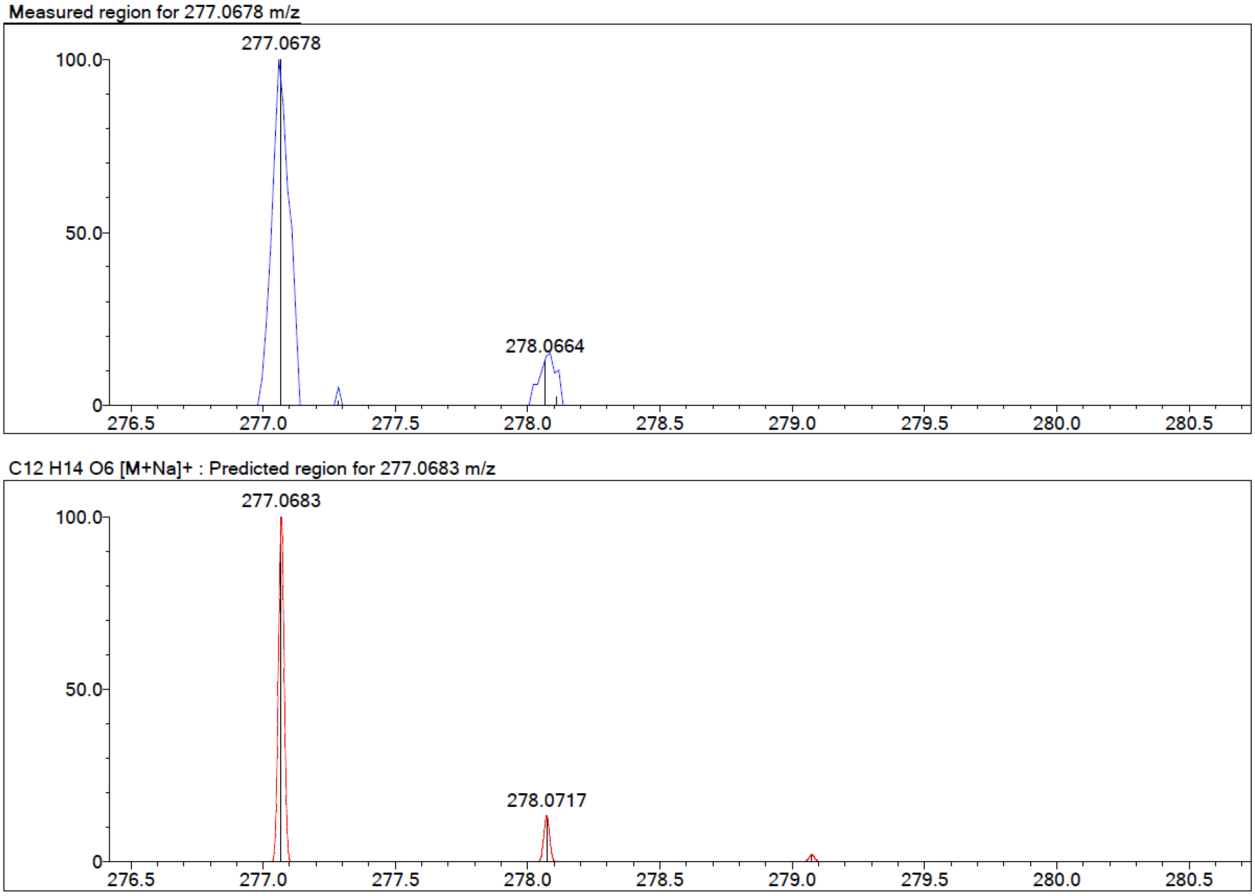

S4.7. HR-ESI-MS spectrum of **7**

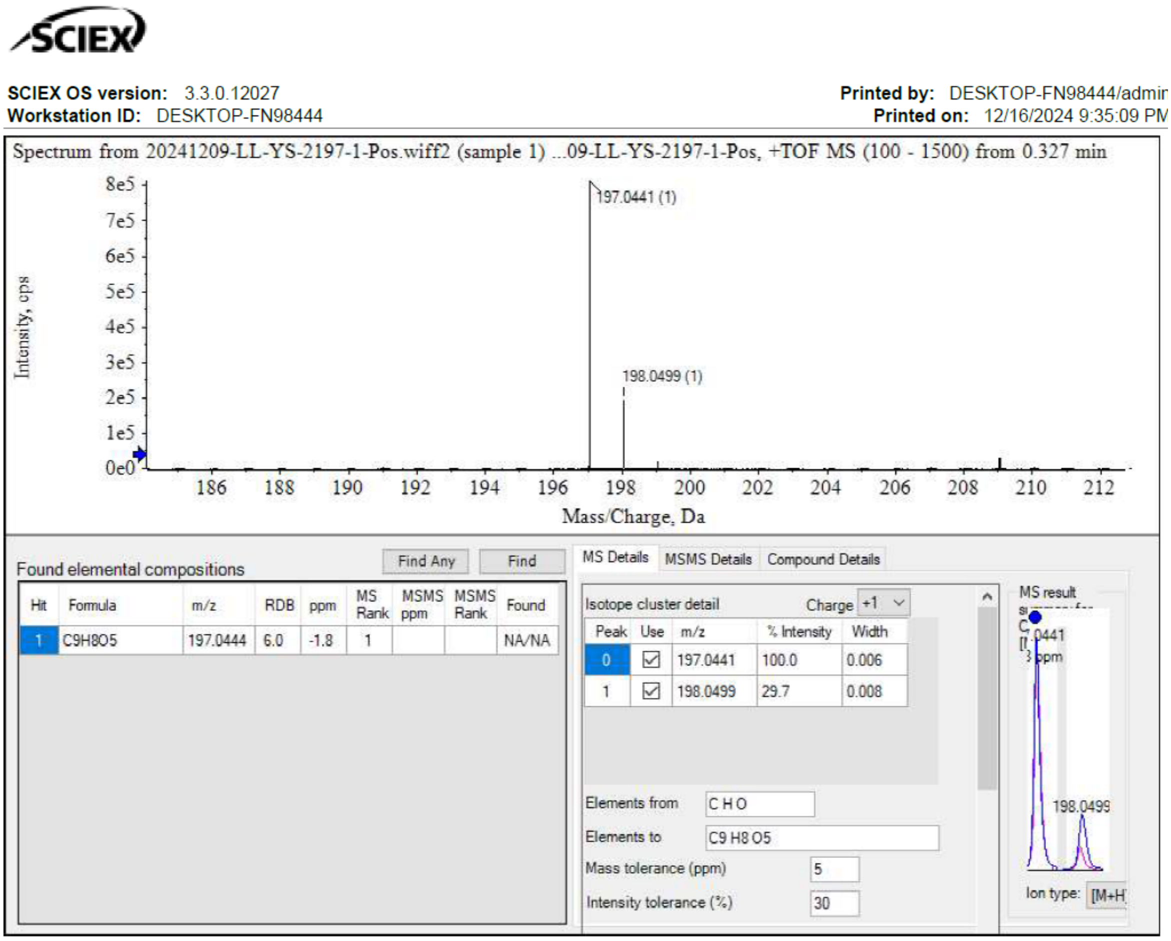

Supplement: Supplementary file 1 — The NMR of 1–34, ECD and HR-ESI-MS spectra of new compounds, 1D NMR spectroscopic data for 7–34, and ECD calculation for 2 and 4. [file 13659_2025_503_MOESM1_ESM.pdf]
